# Soaring Systematics: An Evaluation of Biogeography and Flight Behavior in Dragonflies and Damselflies (Insecta: Odonata) Using Phylogenomics

**DOI:** 10.1093/sysbio/syag005

**Published:** 2026-01-22

**Authors:** Lacie G Newton, John C Abbott, Seth M Bybee, Payton Carter, Paul B Frandsen, Aaron Goodman, Robert Guralnick, Brittney Hahn, Jacob Idec, Vincent J Kalkman, Manpreet Kolhi, Judicaël Fomekong-Lontchi, Pungki Lupiyaningdyah, Violet Onsongo, Emma Rowe, Melissa Sanchez-Herrera, Stefan Pinkert, Laura Sutherland, Ethan Tolman, Rhema Uche-Dike, Phil Barden, Michael Belitz, Cornelio A Bota-Sierra, Adolfo Cordero-Rivera, Alex Córdoba-Aguilar, Klaas-Douwe B Dijkstra, Rory A Dow, Juliana Ehlert, Rhainer G Ferreira, Matti Hämäläinen, Leandro Juen, M Olalla Lorenzo-Carballa, Bill Mauffray, Anne L Nielsen, Pablo Pessacq, Thai Hong Pham, Ângelo Parise Pinto, Stephen J Richards, Ruth Salas, Jeffrey H Skevington, Gunther Theischinger, Haomiao Zhang, Jessica L Ware

**Affiliations:** Division of Invertebrate Zoology, American Museum of Natural History, 200 Central Park West, New York, NY 10024, USA; Department of Biological Sciences, New Jersey Institute of Technology, 337 Central King Building, University Heights, Newark, NJ 07102, USA; Alabama Museum of Natural History, Department of Research and Collections, The University of Alabama, 427 Sixth Avenue, Tuscaloosa, AL 35487, USA; Department of Biology and Monte L. Bean Museum, Brigham Young University, 645 E Phillips Lane, Provo, UT 84604, USA; Department of Biology and Monte L. Bean Museum, Brigham Young University, 645 E Phillips Lane, Provo, UT 84604, USA; Department of Plant and Wildlife Sciences and Monte L. Bean Museum, Brigham Young University, 645 E Phillips Lane, Provo, UT 84604, USA; Division of Invertebrate Zoology, American Museum of Natural History, 200 Central Park West, New York, NY 10024, USA; Florida Museum of Natural History, Department of Natural History, University of Florida, 3215 Hull Road, Gainesville, FL 32611, USA; Division of Invertebrate Zoology, American Museum of Natural History, 200 Central Park West, New York, NY 10024, USA; Florida Museum of Natural History, Department of Natural History, University of Florida, 3215 Hull Road, Gainesville, FL 32611, USA; Naturalis Biodiversity Center, Darwinweg 2, 2333 CR Leiden, The Netherlands; Division of Invertebrate Zoology, American Museum of Natural History, 200 Central Park West, New York, NY 10024, USA; Baruch College, The City University of New York, 1 Bernard Baruch Way, New York, NY 10010, USA; Department of Biology and Monte L. Bean Museum, Brigham Young University, 645 E Phillips Lane, Provo, UT 84604, USA; Department of Biology and Monte L. Bean Museum, Brigham Young University, 645 E Phillips Lane, Provo, UT 84604, USA; Research Center for Biosystematics and Evolution, Research Organization of Life Sciences, National Research and Innovation Agency (BRIN), Kawasan Sains Teknologi Dr. (H.C.) Ir. H. Soekarno, Jl. Raya Jakarta-Bogor No. Km. 46, Cibinong, West Java, 16911, Indonesia; Division of Invertebrate Zoology, American Museum of Natural History, 200 Central Park West, New York, NY 10024, USA; Department of Biology and Monte L. Bean Museum, Brigham Young University, 645 E Phillips Lane, Provo, UT 84604, USA; Alabama Museum of Natural History, Department of Research and Collections, The University of Alabama, 427 Sixth Avenue, Tuscaloosa, AL 35487, USA; Department of Conservation Ecology, University of Marburg, Karl-von-Frisch-Straße 8, 35043 Marburg, Germany; Department of Biology and Monte L. Bean Museum, Brigham Young University, 645 E Phillips Lane, Provo, UT 84604, USA; Division of Invertebrate Zoology, American Museum of Natural History, 200 Central Park West, New York, NY 10024, USA; Department of Biological Sciences, Virginia Tech, Derring Hall, Room 2125, 926 West Campus Drive, Mail Code 0406, Blacksburg, VA 24061, USA; Division of Invertebrate Zoology, American Museum of Natural History, 200 Central Park West, New York, NY 10024, USA; Department of Biological Sciences, New Jersey Institute of Technology, 337 Central King Building, University Heights, Newark, NJ 07102, USA; Department of Integrative Biology, Michigan State University, Natural Science Building, 288 Farm Ln #203, East Lansing, MI 48824, USA; Grupo de Entomología Universidad de Antioquia (GEUA), Universidad de Antioquia, Calle 67 # 53 - 108 (Bloque 7), Medellin, Antioquia, 050010, Colombia; Grupo de Ecoloxía Evolutiva e da Conservación, Departamento de Ecoloxía e Bioloxía Animal, Universidade de Vigo. EUET Forestal, Campus Universitario, 36005 Pontevedra, Galiza, Spain; Instituto de Ecología, Universidad Nacional Autónoma de México, Ciudad Universitaria, Coyoacán, 04510 Mexico City, Mexico; Naturalis Biodiversity Center, Darwinweg 2, 2333 CR Leiden, The Netherlands; Naturalis Biodiversity Center, Darwinweg 2, 2333 CR Leiden, The Netherlands; Institute of Biodiversity and Environmental Conservation, Universiti Malaysia Sarawak, 94300 Kota Samarahan, Sarawak, Malaysia; Department of Zoology, Federal University of Paraná, Avenida Coronel Francisco Heráclito dos Santos, 100, Jardim das Américas, Curitiba - PR, CEP 81530-900, Brazil; Departamento de Ciências Biológicas, Federal University of Triangulo Mineiro, Rua Vigário Carlos, 100, Bairro Abadia, Uberaba, MG, CEP 38025-350, Brazil; Naturalis Biodiversity Center, Darwinweg 2, 2333 CR Leiden, The Netherlands; Institute of Biological Sciences (ICB), Federal University of Para, Av. Perimetral, 2-224 - Guamá, Belém - PA, 66077-830, Brazil; Department of Zoology, Genetics and Physical Anthropology, Universidade de Santiago de Compostela, Rúa Lope Gómez de Marzoa, s/n, 15782 Santiago de Compostela, Spain; Florida State Collection of Arthropods, 1911 SW 34th St, Gainesville, FL 32608, USA; Department of Entomology, Rutgers University, 96 Lipman Dr, New Brunswick, NJ 08901, USA; Laboratorio de Investigaciones en Ecología y Sistemática Animal, Sarmiento 849, 9200 Esquel, Chubut, Argentina; Vietnam National Museum of Nature, 18 Hoang Quoc Viet Street, Cau Giay District, Hanoi, Vietnam; Department of Zoology, Federal University of Paraná, Avenida Coronel Francisco Heráclito dos Santos, 100, Jardim das Américas, Curitiba - PR, CEP 81530-900, Brazil; South Australian Museum, North Terrace, Adelaide, South Australia, 5000, Australia; Division of Invertebrate Zoology, American Museum of Natural History, 200 Central Park West, New York, NY 10024, USA; Department of Biology, Carleton University, 1125 Colonel By Drive, Ottawa, Ontario, K1S 5B6, Canada; Australian Museum Sydney, 1 William Street, Darlinghurst, Sydney, NSW 2010, Australia; Kunming Natural History Museum of Zoology, Kunming Institute of Zoology, Chinese Academy of Sciences, No. 32, Jiaochang East Road, Wuhua District, Kunming Yunnan Province, 650223, China; Division of Invertebrate Zoology, American Museum of Natural History, 200 Central Park West, New York, NY 10024, USA

**Keywords:** Biogeography, diversification, flight behavior, molecular dating, odonata, phylogenomics, wing morphology

## Abstract

Dragonflies and damselflies (Insecta: Odonata) are descended from what were most likely the first winged animals, which flew ~320 million years ago (Ma). They comprise ~6400 extant species distributed across all continents except Antarctica. Examination of long-standing hypotheses regarding the role of flight behavior and wing morphology in shaping the global distribution of odonates has been limited by spatial and taxonomic scope. Here, we leverage mobilized trait and distribution data derived from specimens and literature combined with a uniquely comprehensive target-enriched phylogeny (~940 loci) covering all families and 67% of recognized genera. Ancestral state reconstruction of flight behavior strategies (“flyer” vs. “percher”) suggests the odonate ancestor was a flyer, spending a majority of its time when active on the wing, with multiple independent transitions to percher. Several transitions back to the flyer behavior have also occurred. Aspect ratios for forewings and hindwings showed a strong relationship between these traits and perching and flying behavioral strategies. Divergence time estimation suggests the crown age of Odonata to be 290–325 Ma. Bayesian biogeographical evolutionary analysis of nine biogeographical realms provides a preliminary biogeographical history for odonates spanning 325 Ma. Key family-level splits occurred during the Jurassic and Cretaceous, paralleling the increasing isolation of landmasses and the poleward drift of the contemporary Australasian and Holarctic regions. Both behavioral and morphological adaptations likely facilitated the distributional success of select odonate lineages. This study lays the foundation for a revised classification of odonates and a more complete understanding of the influence of flight behavior and wing morphology in relation to evolutionary processes shaping past and current odonate diversity.

The order Odonata, comprising dragonflies and damselflies, represents one of the earliest diverging lineages of winged animals, with a fossil record dating back ~300 million years (e.g., Misof et al. 2014; Nel and Piney 2022; Nel and Piney 2023). The group consists of >6400 extant species distributed globally across freshwater habitats, except for Antarctica (e.g., Corbet 1999; Kalkman et al. 2008; Sandall et al. 2022; Córdoba-Aguilar et al. 2023; Dijkstra 2025). Molecular dating suggests that this ancient lineage has persisted through dramatic climatic and geological changes, such as breakups of the supercontinents Pangea, Laurasia, and Gondwana, and the Permian–Triassic (252 Ma), and through two major mass extinction events: the Triassic–Jurassic (201 Ma) and the Cretaceous–Paleogene (66 Ma) (e.g., Misof et al. 2014; Kohli et al. 2021; Suvorov et al. 2022).

Odonates display a variety of flying capabilities, from transoceanic migrants like *Pantala flavescens* (e.g., Anderson 2009; Hobson et al. 2012; Ware et al. 2022) to narrowly endemic species such as *Amanipodagrion gilliesi* or *Heteragrion tatama*, known from a single stream (Clausnitzer 2003; Bota-Sierra and Novelo-Gutiérrez 2017). There is a need for better understanding of the interplay among dispersal dynamics (flight strategy and capacity), vicariance events, and subsequent diversification of Odonata over time. Odonata are also exclusively predatory insects in both life stages and highly mobile during their adulthood, with a phenotype and life history still largely resembling that of †Meganeuridae—the largest known insects and dominant predators during the Carboniferous (e.g., May 1982; Kukalová-Peck 2009; Nel et al. 2018). This “living fossil” status of Odonata exemplifies the resilience and evolutionary success of traits related to insect flight. However, the group still lacks a broad evolutionary understanding that integrates morphological and phylogenetic approaches to elucidate evolutionary origins, and long-term evolutionary trajectories related to key flight adaptations (Lidgard and Love 2018; Córdoba-Aguilar et al. 2023).

The role of flight behavior and wing morphology on species distributions in a wide spectrum of taxa points to mechanisms of broad eco-evolutionary relevance (Alzate and Onstein 2022). For instance, in birds, high-aspect-ratio wings (a measure of how relatively long and thin or short and wide wings are) are characteristic of long-distance migratory species (e.g., seabirds), whereas sedentary species typically exhibit lower aspect ratios (Beauchamp 2023). Similarly, in stoneflies, species with longer wings tend to have larger ranges, with associated greater dispersal capabilities that enable them to colonize diverse habitats, whereas species with shorter wings are often confined to more localized environments (McCulloch et al. 2017). Beyond driving species’ distribution, wing morphology and flying behavior may potentially promote speciation through allopatric divergence (Kennedy et al. 2016). Strong migratory species often have large ranges occurring in seemingly isolated islands but showing limited genetic variation as their long-distance flight capability still leads to an exchange of genetic material. Indeed, *P. flavescens*, despite its global distribution, shows minor genetic variation among populations due to its transoceanic migrations (Hobson et al. 2012; Troast et al. 2016; Ware et al. 2022). Although dragonflies often use active dispersal to colonize oceanic islands, some small damselflies have dispersed over the ocean and have been able to colonize distant archipelagos apparently driven by air currents or transport of macrophytes, such as *Ischnura hastata* and *Ischnura capreolus*, the only damselflies in the Galapagos in the Pacific Ocean and Fernando de Noronha in the North Atlantic (Rafael et al. 2020; Cordero-Rivera et al. 2023, respectively). *Ischnura hastata* is also the dominant damselfly in the Azores, where it reproduces parthenogenetically (Lorenzo-Carballa and Cordero-Rivera 2009), and, as with *P. flavescens*, shows very little genetic differentiation over large geographic scales (Lorenzo-Carballa et al. 2012). Colonization events in more sedentary species are more likely to result in allopatric speciation, with species reaching a new island developing into new species or radiations, such as in the damselfly genera *Nesobasis* and *Nikoulabasis* on Fiji or *Megalagrion* on Hawaii (Ferguson et al. 2023; Donnelly and Marinov 2024; Hadfield et al. 2025). How flight behavior and morphology have evolved across the odonates and their implications for how diversity is structured at the broadest scales remain poorly understood and should be considered in light of both active and passive dispersal. A few limited studies have shown morphological changes related to ability to glide in migratory subgroups, such as among libellulid species (Suárez-Tovar and Sarmiento 2016), but we lack a broader synthesis across the whole of the clade that can better connect wing morphology, flight behavior, and broad-scale biogeographic trends.

Due to their high surrogacy for both terrestrial and aquatic diversity (Darwall et al. 2011) and exceptionally rich natural history records, odonates are among the most frequently used taxa for trait-based analysis of distribution-abundance dynamics (e.g., Mähn et al. 2023; Novella-Fernandez et al. 2023) and ecological responses to climatic changes, such as range shifts and range contractions (Letsch et al. 2016; Engelhardt et al. 2022). Among odonates, flight behavior and wing morphology are considered key adaptations in their ecological strategies and evolutionary success (Corbet 1999; Corbet and May 2008). Flight behaviors within dragonflies are generally grouped into two main categories: “percher” and “flyer” (e.g., Corbet 1999; Corbet and May 2008). Perchers spend the majority of their active time perched on the ground or vegetation, occasionally leaving their perch to snatch prey or engage another odonate; they are also species that regulate their body temperature through movement or through choice of habitat (Corbet and May 2008). Flyers, by contrast, spend the majority of their active time continuously hunting or engaging other odonates while on the wing (Corbet and May 2008). These flying behaviors are linked to wing morphology. Flyers, like species in Aeshnidae, tend to have longer wings, with high-aspect ratios, used for sustained flight and broad dispersal (e.g., Grabow and Rüppell 1995). Perchers, such as most members of Libellulidae, have shorter wings, with lower-aspect ratios (e.g., Grabow and Rüppell 1995), used for quick, agile flight bouts (Wootton 2020). Zygoptera are generally considered “perchers,” but there may be variation in how we define “percher” behavior. Indeed, perching in Anisoptera (e.g., many Libellulidae) and perching in Zygoptera (e.g., Coenagrionidae) are likely different in terms of both the actual behavior and the physiological components related to thermoregulation via perching (Castillo-Pérez et al. 2022). Although perching and flying behavior are typically associated with certain families, the behaviors have evolved multiple times, suggesting an important role in ecological specialization and interactions with key changes in wing morphology (Corbet and May 2008).

Despite the unique potential of Odonata for studying the evolutionary processes underpinning the remarkable diversity of insects, simply recovering the topology and the timing of diversifications over 300 million years of evolutionary history poses major challenges. Advances in phylogenomics highlight the need for and facilitate comprehensive taxon sampling and expanded gene coverage. Such advances can clarify evolutionary signals, improve divergence timing estimates, and test comparative evolutionary questions that can inform evolutionary processes shaping current diversity (Stiller et al. 2024). Therefore, the aim of this paper is fivefold: (1) to provide a well-supported phylogenetic tree for the complete order, (2) to estimate divergence times for the major groups within Odonata, (3) to provide a basic biogeographic framework indicating the relative importance of different biogeographic realms on extant diversity, (4) to test the relationship between wing aspect ratio and flight behavior, and (5) to reconstruct the evolution of flight behavior strategies throughout time. We answered these aims based on extensive gene coverage (~940 loci) and the most comprehensive odonate taxon sampling to date (591 taxa), which includes all 55 currently recognized families and 67% of genera.

## Materials and Methods

### Taxon Sampling

A total of 591 taxa were included in our study (Supplementary Table S1). We incorporated sequence data from 142 taxa from Bybee et al. (2021), one mayfly outgroup from Simon et al. (2018), and four mayfly outgroups from the 1KITE project (Misof et al. 2014; Kawahara et al. 2019). We additionally included 444 samples representing all 55 currently recognized odonate families (*sensu* Goodman et al., 2026). In total, our taxon sampling represents 466 odonate genera, which is approximately 67% of the currently recognized genera and the largest taxon sampling of dragonfly and damselfly phylogenetics to date.

### Target Capture Data Collection and Processing

We extracted genomic DNA from leg tissue and, for some species, flight muscle using either the Qiagen DNeasy Blood & Tissue kit or QuickDNA Miniprep kit (Zymo Research) generally following the manufacturer’s protocol, with modifications in incubation (for 1 week) and heating the elution buffer for 15 min before elution; we also used 100 μL of elution buffer. DNA quantification was conducted using Qubit 4.0 fluorometer (Life Technologies). All DNA extractions were sent to Rapid Genomics (Gainesville, FL) for library prep using the AHE Odonata probe set from Goodman et al. (2023) and sequenced on an Illumina HiSeq 2500 machine.

Sequence data were processed and analyzed on the MENDEL HPC at the American Museum of Natural History and the Office of Research Computing HPC at Brigham Young University. Adapters from raw reads were trimmed and assessed for quality with fastp (Chen 2023) and MultiQC (Ewels et al. 2016). We followed a modified assembly, orthology, and contamination filtering pipeline from Breinholt et al. (2018). In brief, cleaned reads were mapped to probe regions and assembled individually (separate assembly process for each locus) with SPAdes v3.15.5 (Prjibelski et al. 2020). The target probe set (see Goodman et al. 2023) was queried against the assembled scaffolds with the TBLASTX algorithm from BLAST+ v2.15.0 (Camacho et al. 2009) to identify putative orthologs. Orthology was confirmed by ensuring that the scaffold and its associated probe had hits in the same location when queried against the reference *Tanypteryx hageni* genome (Tolman et al. 2023) with TBLASTX. Hits were further confirmed with best reciprocal hit BLAST, by querying the sequence hit in the reference genome against the new locus assemblies. Each resulting locus was aligned using the MAFFT v7.526 (Katoh and Standley 2013) linsi algorithm with the option -adjustdirectionaccurately. Each alignment was trimmed with TrimAl v1.4.1 (Capella-Gutiérrez et al. 2009) with the heuristic automated method and subsequently visualized manually in AliView v1.28 (Larsson 2014). Aligned loci with 75% minimum locus completeness (i.e., only loci with at least 75% of the taxa present were retained) were concatenated into a data matrix for downstream analyses.

### Phylogenetic Analysis

Phylogenies were estimated with a maximum likelihood (ML) inference using IQ-TREE v2.2.0 (Minh et al. 2020). ML inference was conducted three times, and the best tree was chosen as the one with the highest likelihood. We implemented ModelFinder (Kalyaanamoorthy et al. 2017) to select the best-fit partitioning scheme and best nucleotide substitution model for each partition. Support values were inferred from 1000 ultrafast bootstrap replicates (Hoang et al. 2018) with the -bnni option to reduce the potential for severe model violations.

### Fossil Calibrations and Divergence Time Estimation

To calibrate our divergence time estimation analysis, we chose 22 vetted fossil calibrations across the phylogeny representing the crown nodes of (a) Palaeoptera, (b) Ephemeroptera, (c) Odonata, (d) Zygoptera, (e) Epiprocta, (f) Anisoptera, (g) Aeshnidae, (h) Gomphidae, (i) Petaluridae, (j) Cavilabiata, (k) Chlorogomphidae + Cordulegastridae, (l) Libellulidae + Corduliidae, (m) Libellulidae, (n) Corduliidae, (o) Macromiidae, (p) Calopterygidae + Chlorocyphidae, (q) Calopteryginae subgroup hereafter referred to as the Calopterygid Complex (i.e., clade comprised of *Sapho, Umma, Mnais, Vestalis, Echo, Archineura, Mnais, Psolodesmus, Calopteryx, Atrocalopteryx, Matrona, Matronoides*, and *Neurobasis*), (r) Lindeniinae, (s) *Erpetogomphus* + *Nihonogomphus*, (t) *Anax*, (u) Libellulinae, and (v) *Sympetrum* + *Celithemis*. Several molecular dating analyses for Odonata have implemented fossil calibration schemes, with Kohli et al. (2016) outlining a comprehensive list of vetted Anisoptera fossil calibrations. In Kohli et al. (2021), the authors re-evaluated the most appropriate fossil calibrations for all of Odonata and tested the placement of several fossils such as *Proterogomphus*, which has been previously considered to be either stem- or crown-Gomphidae. The impact of this fossil significantly impacted ages throughout the topology depending on the placement. Here, based on Kohli et al. (2021), we chose to place *Proterogomphus* as crown-Gomphidae due to our increased taxon sampling compared with Kohli et al. (2021), specifically due to our inclusion of gomphid species from Lindeniinae. For all fossils, we followed the best practices outlined in Parham et al. (2012), including ages, citations, phylogenetic justifications, accession numbers, etc., for all fossils; these details can be found in Supplementary Table S2 and in Kohli et al. (2021).

Divergence time estimation analysis was conducted with our 75% loci completeness data matrix in MCMCtree, implemented in the PAML package v4.9j (Yang 2007), using our inferred ML tree as the input topology. We used the closest match to the best-fit substitution model for our ML tree (TVM+F+I+I+R10) available in MCMCtree (i.e., HKY85) for calculating the Hessian matrix. We ran four independent MCMC runs over a million generations with a uniform prior distribution for each fossil calibration and used an independent rates clock model. We checked for convergence (where plots showed convergence and Effective Sample Size values were greater than 200) in the program Tracer v 1.7.2 (Rambaut et al. 2018), and adjusted our burnin accordingly. We examined our priors, realized priors, and posteriors using ggplot2 (Wickham 2016) in R (Supplemental Fig. 1).

### Flight Behavior and Wing Morphology Analyses

To reconstruct the evolution of flight behavior strategies across the phylogeny, we ran an ancestral state reconstruction analysis using the R-package *corHMM* (Beaulieu et al. 2013). Flight behavior characters were scored at the genus level as either “flyer” or “percher” (Corbet and May 2008) and are listed in Supplementary Table S3. The definitions for these two behaviors, related to their thermoregulation, are detailed above. When it was uncertain whether a species was a flyer or an intermediate form, we opted to classify them as flyers. Character state optimizations utilized models with transition rates being equal (ER), transition rates between any two states being equal (SYM), and all transition rates differing (ARD). The best-fit model was chosen by selecting the lowest corrected Akaike Information Criterion (AICc) value computed by the ancestral state reconstruction analysis.

Wing measurements for the length, width, and area of forewings and hindwings were done for the lineages in our phylogeny from wing scans, images of pinned specimens or drawn outlines of specimens where both the fore- and hindwing were in the same plane. We measured at least one individual per species, but for a majority of species we measured 5–10 individuals (males and females; see Supplementary Table S4 for details on the number of scans for each genus). We did not measure every species in a genus, but, where possible, measured multiple species in a genus. Briefly, wings were excised from the odonates’ bodies and placed on a flatbed scanner. Wings and the whole bodies of each odonate were scanned with a scale bar and color standard as outlined in the TOWD project (see Sanchez Herrera 2016; Sáenz Oviedo et al. 2022; Idec et al. 2024 for details on the TOWD dataset; https://digitizingdragonflies.org/). In a majority of samples, the left fore- and hindwing of each specimen were excised and scanned using an Epson scanner in color (RGB) at a resolution of 600 dpi; when the left wing was damaged, we scanned the right wing. From these images, we measured forewing and hindwing length and width. Measurements for wing scans and traces were calculated using ODOMATIC and through a custom Python script that used an adaptive threshold to remove wings from the background and then took the length and width of an ellipse fit around each wing, using image scale bars for accurate measurement (https://github.com/jidec/new-odonate-wings-measurement). With these measurements, we calculated the mean length or width value for every genus except *Arabineura, Azuragrion, Mastigogomphus, Tragogomphus*, and *Trithetrum* (Supplementary Table S3). We then calculated a simple aspect ratio measurement for each wing (wing length divided by wing width), which reflects flight behavior; wings with high aspect ratios that are longer and thinner tend to be found in species that fly fast with lower drag and more lift, compared with those that have shorter, wider wings (e.g., Vargas et al. 2008; Fu et al. 2014; Bomphrey et al. 2016; Wootton 2020).

To determine whether there were significantly different aspect ratios in each wing among perchers and flyers within Odonata, a binomial PGLM analysis was run. The aspect ratios were subset to include only the species within the phylogeny. The PGLM (Flight_strategy ~ forewing_aspect_ratio*hindwing_aspect_ratio) was conducted using the *phyloglm* function in the R-package *phylolm* (Ho et al. 2024). A generalized linear model analyzed wing aspect ratio for hindwing and forewings with normal distribution and identity-link function using flight strategy and family as independent variables in JMP Pro 18.

### Biogeographic Analysis

We ran our biogeographic analysis in BioGeoBEARS v1.1.3 (Matzke 2018). We ran the six available models and performed model selection to pick the best-fit model. We used default parameters so as to not constrain dispersal or allowed areas because odonates are old taxa, which diversified when many continents were connected and have a relatively large range of dispersal capacities. We scored each taxon to exist within any of nine defined biogeographic realms (e.g., Olson et al. 2001; Abbott et al. 2022; Kalkman et al. 2022): Nearctic, Neotropics, Palearctic, Afrotropics, Madagascar + Seychelles, Indomalayan, Wallacea, Australasia, and Oceania (Supplementary Table S5). Once the best model was selected (i.e., BAYAREALIKE), the ancestral area reconstruction was performed in BioGEOBEARS (Matzke 2018) and corroborated in RASP (Yu et al. 2020). RASP was also used to estimate the number of speciation events (both recent, and ancestrally) and dispersal events.

Briefly, realms were defined as follows. The Nearctic realm was defined by a distinct assemblage of natural communities in North America delimited in the southern part of the realm by the start of a transition zone that includes three mountain ranges of northern and central Mexico: the Sierra Madre Occidental, Sierra Madre Oriental, and Eje Neovolcánico Transversal (Escalante and Morrone 2021). The Neotropical realm was defined by the aforementioned transition zone at the southern part of the Nearctic realm, extending southward through South America. We followed the definition of Olson et al. (2001) for the Palearctic realm, including the Palearctic parts of China, India, Nepal, and Bhutan. We defined the Afrotropics to include Africa from the sub-Saharan region southward, excluding Madagascar, which we combined with the Seychelles as a separate and unique realm. We followed the definition of Olson et al. (2001) for the Indomalayan realm, including the Chinese provinces of Sichuan, Hubei, Anhui, and Jiangsu southward. Wallacea was defined to include the Philippines and all islands that were never connected to mainlands within the Indomalayan and Australasian regions. We defined Australasia as including Australia, New Guinea, New Zealand, New Caledonia, and the Solomon Islands, but unlike Olson et al. (2001), we excluded Vanuatu, as all other islands contain at least fragments of Gondwana and share a similar fauna, whereas the islands of Vanuatu are all oceanic in nature. Oceania is a region of islands in the South Pacific Ocean, including Hawaii and Vanuatu, unlike Olson et al. (2001).

## Results

### AHE Stats and Phylogenetic Analysis

In our 75% loci completeness data matrix, we recovered a total of 940 AHE loci with 179,376 characters consisting of 104,309 parsimony-informative sites, 15,885 singleton sites, and 59,182 constant sites. We recovered 883 loci on average for each taxon, with a range from 182 to 939 loci as detailed in Supplementary Table S3. Our topology (Fig. 1) largely reflects the taxonomic groupings recovered in recent large-scale multi-gene phylogenies but with a higher taxon sampling and higher support (Bybee et al. 2021; Kohli et al. 2021; Suvorov et al. 2022; Goodman et al., submitted).

**Figure 1.**
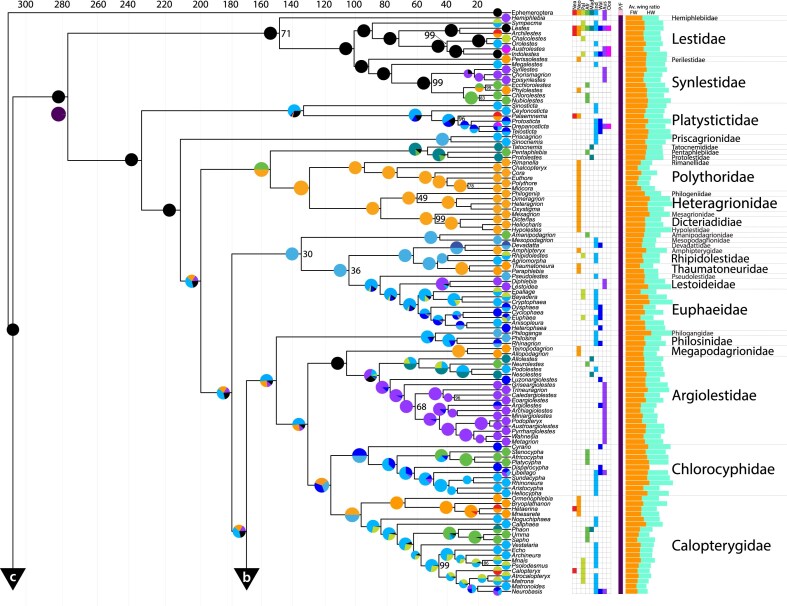
Dated topology of Zygoptera (1a and 1b) and Epiprocta (1c and 1d). Ancestral state reconstructions shown by pie chart: generic biogeography is shown at the node (size of pie charts is only for readability), flight behavior is shown subtending the nodes along the backbone only. The pie charts for biogeography are a summary of the ancestral areas. Current geographic distribution of each genus is shown in the grid immediately to the right of the generic name. Flight behavior (percher vs. flyer) is shown in a ribbon to the right of the current distribution grid. The aspect ratio of the fore- and hindwings is shown as well.

**Figure 1.**
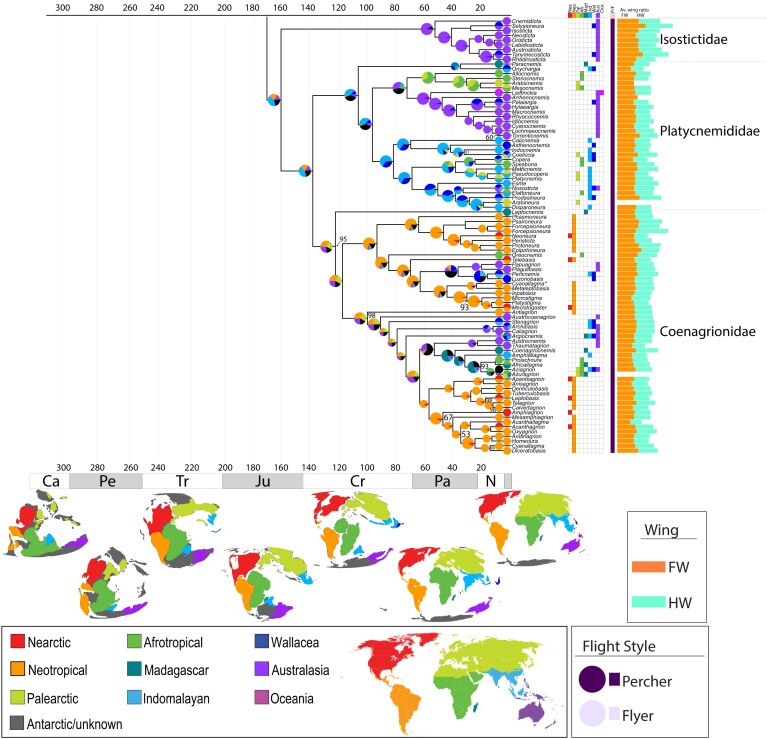
Continued.

**Figure 1.**
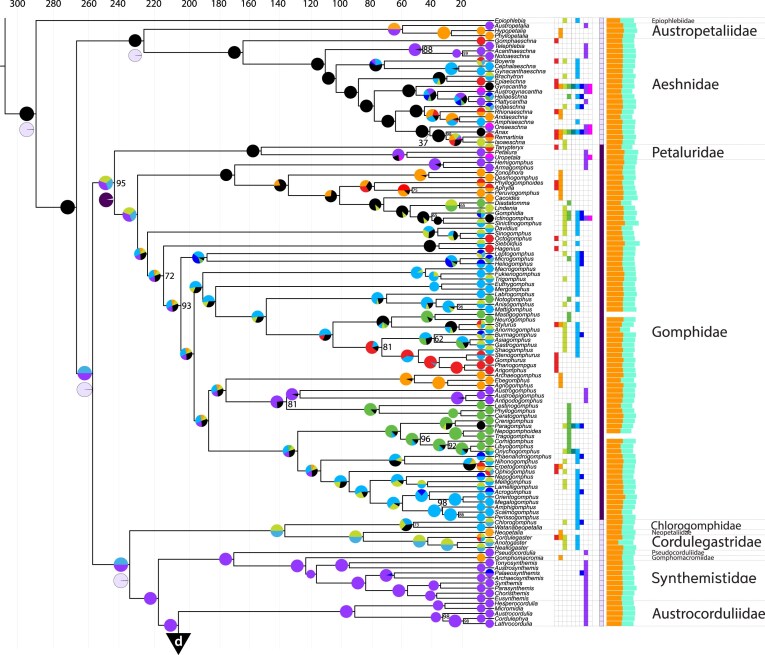
Continued.

**Figure 1.**
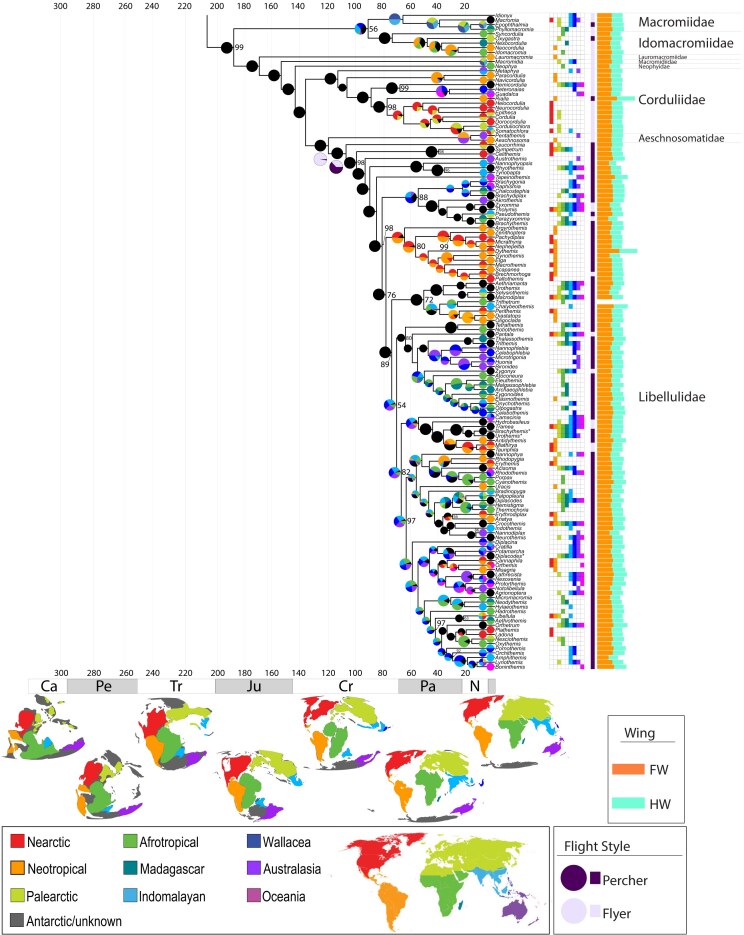
Continued.

Zygoptera, damselflies, were recovered as monophyletic. Within Zygoptera, all superfamilies were found to be monophyletic except for “Calopterygoidea.” A clade comprising the “Calopterygoidea” families Philogangidae, Philosinidae, Megapodagrionidae, Argiolestidae, Chlorocyphidae, and Calopterygidae was recovered as sister group to Coenagrionoidea. Epiprocta was recovered as monophyletic, and all Anisoptera superfamilies were monophyletic. Petaluridae was recovered as a sister lineage to Gomphidae with relatively high support (95% bootstrap) and Petaluridae + Gomphidae as the sister clade to Cavilabiata (100% bootstrap). We recovered *Aeschnosoma* + *Pentathemis* as sister to Libellulidae with full support as found in Goodman et al. (submitted). Libellulidae was recovered as monophyletic, with the backbone of this family possessing short branch lengths and varying from low to high support as in previous studies (e.g., Ware et al. 2007; Pilgrim and von Dohlen 2008).

### Divergence Time Estimates

All divergence time estimates and 95% credible intervals (CI) for deeper level nodes (i.e., ordinal, subordinal, superfamily, and family level nodes) are shown in Supplementary Table S6. Divergence time estimates suggest the divergence of crown-Epiprocta (i.e., the time of most recent common ancestor, TMRCA, of Anisoptera and Anisozygoptera) and crown-Zygoptera occurred in the early Permian (~290 Ma) and mid-Permian (~278 Ma), respectively, during a time when the supercontinent Pangea existed. The TMRCA of the damselfly superfamilies “Calopterygoidea” + Coenagrionoidea occurred in the late Triassic (~213 Ma), during Pangea, with superfamilies Coenagrionoidea and Lestoidea emerging in the late Jurassic (~160 and ~149 Ma, respectively, during a time when Gondwana and Laurasia existed) and Platystictoidea in the early Cretaceous (~134 Ma, during a time when Gondwana and Laurasia existed).

Crown-Anisoptera emerged in Pangea during the mid-Permian (~274 Ma) with subsequent origin of the dragonfly superfamilies Aeshnoidea, Gomphoidea, and Libelluloidea on Pangea in the late Triassic (~230, ~234, and ~220 Ma, respectively). Across Gondwana and Laurasia, Petaluroidea arose in the late Jurassic (~153 Ma) and Cordulegastroidea in the early Cretaceous (~137 Ma).

The superfamily Lestoidea was recovered as sister to all other damselflies, and the TMRCA suggests they diverged ~270 Ma in the mid-Permian of Pangea. The extant Hemiphlebiidae separated from all the other families (Lestidae) (Perilestidae + Synlestidae) during the late Jurassic. Lestidae diverged after Pangea had split into Gondwana and Laurasia, 101 Ma, followed by the separation between Perilestidae and Synlestidae 91 Ma during the mid-Cretaceous when the continents had begun splitting up into their current forms.

Within “Calopterygoidea,” divergence estimates suggest that major lineages originated between the late Triassic and mid-Jurassic, with subsequent diversification occurring primarily in the Cretaceous (see Supplementary Table S6; Fig. 1). Prisciagrionidae is recovered as the earliest-diverging damselfly lineage, whereas other families form distinct clades with TMRCAs spanning the Jurassic.

### Biogeography

**Figure 2.**
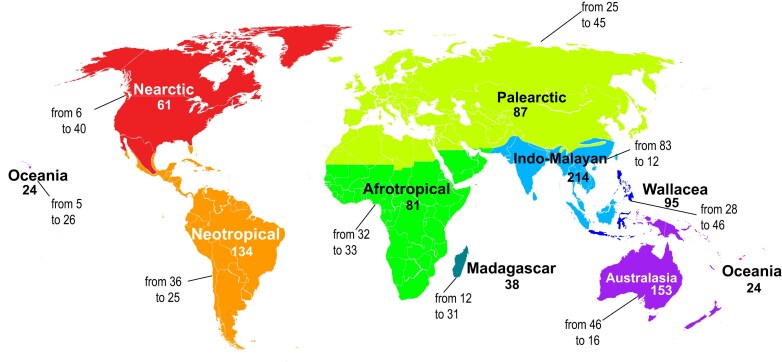
Speciation and dispersal events inferred across evolutionary time using the best-fit biogeographical model, BayArea. The number of speciation events within each biogeographical realm (**bold** below the name), and dispersal events occurring from and to these realms.

Figure 1 summarizes the global biogeographic history of Odonata, integrating a dated phylogeny, the nine ancestral areas considered, and the present-day distribution of the 55 extant families and 466 genera. The Neotropical and Indomalayan realms each host 27 families, making them by far the richest at the family level. Of the 55 families, 26 are endemic to a single realm, with this pattern being more pronounced in Zygoptera (21 of 34) than in Epiprocta (5 of 20). Figure 1 shows that family-level diversification events are by and large evenly distributed over the past 300 Ma. There is however a difference between Zygoptera and Anisoptera; across the tree, speciation events in Zygoptera are relatively more recent, with 15 of the 33 events (45%) taking place in the past 100 million years. Only 3 of the 18 (27%) speciation events were as recent in Anisoptera. The ancestral area reconstruction reveals further distinct differences between diversification in Zygoptera and Epiprocta. The biogeographical model predicts that the Australasian realm—and to a lesser extent, the Palearctic and Indomalayan realms—played a key role in family-level diversification in Epiprocta (Epiophlebiidae + Anisoptera). In contrast, the Indomalayan and Neotropical realms were central to the diversification of Zygoptera. Figure 2 and Table 1 present a summary of the estimated speciation and dispersal events for the 466 genera included in our analyses (please see Supplementary Table S7 for additional details). The results highlight again the prominent role of the Indomalayan, Neotropical, and Australasian realms, which respectively account for 24%, 15%, and 17% of speciation events. This trend is mirrored in the dispersal dynamics, with these same realms—particularly Indomalayan—acting as net donors of diversity. In contrast, other realms either show a balance between incoming and outgoing dispersal (e.g., Afrotropical) or are net receivers of diversity (Fig. 2). The latter category includes mostly temperate realms, such as Palearctic and Nearctic, as well as island systems not connected to major continental landmasses, including Madagascar, Wallacea, and Oceania.

**Table 1.** The number of vicariance and dispersal events for each of the superfamilies and suborders, with the last column indicating the ratio between vicariance and dispersal events

		Dispersal events nodes							
Lineage	Vicariance events nodes	1–4	5	6	7	8	9	Total	Ratio: vicariance/dispersal
Zygoptera	34	68	2	**–**	**–**	**–**	2	72	32/68
*Lestoidea*	5	2	1	–	–	–	2	5	50/50
*Platystictoidea*	1	3	–	–	–	–	–	3	25/75
*“Calopterygoidea”*	18	15	–	–	–	–	–	15	55/45
*Coenagrionoidea*	12	48	1	–	–	–	–	49	20/80
Epiprocta	43	189	3	1	2		2	228	16/84
*Epiophlebioidea*	–	1	–	–	–	–	–	1	
*Aeshnoidea*	5	12	3	1	1	**–**	2	19	21/79
*Petaluroidea*	1	2	–	–	–	–	–	2	33/67
*Gomphoidea*	11	49	**–**	**–**	1	**–**	–	50	18/82
*Cordulegastroidea*	–	5	–	–	–	–	–	5	
*Libelluloidea*	26	120	0		0			151	15/85

Biogeographic event reconstructions provide further insight (Table 1). The ratio between vicariance and dispersal shows strong differences between taxa. Zygoptera shows a higher proportion of vicariance (34 events, 32% of total), with dispersal inferred at 72 nodes (68%); species-level taxon sampling may affect these estimates of dispersal, however. By contrast in Epiprocta, dispersal dominates, partly because the most species-rich family Libellulidae has high dispersal: 228 of the 271 inferred events in Epiprocta (84%) are due to dispersal. Beyond the overall frequency of dispersal (i.e., 72 vs. 228 events in Zygoptera and Epiprocta, respectively), there are also notable differences in the intensity of dispersal events between Zygoptera and Epiprocta (Supplemental Fig. 2a and b). In Zygoptera, only two families—Lestidae and Isostictidae—exhibited high-level dispersal (i.e., five or more dispersal events at a node), accounting for just 4 of the 72 events, 6%; as noted above, taxon sampling likely impacts these estimates, and several families (Platycnemididae, Chlorocyphidae, Calopterygidae, and Coenagrionidae) have species-level sampling that may have underestimated their dispersal patterns. Meanwhile, high-level dispersal was considerably more common in Epiprocta, with 39 of the 228 (17%) falling into this category. This elevated dispersal was particularly pronounced in the families Aeshnidae, Gomphidae, Corduliidae, and Libellulidae.

Extinction events were found to be rare in both suborders (Supplemental Fig. 2a and b); extinction is difficult to estimate, however, and we know that the fossil record reflects extinction events that have occurred across crown Odonata. Within Zygoptera, only a single extinction event was inferred—at the MRCA of “Calopterygoidea” Group 3. Epiprocta showed slightly higher extinction frequencies, with four events: one at the MRCA of Austropetaliidae and Aeshnidae, two within Gomphidae, and one within Libellulidae (associated with *Austrothemis*).

### Character Mapping

Of the 531 extant species, 116 were categorized as flyers and 415 as perchers (Supplementary Table S3). All 210 species of Zygoptera were categorized as perchers, whereas for Epiprocta, 118 are flyers and 202 are perchers. Flyer behavior was reconstructed as the ancestral state for the root of Odonata, with perching style flight independently arising in Zygoptera, Petaluridae + Gomphidae, and Libellulidae (Fig. 1). Within Libellulidae, multiple independent reversals to flyer behavior were inferred. The ancestral state for Libellulidae was uncertain, with both percher and flyer states essentially equivocal in the ancestor.

### Wing Analyses

**Figure 3.**
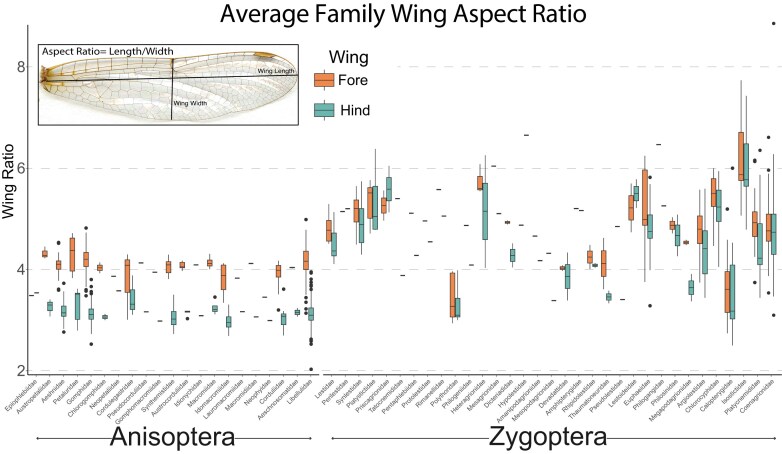
Box and whisker plot of the wing aspect ratios for each family. Note that Epiprocta (Anisoptera + Epiophlebiidae, left-hand section of the graph) has lower aspect ratios overall and more consistency in aspect ratios across the suborder. The highly speciose Anisoptera families Gomphidae and Libellulidae have several outliers compared with other families. Zygoptera has higher aspect ratios overall as well as much more diversity in wing aspect ratios.

For a summary of aspect ratio calculations and aspect ratio diversity across families, please see Supplementary Table S3 and Figure 3. The result of the PGLM for all Odonata indicates that there is no difference in aspect ratios between perchers and flyers (Fw *P* = 1.0, Hw *P* = 1.0, Fw:Hw *P* = 1.0) with an α of 1.246. A generalized linear model showed that flight strategy (percher vs. flyer) was a significant predictor for hindwing aspect ratio in Anisoptera, χ^2^ = 5.73, *df* = 1, *P* = 0.0167. Family was also a significant predictor, *χ*^2^ = 52.38, *df* = 14, *P* < 0.001. Not all families were represented with both flyer and percher flight strategies. When these were removed from the hindwing analysis (292 observations down from 321), the AIC value improved from 11.9881 to 3.2890. Constraining the analysis to families represented by both flight strategies found that both flight strategy and family were still significant predictors of hindwing aspect ratio (flight: *χ*^2^ = 5.68, *df* = 1, *P* = 0.0171; family: *χ*^2^ = 24.19, *df* = 8, *P* = 0.0021) with Argiolestidae, Gomphidae, and Libelluilidae being significant predictors. The model also showed that flight strategy was a significant predictor for Anisoptera forewing aspect ratio, *χ*^2^ = 11.54, *df* = 1, *P* = 0.0007, with an AIC of 104.5173. Family was not a significant predictor for forewings. Constraining the analysis to families represented by both flight strategies showed consistent results with flight strategy as a significant predictor of forewing aspect ratio (*χ*^2^ = 11.47, *df* = 1, *P* = 0.007 and an AIC of 86.5584).

## Discussion

### Phylogeny and Divergence Times

The phylogeny presented here is the most extensive and best supported to date and supports the numerous changes made in the classification based on molecular data in the past decade, including the establishment and revised definition of the families included in Libelluloidea proposed by Goodman et al. (submitted). Our phylogeny shows that Calopterygoidea, as currently defined, is non-monophyletic. For this reason, we refer to it as “Calopterygoidea.” An updated classification including new superfamily definitions for the families of “Calopterygoidea” will be published elsewhere (Carter et al. forthcoming). With the current paper, the 200-year struggle to reach a best-supported classification for odonates is nearing its end, with the remaining issues on family level being restricted to the position of the genus *Sciotropis* (either placed in an existing family or better regarded as a monotypic family) and the definition of Coenagrionidae, to be kept as is or better split into multiple families, as was recently proposed by Pessacq et al. (2025).

Our divergence time estimates at the deeper nodes are generally older than estimates from recent phylogenomic studies (Kohli et al. 2021; Suvorov et al. 2022), with the exception of Palaeoptera, which was younger than prior estimates (i.e., recovering ~314 Ma compared with ~397 Ma in Kohli et al. 2021 and ~348 Ma in Suvorov et al. 2022). For example, our crown-Odonata estimate (~313 Ma) is 50 Ma older (~263 Ma; Kohli et al. 2021) and 87 Ma older (~226 Ma; Suvorov et al. 2022). As in past studies, divergence times for both Zygoptera and Anisoptera are very similar: both our crown-Anisoptera and crown-Zygoptera estimates suggest these lineages originated in the Permian (~274 and ~278 Ma, respectively). These ages are older than in past studies, as estimates from both Kohli et al. (2021) and Suvorov et al. (2022) suggest that Anisoptera arose in the early Jurassic (~201 and ~187 Ma, respectively) and Zygoptera originated in the early Jurassic (~189 Ma; Suvorov et al. 2022) or late Triassic (~206 Ma; Kohli et al. 2021). We attribute the differences between our study and those of two prior transcriptome studies to our much more extensive taxon sampling (586 odonate taxa vs. 103 taxa in Kohli et al. 2021 and 83 taxa in Suvorov et al. 2022). Additionally, because of our comprehensive taxon sampling, we were able to include six additional fossil calibrations (see Supplementary Table S2) in addition to the 16 included from Kohli et al. (2021), which also likely contributed to differences in divergence time estimates. However, fossil-calibrated molecular phylogenetic studies are sensitive to various parameters and are recognized for exhibiting biases that estimate an earlier origin of lineages compared with the empirical oldest fossil evidence. These biases have been examined in the literature (e.g., Bromham et al. 2018; Brown and Smith 2018; Coiro et al. 2019), including studies involving winged insects (Wang et al. 2016).

### Biogeography

Dragonflies and damselflies, though representing a relatively small insect order, have long garnered significant interest from scientists, conservationists, and citizen scientists. This sustained attention has resulted in well-documented distributions for most species and comparatively detailed descriptions of global diversity patterns (Clausnitzer et al. 2012; Endersby 2021; Abbott et al. 2022; Kalkman et al. 2022; Alves-Martins et al. 2024). Over the past decade, this wealth of data has fueled a growing number of studies aimed at uncovering the drivers behind observed biogeographic and diversity patterns. These include investigations into the roles of habitat preference and morphological traits in shaping range size and richness (Hof et al. 2006; Juen and De Marco 2012; Zeuss et al. 2014; Pinkert et al. 2017; Mähn et al. 2023; Novella-Fernandez et al. 2023). In parallel, the availability of detailed distribution data has produced numerous hypotheses on odonate biogeography, many proposed within taxonomic contexts. These were often based on correlations between morphologically inferred relationships and geographic distributions. Only recently have robust methods become available to generate well-supported large-scale phylogenies, offering the tools necessary to test such biogeographic hypotheses rigorously. Examples of these studies on biogeography include publications on the role of continental drift in the phylogeny and distribution of Petaluridae, the diversification on Oceanic islands by *Megalagrion*, and the faunal exchange between Neartic and Palaearctic of the temperate genus *Somatochlora* (Tolman et al. 2024; Hadfield et al. 2025; Goodman et al. submitted).

The analysis presented here is one of the few biogeographical studies in which the evolutionary history of a complete order is analyzed on a global scale with strong molecular support. Comparable studies of this scale remain limited (e.g., in Lepidoptera; Kawahara et al. 2023; Gross et al. 2025). To our knowledge, it is the only comprehensive biogeographical study that spans as far back as 300 Ma, encompassing the breakup of the supercontinents Pangaea, Laurasia, and Gondwana, and intersecting three global mass extinction events. Our results show that most superfamilies in Odonata diverged before the full separation of Pangaea, approximately 200 Ma, into Gondwana and Laurasia. The MRCA of the 55 families of odonates ranges from 17 to 164 Ma, supporting the hypothesis that continental drift and resulting vicariance were major drivers of diversification at the family level. This is reinforced by the reconstructed ancestral areas, which highlight the early role of regions such as Australasia, Indomalaya, and the Neotropics in the origins of both suborders.

The phylogeny presented includes several cases of extensive temporal diversification at the genus and even the family level within the biogeographical realm. For example, the diversification of families Argiolestidae and Isostictidae occurred largely within the Australasian realm, whereas multiple endemic families within the Neotropics (Polythoridae, Philogeniidae, Heteragrionidae, Mesagrionidae, Dicteriadidae, and Hypolestidae) illustrate deep regional diversification. These patterns are consistent with the historical configuration of landmasses and suggest ancient radiation following the breakup of Gondwana. The dated phylogeny further reveals asymmetries between Zygoptera and Epiprocta. In Zygoptera, speciation events skew toward the past 100 Ma, with a biogeographic signature marked by limited dispersal and higher regional endemism. In contrast, Epiprocta exhibits a greater proportion of older lineages and is strongly shaped by frequent and high-intensity dispersal events, particularly in Aeshnidae and Libellulidae.

The spatial dynamics of diversification are also reflected in the estimated dispersal and vicariance events. Zygoptera displays a more vicariant pattern, with a smaller proportion of high-intensity dispersal, as seen in Lestidae, for example, as described above. Epiprocta, however, is dominated by dispersal events, including many high-intensity episodes (17%), reflecting its broader ecological tolerance and greater vagility, but also likely influenced in particular by the characteristics of Libellulidae especially. For example, aeshnids of *Anax*, corduliids of *Hemicordulia*, and libellulids of *Tramea*, and *Pantala* are genera seemingly not bound by biogeographical limits, having a dispersal capacity to extend their range beyond biogeographical boundaries even after land connection ceased to exist.

Inferred extinction events are found to be rare in our dataset, but informative. These events are minimum estimates of course, as we know from the fossil record of a variety of extinct lineages. Within Zygoptera, a single extinction event is inferred in “Calopterygoidea” (~135 Ma), whereas in Epiprocta, four events are inferred, including one at the MRCA of Austropetaliidae and Aeshnidae (~229 Ma) and others within Gomphidae and Libellulidae. These extinctions, although sparse, likely shaped present-day patterns of restricted diversity and may represent relictual distributions. However, the fossil record, though limited, indicates that extinction events were more common than inferred in our analyses. For instance, fossils of *Palaeophya argentina* (South America) and *Neophya legrandi* (Europe) likely belong to Neophyidae, now confined to the Afrotropics (Goodman et al. submitted). Also, a fossil of the genus *Chlorocypha*, now restricted to sub-Saharan Africa, was collected in Europe (Nel et al. 2017). This suggests that many current distributions may be relictual, remnants of ancestral cosmopolitan ranges.

Modern-day regional contributions to diversity further reflect ancient processes. The Indomalayan, Neotropical, and Australasian realms emerge as centers of origin and diversity in both suborders. These realms show high frequencies of inferred speciation and dispersal events, whereas temperate (e.g., Palearctic, Nearctic) and isolated island regions (e.g., Wallacea, Oceania, Madagascar) are net receivers of diversity, often acting as sinks rather than sources.

Our data offer a robust framework to evaluate how present-day diversity patterns are shaped by a combination of deep-time geological processes, dispersal capacity, and modern climate. Previous studies have shown the role of current climatic conditions in species richness, with warmer and wetter areas being more diverse (Abbott et al. 2022; Kalkman et al. 2022; Cortés-Guzmán et al. 2024; Willink et al. 2024). Thus, distribution patterns seen among families are largely shaped by continental drift and dispersal capacity, whereas generic diversity is influenced by species’ dispersal capacities but also determined by recent climatic gradients. The data presented here provide a foundation for exploring how continental drift, dispersal capacity, and present-day environmental conditions interact to shape current patterns of diversity.

The data presented in this study are based on an extensive taxon sampling (all 55 families and 466 genera, ~67% of extant genera), allowing for future studies in which comparisons across lineages and regions to rigorously test proposed biogeographical scenarios. This broad coverage allows us to study congruent patterns, such as the relict Pangaean or Gondwanan distributions seen in Aeschnosomatidae, Argiolestidae, Petaluridae, and Synlestidae, or the complex biogeographic origins of Madagascar's fauna. The analysis also reveals how barriers like Wallacea and Central America have acted as biogeographic filters, influencing the flow of lineages across regions.

Together, integrating biogeographic reconstructions with a dated phylogeny spanning 300 million years provides new insights into the complex and layered evolutionary history of Odonata. The observed patterns highlight the interplay between ancient continental shifts, lineage-specific dispersal capacities, extinction events, and recent climatic influences in shaping global dragonfly and damselfly diversity. The data presented here serve as a critical foundation for future studies aimed at disentangling the roles of deep-time processes, dispersal dynamics, and contemporary ecological conditions in generating present-day biodiversity.

### The Evolution of Flyers/Perchers and Differences in Dispersal Mode and Ability between Flight Strategies

Combining our comprehensive phylogenetic backbone for Odonata with trait information reveals the major role of broad differences in flight behavior. Flying and perching are flight behaviors, but as discussed in Corbet and May (2008), these behavioral categories are also related to thermoregulatory strategies. We can see from our data that wing aspect ratio in Anisozygoptera and Anisoptera (Epiprocta) is highly different between perchers and flyers but more information is needed to understand these behaviors in Zygoptera, especially as it relates to thermoregulation.

Flight behavior category and aspect ratio are two traits that carry a strong phylogenetic signal in our data, but more data are needed to evaluate the variance we see in several clades. As continents drifted, and the distance among them increased, and whereas some odonates appear to have remained short-distance dispersers (i.e., staying local), others have evolved into long-distance dispersers occupying large distributions or, as in *Pantala flavescens* and some *Anax* species, have been selected as migrants (e.g., Corbet 1999; Wikelski et al. 2006; May 2012; Clement et al. 2022; Ware et al. 2022). Of the flight styles we used to categorize Odonata, flyer seems to have been the ancestral state, which diverged in the Carboniferous, a time when Pangaea was still intact. Flyer-style odonates have been suggested to have higher flight speeds in general and include taxa with longer-distance flight capabilities, but the speed at which perchers are maneuvering may also be high (Wootton 2020). Note that some tiny Zygoptera (e.g., *Ischnura*) have been able to consistently colonize oceanic islands, even over 1500 km from the nearest continent, likely because they use passive dispersal instead of active migration (Cordero-Rivera forthcoming).

Although higher aspect ratios are associated with reduced drag, more efficiency in terms of lift, and are associated with faster flyer style flight, lower aspect ratios are associated with maneuverability and gliding flight (Wootton 1991, 2020; Wakeling 1997). However, within Epiprocta wing aspect ratios were significantly different between perchers and flyers. Zygoptera were scored as perchers across all species, but we can visually note that there is high variance in the aspect ratios across the Zygoptera portion of the “percher” wings, when compared with the Anisoptera wings, suggesting that perhaps Zygoptera-style “perching” should be classified as multiple flight styles. Indeed, the aspect ratios in Anisoptera of perchers may not be comparable to the aspect ratios of perchers in Zygoptera (e.g., Wootton 2020), so it is hard to make inferences across the suborders. Within Epiprocta, shifts to flyer-style flight in the Triassic corresponded with morphological shifts in wing length versus width. Anisoptera perching seems to have arisen in the ancestor to Gomphidae + Petaluridae, and again in the Libellulidae. We reconstruct the ancestral state for Anisoptera as flyer, suggesting there was an independent shift to perching in the Gomphidae + Petaluridae ancestor during the Triassic, and then again in the Cretaceous and subsequent years in Libellulidae. Because the ancestral state for Libellulidae is equivocal, we can conclude that there are multiple origins of flyers or perchers in the family. Epiproctan flyers differ significantly in their aspect ratios between perchers and flyers when considering their forewings and their hindwings separately, which has been suggested in past studies (e.g., Wootton 2020). It is likely that forewing and hindwing differences independent of flight style would be dramatic between Zygoptera and Epiprocta/Anisoptera, but we did not test that here. Several Libellulidae genera in our topology (the ancestor of *Miathyria* + *Tauriphila, Pantala, Tholymis*, and *Tramea*) gained a flyer flight strategy—several of these genera are putative migrants, and this suggests that migratory behavior in Libellulidae and associated statistically significantly different flyer-type aspect ratios evolved after the rise of modern birds during a time when the continents were already broken apart into seven main landmasses.

Variation in wing aspect ratios among Zygoptera, as likely in Epiprocta, reflects a wide range of selective pressures, including natural and sexual selection. Several Zygoptera families, including Coenagrionidae and Lestidae, also show signatures of high dispersal capacity in our biogeographic analyses (Table 1; Supplemental Fig. 2a and b), potentially facilitated by wing morphologies that enable more sustained or directed flight. This functional diversity in wing shape may have contributed to their wider geographic ranges and colonization success, but we did not analyze such variation here. Future studies should evaluate this possibility in the context of phylogenetic patterns. Relatively high aspect ratios with respect to the mean for Zygoptera (i.e., having relatively narrow wings) are typical of coenagrionids of Protoneurinae, which are adapted for hovering or fluttering flight in shaded stream habitats. In *Protoneura amatoria*, for instance, males employ conditional mating tactics, including hovering displays, that are influenced by environmental light and female density—behaviors that link wing morphology with fine-scale habitat use and predation risk (Larison 2007). Similarly, Pseudostigmatinae adapted to hunt web spiders need to carefully hover near the web while catching their prey. Góral (2024) suggests that despite traditional mark–recapture studies proposing limited dispersal in Zygoptera, such as in Coenagrionidae and Lestidae, recent indirect evidence—including range expansions, phylogeography and biogeography, similarly seen in our findings—supports higher-than-expected dispersal capacity in several Zygoptera groups, a fact supported by an analysis of island colonization worldwide (Cordero-Rivera forthcoming).

Sexual selection adds further complexity in terms of our understanding of flight behavior, as wings are used in some taxa for displays in addition to being functional for flight. Although this is true in several Odonata, it is particularly striking in several Zygoptera families such as Calopterygidae. Species like *Calopteryx splendens* have low aspect ratios due to having broad wing shapes; their wings are highly pigmented and used in courtship displays. These wing color and shape traits, likely influenced by sexual selection, are advantageous in visual signaling (Outomuro and Johansson 2011) but inherently affect wing aerodynamics. In *Calopteryx haemorrhoidalis*, each pair of wings in males works differently during courtship: although the hindwings are shown to females, the forewings flutter and sustain flight (Córdoba-Aguilar 2000). Additionally, relatively low aspect ratios in the Zygoptera family Polythoridae (e.g., *Euthore, Polythore*) are linked to Batesian mimicry, where species mimic Ithomiini butterflies in color, wing shape, and flight style. These damselflies exploit mimicry rings through convergent visual and locomotor traits that deceive visually sensitive avian predators (Outomuro et al. 2016). *Chalcopteryx* (Guillermo-Ferreira et al. 2014) and *Pseudolestes* (Cordero-Rivera and Zhang 2018) have independently evolved short, wide, and colorful hindwings, intensively used in intra-sexual agonistic displays, and in the last case these colored hindwings are not used for flying in these displays. These examples illustrate how non-aerodynamic selective pressures—such as predator deception or sexual selection—can have an effect, which might be additive, on the evolution of wing morphology in Zygoptera. Indeed, we find that Zygoptera exhibit high morphological diversity in wing aspect ratio (although not statistically tested here, the variance seen in aspect ratios in Zygoptera is notable) and further tests are needed to determine whether this is due to an ecological, behavioral, evolutionary, or a combination of such forces.

### Limitations and Extensions

A caveat to running biogeographical analyses on taxa as old as Odonata is that there has likely been the extinction of taxa whose distribution may influence biogeographic interpretation (e.g., *Neophya-*like fossils from South America are recovered as sister to taxa presently restricted to the Afrotropics; Barden and Ware 2017) and for older taxa that diverged during Pangea, interpreting ancestral ranges is challenging and not very meaningful. Further, tracking the diversification of ancient groups into biogeographic realms that vary significantly over time due to sea level and/or geologic change (e.g., continental shelves, lowlands, and islands, especially volcanic ones) can be complex and difficult. As we continue to gain insight into fossil odonates, future work should assess ways to incorporate extinct lineages into odonate biogeographical studies to evaluate their impact on ancestral states.

A difficulty in evaluating the statistical differences between perchers and flyers is that we lack sufficient information for Zygoptera regarding their thermoregulation and the category of “percher” is likely too crude. Thus, future studies should subdivide the flight styles of Zygoptera using thermoregulation data and quantitative data about flight in the suborder. A limitation to aspect ratio data in general is that there are several methods used to calculate this parameter, making it difficult to compare among studies. Although some studies use ratios of length to width, as we have done here, others use twice the length divided by the width, or the total forewing or hindwing span divided by the area of the pair of fore- or hindwings. Although these variations in calculations make it hard to compare raw numbers, the general patterns of high or low aspect ratios appear consistent across studies.

## Conclusions

Flyers are the ancestral flight behavior in Odonata and the percher behavior arose independently in Zygoptera and two groups of Anisoptera (i.e., Petaluridae + Gomphidae and Libellulidae); several transitions back to flyer-style behavior have occurred in certain lineages of Libellulidae. Aspect ratios highly differ between perchers and flyers from the Epiprocta. We found that particular wing shapes evolved multiple times across Anisoptera likely due to selection for flight styles and dispersal behaviors. Future work should evaluate in more detail variation that may exist in flight behaviors across Odonata, especially among the kinds of perching and particularly that exhibited among the genera of Zygoptera. This future work should also include other functions that underlie wing morphometry (e.g., sexual selection and predation evasion). We provide a significant evolutionary scaffold for such research. Herein, we demonstrate that major relationships within the Odonata are becoming resolved with high support for monophyletic suborders and families. Further work testing the relationship of flight behavior, wing morphology, phylogeny, distribution, and other traits as drivers of odonate diversity are now possible, unlocking a more complete understanding of evolutionary history of Odonata, which has remained elusive for many invertebrate groups.

## Supplementary Material

syag005_Supplemental_Files

## Data Availability

All sequence data can be found in the Sequence Read Archive with the BioProject ID PRJNA1265785. All tree files, data matrices, etc. can be found at https://doi.org/10.5061/dryad.f7m0cfz7p.
