# Supplementary material for: Soaring Systematics: An Evaluation of Biogeography and Flight Behavior in Dragonflies and Damselflies (Insecta: Odonata) Using Phylogenomics
Source: Syst Biol. 2026 Jan 22;75(5):984–1002. doi: 10.1093/sysbio/syag005 (PMC13445619; doi:10.1093/sysbio/syag005)
Supplement: Supplementary file 1 [file syag005_supplemental_files.zip › Supplemental Figure 1 - Posterior and Prior Visualization.pdf]

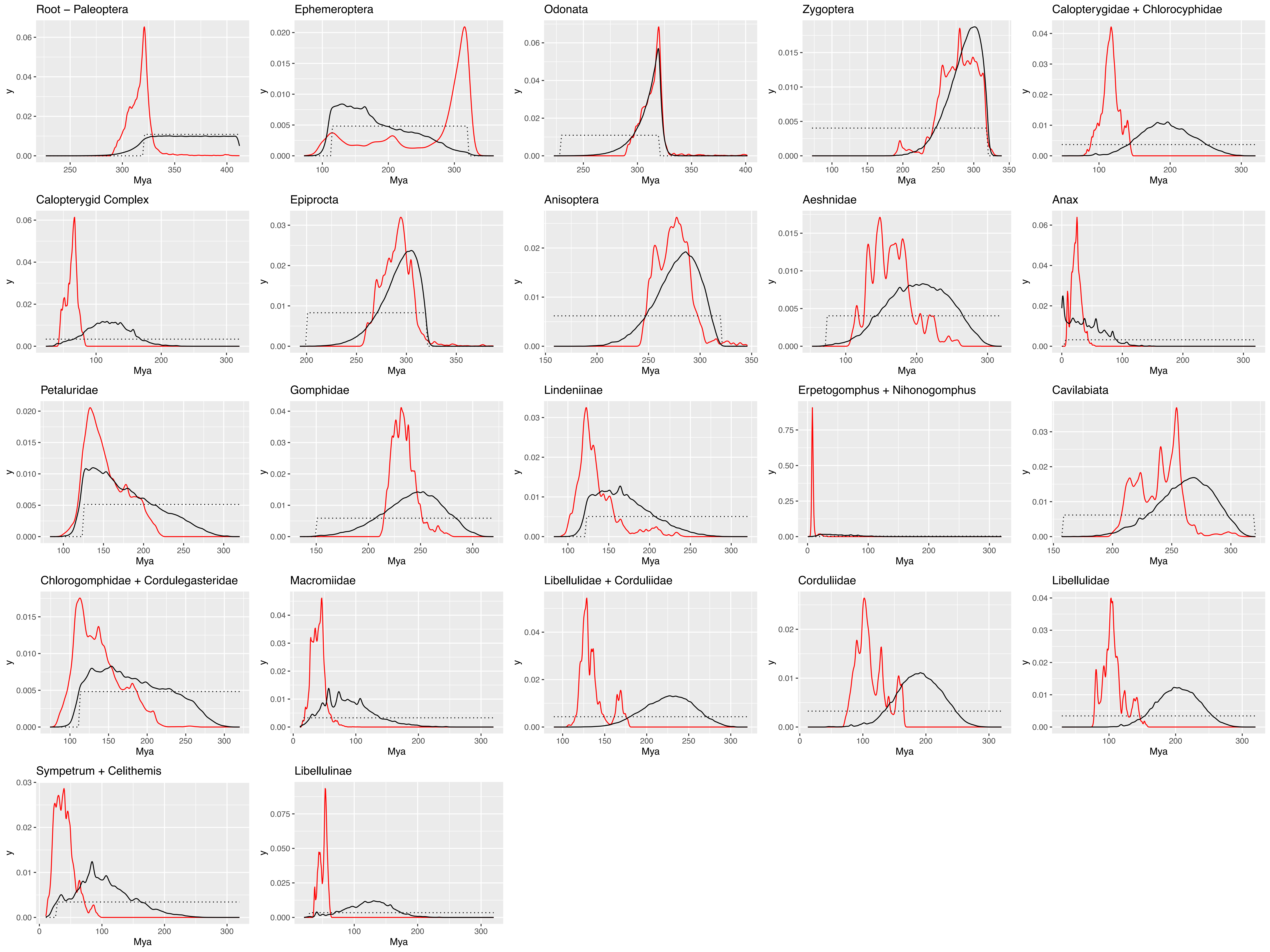
Root – Paleoptera
Ephemeroptera
Odonata
Zygoptera
Calopterygidae + Chlorocyphidae
Calopterygid Complex
Epiprocta
Anisoptera
Aeshnidae
Anax
Petaluridae
Gomphidae
Lindeniinae
Erpetogomphus + Nihonogomphus
Cavilabiata
Chlorogomphidae + Cordulegasteridae
Macromiidae
Libellulidae + Corduliidae
Corduliidae
Libellulidae
Sympetrum + Celithemis
Libellulinae
y
Mya
