# Supplementary material for: Soaring Systematics: An Evaluation of Biogeography and Flight Behavior in Dragonflies and Damselflies (Insecta: Odonata) Using Phylogenomics
Source: Syst Biol. 2026 Jan 22;75(5):984–1002. doi: 10.1093/sysbio/syag005 (PMC13445619; doi:10.1093/sysbio/syag005)
Supplement: Supplementary file 1 [file syag005_supplemental_files.zip › Supplemental Figure 2a - Dispersal-Vicariance-Extinction_Zygoptera.pdf]

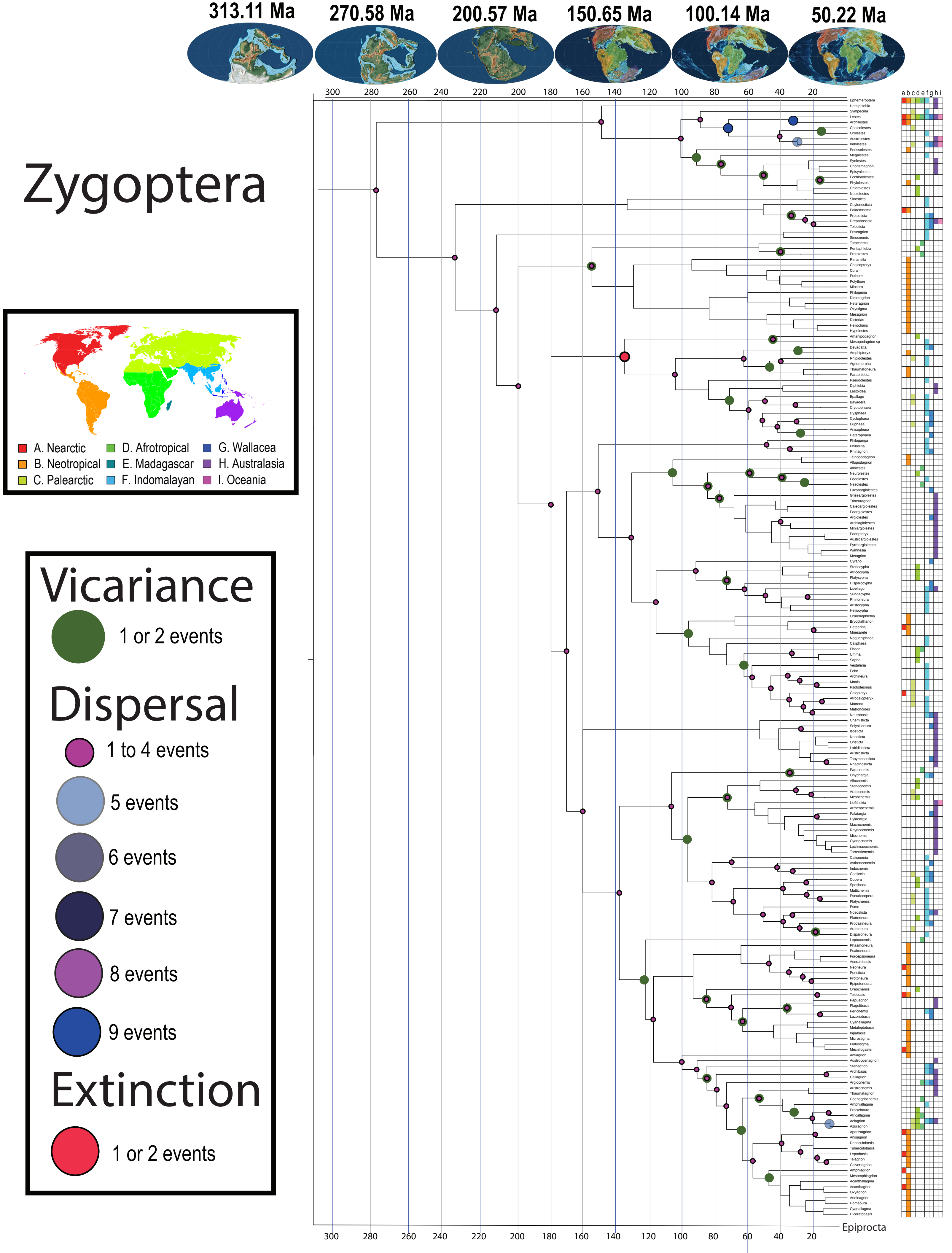

313.11 Ma
270.58 Ma
200.57 Ma
150.65 Ma
100.14 Ma
50.22 Ma
Zygoptera
A. Nearctic
B. Neotropical
C. Palearctic
D. Afrotropical
E. Madagascar
F. Indomalayan
G. Wallacea
H. Australasia
I. Oceania
Vicariance
1 or 2 events
Dispersal
1 to 4 events
5 events
6 events
7 events
8 events
9 events
Extinction
1 or 2 events
300 280 260 240 220 200 180 160 140 120 100 80 60 40 20
a b c d e f g h i
Epiprocta
