# Supplementary material for: Soaring Systematics: An Evaluation of Biogeography and Flight Behavior in Dragonflies and Damselflies (Insecta: Odonata) Using Phylogenomics
Source: Syst Biol. 2026 Jan 22;75(5):984–1002. doi: 10.1093/sysbio/syag005 (PMC13445619; doi:10.1093/sysbio/syag005)
Supplement: Supplementary file 1 [file syag005_supplemental_files.zip › Supplemental Figure 2b - Dispersal-Vicariance-Extinction_Epiprocta.pdf]

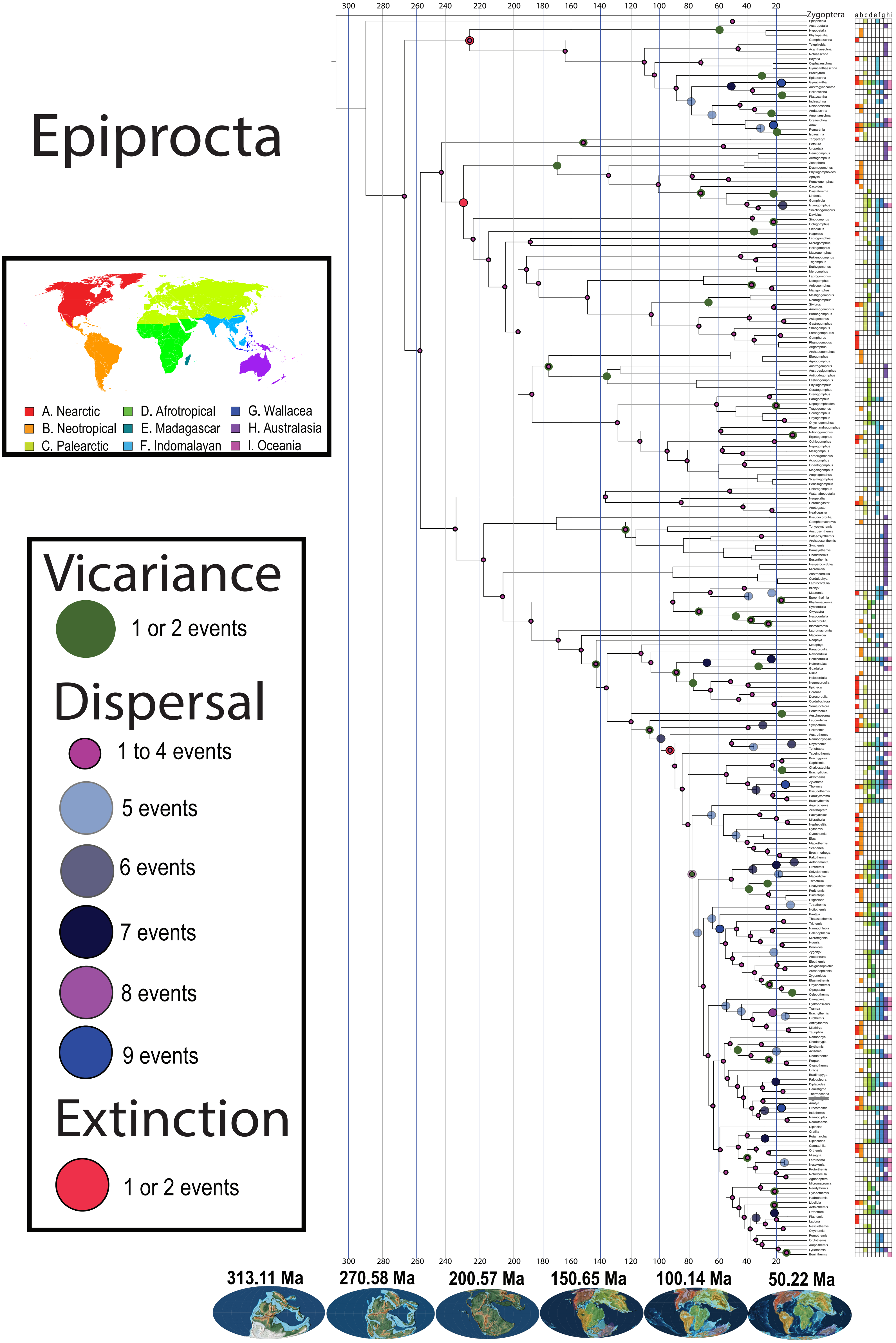
Epiprocta
Zygoptera
a b c d e f g h i
300 280 260 240 220 200 180 160 140 120 100 80 60 40 20
A. Nearctic
B. Neotropical
C. Palearctic
D. Afrotropical
E. Madagascar
F. Indomalayan
G. Wallacea
H. Australasia
I. Oceania
Vicariance
1 or 2 events
Dispersal
1 to 4 events
5 events
6 events
7 events
8 events
9 events
Extinction
1 or 2 events
313.11 Ma
270.58 Ma
200.57 Ma
150.65 Ma
100.14 Ma
50.22 Ma
