# Supplementary material for: Soaring Systematics: An Evaluation of Biogeography and Flight Behavior in Dragonflies and Damselflies (Insecta: Odonata) Using Phylogenomics
Source: Syst Biol. 2026 Jan 22;75(5):984–1002. doi: 10.1093/sysbio/syag005 (PMC13445619; doi:10.1093/sysbio/syag005)
Supplement: Supplementary file 1 [file syag005_supplemental_files.zip › Supplemental Table 1 - Locality Information.docx]

| **Sample** | **Date (yyyy-mm-dd)** | **Collector** | **Country** | **Locality (if Coordinates, separate with a ";")** | **Other details (notes, other)** |
| --- | --- | --- | --- | --- | --- |
| RMNH.INS.501973_Argiolestidae_Argiolestes_muller | 2011 |  | Papua New Guinea |  |  |
| RMNH.INS.503407_Argiolestidae_Caledopteryx_sarasini | 2010-10-12 |  | New Caledonia | Kompwara R. Below, Refuge Blaffart; -20.624, 164.782 |  |
| RMNH.INS.507519_Gomphidae_Ophiogomphus_cecilia | 2009-08-04 |  | Netherlands | Paarlo; 351, 200 |  |
| RMNH.INS.508536_Cordulegastridae_Anotogaster_sieboldii | 2010-09-28 |  | China | Guangdong, Shaoguan City, Nanling National reserve; |  |
| RMNH.INS.508545_Gomphidae_Trigomphus_beatus | 2011-04-15 |  | China | Guangdong, Conghua City, Weidong; |  |
| RMNH.INS.508624_Gomphidae_Fukienogomphus_promineus | 2010-06-05 |  | China | Guangdong, Huizhou City, Nankunshan; |  |
| GEODE17901_Synthemistidae_Synthemis_tasmanica | 1954-01-24 | Angel, S. | Australia | Tasmania Lake Dobson 3400ft |  |
| TM155_Heteragrionidae_Heteragrion_tatama | 2016-06-29 | Ayala, D., A. Orejuela & C. Bota | Colombia | Parque Nacional Natural Tatamá, Vereda Monte Bello |  |
| GEODE7876_Platystictidae_Ceylonosticta_lankanensis | 2009-05-21 | Bedjanic, M. | Sri Lanka | Sri Lanka, Kegalla district, small stream in secondary forest 1 km S of bridge near Plantation Hotel in Kithulgala, loc. 52, 430 m asl., 6°59.063'N 80°24.821'E, 21 May 2009, leg. M. Bedjanic; 6°59.063'N 80°24.821'E, |  |
| GEODE7681_Gomphidae_Phyllogomphoides_undulatus | 1961-10-28 | Belle, J. | Suriname | Para River (upper part) | RMNH.ins.516366 |
| GEODE7682_Aeschnosomatidae_Aeschnosoma_forcipula | 1959-01-31 | Belle, J. | Suriname | Suriname, Zanderij, Pontjikreek | RMNH.ins.516376 |
| GEODE7692_Gomphidae_Phyllogomphoides_fuliginosus | 1963-09-03 | Belle, J. | Suriname | Kabalebo River | RMNH.ins.516246 |
| GEODE2652_Platycnemididae_Asthenocnemis_stephanodera | 1992-05-22 | Borromeo, T. | Philippines | Palawan Ad, Quezon, Magmuni steam, 1000ft |  |
| TM170_Aeshnidae_Remartinia_luteipennis | 2014-04-16 | Bota, C. | Colombia | Parque Nacional Natural Tatamá, Vereda Monte Bello; 5.22855; -76.09826667 |  |
| TM201_Philogeniidae_Philogenia_martae | 2016-08-17 | Bota, C. | Colombia | Parque Nacional Natural Tatamá, Vereda Monte Bello; 5.22855; -76.09826668 |  |
| TM332_Libellulidae_Cannaphila_vibex | 2016-08-26 | Bota, C. | Colombia | Parque Nacional Natural Tatamá, Vereda Monte Bello; 5.2149; -76.0857 |  |
| TM342_Libellulidae_Macrothemis_pseudimitans | 2016-09-17 | Bota, C. | Colombia | Risaralda, Pueblo Rico, Corregimiento Santa Cecilia; 5.3283; -76.1487 |  |
| TM392_Aeshnidae_Rhionaeschna_joannisi | 2016-07-28 | Bota, C. | Colombia | Parque Nacional Natural Tatamá, Vereda Monte Bello; 5.22855; -76.09826673 |  |
| TM404_Heteragrionidae_Heteragrion_erythrogastrum |  | Bota, C. | Colombia | Parque Nacional Natural Tatamá, Vereda Monte Bello; Bochoroma, 5.22855; -76.09826674 |  |
| TM409_Libellulidae_Rhodopygia_hinei |  | Bota, C. | Colombia | Risaralda, Pueblo Rico, Corregimiento Santa Cecilia; 5.3283; -76.1487 |  |
| TM41_Idomacromiidae_Neocordulia_batesi |  | Bota, C. | Colombia | Parque Nacional Natural Tatamá, Vereda Monte Bello; 5.22855; -76.09826676 |  |
| TM532_Libellulidae_Erythemis_plebeja |  | Bota, C. | Colombia | Parque Nacional Natural Tatamá, Vereda Monte Bello; 5.22855; -76.09826677 |  |
| TM581_Aeshnidae_Andaeschna_occidentalis |  | Bota, C. | Colombia | Parque Nacional Natural Tatamá, Vereda Monte Bello; 5.22855; -76.09826678 |  |
| TMCH16_Coenagrionidae_Protoneura_amatoria |  | Bota, C. | Colombia | Risaralda, Rio Sucio |  |
| UAE1_Polythoridae_Euthore_fassli |  | Bota, C. | Colombia | Risaralda, Parque Nacional Natural Tatamá, Vereda Monte Bello, Quebrada Los Chorros; 5,25496° N; 76,11093° W |  |
| RMNH.INS.508201_Platycnemididae_Arabineura_khalidi | 2015-03-30 | Boudot, J.-P. | Oman | Hadjar, Hawqayn; 23.52797, 57.32791 |  |
| GEODE17698_Corduliidae_Dorocordulia_lepida | 2011-07-17 | Bridgehouse, D.W. | Canada | Halifax Drysdale Bog, Big Indian & Pipeline Rd. |  |
| GEODE17699_Corduliidae_Dorocordulia_libera | 2012-06-02 | Bridgehouse, D.W. | Canada | Nova Scotia Cumberland 8.5 NNE of Amherst |  |
| GEODE17703_Corduliidae_Epitheca_cynosura | 2012-06-30 | Bridgehouse, D.W. | Canada | Nova Scotia Halifax Pockwock Watershed |  |
| GEODE17912_Gomphidae_Hagenius_brevistylus | 2010-07-05 | Bridgehouse, D.W. | Canada | Nova Scotia, Hants County. Mt Uniacke, DND bog/fen |  |
| RMNH.5009494_Euphaeidae_Epallage_fatime | 2015-05-08 | Brochard, C. | Bulgaria | Strandzha NP, Varovnik; 42.22322, 27.19295 |  |
| RMNH.5009497_Corduliidae_Corduliochlora_borisi | 2015-05-08 | Brochard, C. | Bulgaria | Strandzha NP, Varovnik; 42.22322, 27.19295 |  |
| RMNH.INS.507528_Aeshnidae_Boyeria_cretensis | 2012-06-19 | Brochard, C. & E. van der Ploeg | Greece | Crete, Filaki, river 200 m NE of,; |  |
| RMNH.INS.507529_Libellulidae_Trithemis_annulata | 2012-06-19 | Brochard, C. & E. van der Ploeg | Greece | Crete, Greece, Crete, Kaloniktis, river 2 km NW of; |  |
| RMNH.INS.507551_Libellulidae_Selysiothemis_nigra | 2012-05-09 | Brochard, C. & E. van der Ploeg | Greece | Corfu, Greece, Corfu, Kavos, 1.3 km SW of,; |  |
| GEODE7723_Synlestidae_Chlorolestes_elegans | 2013-03-12 | Bybee, S.M. | South Africa | Kruger National Park |  |
| GEODE7753_Coenagrionidae_Telebasis_salva | 2016-05-28 | Bybee, S.M. | United States | Placerville; Gold Trail Park; Kathy Lane; 38.752413 -120.844354 |  |
| GEODE7813_Lestidae_Lestes_plagiatus | 2013-03-12 | Bybee, S.M. | South Africa | Kruger National Park |  |
| OD1315_Chlorogomphidae_Chlorogomphus_auratus | 2016-06-21 | Bybee, S.M. | Vietnam | NE Region Ninh Binh Provience Cuc Phuong National Park 20°16.745N 105°40.757E |  |
| OD1322_Corduliidae_Rialla_villosa | 2015-11-01 | Bybee, S.M. | Argentina |  |  |
| OD830_Aeshnidae_Anax_walsinghami | 2015 | Bybee, S.M. | United States | Utah |  |
| GEODE7733_Gomphidae_Tragogomphus_ellioti | 2019-02-02 | Bybee, S.M. & G. Powell | Gabon | Ivindo Nat. Pk. ; 0.5116N, 12.8032 |  |
| GEODE7807_Libellulidae_Tramea_transmarina | 2018-05-14 | Bybee, S.M. & G.S. Powell | Vanuatu | Unpongkor; -18.771033, 169.013417 |  |
| OD1112_Austropetaliidae_Phyllopetalia_apollo | 2015-11-19 | Bybee, S.M. & Pessacq P. | Argentina | Puelo lake, Puerto Patriada, Chubut Province; 42°08'17'' S 71°31'56'' W |  |
| OD1113_Neopetaliidae_Neopetalia_punctata | 2015-11-19 | Bybee, S.M. & Pessacq P. | Argentina | Pil Pil stream, Neuquén Province; 40°11'05'' S 71°23'27'' W |  |
| OD1115_Austropetaliidae_Hypopetalia_pestilens | 2015-11-19 | Bybee, S.M. & Pessacq P. | Argentina | Puelo lake, Puerto Patriada, Chubut Province; 42°08'17'' S 71°31'56'' W |  |
| GEODE7709_Libellulidae_Rhyothemis_notata | 2018-02-21 | Bybee, S.M. & Y. M. Pacheco | Gabon | Stream at bottom of ravine; S02.36927, E11.16140 |  |
| GEODE7805_Libellulidae_Acisoma_trifidum | 2018-02-17 | Bybee, S.M. & Y. M. Pacheco | Gabon | Coastal forest S. of Gamba; 2.8355, 10.096683 |  |
| GEODE7714_Libellulidae_Brachythemis_leucosticta | 2013-05-12 | Bybee, S.M., G. Svenson, N. Hardy | Rwanda | Akagera National Park, Swamp/wetland by entrance gate; 1.928251S 30.674073E |  |
| GEODE7715_Macromiidae_Phyllomacromia_picta | 2013-05-07 | Bybee, S.M., G. Svenson, N. Hardy | Rwanda | N. U. of Rwanda University Arboretum; S2.612869, E29.75995 |  |
| GEODE7722_Platystictidae_Protosticta_satoi | 2016-06-21 | Bybee, S.M., M. F. Whiting, T. H. Ogden | Vietnam | Cuc Phuong National Park; 20.20995N 103.35608E |  |
| GEODE7731_Gomphidae_Ictinogomphus_pertinax | 2016-06-21 | Bybee, S.M., M. F. Whiting, T. H. Ogden | Vietnam | Cuc Phuong National Park; 20 20.995N 105 35.608E |  |
| GEODE7734_Gomphidae_Heliogomphus_scorpio | 2016-06-10 | Bybee, S.M., M. F. Whiting, T. H. Ogden | Vietnam | Tav Yen Tu Nature Reserve; 21 11.029N 106 43.720E |  |
| GEODE7705_Calopterygidae_Noguchiphaea_yoshikoae | 2013-09-12 | Bybee, S.M., N. P. Lord | Vietnam | Tam Dao National Park; N21.45255 S105.64282 |  |
| GEODE7698_Libellulidae_Erythemis_credula | 2017-06-09 | Bybee, S.M., R. A. Clement, S. Smith & R. Ferreira | Brazil | Manaus; S3.230439, W60.64309 |  |
| GEODE7699_Libellulidae_Erythrodiplax_umbrata | 2017-06-15 | Bybee, S.M., R. A. Clement, S. Smith & R. Ferreira | Brazil | Agropalma; S2.543239, W48.798426 |  |
| GEODE7700_Libellulidae_Oligoclada_sp | 2017-06-15 | Bybee, S.M., R. A. Clement, S. Smith & R. Ferreira | Brazil | Agropalma; S2.543239, W48.798426 |  |
| GEODE7702_Libellulidae_Uracis_fastigiata | 2017-06-10 | Bybee, S.M., R. A. Clement, S. Smith & R. Ferreira | Brazil | Manaus; S3.230439, W60.64309 |  |
| GEODE7707_Libellulidae_Elga_sp | 2017-06-15 | Bybee, S.M., R. A. Clement, S. Smith & R. Ferreira | Brazil | Agropalma; S2.543239, W48.798426 |  |
| GEODE7710_Gomphidae_Ebegomphus_sp | 2017-06-15 | Bybee, S.M., R. A. Clement, S. Smith & R. Ferreira | Brazil | Agropalma; S2.543239, W48.798426 |  |
| GEODE7712_Libellulidae_Perithemis_sp | 2017-06-14 | Bybee, S.M., R. A. Clement, S. Smith & R. Ferreira | Brazil | Agropalma; S2.521002, W48.855789 |  |
| GEODE7741_Coenagrionidae_Psaironeura_tenuissima | 2017-06-09 | Bybee, S.M., R. A. Clement, S. Smith & R. Ferreira | Brazil | Manaus; 2.833831S 60.931534W |  |
| GEODE7742_Heteragrionidae_Oxystigma_petiolatum | 2017-06-14 | Bybee, S.M., R. A. Clement, S. Smith & R. Ferreira | Brazil | Agropalma; 2.521002S 48.855789W |  |
| GEODE7748_Calopterygidae_Mnesarete_williamsoni | 2017-06-15 | Bybee, S.M., R. A. Clement, S. Smith & R. Ferreira | Brazil | Agropalma; 2.543239S 48.798426W |  |
| GEODE7704_Rimanellidae_Rimanella_arcana | 2017-06-11 | Bybee, S.M., R. A. Clement, S. Smith, J. F. Lontchi | Brazil | Manaus; 1.992748S 59.557015W |  |
| GEODE7718_Dicteriadidae_Dicterias_atrosanguinea | 2017-06-10 | Bybee, S.M., R. A. Clement, S. Smith, J. F. Lontchi | Brazil | Manaus; S2.846025, W60.916522 |  |
| GEODE7725_Coenagrionidae_Mecistogaster_linearis | 2017-06-15 | Bybee, S.M., R. A. Clement, S. Smith, J. F. Lontchi | Brazil | Agropalma; 2.605046S 48.738174E |  |
| GEODE7750_Platycnemididae_Copera_nyansana | 2017-07-28 | Bybee, S.M., R. A. Clement, S. Smith, J. F. Lontchi | Cameroon | Bouamir Swampy Trail; 3.18965N 12.82299E |  |
| GEODE7758_Coenagrionidae_Acanthagrion_adustum | 2017-06-09 | Bybee, S.M., R. A. Clement, S. Smith, J. F. Lontchi | Brazil | Manaus; 3.230439S 60.64309W |  |
| GEODE7796_Platycnemididae_Elattoneura_acuta | 2017-07-27 | Bybee, S.M., R. A. Clement, S. Smith, J. F. Lontchi | Cameroon | Bouamir Tree Fall Stream; 3.18880N 12.83396E |  |
| GEODE7798_Libellulidae_Palpopleura_lucia | 2017-07-30 | Bybee, S.M., R. A. Clement, S. Smith, J. F. Lontchi | Cameroon | Somalomo River crossing; 3.37148N 12.73367E |  |
| RMNH.INS.506617_Idionychidae_Idionyx_philippa | 2012-06-11 | Cahilog, H. | The Philippines | Mindanao, Mindanao - Dispatcheng, Purok 5, Aliwagwag, Cateel, Davao Oriental; |  |
| RMNH.INS.506657_Corduliidae_Heteronaias_heterodoxa | 2012-06-13 | Cahilog, H. | The Philippines | Mindanao, Mindanao - Marahan, Marilog district, Davao City; |  |
| TM305_Coenagrionidae_Acanthagrion_trilobatum | 2017-01-01 | Cárdenas, B., J. Sandoval & C. Bota | Colombia | Risaralda, Pueblo Rico, Corregimiento Santa Cecilia, Vereda El Silencio; 5.347297; -76.134167 |  |
| RMNH.5009376_Gomphidae_Onychogomphus_styx | 2014-07-23 | Clausnitzer, V. | Uganda | Kibale National Park, around Field station; 0.55389, 30.36365 |  |
| GEODE17720_Corduliidae_Hemicordulia_kalliste | 2005-01-17 | Cleminson, G. | Australia | Queensland Ningi |  |
| GEODE17917_Gomphidae_Octogomphus_specularis | 1994-07-10 | Cole, J. | United States | California: Tuolumne Co., North Fork of the Tuolumne River at Lair of the Bear, Camp Blue, 2 miles southwest of Pinecrest Lake, Stanislaus national Forest. | M. Elevation 1700 meters. |
| GEODE17610_Aeshnidae_Gomphaeschna_furcillata | 2002-04-01 | Cook, C. | Florida | Okaloosa County, Niceville Robert Lake at rd #190 |  |
| OD1074_Hemiphlebiidae_Hemiphlebia_mirabilis |  | Cordero Rivera, A. | Australia | Nelson, Victoria; 141.137474, -38.110307 | Long swamp, WGS84, 11 |
| OD1083_Argiolestidae_Austroargiolestes_icteromelas | 2013-12-09 | Cordero Rivera, A. | Australia | Victoria, Coral Bank, Kiewa river; 147.093135, -36.583770 | 266 m elevation |
| OD1085_Rhipidolestidae_Agriomorpha_xinglongensis | 2014-05-29 | Cordero Rivera, A. | China | Hainan, Lingshui, Diaoluoshan, stream near road; 109.879155, 18.685422 | 574 m elevation |
| OD1283_Pseudolestidae_Pseudolestes_mirabilis |  | Cordero Rivera, A. | China | Diaoluoshan National Park, Lingshui, Hainan; 109.880018, 18.690587 | ACR-01154, WGS84, 601 |
| GEODE17728_Libellulidae_Orthemis_sulphurata | 20122-08-02 | Daigle, J.J. | Aruba | Aruba, ditch east of church, Calbas road, Noord | 2M. 900am. IORI #: 000094011. EX Jerrel J. Daigle Collection. Florida State Collection of Arthropods. Processed by: Bill Mauffray 12/5/2021 |
| GEODE17781_Corduliidae_Neurocordulia_virginiensis | 1981-05-21 | Daigle, J.J. | United States | Arkansas Montgomery Little Missouri River at Camp |  |
| GEODE17873_Austrocorduliidae_Lathrocordulia_metallica | 1979-12-15 | Davies, D.A.L. | Australia | Western Australia 120km S of Perth, Waroona, Nanga Brook |  |
| GEODE17885_Synthemistidae_Archaeosynthemis_leachii | 1979-12-09 | Davies, D.A.L. | Australia | Western Australia Nanga Brook, Waroona 120km S. of Perth |  |
| GEODE17888_Synthemistidae_Archaeosynthemis_spiniger | 1979-12-12 | Davies, D.A.L. | Australia | Western Australia Nanga Brook, Waroona 120km S. of Perth |  |
| GEODE17889_Synthemistidae_Austrosynthemis_cyanitincta | 1979-11-30 | Davies, D.A.L. | Australia | Western Australia Drake's Drain, Waroona |  |
| NESOMASC_Idomacromiidae_Nesocordulia_mascarenica | 2007 | Dijkstra, K.-D.B. | Madagascar |  |  |
| OD1305_Synlestidae_Chlorolestes_umbratus | 2015-12-23 | Dijkstra, K.-D.B. | Madagascar |  |  |
| OD1306_Platycnemididae_Elattoneura_glauca | 2015-12-23 | Dijkstra, K.-D.B. | Madagascar |  |  |
| OD1308_Protolestidae_Protolestes_kerckhoffae | 2016-01-13 | Dijkstra, K.-D.B. | Madagascar |  |  |
| OD1309_Argiolestidae_Nesolestes_sp | 2016-01-14 | Dijkstra, K.-D.B. | Madagascar |  |  |
| RMNH.5007797_Coenagrionidae_Azuragrion_nigridorsum | 2013-12-06 | Dijkstra, K.-D.B. | Zimbabwe | Manicaland, 21 km SSE of Chipinge, Mt Selinda-Chipinge road, Ruwadzano |  |
| RMNH.5010307_Coenagrionidae_Proischnura_polychromatica | 2015-02-14 | Dijkstra, K.-D.B. | South Africa | Western Cape, 22 km NE of Citrusdal, Cederberg, Driehoek; -32.45, 19.18 |  |
| RMNH.INS.229208_Coenagrionidae_Oreocnemis_phoenix | 2000-11-16 | Dijkstra, K.-D.B. | Malawi | Mt Mulanje, Dzole stream; |  |
| RMNH.INS.501444_Gomphidae_Libyogomphus_christinae | 2011-01-10 | Dijkstra, K.-D.B. | Liberia | Nimba County, East Nimba Nature Reserve, Yiti valley; 7.48, -8.57 |  |
| RMNH.INS.501662_Libellulidae_Eleuthemis_buettikoferi | 2011-02-25 | Dijkstra, K.-D.B. | Sierra Leone | Eastern Province, 7 km south of Kenema, Moa River at Tilorma; 7.81236, -11.17794 |  |
| RMNH.INS.502121_Libellulidae_Oxythemis_phoenicosceles | 2010-05-23 | Dijkstra, K.-D.B. | Democratic Republic of Congo | Province Orientale, Lower Aruwimi, Yayofa (=Yaofa); 1.267, 23.68 |  |
| RMNH.INS.502132_Calopterygidae_Phaon_iridipennis | 2010-05-23 | Dijkstra, K.-D.B. | Democratic Republic of Congo | Province Orientale, Lower Aruwimi, Yayofa (=Yaofa); 1.267, 23.68 |  |
| RMNH.INS.502150_Libellulidae_Chalcostephia_flavifrons | 2010-05-28 | Dijkstra, K.-D.B. | Democratic Republic of Congo | Province Orientale, Lower Lomami, Iloko stream on Lieki-Lileke path; 0.6548, 24.2685 |  |
| RMNH.INS.502177_Libellulidae_Notiothemis_robertsi | 2010-06-03 | Dijkstra, K.-D.B. | Democratic Republic of Congo | Province Orientale, Lower Lomami, Yendega, Bosega & Bogunu streams in forest west of Djabir & Yaengo; 0.51, 24.16 |  |
| RMNH.INS.502225_Libellulidae_Aethiothemis_erythromelas | 2010-05-02 | Dijkstra, K.-D.B. | Democratic Republic of Congo | Province Orientale, Yaekela; 0.81, 24.28 |  |
| RMNH.INS.502232_Aeshnidae_Gynacantha_victoriae | 2010-05-02 | Dijkstra, K.-D.B. | Democratic Republic of Congo | Province Orientale, Yaekela; 0.81, 24.28 |  |
| RMNH.INS.502285_Libellulidae_Cyanothemis_simpsoni | 2010-05-04 | Dijkstra, K.-D.B. | Democratic Republic of Congo | Province Orientale, Lilanda & Baombo streams at and upstream of Lilanda & Yafake villages; 0.83, 24.34 |  |
| RMNH.INS.502287_Libellulidae_Micromacromia_camerunica | 2010-05-04 | Dijkstra, K.-D.B. | Democratic Republic of Congo | Province Orientale, Lilanda & Baombo streams at and upstream of Lilanda & Yafake villages; 0.83, 24.34 |  |
| RMNH.INS.502304_Libellulidae_Parazyxomma_flavicans | 2010-05-11 | Dijkstra, K.-D.B. | Democratic Republic of Congo | Province Equateur, Lower Itimbiri, Kona camp; 2.033, 22.787 |  |
| RMNH.INS.502305_Libellulidae_Hadrothemis_camarensis | 2010-05-07 | Dijkstra, K.-D.B. | Democratic Republic of Congo | Province Orientale, Lilanda & Baombo streams at and upstream of Lilanda & Yafake villages; 0.83, 24.34 |  |
| RMNH.INS.502324_Neophyidae_Neophya_rutherfordi | 2010-05-04 | Dijkstra, K.-D.B. | Democratic Republic of Congo | Province Orientale, Lilanda & Baombo streams at and upstream of Lilanda & Yafake villages; 0.83, 24.34 |  |
| RMNH.INS.502394_Gomphidae_Lestinogomphus_congoensis | 2010-06-09 | Dijkstra, K.-D.B. | Democratic Republic of Congo | Province Orientale, Old Buta Road, PK 13 (Point Kilometre from Kisangani); 0.59193, 25.25786 |  |
| RMNH.INS.502397_Libellulidae_Malgassophlebia_bispina | 2010-06-08 | Dijkstra, K.-D.B. | Democratic Republic of Congo | Province Orientale, Ubundu Road, Yoko Forest, Mokonoka Stream; 0.29402, 25.2888 |  |
| RMNH.INS.502400_Libellulidae_Nesciothemis_farinosa | 2010-06-10 | Dijkstra, K.-D.B. | Democratic Republic of Congo | Province Orientale, Kisangani, Scolasticat; 0.545, 25.215 |  |
| RMNH.INS.502847_Libellulidae_Thermochoria_jeanneli | 2009-08-24 | Dijkstra, K.-D.B. | Tanzania | Pemba North Region, 13km N of Wete, Pemba Island, Ngezi Forest; -4.93, 39.7 |  |
| RMNH.INS.502891_Gomphidae_Nepogomphoides_stuhlmanni | 2009-08-26 | Dijkstra, K.-D.B. | Tanzania | Tanga Region, Amani Nature Reserve, Zigi; -5.10009, 38.65206 |  |
| RMNH.INS.504459_Libellulidae_Hemistigma_albipunctum | 2011-11-25 | Dijkstra, K.-D.B. | Democratic Republic of Congo | Katanga, Kundelungu National Park, 2 km ESE of Katwe; -10.57, 27.88 |  |
| RMNH.INS.505345_Chlorocyphidae_Platycypha_caligata | 2011-11-06 | Dijkstra, K.-D.B. | Democratic Republic of Congo | Katanga, Kiubo; -9.52, 27.05 |  |
| RMNH.INS.505354_Libellulidae_Trithetrum_navasi | 2011-11-06 | Dijkstra, K.-D.B. | Democratic Republic of Congo | Katanga, Kiubo; -9.52, 27.05 |  |
| RMNH.INS.505362_Libellulidae_Crocothemis_erythraea | 2011-11-06 | Dijkstra, K.-D.B. | Democratic Republic of Congo | Katanga, Kiubo; -9.52, 27.05 |  |
| RMNH.INS.505380_Libellulidae_Olpogastra_lugubris | 2011-11-07 | Dijkstra, K.-D.B. | Democratic Republic of Congo | Katanga, Kiubo; -9.52, 27.05 |  |
| RMNH.INS.505397_Gomphidae_Neurogomphus_cocytius | 2011-11-07 | Dijkstra, K.-D.B. | Democratic Republic of Congo | Katanga, Kiubo; -9.52, 27.05 |  |
| RMNH.INS.505547_Libellulidae_Porpax_risi | 2011-11-15 | Dijkstra, K.-D.B. | Democratic Republic of Congo | Katanga, Upemba National Park, Kabwekanono, source area of Kafwi; -8.937, 27.166 |  |
| RMNH.INS.505588_Libellulidae_Diplacodes_lefebvrii | 2011-11-17 | Dijkstra, K.-D.B. | Democratic Republic of Congo | Katanga, Upemba National Park, Mukana, west of Lusinga; -8.91, 27.18 |  |
| RMNH.INS.505652_Libellulidae_Bradinopyga_cornuta | 2011-11-22 | Dijkstra, K.-D.B. | Democratic Republic of Congo | Katanga, Kundelungu National Park, Masansa; -10.532, 27.978 |  |
| RMNH.INS.505653_Gomphidae_Notogomphus_praetorius | 2011-11-22 | Dijkstra, K.-D.B. | Democratic Republic of Congo | Katanga, Kundelungu National Park, Lutshipuka; -10.557, 27.958 |  |
| RMNH.INS.559644_Platycnemididae_Spesbona_angusta | 2013-10-27 | Dijkstra, K.-D.B. | South Africa | Western Cape, 6 km SE of Franschhoek, Hottentots-Holland Nature Reserve, Purgatory; -33.97, 19.175 |  |
| RMNH.INS.559789_Libellulidae_Zygonoides_fuelleborni | 2013-11-30 | Dijkstra, K.-D.B. | Zimbabwe | Manicaland, 62 km S of Mutare, Umvumvumvu river below Mutambara; -19.528, 32.622 |  |
| RMNH.INS.559802_Gomphidae_Crenigomphus_hartmanni | 2013-11-30 | Dijkstra, K.-D.B. | Zimbabwe | Manicaland, 62 km S of Mutare, Umvumvumvu river below Mutambara; -19.528, 32.622 |  |
| RMNH.INS.501518_Gomphidae_Microgomphus_camerunensis | 2011-01-22 | Dijkstra, K.-D.B. & A. Dayeker | Liberia | Grand Gedeh County, Putu Iron Ore Mining concession, Biodiversity Camp; 5.65921, -8.20411 |  |
| RMNH.INS.503108_Gomphidae_Diastatomma_gamblesi | 2011-03-01 | Dijkstra, K.-D.B. & A. Dayeker | Sierra Leone | Eastern Province, Gola Forest, Wibwa (Wegbua) stream at Mayengema; 7.64262, -10.78986 |  |
| RMNH.INS.503116_Calopterygidae_Sapho_fumosa | 2011-03-02 | Dijkstra, K.-D.B. & A. Dayeker | Sierra Leone | Eastern Province, Gola Forest, Gagbe stream, 1 km west of Mogbaima; 7.65743, -10.77889 |  |
| RMNH.INS.503184_Idomacromiidae_Idomacromia_proavita | 2011-03-11 | Dijkstra, K.-D.B. & A. Dayeker | Sierra Leone | Eastern Province, Gola Forest, Sembehun (abandoned village); 7.7598, -10.71747 |  |
| RMNH.5007835_Synlestidae_Ecchlorolestes_nylephtha | 2014-01-06 | Dijkstra, K.-D.B. & F.I. Grundlingh | South Africa | Eastern Cape, 49 km ENE of Plettenberg Bay, Garden Route National Park (Tsitsikamma Section), Big Tree off N2 at Stormrivier; -33.96667, 23.89519 |  |
| RMNH.5007870_Platycnemididae_Metacnemis_valida | 2014-01-09 | Dijkstra, K.-D.B. & F.I. Grundlingh | South Africa | Eastern Cape, 4 km SE of Stutterheim, Kubusi river at railway bridge; -32.59899, 27.45191 |  |
| RMNH.5008090_Gomphidae_Ceratogomphus_pictus | 2014-01-10 | Dijkstra, K.-D.B. & F.I. Grundlingh | South Africa | Eastern Cape, 19 km SSW of Dordrecht, R392 just N of Bankies and Zixinene; -31.53109, 26.96422 |  |
| RMNH.5008153_Idomacromiidae_Syncordulia_gracilis | 2014-01-19 | Dijkstra, K.-D.B. & F.I. Grundlingh | South Africa | Western Cape, between Kleinmond and Betty's Bay, Kogelberg Nature Reserve, Palmiet River at Oudebosch; -34.31542, 18.96283 |  |
| RMNH.5007803_Libellulidae_Atoconeura_biordinata | 2013-12-08 | Dijkstra, K.-D.B. & U. Bjelke | Zimbabwe | Manicaland, 9 km NE of Nyanga village, northside of Nyanga National Park, Troutbeck; -18.18311, 32.80346 |  |
| RMNH.5007810_Coenagrionidae_Africallagma_glaucum | 2013-12-08 | Dijkstra, K.-D.B. & U. Bjelke | Zimbabwe | Manicaland, 9 km NE of Nyanga village, northside of Nyanga National Park, Troutbeck; -18.18311, 32.80346 |  |
| RMNH.INS.502555_Gomphidae_Gomphidia_gamblesi | 2010-02-10 | Dijkstra, K.-D.B., N. Mézière & C. Vanappelghem | Gabon | Haut-Ogooué, 50km N of Franceville, Franceville-Okondja Road, Léconi River at bridge N of Onvouri-Olounga; -1.17758, 13.53886 |  |
| GEODE17622_Macromiidae_Macromia_astarte | 1972-10-25 | Donnelly, N. | Papua New Guinea | Morobe Dist, stream at Gurakor, Wau Rd. | 500 m |
| GEODE17711_Corduliidae_Guadalca_insularis | 1987-02-13/24 | Donnelly, N. | Solomon Islands | Guadalcanal Honiara Rove Cr. At Reservoir |  |
| GEODE17883_Pseudocorduliidae_Pseudocordulia_circularis | 1972-11-27 | Donnelly, N. | Australia | Queensland Tchooratippa Cr., 30mi W. of Innisfail 2000ft |  |
| GEODE17887_Synthemistidae_Archaeosynthemis_orientalis | 1972-12-30 | Donnelly, N. | Australia | A.C.T (Australian Capital Territory) Ginini Flats at Mt. Ginini 5200ft |  |
| GEODE7866_Platycnemididae_Lieftinckia_malaitae | 1987-02-19 | Donnelly, N. | Solomon Islands |  |  |
| GEODE17607_Aeshnidae_Andaeschna_rufipes | 1997-01-15 | Donnelly, T.W. | United States | Argentina, Jujuy; P.N. Calilegua; Ao. Tres Cruces (23°41.73'S 64°52.18'W; 1250m) 97x023 |  |
| GEODE17870_Idionychidae_Idionyx_optatus | 1998-07-21/22 | Donnelly, T.W. | Thailand | Chieng Mai Siriphum waterfall, nr Doi Inthanon 1000m |  |
| GEODE17874_Macromidiidae_Macromidia_genialis | 1994-07-23/24 | Donnelly, T.W. | Thailand | Chantaburi Khao Soi Dao N.P. |  |
| GEODE17881_Idomacromiidae_Neocordulia_griphus | 1976-08-24 | Donnelly, T.W. | Guatemala | Zacapa Quebrada La Ceiba, 2km W of Juan de Paz 100m |  |
| GEODE7827_Coenagrionidae_Amphiallagma_parvum | 2001-06-18 | Donnelly, T.W. | Myanmar | Kandawgyi Botanical Garden; 21.093N, 96.470167E |  |
| GEODE7863_Megapodagrionidae_Allopodagrion_contortum | 1997-01-23 | Donnelly, T.W. | Argentina | 25°37.48'S 54°35.19'W |  |
| GEODE7874_Thaumatoneuridae_Paraphlebia_duodecima | 2007-06-11 | Donnelly, T.W. | Guatemala | near Riachuelo Colorado tiny waterfall; 15°12.98'N 90°13.27'W |  |
| GEODE17717_Corduliidae_Hemicordulia_fidelis | 2013-04-14 | Donnelly, T.W. & J. Michalski | New Caledonia | Nord Province Highway RP5 (Sarramea - Canala Rd) stream and picnic area 310m |  |
| GEODE17896_Synthemistidae_Calesynthemis_campioni | 0203-04-04 | Donnelly, T.W. & J. Michalski | New Caledonia | Sud highway RP5 (Sarramea-Canala road) small stream; 330m |  |
| GEODE7821_Argiolestidae_Eoargiolestes_ochraceus | 2013-04-05 | Donnelly, T.W. & J. Michalski | New Caledonia | highway RP5 (Sarramea-Canala Road), stream and picnic area; S21.5641, E165, 8319 |  |
| RMNH.5008306_Libellulidae_Neurothemis_fluctuans | 2014-03-19 | Dow, R.A. | Malaysia | Sarawak, Bintulu, Samarakan - T2N - stream with buffer near nursery; |  |
| RMNH.5008334_Argiolestidae_Podolestes_orientalis | 2014-03-18 | Dow, R.A. | Malaysia | Sarawak, Bintulu, Samarakan - Sg Philip; |  |
| RMNH.5008397_Platystictidae_Telosticta_ulubaram | 2014-08-25 | Dow, R.A. | Malaysia | Sarawak, Miri Division, Ulu Moh; |  |
| RMNH.INS.228976_Libellulidae_Tetrathemis_irregularis | 2008-01-02 | Dow, R.A. | Malaysia | Sarawak, Miri Division, Gunung Mulu NP, Swamp on trail to Deer Cave; |  |
| RMNH.INS.228999_Libellulidae_Raphismia_bispina | 2008-02-02 | Dow, R.A. | Malaysia | Sarawak, Bintulu Division, Similajau NP,; |  |
| RMNH.INS.500989_Chlorocyphidae_Libellago_aurantiaca | 2010-11-04 | Dow, R.A. | Malaysia | Sarawak, Miri Division, Lambir Hills area, Simon La'ang's farm; 4.2362166667, 113.98548333 |  |
| RMNH.INS.501024_Chlorocyphidae_Sundacypha_petiolata | 2010-11-20 | Dow, R.A. | Malaysia | Sarawak, Bintulu Division, Anap Muput, Sg Sebelalang; 2.3433833333, 112.80668333 |  |
| RMNH.INS.501070_Calopterygidae_Echo_modesta | 2010-12-11 | Dow, R.A. | Malaysia | Pahang, Kuala Tahan, Tributary 3 to Sg Tembeling; 4.38155, 102.42205 |  |
| RMNH.INS.501089_Euphaeidae_Dysphaea_dimidiata | 2010-12-10 | Dow, R.A. | Malaysia | Pahang, Kuala Tahan,; |  |
| RMNH.INS.501123_Libellulidae_Acisoma_panorpoides | 2010-12-17 | Dow, R.A. | Malaysia | Pahang, Gelanggi Caves; 3.8998, 102.47331667 |  |
| RMNH.INS.501127_Devadattidae_Devadatta_argyoides | 2010-12-09 | Dow, R.A. | Malaysia | Pahang, Kuala Tahan, Tributary 1 to Sg Tembeling; 4.39755, 102.4307 |  |
| RMNH.INS.501152_Libellulidae_Pseudothemis_jorina | 2010-12-14 | Dow, R.A. | Malaysia | Pahang, Kuala Tahan,; 4.3719833333, 102.40291667 |  |
| RMNH.INS.501184_Gomphidae_Phaenandrogomphus_asthenes | 2010-12-12 | Dow, R.A. | Malaysia | Pahang, Kuala Tahan, Tributary 1 to Sg Tembeling; 4.39755, 102.4307 |  |
| RMNH.INS.501189_Libellulidae_Aethriamanta_brevipennis | 2010-12-17 | Dow, R.A. | Malaysia | Pahang, Gelanggi Caves; 3.8998, 102.47331667 |  |
| RMNH.INS.501196_Libellulidae_Rhodothemis_rufa | 2010-12-18 | Dow, R.A. | Malaysia | Pahang, Gelanggi Caves; 3.8998, 102.47331667 |  |
| RMNH.INS.501275_Libellulidae_Orchithemis_xanthosoma | 2010-05-28 | Dow, R.A. | Malaysia | Sarawak, Kuching, Matang Rd, remnant peatswamp/rubber; 1.5736, 110.27875 |  |
| RMNH.INS.501284_Libellulidae_Brachygonia_oculata | 2010-05-10 | Dow, R.A. | Malaysia | Sarawak, Sibu, Sg. Teku area; 2.3503, 111.86538333 |  |
| RMNH.INS.501296_Libellulidae_Tholymis_tillarga | 2010-06-19 | Dow, R.A. | Malaysia | Sarawak, Bintulu, Camp C; 2.72445, 113.3492 |  |
| RMNH.INS.501373_Libellulidae_Chalybeothemis_fluviatilis | 2009-09-21 | Dow, R.A. | Malaysia | Pahang, Sg Bebar; 3.3116, 103.23533333 |  |
| RMNH.INS.501377_Libellulidae_Zyxomma_petiolatum | 2009-09-21 | Dow, R.A. | Malaysia | Pahang, Sg Bebar, swamp downstream; 3.2608333333, 103.24478333 |  |
| RMNH.INS.501398_Libellulidae_Pornothemis_sp | 2009-09-22 | Dow, R.A. | Malaysia | Pahang, Sg Bebar, swamp upstream; 3.3116, 103.23533333 |  |
| RMNH.INS.503416_Libellulidae_Lyriothemis_cleis | 2010-06-25 | Dow, R.A. | Malaysia | Sarawak, Bintulu, Camp C waterpipe path; 2.7192, 113.34625 |  |
| RMNH.INS.503419_Libellulidae_Nesoxenia_lineata | 2010-06-30 | Dow, R.A. | Malaysia | Sarawak, Bintulu, Kakus nursery, swamp near stream; 2.7701166667, 113.21476667 |  |
| RMNH.INS.503523_Gomphidae_Sieboldius_japponicus | 2010-05-07 | Dow, R.A. | Malaysia | Sarawak, Miri, Mulu, sg Melinau Paku upstream of w/f; 4.0475666667, 114.83643333 |  |
| RMNH.INS.503524_Gomphidae_Acrogomphus_jubilaris | 2010-05-24 | Dow, R.A. | Malaysia | Sarawak, Kapit, Hose range, perched at side of tractor path in morning, ca 900m; 2.07965, 113.69446667 |  |
| RMNH.INS.503619_Coenagrionidae_Aciagrion_borneense | 2010-06-19 | Dow, R.A. | Malaysia | Sarawak, Bintulu, Camp C; 2.72445, 113.3492 |  |
| RMNH.INS.503929_Platycnemididae_Calicnemia_chaseni | 2011-08-21 | Dow, R.A. | Malaysia | Terengganu, Small stream near Sg Lasir; |  |
| RMNH.INS.503939_Libellulidae_Hydrobasileus_croceus | 2011-08-22 | Dow, R.A. | Malaysia | Terengganu, Pond just outside Sekayu Rec Forest; |  |
| RMNH.INS.503943_Libellulidae_Indothemis_limbata | 2011-08-22 | Dow, R.A. | Malaysia | Terengganu, Pond just outside Sekayu Rec Forest; |  |
| RMNH.INS.503944_Platycnemididae_Pseudocopera_ciliata | 2011-08-22 | Dow, R.A. | Malaysia | Terengganu, Open stream just outside Sekayu Rec Forest; |  |
| RMNH.INS.503951_Coenagrionidae_Argiocnemis_rubescens | 2011-08-23 | Dow, R.A. | Malaysia | Terengganu, Bukit Kesing Recreational forest, muddy trail pool; |  |
| RMNH.INS.503957_Libellulidae_Potamarcha_congener | 2011-08-24 | Dow, R.A. | Malaysia | Terengganu, Marshy area by Sg Peres just outside Sekayu Rec Forest; |  |
| RMNH.INS.503963_Libellulidae_Lathrecista_asiatica | 2011-08-24 | Dow, R.A. | Malaysia | Terengganu, Marshy area by Sg Peres just outside Sekayu Rec Forest; |  |
| RMNH.INS.503964_Libellulidae_Urothemis_signata | 2011-08-24 | Dow, R.A. | Malaysia | Terengganu, Pond near Sekayu Rec Forest; |  |
| RMNH.INS.504032_Libellulidae_Onychothemis_culminicola | 2011-09-14 | Dow, R.A. | Malaysia | Sarawak, Bintulu, T1C stream, SPFP; |  |
| RMNH.INS.504039_Libellulidae_Orchithemis_pulcherrima | 2011-09-19 | Dow, R.A. | Malaysia | Sarawak, Bintulu, T1?, small stream in acacia, upstream of road; |  |
| RMNH.INS.506247_Macromidiidae_Macromidia_fulva | 2012-03-23 | Dow, R.A. | Malaysia | Sarawak, Bintulu, Camp stream; |  |
| RMNH.INS.506254_Libellulidae_Camacinia_gigantea | 2012-03-27 | Dow, R.A. | Malaysia | Sarawak, Bintulu, Bukit Kana/Bukit Naong - logging track on lower slopes; |  |
| RMNH.INS.506261_Platystictidae_Telosticta_bidayuh | 2012-03-30 | Dow, R.A. | Malaysia | Sarawak, Kuching, Kubah, high stream; |  |
| RMNH.INS.506264_Gomphidae_Leptogomphus_coomansi | 2012-04-01 | Dow, R.A. | Malaysia | Sarawak, Kuching, MWC - Sg Rayu; |  |
| RMNH.INS.506290_Libellulidae_Cratilla_lineata | 2012-04-06 | Dow, R.A. | Malaysia | Sarawak, Miri, Outside Jaman's lodge, ditches etc; |  |
| RMNH.INS.506367_Gomphidae_Megalogomphus_borneensis | 2012-05-13 | Dow, R.A. | Malaysia | Sarawak, Miri, Mulu - BO stream; |  |
| RMNH.INS.506835_Philosinidae_Rhinagrion_macrocephalum | 2012-08-02 | Dow, R.A. | Malaysia | Johor, Gn Belumut - backwater & trib to main stream; |  |
| RMNH.INS.506912_Aeshnidae_Indaeschna_grubaueri | 2012-09-30 | Dow, R.A. | Malaysia | Sarawak, Kuching, Kubah - frog pond; |  |
| RMNH.INS.507659_Platystictidae_Drepanosticta_actaeon | 2012-09-10 | Dow, R.A. | Malaysia | Sabah, Poring - stream running into Bamboo garden; |  |
| RMNH.INS.507703_Coenagrionidae_Stenagrion_dubium | 2012-09-13 | Dow, R.A. | Malaysia | Sabah, Liwagu at side; |  |
| RMNH.INS.507707_Chlorocyphidae_Rhinoneura_villosipes | 2012-09-13 | Dow, R.A. | Malaysia | Sabah, Kinabalu HQ - Silau-Silau; |  |
| RMNH.INS.507722_Calopterygidae_Matronoides_cyaneipennis | 2012-09-14 | Dow, R.A. | Malaysia | Sabah, Sayap - sg Kemantis; |  |
| RMNH.INS.507746_Libellulidae_Hylaeothemis_clementia | 2012-09-17 | Dow, R.A. | Malaysia | Sabah, Sayap - drain by road; |  |
| RMNH.INS.507799_Calopterygidae_Vestalis_amaryllis | 2012-09-29 | Dow, R.A. | Malaysia | Sarawak, Kuching, Kubah - stream behind rest house; |  |
| RMNH.INS.507831_Libellulidae_Tyriobapta_torrida | 2012-10-05 | Dow, R.A. | Malaysia | Sarawak, Kuching, Kpg Sebako - slow stream & swamp area; |  |
| RMNH.INS.557636_Coenagrionidae_Pericnemis_stictica | 2013-08-07 | Dow, R.A. | Malaysia | Sarawak, Bintulu, Bukit Sarang - swamp and small streams on trail to clearings; |  |
| GEODE17832_Corduliidae_Somatochlora_cingulata | 2000-08-01 | Dunkle, S.W. | Canada | British Columbia Solco Lake & Fen, E of Okanagan Falls 5600ft |  |
| GEODE17891_Synthemistidae_Eusynthemis_guttata | 2003-01-07 | Dunkle, S.W. | Australia | Victoria Eurobin Creek at entrance to Buffalo National Park |  |
| GEODE7836_Coenagrionidae_Diceratobasis_melanogaster | 1989-06-01 | Dunkle, S.W. | Dominican Republic | stream on road to Constanza, 8.4 km from Autopista Duarte |  |
| OD146_Corduliidae_Cordulia_aenea | 1999-12-12 | Ekestubbe, K. | Sweden | Sverige, So, Sodertalje Kn 656016 159643 | male |
| OD1314_Gomphidae_Ophiogomphus_morrisoni | 2016-10-20 | Erickson, R.J. | United States | Utah Beaver County Beaver River, Hanging Rock Recreation Site N38.21437° W112.86496° |  |
| GEODE7800_Libellulidae_Diplacodes_trivialis | 2018-07-16 | Erickson, R.J. , Y. M. Pacheco & S. M. Bybee | Vietnam | Near checkpoint station; 22.45518N 105.67502E |  |
| GEODE7801_Libellulidae_Crocothemis_servilia | 2018-07-16 | Erickson, R.J. , Y. M. Pacheco & S. M. Bybee | Vietnam | Stream outside of Ba Be N.P.; 22.42314N 105.64756E |  |
| GEODE7811_Platycnemididae_Copera_marginipes | 2018-07-17 | Erickson, R.J., Y. M. Pacheco & S. M. Bybee | Vietnam | Cuc Phuong N.P., Hotel; N20.2501, E105.71429 |  |
| GEODE7721_Calopterygidae_Neurobasis_chinensis | 2018-07-15 | Erickson, R.J., Y. M. Pacheco, S. M. Bybee | Vietnam | Ba Be N.P, trail to waterfall; N22.42055, E105.63834 |  |
| GEODE7724_Platycnemididae_Coeliccia_cyanomelas | 2018-07-12 | Erickson, R.J., Y. M. Pacheco, S. M. Bybee | Vietnam | Pia Oac Nature Reserve |  |
| GEODE7727_Platycnemididae_Prodasineura_autumnalis | 2018-07-16 | Erickson, R.J., Y. M. Pacheco, S. M. Bybee | Vietnam | Stream outside of Ba Be N.P; N22.42314, E105.64756 |  |
| GEODE7812_Chlorocyphidae_Heliocypha_perforata | 2018-07-16 | Erickson, R.J., Y. M. Pacheco, S. M. Bybee | Vietnam | Stream outside of Ba Be N.P; N22.42314, E105.64756 |  |
| GEODE7802_Libellulidae_Aethriamanta_rezia | 2020-12-20 | Fomekong Lontchi, J. | Cameroon | Mbalmayo 4; N3.4848 E11.5010 |  |
| GEODE7804_Libellulidae_Tetrathemis_fraseri | 2020-12-20 | Fomekong Lontchi, J. | Cameroon | Mbalmayo 1; N3.4741 E11.4006 |  |
| GEODE7806_Libellulidae_Orthetrum_julia | 2020-07-27 | Fomekong Lontchi, J. | Cameroon | Mbalmayo 6; N3.4697 E11.5004 |  |
| GEODE7624_Platycnemididae_Disparoneura_apicalis | 1923-05-06 | Fraser, F.C. | India | S.India, Cauvery River, Fraserpet, Coorg |  |
| GEODE7873_Synlestidae_Phylolestes_ethelae | 1980-08-25 | Garrison, R.W. | Dominican Republic | small stream on Constaza rd. 14 km E of Autopista Duarte |  |
| GEODE7879_Coenagrionidae_Forcepsioneura_sancta | 2000-12-02 | Garrison, R.W. | Brazil | Ilha de Marambaia, Gruta da Santa; 23 3'35" S, 43 57'56" W |  |
| RMNH.INS.500397_Argiolestidae_Allolestes_maclachlanii | 2008-12-12 | Gerlach, J. | Seychelles | Mahe,; |  |
| RMNH.INS.500398_Coenagrionidae_Leptocnemis_cyanops | 2008-12-12 | Gerlach, J. | Seychelles | Mahe,; |  |
| GEODE7628_Libellulidae_Rhodopygia_hinei | 1936-03-19 | Gertsch, W.J. | Panama | Barro Colorado Island |  |
| GEODE17757_Libellulidae_Archaeophlebia_martini | 1963-01-16/20 | Gould | Madagascar | Perinet |  |
| GEODE7669_Aeshnidae_Amphiaeschna_ampla | 1941-05-16 | Groenendael, J.M.A. | Indonesia | West Java, Gunung Gedeh | RMNH.ins.1153207 |
| OD132_Euphaeidae_Euphaea_ochracea |  | Grummel | Thailand | Chiay Mai Prov., Doi Sutheds Matl Parks |  |
| TM154_Polythoridae_Polythore_gigantea | 2016-08-23 | Guzmán, C., J. Sandoval & C. Bota | Colombia | Parque Nacional Natural Tatamá, Vereda Monte Bello, La Clarita |  |
| GEODE12747_Libellulidae_Amphithemis_curvistyla | 1999-10-20 | Hamalainen, M. | Thailand | Kanchanaburi, Thong Pha Phum area, Nam Tok Nang Kroan |  |
| GEODE12754_Gomphidae_Mattigomphus_pinratani | 1999-05-19 | Hamalainen, M. | Thailand | Chiang Mai, Doi Inthanon, 1300 m, Siriphum wf. |  |
| RMNH.INS.502054_Calopterygidae_Caliphaea_confusa | 2009-05-20 | Hamalainen, M. | Vietnam | Lao Cai, Sapa, Hoang Lien, small streams between 22°20'59"N 103°46'18"E and 22°20'51"N 103°46'10"E; 22.35, 103.772 |  |
| RMNH.INS.505736_Chlorocyphidae_Aristocypha_fenestrella | 2009-01-15 | Hamalainen, M. | Thailand | Ranong, Khlong Nakha, Khlong Bangmun; |  |
| RMNH.INS.505747_Calopterygidae_Vestalaria_smaragdina | 2009-01-08 | Hamalainen, M. | Thailand | Chiang Mai, Near the Headquarters Garden, Doi Suthep, Stream below Pau Pau w/f; |  |
| RMNH.INS.505754_Gomphidae_Amphigomphus_somnuki | 2009-06-04 | Hamalainen, M. | Thailand | Chiang Mai, Doi Suthep, Pau Pau w/f; |  |
| RMNH.INS.505757_Euphaeidae_Anisopleura_furcata | 2002-05-20 | Hamalainen, M. | Thailand | Chiang Mai, Doi Inthanon,; |  |
| RMNH.INS.505806_Calopterygidae_Psolodesmus_kuroiwae |  |  | Japan | Okinawa |  |
| RMNH.INS.505817_Euphaeidae_Cryptophaea_vietnamensis | 2009-04-28 | Hamalainen, M. | Laos | Lak Sao; |  |
| RMNH.INS.506232_Platycnemididae_Matticnemis_doi | 2010-06-21 | Hamalainen, M. | Vietnam | Lang Son, Huu Lien, Tan Lai village, Hang Chau stream; |  |
| OD122_Lestidae_Sympecma_paedisca | 2002-08-27 | Jarvis & Taylor | Russia | Altaiskii Krai, Barnaul City, SE side, 2 km from Ob River |  |
| SRR1695233_Ephemeroptera_Ecdyonurus_insignis | 2013-06-05 | Jeanne Wilbrandt, Tanja Ziesmann, Oliver Niehuis, Ralph Peters | Germany | North Rhine-Westphalia, Donrath |  |
| OD1172_Austrocorduliidae_Austrocordulia_refracta |  | JLW | Australia |  |  |
| OD1183_Mesagrionidae_Mesagrion_leucorrhinum |  | JLW | Colombia |  |  |
| GEODE7767_Libellulidae_Nephepeltia_phryne | 2016-11-12 | Juillerat, J. & N. Mézière | French Guiana | Sinnamary, 3.611356, -53.174503 | 121116-6, male |
| GEODE7785_Coenagrionidae_Inpabasis_rosea | 2016-11-04 | Juillerat, J. & N. Mézière | French Guiana | Saül; 3.603129, -53.208978 | 041116-33, male |
| GEODE7786_Coenagrionidae_Acanthallagma_luteum | 2016-11-02 | Juillerat, J. & N. Mézière | French Guiana | Saül; 3.611356, -53.174503 |  |
| GEODE12746_Libellulidae_Akrothemis_risi | 2009-07-25 | Kaize, J. | Indonesia | Province Papua, Kab. Asmat, Vrinedschap R.; S05 20 20, E138 52 72 |  |
| GEODE2758_Coenagrionidae_Plagulibasis_ciliata | 2009-07-12 | Kaize, J. | Indonesia | Prov Papua, Kab. Mappi-Katan, S06'40,60 E139'14.87 |  |
| RMNH.INS.502075_Argiolestidae_Metagrion_ornatum | 2009-08-18 | Kaize, J. | Indonesia | Papua Barat, Kabupaten Wondama,, Mioswaar, Pantai Barat, Waprak; |  |
| OD1126_Heteragrionidae_Dimeragrion_percubitale |  | Kalkman, V.J. | Venezuela |  |  |
| OD1130_Philosinidae_Philosina_buchi |  | Kalkman, V.J. | China |  |  |
| OD1132_Platycnemididae_Onychargia_atrocyana |  | Kalkman, V.J. | Malaysia |  |  |
| OD1133_Amanipodagrionidae_Amanipodagrion_gilliesi |  | Kalkman, V.J. | Tanzania |  |  |
| OD1135_Thaumatoneuridae_Paraphlebia_sp |  | Kalkman, V.J. | Mexico |  |  |
| OD1136_Protolestidae_Protolestes_fickei |  | Kalkman, V.J. | Madagascar |  |  |
| OD1137_Tatocnemididae_Tatocnemis_malgassica |  | Kalkman, V.J. | Madagascar |  |  |
| OD1139_Pentaphlebiidae_Pentaphlebia_mangana |  | Kalkman, V.J. | Gabon |  |  |
| OD1141_Thaumatoneuridae_Thaumatoneura_inopinata |  | Kalkman, V.J. | Costa Rica |  |  |
| OD1143_Platycnemididae_Mesocnemis_singularis |  | Kalkman, V.J. | South Africa |  |  |
| OD1146_Hypolestidae_Hypolestes_sp |  | Kalkman, V.J. | Dominican Republic |  |  |
| OD1148_Amphipterygidae_Amphipteryx_agrioides |  | Kalkman, V.J. | Dominican Republic |  |  |
| OD1278_Lestidae_Indolestes_cyaneus | 2016-08-13 | Kalkman, V.J. | Bhutan |  |  |
| OD1302_Epiophlebiidae_Epiophlebia_laidlawi |  | Kalkman, V.J. | Bhutan |  | RMNH.5011536 |
| OD1303_Aeshnidae_Gynacanthaeschna_sikkima |  | Kalkman, V.J. | Bhutan | Chirang, Kikhorthang | RMNH.5015076 |
| OD1304_Cordulegastridae_Neallogaster_hermionae |  | Kalkman, V.J. | Bhutan |  |  |
| RMNH.5011399_Gomphidae_Stylurus_flavipes | 2014-08-27 | Kalkman, V.J. | Netherlands | Noord-Brabant, Klundert - Kleine Ton; |  |
| RMNH.INS.229243_Isostictidae_Selysioneura_capreola | 2006-08-02 | Kalkman, V.J. | Indonesia | Papua, Abepura,; -2.582, 140.651 |  |
| RMNH.INS.229291_Platycnemididae_Torrenticnemis_filicornis | 2008-10-27 | Kalkman, V.J. | Indonesia | Papua, Walmak (Nipsan),; -4.117, 139.633 |  |
| RMNH.INS.500513_Platycnemididae_Lochmaeocnemis_malacodora | 2006-07-28 | Kalkman, V.J. | Indonesia | Papua, Star mountains, Borme; -4.396, 140.434 |  |
| RMNH.INS.500518_Corduliidae_Procordulia_leopoldi | 2006-07-29 | Kalkman, V.J. | Indonesia | Papua, a few km from Borme village, Star mountains, Borme; -4.392, 140.428 |  |
| RMNH.INS.500525_Synthemistidae_Palaeosynthemis_primigenia | 2006-07-27 | Kalkman, V.J. | Indonesia | Papua, Star mountains, Borme; -4.396, 140.434 |  |
| RMNH.INS.500580_Platycnemididae_Palaiargia_charmosyna | 2006-08-02 | Kalkman, V.J. | Indonesia | Papua, Abepura,; -2.582, 140.651 |  |
| RMNH.INS.500592_Libellulidae_Huonia_epinephela | 2006-07-11 | Kalkman, V.J. | Indonesia | Papua, Yapen, Ambaidiru village; -1.763, 136.179 |  |
| RMNH.INS.500639_Platycnemididae_Idiocnemis_obliterata | 2008-10-25 | Kalkman, V.J. | Indonesia | Papua, Lelambo,; -4.033, 139.787 |  |
| RMNH.INS.500644_Libellulidae_Protorthemis_coronata | 2008-10-25 | Kalkman, V.J. | Indonesia | Papua, Lelambo,; -4.033, 139.787 |  |
| RMNH.INS.501977_Platycnemididae_Arrhenocnemis_parvibullis | 2009 | Kalkman, V.J. | Papua New Guinea |  |  |
| RMNH.INS.504852_Coenagrionidae_Austrocoenagrion_lyelli | 2011-12-21 | Kalkman, V.J. | Australia | NSW, Guyra, Mother of Duck Lagoon, SE corner,; -30.2466, 151.6667 |  |
| RMNH.INS.504908_Aeshnidae_Telephlebia_godeffroyi | 2011-12-30 | Kalkman, V.J. | Australia | NSW, Blue Mountains, Blackheath, Govetts Leap Brook,; -33.6328, 150.313 |  |
| RMNH.INS.504910_Synthemistidae_Synthemis_eustalacta | 2011-12-31 | Kalkman, V.J. | Australia | NSW, Kanangra Boyd NP, Boyd River Campground,; -33.9702, 150.057 |  |
| RMNH.INS.504941_Aeshnidae_Notoaeschna_sagittata | 2012-01-01 | Kalkman, V.J. | Australia | NSW, Blue Mountains, Blackheath, Govetts Leap Brook,; -33.6328, 150.313 |  |
| RMNH.INS.504961_Gomphidae_Austrogomphus_ochraceus | 2012-01-04 | Kalkman, V.J. | Australia | NSW, Blue Mountains, Glenbrook, Bruce Road,; -33.7788, 150.6184 |  |
| RMNH.INS.504999_Libellulidae_Orthetrum_cancellatum | 2012-07-24 | Kalkman, V.J. | Netherlands | Meinweg; 51.15, 6.12 |  |
| RMNH.INS.505016_Aeshnidae_Anax_imperator | 2012-06-02 | Kalkman, V.J. | Netherlands | Leidse Hout; 52.17, 4.47 |  |
| RMNH.INS.505017_Aeshnidae_Isoaeshna_isoceles | 2012-06-02 | Kalkman, V.J. | Netherlands | Leidse Hout; 52.17, 4.47 |  |
| RMNH.INS.505254_Coenagrionidae_Austrocnemis_splendida | 2011-12-09 | Kalkman, V.J. | Australia | Queensland, Mapleton, Mapleton, Lilyponds park,; -26.622, 152.8643 |  |
| RMNH.INS.507392_Libellulidae_Leucorrhinia_rubicunda | 2012-05-18 | Kalkman, V.J. | Netherlands | Bosbeek, Meinweg; 51.16, 6.09 |  |
| RMNH.INS.507400_Libellulidae_Libellula_depressa | 2012-05-19 | Kalkman, V.J. | Netherlands | Meinweg; 51.156, 6.137 |  |
| RMNH.INS.507402_Aeshnidae_Brachytron_pratense | 2012-05-19 | Kalkman, V.J. | Netherlands | Meinweg; 51.151, 6.125 |  |
| RMNH.INS.507439_Libellulidae_Libellula_quadrimaculata | 2012-05-26 | Kalkman, V.J. | Netherlands | Leidse Hout; 52.17, 4.47 |  |
| RMNH.INS.507448_Lestidae_Chalcolestes_viridis | 2012-08-01 | Kalkman, V.J. | Netherlands | De Wolden; 533, 224 |  |
| RMNH.INS.507465_Libellulidae_Sympetrum_danae | 2012-08-18 | Kalkman, V.J. | Netherlands | Balloerveld; 558, 239 |  |
| RMNH.INS.507492_Libellulidae_Nannodiplax_rubra | 2012-10-13 | Kalkman, V.J. | Australia | Northern Territory, Howard Spring, old swimming pool; -12.4565, 131.0522 |  |
| OD1277_Isostictidae_Neosticta_fraseri | 2015-01-10 | Kalkman, V.J. & G. Theischinger | Australia | Queensland, Wooroonooran Park, Gooligans creek, stream; -17.6048 S 145.7622 E |  |
| RMNH.5011424_Lestoideidae_Diphlebia_euphoeoides | 2014-12-31 | Kalkman, V.J. & G. Theischinger | Australia | Queensland, Station Creek, west of Mt Molloy; 145.2226, -16.6121 |  |
| RMNH.5011429_Gomphidae_Hemigomphus_comitatus | 2014-12-31 | Kalkman, V.J. & G. Theischinger | Australia | Queensland, Station Creek, west of Mt Molloy; 145.2226, -16.6121 |  |
| RMNH.5011432_Synthemistidae_Tonyosynthemis_claviculata | 2014-12-31 | Kalkman, V.J. & G. Theischinger | Australia | Queensland, Station Creek, west of Mt Molloy; 145.2226, -16.6121 |  |
| RMNH.5011475_Isostictidae_Oristicta_filicicola | 2015-01-07 | Kalkman, V.J., G. Theischinger & S. Richards | Australia | Queensland, Wooroonooran Park, Josephine Falls; 145.8594, -17.4363 |  |
| RMNH.5011478_Gomphidae_Austrogomphus_prasinus | 2015-01-08 | Kalkman, V.J., G. Theischinger & S. Richards | Australia | Queensland, Little Mulgrave, Gillies Hw; 145.7232, -17.1405 |  |
| RMNH.5011485_Synlestidae_Chorismagrion_risi | 2015-01-09 | Kalkman, V.J., G. Theischinger & S. Richards | Australia | Queensland, Paluma Range NP, Paluma Crystal Creek, first small creek; 146.2645, -190163 |  |
| OD1076_Epiophlebiidae_Epiophlebia_superstes | 2013-05-06 | Karube, H. | Japan | Sagamihara city, Shiroyama tawn, Amefurasshi |  |
| RMNH.INS.507575_Macromiidae_Macromia_splendens | 2012-06-18 | Keijl, G.O. | France | Mialet; 44.106, 3.948 |  |
| RMNH.INS.507576_Idomacromiidae_Oxygastra_curtisii | 2012-06-18 | Keijl, G.O. | France | Mialet; 44.106, 3.948 |  |
| GEODE17756_Libellulidae_Antidythemis_trameiformis | 1963-01 | Kesselring, J. | Brazil | Pará, Belém |  |
| RMNH.INS.507929_Gomphidae_Phyllogomphus_annulus | 2012-05-19 | Kipping, J. | Angola | Cuando-Cubango, Cuanavale River NE of Cuito Cuanavale,; -15.13569, 19.19571 |  |
| RMNH.INS.508001_Libellulidae_Zygonyx_torridus | 2010-03-08 | Kipping, J. | Zambia | Livingstone, Livingstone, Maramba River Lodge; -17.887817, 25.854138 |  |
| RMNH.INS.508012_Gomphidae_Paragomphus_genei | 2010-01-12 | Kipping, J. | Botswana | Ngamiland, Meno-a-Kwena Camp, Boteti; -20.32262, 24.31857 |  |
| RMNH.INS.509207_Gomphidae_Burmagomphus_divaricatus | 2011-08-23 | Kosterin, O. | Cambodia | Koh Kong,; |  |
| RMNH.INS.509920_Gomphidae_Lamelligomphus_castor |  | Kosterin, O. | Cambodia |  |  |
| RMNH.INS.509971_Gomphidae_Orientogomphus_minor | 2013-06-02 | Kosterin, O. | Cambodia | Rattanakiri, O'Sieng Lei waterfalls; |  |
| GEODE7691_Aeshnidae_Heliaeschna_crassa | 1936-09-26 | Kuiper, F.J. | Indonesia | W Billiton, Tandjong Pandan | RMNH.ins.1153186 |
| GEODE17761_Libellulidae_Boninthemis_insularis | 1977-06-18 | Kuwahara | Japan | Okimura-Mt. Funeki Hahashima, Ogasawara-ls Tokyo-To |  |
| GEODE7854_Coenagrionidae_Leptobasis_melinogaster | 2006-10-16 | Langshied, T. | United States | Santa Gertrudis Creek, King Ranch; 27°35.4'N, 98°2.6'W |  |
| GEODE17935_Gomphidae_Anormogomphus_kiritshenkoi | 1935-06-10 | Lazar, Y. | Iraq | Baghdad | Carl Cook Collection |
| GEODE7839_Coenagrionidae_Telagrion_longum | 1998-04-10 | Lencioni, A.A. | Brazil | Fazenda Santana do Rio Abaixo - Jacarei - SP; 23°14'52"S E, 46°59'58" W |  |
| GEODE7835_Coenagrionidae_Cyanallagma_angelae | 1999-03-14 | Lencioni, F.A.A. | Brazil | -23.5975S, -45.7280556W |  |
| GEODE7665_Lestidae_Indolestes_albicaudus | 1948-08-09 | Lieftinck, M.A. | Indonesia | NW New Guinea, Sorong |  |
| GEODE7667_Chlorocyphidae_Libellago_greeni | 1938-09-16 | Lieftinck, M.A. | Sri Lanka | Ceylon, C.P.,Haragama, 500m |  |
| GEODE7683_Euphaeidae_Anisopleura_lestoides | 1978-10-20 | Lieftinck, M.A. | India | NW India, UP, Kumaon, 1500m, Sat Tal |  |
| OD1298_Isostictidae_Cnemisticta_latilobata | 2015-09-11 | Marinov, M. | Solomon Islands | Rap-Guadalcanal, 2015 Valevahalo base camp; 9.6468° S 160.0447° E | 810 m a.s.l. |
| RMNH.INS.500853_Argiolestidae_Eoargiolestes_ochraceus | 2009-01-11 | Marinov, M. | New Caledonia | ; |  |
| RMNH.INS.500854_Argiolestidae_Trineuragrion_percostale | 2009-01-18 | Marinov, M. | New Caledonia | ; |  |
| RMNH.INS.505880_Libellulidae_Tapeinothemis_boharti | 2012-04-28 | Marinov, M. | Solomon Islands | Salapuka River in the Honiara Botanical Garden, Guadalcanal Island; -9.4363, 159.9417 |  |
| OD55_Isostictidae_Rhadinosticta_simplex | 2000-02-21 | Mathieson, M. | Australia | Queensland, Barakula State Forest 302; 26 20' S. 150 58' E. |  |
| OD69_Gomphidae_Ictinogomphus_australis | 2000-01-20 | Mathieson, M. | Australia | Queensland, Tuchekoi, Area near Gympie: Grassy edges and pools around Chinaman's creek; 26 24'43" S. 152 45'44" E. |  |
| OD1312_Isostictidae_Labidiosticta_vallisi | 2000-02-05 | Mathieson, M., J. Skevington & A. Skevington | Australia | Queensland, Brisbane, Forest Park; Scrub Creek; 27°25'05"S 152°50'13"E |  |
| OD1317_Platycnemididae_Nososticta_solida | 2000-01-03 | Mathieson, M., J. Skevington & A. Skevington | Australia | Queensland Slanthorpe Along River 28°39'54"S 151°56'26"E |  |
| OD1319_Libellulidae_Macrodiplax_cora | 2000-03-04 | Mathieson, M., J. Skevington & A. Skevington | Australia | Queensland Lake Clarendon 27°31'04"S 152°21'22"E |  |
| OD43_Lestoideidae_Diphlebia_coerulescens | 2000-02-05 | Mathieson, M., J. Skevington & A. Skevington | Australia | Queensland, Brisbane, Forest Park; Scrub Creek; 27 25'05" S, 152 50'13"E |  |
| OD85_Gomphidae_Austrogomphus_amphiclitus | 2000-02-05 | Mathieson, M., J. Skevington & A. Skevington | Australia | Brisbane, Forest Park: Scrub Creek, 27 25 05 S. 152 50'13" |  |
| GEODE7777_Libellulidae_Dythemis_nigra | 2020-05-20 | Mauffray, B. | Panama | Oeste Province; capira district; el carmen, CA, 4KM west off of via el Caco de Capira, small, intermittent stream |  |
| GEODE7833_Coenagrionidae_Calvertagrion_mauffrayi | 1995-06-12 | Mauffray, B. | Ecuador | borrow pit nr. Rio Savaleto, km marker 31; -0.47, -76.85 |  |
| GEODE7852_Coenagrionidae_Homeoura_lindneri | 2019-12-17 | Mauffray, B. | Paraguay | San Antonio,,Rio Parana,; -27.3913, -56.8367 |  |
| GEODE7857_Coenagrionidae_Platystigma_astictum | 1998-11-12 | Mauffray, B. | Bolivia |  |  |
| GEODE7761_Libellulidae_Tauriphila_argo | 2015-11-28 | Mézière, N. | French Guiana | Sinnamary; 5.1233611, -52.9553055 |  |
| GEODE7762_Libellulidae_Macrothemis_nobilis | 2016-06-24 | Mézière, N. | French Guiana | Sinnamary; 5.1233611, -52.9553055 |  |
| GEODE7763_Gomphidae_Archaeogomphus_nanus | 2016-07-03 | Mézière, N. | French Guiana | Sinnamary; 5.1233611, -52.9553055 |  |
| GEODE7764_Libellulidae_Miathyria_marcella | 2017-06-28 | Mézière, N. | French Guiana | Kourou; 5.148015, -52.635704 | 280617-1, male |
| GEODE7766_Libellulidae_Zenithoptera_fasciata | 2015-12-01 | Mézière, N. | French Guiana | Sinnamary; 5.1233611, -52.9553056 | 011215-1, male |
| GEODE7768_Libellulidae_Elasmothemis_williamsoni | 2015-11-28 | Mézière, N. | French Guiana | Sinnamary; 5.1233611, -52.9553055 |  |
| GEODE7770_Libellulidae_Gynothemis_pumilia | 2016-06-17 | Mézière, N. | French Guiana | Sinnamary; 5.1233611, -52.9553055 |  |
| GEODE7771_Lauromacromiidae_Lauromacromia_dubitalis | 2016-01-16 | Mézière, N. | French Guiana | Sinnamary; 5.1233611, -52.9553055 |  |
| GEODE7772_Gomphidae_Ebegomphus_minutus | 2016-01-26 | Mézière, N. | French Guiana | Sinnamary; 5.1233611, -52.9553055 |  |
| GEODE7773_Gomphidae_Zonophora_batesi | 2015-12-01 | Mézière, N. | French Guiana | Sinnamary; 5.1233611, -52.9553055 |  |
| GEODE7774_Gomphidae_Desmogomphus_tigrivensis | 2016-07-03 | Mézière, N. | French Guiana | Sinnamary; 5.1233611, -52.9553055 |  |
| GEODE7775_Gomphidae_Agriogomphus_sylvicola | 2015-12-01 | Mézière, N. | French Guiana | Sinnamary; 5.1233611, -52.9553055 |  |
| GEODE7776_Libellulidae_Micrathyria_didyma | 2015-12-05 | Mézière, N. | French Guiana | Kourou |  |
| GEODE7778_Coenagrionidae_Metaleptobasis_bicornis | 2016-10-24 | Mézière, N. | French Guiana | Iracoubo; 5.489229, -53.303506 |  |
| GEODE7779_Calopterygidae_Mnesarete_cupraea | 2016-07-16 | Mézière, N. | French Guiana | Nouragues; 4.03804, -52.67727 |  |
| GEODE7780_Calopterygidae_Hetaerina_caja | 2016-06-24 | Mézière, N. | French Guiana | Sinnamary; 5.1233611, -52.9553055 |  |
| GEODE7781_Coenagrionidae_Phasmoneura_exigua | 2016-02-09 | Mézière, N. | French Guiana | Sinnamary; 5.1233611, -52.9553055 |  |
| GEODE7782_Coenagrionidae_Tuberculobasis_yanomami | 2016-07-06 | Mézière, N. | French Guiana | Iracoubo; 5.489229, -53.303506 |  |
| GEODE7783_Coenagrionidae_Telebasis_demarara | 2016-07-06 | Mézière, N. | French Guiana | Iracoubo |  |
| GEODE7784_Coenagrionidae_Epipotoneura_nehalennia | 2016-07-14 | Mézière, N. | French Guiana | Nouragues; 4.083041, -52.679985 |  |
| GEODE7788_Dicteriadidae_Heliocharis_amazona | 2016-09-25 | Mézière, N. | French Guiana | Sinnamary; 5.1233611, -52.9553056 | G1-025, male |
| GEODE7818_Gomphidae_Aphylla_sp | 2018-10-17 | Mézière, N. | French Guiana | Sinnamary; 5,1233611, -52,95530556 |  |
| GEODE7819_Coenagrionidae_Aeolagrion_dorsale | 2015-06-26 | Mézière, N. | French Guiana | Sinnamary; 5,4176111, -53,04366667 |  |
| RMNH.INS.506145_Argiolestidae_Neurolestes_trinervis | 2012-02-09 | Mézière, N. | Gabon | Ogoué-Lolo, route Koulamoutou-Pana,; -1.30447, 12.50123 |  |
| RMNH.INS.506163_Chlorocyphidae_Africocypha_lacuselephantum | 2012-04-07 | Mézière, N. | Gabon | NGOUNIE, route Mouila-Mimongo,; -1.72887, 11.30718 |  |
| RMNH.INS.508794_Platycnemididae_Stenocnemis_pachystigma | 2013-03-24 | Mézière, N. | Gabon | NGOUNIE, route de Koulamoutou-Mimongo, MAVANGA; -1.41428, 14.14632 |  |
| RMNH.INS.508797_Libellulidae_Neodythemis_afra | 2013-04-03 | Mézière, N. | Gabon | Haut-Ogooué, route Moanda-Francevile, concession Rougier, 2ème Barière, 2ème pont, Moyabi; -1.70565, 13.25595 |  |
| RMNH.INS.508833_Chlorocyphidae_Stenocypha_gracilis | 2013-09-03 | Mézière, N. | Gabon | NGOUNIE, Mbigou, route Mbigou-Moukabou 14km après Mbigou; -1.812, 11.85424 |  |
| RMNH.INS.508844_Calopterygidae_Umma_longistigma | 2013-09-08 | Mézière, N. | Gabon | NGOUNIE, Parc National des monts Birougou, La Louetsie; -1.89434, 12.22619 |  |
| RMNH.INS.554426_Gomphidae_Mastigogomphus_chapini | 2012-09-29 | Mézière, N. | Gabon | Haut-Ogooué, le Clao-centre de loisir, devant l'hotel, OSSKAMA; -1.11259, 13.87533 |  |
| RMNH.INS.554510_Gomphidae_Cornigomphus_guineensis | 2012-11-18 | Mézière, N. | Gabon | Haut-Ogooué, piste à droite avant africa n°1 (direction franceville), Moyabi; -1.68773, 13.31128 |  |
| RMNH.INS.508746_Synlestidae_Nubiolestes_diotima | 2012-12-31 | Mézière, N. & J. Lekogooelle | Gabon | WOLEU-NTEM, route de Kougouleu_Medouneu (Monts de Cristal), ASSSOK; 0.62771, 10.30423 |  |
| GEODE7858_Euphaeidae_Cryptophaea_saukra | 1992-06-30 | Michalski, J. | Thailand | Pi Pau Pau waterfall |  |
| GEODE7864_Platycnemididae_Hylaeargia_magnifica | 1994-08-02 | Michalski, J. | New Guinea |  |  |
| OD1316_Aeshnidae_Gynacantha_gracilis | 2004-02-23 | Miller, K. B. | Costa Rica | Heredia near Puerto Viejo La Selva Biological Station 10°25'N 84°00'W |  |
| GEODE17789_Corduliidae_Procordulia_moroensis | 1995-03-25/30 | Mohagan, D. | Philippines | Mindanao island/Bukidnon prov. Mt. Katanglad, Impulatao 750-900m |  |
| GEODE7689_Chlorogomphidae_Chlorogomphus_spec | 1994-05-02/20 | Mohagan, N. | Philippines | Mindoro, Mount Halcon, 1000-1500m | RMNH.ins.1153162 |
| GEODE17911_Gomphidae_Cacoides_latro | 2017-08-23 | Morris & Wappes | French Guiana | Amazone Nature Lodge, Kaw Rd. #6; 4.550330, -52.170310 |  |
| GEODE7687_Euphaeidae_Cyclophaea_cyanifrons | 1991-06-05 | Muller, R.A. | Philippines | PHILIPPINES, Palawan Id, Iwahig, Balsahan |  |
| GEODE7711_Libellulidae_Celithemis_eponina | 2018-07-21 | Myrup, A.R. | United States | Lake Champlain, Shelburne Bay S of Burlington; 44.40038, -73.23533 |  |
| GEODE7719_Calopterygidae_Calopteryx_aequabilis | 2018-07-20 | Myrup, A.R. | United States | Poultney River, Cogman Road; 43.62525, -73.37101 |  |
| GEODE7730_Lestidae_Archilestes_grandis | 2019-09-14 | Myrup, A.R. | United States | Kanab Creek, near Hog Canyon; 37.07898 -112.53918 |  |
| GEODE7732_Gomphidae_Gomphurus_externus | 2015-07-14 | Myrup, A.R. | United States | Mona Reservoir below Dam |  |
| GEODE7735_Libellulidae_Ladona_julia | 2015-06-26 | Myrup, A.R. | United States | Ten Acre Pond; Scotia Range Road; west of State College; 40.80125 -77.94308 |  |
| GEODE7736_Gomphidae_Erpetogomphus_compositus | 2017-07-27 | Myrup, A.R. | United States | Trout Creek; Whitehorse Ranch Road; southeast of Fields (SR 205); 42.16182 -118.43738 |  |
| GEODE7738_Libellulidae_Brechmorhoga_mendax | 2010-09-12 | Myrup, A.R. | United States | North Creek; Kolob Terrace Road; 4.6 miles north of Virgin; 40.35661 -111.57479 |  |
| GEODE7740_Aeshnidae_Rhionaeschna_multicolor | 2017-07-21 | Myrup, A.R. | United States | Kolob Reservoir; north of Virgin; 37.44433 -113.04946 |  |
| GEODE7746_Libellulidae_Paltothemis_lineatipes | 2017-07-07 | Myrup, A.R. | United States | Zion National Park; Kolob Canyon Area; Taylor Creek; 37.463097 -113.198280 |  |
| GEODE7747_Corduliidae_Cordulia_shurtleffii | 2018-07-13 | Myrup, A.R. | United States | Uinta Mountains; pond near Teapot Lake (SR 150); 40.68161 -110.94016 |  |
| GEODE7749_Coenagrionidae_Amphiagrion_abbreviatum | 2018-06-09 | Myrup, A.R. | United States | Kanosh Canyon near mouth (FR106); Cork Creek; 38.77154 -112.39673 |  |
| GEODE7754_Gomphidae_Stenogomphurus_rogersi | 2015-06-26 | Myrup, A.R. | United States | Rothrock State Forest; Beaver Dam Meadows off Pine Swamp Road; south of State College (off SR 26) ; 40.71184 -77.88047 |  |
| OD1321_Lestidae_Lestes_disjunctus | 2015-07-21 | Myrup, A.R. | United States | Utah, Wastach County, Jordanelle Wetlands below dam (SR 32), N of Heber; N40.58161° W111.42781° |  |
| GEODE7708_Libellulidae_Plathemis_subornata | 2015-06-09 | Myrup, A.R. & M. Clark | United States | UT Hwy 196, 10 miles S of I-80; N40.61401, W112.70823 |  |
| GEODE17923_Gomphidae_Merogomphus_longistigma | 1981-05-01 | Nathan, T.R.S. | India | Mysore State, Agumbe Ghat, Shimoga District | Elevation 2000 ft |
| GEODE2711_Coenagrionidae_Luzonobasis_glauca | 1997-04-20 | Nazareno, C.M. | Philippines | Luzon, Apayao Prov. Flora, Santa Maria, Lumon River area |  |
| GEODE7678_Argiolestidae_Luzonargiolestes_baltazarae | 1997-05-30 | Nazareno, C.M. | Philippines | Luzon, Cagayan prov, Claveria, Brgy Union, 100-500m, |  |
| GEODE17894_Gomphomacromiidae_Gomphomacromia_nodisticta | 1974-01-27 | Neff, J.L. | Argentina | Catamarca Seep near Minas Capillitas |  |
| GEODE7697_Coenagrionidae_Protoneura_tenuis | 2003-12-06 | Ogden et al | Peru | Tambopata N.R. Explorer's Inn Tower River Junction |  |
| GEODE7739_Libellulidae_Perithemis_tenera | 2003-12-14 | Ogden, T.H. | Peru | Puerto Maldonado; 12.401901S, 69.14020 |  |
| GEODE7794_Coenagrionidae_Microstigma_anomalum | 2003-12-06 | Ogden, T.H. | Peru | Tambopata N.R. Explorer's Inn Tower River Junction |  |
| GEODE7868_Platycnemididae_Rhyacocnemis_prothoracica | 2004-06-05 | Oppel, S. | Papua New Guinea | S 06.72° E 145.08° |  |
| GEODE17604_Austropetaliidae_Austropetalia_patricia | 1998-12-10 | Paulson, D.R. | Australia | Bridal veil falls area, 2-3km E Blackhealth, 2800-3000'. New South Wales |  |
| GEODE17609_Aeshnidae_Epiaeschna_heros | 2005-04-22 | Paulson, D.R. | Florida | Liberty County, Sweetwater Creek at Hwy 270 |  |
| GEODE17940_Petaluridae_Petalura_litorea | 1998-12-20 | Paulson, D.R. | Australia | South Queensland, North Stradbroke Island Tortoise Lagoon | Ware et al. 2014 |
| GEODE7832_Coenagrionidae_Caliagrion_billinghursti | 1998-12-12 | Paulson, D.R. | Australia | Kangaroo Creek, S. of Grays Point |  |
| GEODE7880_Coenagrionidae_Denticulobasis_dunklei | 1988-12-26 | Paulson, D.R. | Peru | near Shimigay Lake, varzea forest |  |
| GEODE17788_Corduliidae_Procordulia_jacksoniensis | 1998-12-09 | Paulson, D.R. & N. Smith | Australia | New South Wales Boggy Creek, tributaries and marshy ponds associated with creek on Boggy Creek Road, 9km N Jenolan caves 3600m |  |
| GEODE17884_Pseudocorduliidae_Pseudocordulia_elliptica | 1998-12-25 | Paulson, D.R. & N. Smith | Australia | Queensland streams on Mount Lewis, NW Julatten 3000ft |  |
| GEODE7701_Libellulidae_Agrionoptera_insignis | 1998-12-24 | Paulson, D.R. & N. Smith | Australia | Queensland, Swamp 2.1 rd km N Daintree River Crossing on cape tribulation rd. |  |
| GEODE17915_Gomphidae_Peruviogomphus_pearsoni | 1971-08-25 | Pearson, D.L. | Ecuador | Province Napo, Limoncocha | Lake edge |
| GEODE17758_Libellulidae_Atoconeura_kenya | 1949-06 | Pinhey, E. | Kenya | Kenya, Nyeri |  |
| RU123_Lauromacromiidae_Lauromacromia_bedei | 2001-09 | Pinto, A. | Brazil | MG, P. E. Rio Preto |  |
| RU126_Lauromacromiidae_Lauromacromia_picinguaba | 2004-12 | Pinto, A. | Brazil | PR, Pontal do Paraná |  |
| RU139_Idomacromiidae_Neocordulia_volxemi | 1998-11-01/15 | Pinto, A. | Brazil | GO, Mineiros |  |
| GEODE7826_Coenagrionidae_Aceratobasis_macilenta | 29495 | Plaumann, F. | Brazil |  |  |
| RMNH.INS.229080_Gomphidae_Macrogomphus_quadratus | 2008-03-02 | Ragai, R. | Malaysia | Sarawak, Bintulu Division, SPFP, Samarakan, Sg Philip, Samarakan Conservation Area (disturbed and secondary forest); |  |
| RMNH.INS.506492_Platystictidae_Sinosticta_ogatai | 2012-05-31 | Reels, G.T. | China | Hong Kong, Hong Kong - Ng Tung Chai; |  |
| OD148_Platycnemididae_Platycnemis_pennipes |  | Rehn A.C. | Europe |  | male |
| OD151_Devadattidae_Devadatta_podolestoides |  | Rehn A.C. | Thailand |  | male |
| OD157_Lestoideidae_Lestoidea_conjuncta |  | Rehn A.C. | Australia |  | male |
| OD159_Isostictidae_Isosticta_robustior |  | Rehn A.C. | New Caledonia |  |  |
| OD162_Argiolestidae_Caledargiolestes_uniseries |  | Rehn A.C. | New Caledonia |  | male |
| OD1125_Calopterygidae_Neurobasis_ianthinipennis |  | Rehn, A. | Papua New Guinea |  |  |
| GEODE17943_Petaluridae_Uropetala_chiltoni | 1986-01-24 | Reichel, J. | New Zealand | South Island, Canterbury, Lewis Pass, 1000m | Ware et al. 2014 |
| GEODE7765_Corduliidae_Paracordulia_sericea | 2016-11-01 | Renoult, J. | French Guiana | SA (5) Crique Roche; 3.62672, -53.22751 |  |
| GEODE7787_Platystictidae_Palaemnema_brevignoni | 2016-07-15 | Renoult, J. | French Guiana | Nouragues |  |
| RMNH.5011406_Platycnemididae_Macrocnemis_gracilis | 2013-02-11 | Richards, S.J. | Papua New Guinea | Bilbilokabip; |  |
| RMNH.5011412_Libellulidae_Microtrigonia_curvata | 2013-02-08 | Richards, S.J. | Papua New Guinea | Yukfon Creek; |  |
| RMNH.5011413_Libellulidae_Bironides_glochidion | 2012-06-29 | Richards, S.J. | Papua New Guinea | Purari Camp 9; |  |
| RMNH.5011576_Aeshnidae_Austrogynacantha_heterogena | 2012-04-95 | Richards, S.J. | Australia | Western Australia, Kununurra Airport; |  |
| RMNH.5011578_Synthemistidae_Metaphya_tillyardi | 2011-03-14 | Richards, S.J. | Papua New Guinea | near Bat Cave; |  |
| RMNH.5011582_Isostictidae_Tanymecosticta_leptalea | 2011-04-13 | Richards, S.J. | Papua New Guinea | Tapos Creek; |  |
| RMNH.INS.501979_Coenagrionidae_Thaumatagrion_funereum | 2010 | Richards, S.J. | Papua New Guinea |  |  |
| RMNH.INS.501990_Argiolestidae_Pyrrhargiolestes_aulicus | 2009 | Richards, S.J. | Papua New Guinea |  |  |
| RMNH.INS.501995_Platycnemididae_Cyanocnemis_aureofrons | 2009 | Richards, S.J. | Papua New Guinea | Mine Expedition, secret locality; |  |
| RMNH.INS.543752_Argiolestidae_Wahnesia_kirbyi |  | Richards, S.J. | Papua New Guinea |  |  |
| GEODE7820_Argiolestidae_Archiargiolestes_pusillissimus | 1999-11-28 | S. Dunkle | Australia | brushy swamp W of Denmark, and E of the Kent River |  |
| ANCH188_Polythoridae_Miocora_peraltica | 2020-06-02 | Sanchez Herrera, M. | Colombia | Anchicayá-PNNFarrallones-"La Riqueza"; 3.6094167, -76.8845 |  |
| BOY03_Polythoridae_Cora_xanthostoma | 2006-09-10 | Sanchez Herrera, M. | Colombia | Cundinamarca, Guayabetal, Chirajara. 4.208611; -73.800556 |  |
| PP330_Polythoridae_Polythore_procera |  | Sanchez Herrera, M. | Ecuador | Imbabura |  |
| TM207_Libellulidae_Brechmorhoga_vivax | 2016-08-17 | Sandoval, J. & C. Bota | Colombia | Parque Nacional Natural Tatamá, Vereda Monte Bello; 5.22855; -76.09826669 |  |
| RMNH.INS.505846_Platycnemididae_Arabicnemis_caerulea | 2005-05-31 | Schneider, W. | Yemen | Wadi Dau'an System, Wadi Mara; 15.143, 48.449 |  |
| RMNH.INS.229282_Platycnemididae_Paracnemis_alluaudi | 2011 | Schuette, K. | Madagascar |  |  |
| RMNH.INS.500093_Pentaphlebiidae_Pentaphlebia_stahli | 2008-05-24 | Schuette, K. | Cameroon | Southwest Province, 13 km NW of Loum, Mt Kupe, Nyasoso; 4.8306, 9.6794 |  |
| RMNH.INS.500162_Platycnemididae_Allocnemis_contraria | 2008-06-13 | Schuette, K. | Cameroon | South Province, 26 km E of Campo, Campo-Ma'an National Park (buffer zone), Bitandé River 1km E of Nkoélon; 2.394, 10.054 |  |
| GEODE12749_Libellulidae_Celebothemis_delecollei | 1983-10-11 | Scipio S Parmiono | Sulawesi | Sulawesi, Biseang Labboro |  |
| GEODE17707_Corduliidae_Epitheca_semiaquea | 2016-04-02 | Shiley, S. | United States | Louisiana Natchitoches Par., Kishatchie National Forest, Anderson Pond, |  |
| GEODE7756_Libellulidae_Pachydiplax_longipennis | 2015-06-13 | Shuler, B. & A.R. Myrup | United States | Salem; Salem Pond; north end; across UT Hwy 198; 40.05423 -111.67869 |  |
| GEODE17713_Corduliidae_Helocordulia_uhleri | 2003-06-24 | Sibley, F.C. | United States | New York Fulton Town of Johnstown, Peck Lake Outlet 1350ft |  |
| GEODE17725_Libellulidae_Orthemis_macrostigma | 2016-02-19 | Sibley, F.C. | United States | Virgin Islands: Saint Croix, Southwest sub-district, just Northwest of Subway, small pond; 17 42.569N 64 50.368W | 1F. 130'. IORI #: 00082440. Lot #: 000073. EX Fred C Sibley Collection. Florida State Collection of Arthropods. Proc by: Bill Mauffray 4/4/2019 |
| GEODE17904_Gomphidae_Arigomphus_furcifer | 2000-06-10 | Sibley, F.C. | United States | New York, Orange County, Sterling Forest Lake; lat: 41 10 30N long: 74 16 30W | 9:30-12AM. clear, 80's. One of two along edge of small pond. Yale tag 6422. EX International Odonata Research Institute. Florida State Collection of Arthropods. Processed by: Bill Mauffray 2008-12-26. IORI #000043422 |
| GEODE7823_Calopterygidae_Bryoplathanon_globifer | 2015-02-20 | Sibley, F.C. | Brazil | 6.5 miles NW of Camacan. Overflow seep from water tank near end of tower road; -15.384267S, -39.572183W |  |
| GEODE7828_Coenagrionidae_Anisagrion_allopterum | 2009-03-26 | Sibley, F.C. | Costa Rica | University of Georgia field station, 3 miles SE of Santa Elaena; 10.287833N, -84.7905W |  |
| GEODE7743_Petaluridae_Tanypteryx_hageni | 2003-06-21 | Skevington, J. | United States | Butté Co., Butter Creek near Cherry Hill Campground; 40.0556N, 121.2947W | Ware et al. 2014 |
| GEODE7792_Synthemistidae_Eusynthemis_nigra | 2000-01-01 | Skevington, J. | Australia |  | male |
| OD63_Lestidae_Austrolestes_annulosus | 1999-12-17 | Skevington, J. | Australia | WA, Stirling, Range National Park Talyuberlup Peak Hilltop; 34 24' S. 117 57' E. |  |
| GEODE7791_Austrocorduliidae_Hesperocordulia_berthoudi | 1999-12-21 | Skevington, J. & A. | Australia | Near Wellington Dam; 33.2404S, 115.5723E |  |
| OD1318_Petaluridae_Petalura_gigantea | 2000-01-23 | Skevington, J. & A. | Australia | New South Wales Washpool NationalPark Wetlan 1.3 km west of campground , entrance road 29°29'57"S 152°19'09"E |  |
| OD77_Libellulidae_Rhyothemis_graphiptera | 2000-01-16 | Skevington, J. & A. | Australia | Queensland, Bribie Island |  |
| OD81_Synlestidae_Synlestes_weyersii | 2000-01-22 | Skevington, J. & A. | Australia | New South Wales, Gibralter Range National Park, Suveyors creek; 29 32'17" S. 152 18'38" E. |  |
| OD82_Libellulidae_Brachydiplax_denticauda | 2000-01-16 | Skevington, J. & A. | Australia | Queensland, Bribie Island |  |
| GEODE7728_Lestidae_Austrolestes_leda | 2000-02-03 | Skevington, J. & A. & M. Mathieson | Australia | Along River; -28.665 151.940556 |  |
| GEODE7737_Synthemistidae_Choristhemis_flavoterminata | 2000-02-03 | Skevington, J. & A. & M. Mathieson | Australia | Stanthorpe, Along River; 28.3954S, 151.5626E |  |
| GEODE7744_Austrocorduliidae_Micromidia_convergens | 2000-02-05 | Skevington, J. & A. & M. Mathieson | Australia | Brisbane Forest Park; Scrub Creek; 27.2505S,152.5013E |  |
| GEODE7745_Gomphidae_Armagomphus_armiger | 1999-12-21 | Skevington, J. & A. & M. Mathieson | Australia | Honeymoon Pool Near Wellington Dam; 33.2246S 115.5613E |  |
| GEODE7755_Synlestidae_Episynlestes_albicauda | 2000-03-26 | Skevington, J. & A. & M. Mathieson | Australia | Undercliffe Falls; -28.508889 152.172222 |  |
| GEODE7790_Gomphidae_Antipodogomphus_acolythus | 2000-03-26 | Skevington, J. & A. & M. Mathieson | Australia | Clarence River; 28.3807S 152.1700 E |  |
| GEODE7716_Argiolestidae_Griseargiolestes_albescens | 2000-01-29 | Skevington, J. & D. Reeves | Australia | Stradbroke Island Capembah Creek; S27.28, E153.25 |  |
| OD83_Corduliidae_Hemicordulia_australiae | 2000-01-26 | Skevington, J. & D. Reeves | Australia | Queensland, Stradbroke Island |  |
| OD1310_Austrocorduliidae_Cordulephya_pygmaea | 2000-04-02 | Skevington, J. & M. Mathieson | Australia | Queensland, Brisbane, Forest Park; Northbrook Creek; 21°18'S 152°41'E |  |
| OD1311_Synthemistidae_Parasynthemis_regina | 2000-02-27 | Skevington, J. & M. Mathieson | Australia | Queensland, Brisbane, Karawtha Forest |  |
| RMNH.INS.509787_Coenagrionidae_Coenagriocnemis_rufipes | 2010-11-09 | Skinner, A. | Mauritius |  |  |
| RMNH.INS.509795_Libellulidae_Thalassothemis_marchali | 2010-11-16 | Skinner, A. | Mauritius |  |  |
| RMNH.INS.509800_Coenagrionidae_Coenagriocnemis_insularis | 2010-12-20 | Skinner, A. | Mauritius |  |  |
| OD1119_Philosinidae_Rhinagrion_sp |  | SMB | Vietnam |  |  |
| OD1120_Perilestidae_Perissolestes_remotus |  | SMB | Costa Rica |  |  |
| OD1121_Rhipidolestidae_Agriomorpha_fusca |  | SMB | Vietnam |  |  |
| OD1122_Platycnemididae_Prodasineura_croconota |  | SMB | Vietnam |  |  |
| OD1123_Platycnemididae_Coeliccia_pyriformis |  | SMB | Vietnam |  |  |
| OD1124_Philogeniidae_Philogenia_carrillica |  | SMB | Costa Rica |  |  |
| OD1127_Lestidae_Orolestes_octomaculatus |  | SMB | Vietnam |  |  |
| OD1169_Gomphomacromiidae_Gomphomacromia_paradoxa | 2015-11-29 | SMB | Argentina |  |  |
| OD1170_Calopterygidae_Hetaerina_americana |  | SMB | United States | Utah, Washington county, Leeds, Leeds creek |  |
| GEODE7769_Libellulidae_Argyrothemis_argentea | 1986-10-05 | Spangler, P. & O.S. Flint | Brazil | Para, Rio Xingu Camp, CA 60Km, S of Altamira (52.22W, 3.39.S), 1st jungle stream, trail 1, malaise trap |  |
| GEODE7803_Libellulidae_Diastatops_obscura | 1913-04 | Steinbach, J. | Bolivia | Santa Cruz, Sara province | male |
| GEODE7822_Argiolestidae_Nesolestes_radama | 1990-03-06 | Steiner, W.E. | Madagascar | 7 km W Ranomafana |  |
| GEODE7638_Libellulidae_Nannophlebia_axiagasta | 1970-06-27 | Straatman, R. | Indonesia | Joma, Mambare River, 300 m | RMNH.ins.1153178 |
| GEODE17779_Corduliidae_Navicordulia_errans | 1980-01-21 | Strickman, L. | Paraguay | Itapua San Benito/Pastoreo, flaoting in lake |  |
| GEODE17614_Aeshnidae_Oreaeschna_dictatrix | 1935-08-31 | Stuber, W. | New Guinea | Cycloop | 1000 m |
| GEODE7643_Synthemistidae_Palaeosynthemis_gracilenta | 1931-06 | Stüber, W. | Indonesia | NN Guinea 19 Hollandia Humboldt Baai | RMNH.ins.1153203 |
| GEODE7680_Synthemistidae_Palaeosynthemis_cervula | 1937-03-06 | Stüber, W. | Indonesia | Hollandia, Humboldt Bay, Bewani Hills, Uskwar, 500 m | RMNH.ins.1153205 |
| GEODE7690_Aeshnidae_Plattycantha_cornuta | 1932-09 | Stüber, W. | Indonesia | Hollandia, Humboldt Bay, Cycloop mountains, 1000m | RMNH.ins.1153206 |
| OD126_Calopterygidae_Vestalis_apicalis | 2003-05-30 | Svenson, G. | India | Karnataha, UDUPL- Somershkear, Evergreen Forest |  |
| RMNH.INS.503604_Coenagrionidae_Archibasis_viola |  | Tateh, O. | Malaysia | Sarawak, Bintulu, Tubau, 'new' stream area, swampy section by stream; 3.1277833333, 113.66448333 | O. Tateh |
| RMNH.INS.502077_Argiolestidae_Miniargiolestes_minimus | 2010-01-12 | Taylor, J. | Australia | Western Australia, Gin Gin,; |  |
| RMNH.INS.502084_Argiolestidae_Archiargiolestes_parvulus | 2010-01-12 | Taylor, J. | Australia | Western Australia, Gin Gin,; |  |
| GEODE17712_Corduliidae_Helocordulia_selysii | 2003-05-01 | Tennessen, K.J. | United States | Mississippi Tishomingo Cripple Deer Creek, W of Allsboro, AL nr. Limestone quarry |  |
| GEODE17783_Corduliidae_Neurocordulia_yamaskanensis | 2009-07-07 | Tennessen, K.J. | United States | Wisconsin Wood Wisconsin River, boat launch off Biron Drive |  |
| GEODE17793_Corduliidae_Neurocordulia_michaeli | 2005-07-10 | Tennessen, K.J. | Canada | Ontario Renfrew Petawala River, Hwy. 17, Petawawa |  |
| GEODE17936_Gomphidae_Phanogomphus_exilis | 1993-04-18 | Tennessen, K.J. | United States | Alabama, Clarke County. Choctaw Creek | Hwy. 84, Gosport |
| GEODE7830_Coenagrionidae_Apanisagrion_lais | 2013-05-23 | Tennessen, K.J. | United States | Aravaipa Creek; N32.8986, W110.42084 |  |
| GEODE7848_Coenagrionidae_Oxyagrion_hermosae | 2015-11-25 | Tennessen, K.J. | Peru | Junin, Canaan Road to Tina de Perdra,, small stream; 11.46938, -74.55766 |  |
| OD976_Calopterygidae_Ormenophlebia_imperatrix | 2005-09-21 | Tennessen, K.J. | Ecuador | Pastaza Province, Triutary of Rio Anzu, north of Mera; 1 25'5"S 78 3'9"W | 1190 m elevation |
| GEODE7844_Coenagrionidae_Mesamphiagrion_demarmelsi | 2014-10-14 | Tennessen, K.J. & W. Mauffray | Colombia | Cundinamarca: Sesquilé SE on road to Laguna de Guatavita, , small vegetated farm pond; 04.9934°W, -73.77805°W |  |
| PD166_Petaluridae_Tanypteryx_pryeri | 2008 | Theischinger, G. | Japan |  | Ware et al. 2014 |
| RMNH.5011411_Libellulidae_Notolibellula_bicolor | 2014-02-01 | Theischinger, G. | Australia | Queensland, tributary Isabella Ck, nr. Isabella Falls; |  |
| RMNH.5011415_Gomphidae_Austroepigomphus_praeruptus |  | Theischinger, G. | Australia | New South Wales,; |  |
| RMNH.INS.504965_Aeshnidae_Acanthaeschna_victoria |  | Theischinger, G. | Australia | ; |  |
| GEODE17902_Synthemistidae_Tonyosynthemis_ofarrelli | 1986-01-11 | Theischinger, G. & L. Müller | Australia | Queensland Booloumba Creek |  |
| RMNH.INS.500862_Argiolestidae_Podopteryx_selysi | 1997-04-06 | Theischinger, G. & L. Müller | Australia | Queensland, Palmerston N.P., Along Palmerston Hwy,; |  |
| OD103_Coenagrionidae_Neoneura_mariana |  | Trapero, A. | Cuba |  | male |
| OD105_Coenagrionidae_Protoneura_capillaris |  | Trapero, A. | Cuba |  | male |
| GEODE12748_Libellulidae_Celebophlebia_dactylogastra | 1991-05-02 | Van Tol, J. | Indonesia | Sulawesi, c. 15 km NNE Malili, c. 150 m asl., 2 32'S 121 12'E |  |
| GEODE7675_Chlorocyphidae_Disparocypha_biedermanni | 1993-10-24 | Van Tol, J. | Indonesia | Indonesia, C Sulawesi, 40 km N of Woutu: just N of Batas. Small stream nr bridge road Wotu-Tentena at km 4. Distrirbed primary forest; w=2-4m, d=01=0.3, shade. Boulders. 1200m; 2 13'30''S 120 46'30''E |  |
| GEODE7668_Platycnemididae_Calicnemia_pulverulans | 1985-08-04 | Vick, G. | Nepal | Tarang Marang |  |
| GEODE7882_Coenagrionidae_Andinagrion_peterseni | 2006-12-29 | Von Ellenrieder, N. | Argentina | Cachi, rio Calchaqui; 25 6'0" S, 66 10'60"W |  |
| OD1281_Megapodagrionidae_Teinopodagrion_meridionale | 2012-01-28 | Von Ellenrieder, N. & R.W. Garrison | Argentina | Salta, Lesser, Arroyo de piedras sin nombre, along marshy/rocky runnel; 24°40'56"S 65°28'33"W | 1426 m elevation |
| OD102_Calopterygidae_Matrona_basilaris | 2003-05-12 | Wang, L.J. | Taiwan | Taping | male |
| OD96_Rhipidolestidae_Rhipidolestes_aculeatus | 2003-05-12 | Wang, L.J. | Taiwan | Taipei Co., Wanli, Taping |  |
| OD99_Euphaeidae_Bayadera_brevicauda | 2003-05-12 | Wang, L.J. | Taiwan | Taipei Co., Wanli, Taping |  |
| GEODE7612_Libellulidae_Cannaphila_insularis |  | Ware, J. | Guyana |  |  |
| OD1095_Libellulidae_Sympetrum_corruptum |  | Ware, J. | United States | New Jersey |  |
| OD1158_Aeschnosomatidae_Pentathemis_sp |  | Ware, JL | Australia |  |  |
| OD1164_Cordulegastridae_Cordulegaster_sp |  | Ware, JL | United States |  |  |
| OD107_Libellulidae_Pantala_flavescens | 2003-08-20 | Warrick, G. & R. Larsen | United States | New Mexico, Chaves Co., Pecos River east of the scout camp Bitter lake National Wildlife Refuge | male |
| GEODE7656_Platycnemididae_Nososticta_selysii | 1957-03-27 | Wegner, AMR | Indonesia | Annanese, Kupang |  |
| GEODE7760_Libellulidae_Austrothemis_nigrescens | 1988-02-11 | Westfall, M.J. & M.J. Beech | Australia | SE Queensland, North Stradbroke Island |  |
| GEODE7860_Isostictidae_Austrosticta_soror | 1997-01-25 | Whitesmith, G. | Australia |  |  |
| OD35_Chlorocyphidae_Rhinocypha_tincta | 2001-07-05 | Whiting | Papua New Guinea | Chimbu Province, Haia village, Crater Mountain Research area; S06 42.35' E145 016 |  |
| OD136_Coenagrionidae_Papuagrion_occipitale | 2000-09-21 | Whiting, A.S. & M.F. | Papua New Guinea | Sandaun Province, Menakmofib Village; S 04 49.786' E 141 38.126' | male |
| OD120_Chlorocyphidae_Heliocypha_biseriata | 2003-08-16 | Whiting, Svenson, Bybee | Malaysia | Sabah, Crocker Range N.P., Inabong substation turnoff @ Penam Pang River |  |
| OD134_Chlorocyphidae_Libellago_semiopaca | 2003-08-16 | Whiting, Svenson, Bybee | Malaysia | Sabah, Crocher Range N. P., Inabong substation turnoff @ Penepang River |  |
| GEODE17864_Idionychidae_Idionyx_claudia | 2013-07-30 | Zhang, H. | China | Zhejiang Fuvecun, Jingning co. Lishui City |  |
| GEODE17876_Macromidiidae_Macromidia_kelloggi | 2013-07-30 | Zhang, H. | China | Zhejiang Fuyecun, Lishui, Jingning County |  |
| GEODE17903_Gomphidae_Anisogomphus_anderi | 2009-08-13 | Zhang, H. | China | Qinshuigu in Nanling National reserve, Ruyuan County, Shaoguan City, N. Guangdong Province; 24° 55'N 113° 01'E | Zhang Haomiao world odonata collection. 750 m |
| GEODE17913_Gomphidae_Fukienogomphus_promineus | 2010-05-15 | Zhang, H. | China | Yadongxi, Conghua City, Guangdong Province, China; 23°41'-23°42' N 113°43'-113°49' E | 1 Male. 500 m. Zhang Haomiao World Odonata Collection |
| GEODE17914_Gomphidae_Shaogomphus_postocularis | 2006-06-19 | Zhang, H. | China | Mudanjiang City, Heillongjiang Province, Lowland Marsh Ponds | Zhang Haomiao World Odonata Collection |
| GEODE17919_Gomphidae_Gastrogomphus_abdominalis | 2003-04-15 | Zhang, H. | China | Anhui Province. Nanyanhu, Hefei City |  |
| GEODE17922_Gomphidae_Melligomphus_ardens | 2009-05-15 | Zhang, H. | China | Yadongxi, Conghua City, Guangdong Province; 23 41' N, 113 43' E |  |
| GEODE17933_Gomphidae_Scalmogomphus_falcatus | 2013-06-17 | Zhang, H. | China | Yunnan Province, Yangbi County, Dali, Yangbijiang River | Ex Haomiao Zhang's Odonata Collection. Carl Cook Collection. Florida State Collection of Arthropods |
| GEODE7871_Platystictidae_Sinosticta_sylvatica | 2009-04-27 | Zhang, H. | China |  |  |
| GEODE7872_Priscagrionidae_Sinocnemis_henanensis | 2012-07-20 | Zhang, H. | China | Shennonjia National Reserve |  |
| OD1079_Philogangidae_Philoganga_vetusta | 2014-06-20 | Zhang, H. | China | Hainan, Wuzhishan, Shuiman, stream; 109.661628, 18.850304 | 730 m elevation |
| RMNH.INS.502064_Rhipidolestidae_Rhipidolestes_truncatidens | 2009-05-21 | Zhang, H. | China | N. Guadong, Nanling Reserve,; |  |
| RMNH.INS.508447_Libellulidae_Palpopleura_sexmaculata | 2011-10-07 | Zhang, H. | China | Guangdong, Conghua city, Weidong; |  |
| RMNH.INS.508515_Libellulidae_Nannophyopsis_clara | 2010-05-04 | Zhang, H. | China | Guangdong, Huizhou City, Ruhu; |  |
| RMNH.INS.508528_Chlorogomphidae_Watanabeopetalia_usignata | 2011-07-22 | Zhang, H. | China | Guizhou, Guiyang City, Xiangzhigou; |  |
| RMNH.INS.508607_Calopterygidae_Mnais_mneme | 2011-05-18 | Zhang, H. | China | Guangdong, Conghua City, Foot of Nankunshan, Conghua village; |  |
| RMNH.INS.508616_Calopterygidae_Atrocalopteryx_melli | 2011-08-08 | Zhang, H. | China | Guangdong, Shaoguan City, Shixing County, Chebaling Reserve; |  |
| RMNH.INS.508632_Synlestidae_Megalestes_heros | 2009-05-21 | Zhang, H. | China | Guangdong, Shaoguan City, Ruyuan, Nanling Reserve; |  |
| RMNH.INS.508637_Platycnemididae_Indocnemis_orang | 2009-09-17 | Zhang, H. | China | Guangdong, Shaoguan City, Ruyuan, Nanling Reserve; |  |
| RMNH.INS.508661_Gomphidae_Lamelligomphus_camelus | 2009-07-27 | Zhang, H. | China | Guangdong, Huizhou City, Nankunshan; |  |
| RMNH.INS.508662_Gomphidae_Melligomphus_sinicus | 2011-06-20 | Zhang, H. | China | Guangdong, Huizhou City, Nankunshan; |  |
| RMNH.INS.508663_Gomphidae_Nihonogomphus_semanticus | 2011-05-27 | Zhang, H. | China | Guangdong, Huizhou City, Nankunshan; |  |
| RMNH.INS.508672_Gomphidae_Nepogomphus_walli | 2009-09-01 | Zhang, H. | China | Yunnan, Xishbuangbanna, Mengla, Buguzhai; |  |
| RMNH.INS.508674_Calopterygidae_Archineura_incarnata | 2011-05-12 | Zhang, H. | China | Guangdong, Huizhou City, Nankunshan; |  |
| RMNH.INS.508691_Gomphidae_Asiagomphus_hainanensis | 2011-5-20 | Zhang, H. | China | Guangdong, Conghua City, Yingcun, foot of Nankunshan; |  |
| RMNH.INS.508700_Gomphidae_Labrogomphus_torvus | 2011-08-07 | Zhang, H. | China | Guangdong, Conghua City, Yadongxi; |  |
| RMNH.INS.509134_Gomphidae_Euthygomphus_koxingai | 2009-04-18 | Zhang, H. | China | Hainan, Xinglong County, Nanling; |  |
| RMNH.INS.509144_Gomphidae_Dubitogomphus_bicornutus | 2006-08-27 | Zhang, H. | China | Beijing, Beijing, Yunmenshan; |  |
| RMNH.INS.509145_Aeshnidae_Cephalaeschna_obversa | 2011-08-02 | Zhang, H. | China | Guizhou, Guiyang City, Xiangzhigou; |  |
| RMNH.INS.509146_Gomphidae_Sinictinogomphus_clavatus | 2011-08-03 | Zhang, H. | China | Guizhou, Guiyang City, Qianlingshan; |  |
| GEODE17704_Corduliidae_Epitheca_marginata | 2004-04-15 | Zhaohui, L. | China | Jiangsu Mt. Yousi, Goochin |  |
| GEODE7634_Gomphidae_Sinogomphus_telamon | 1943-07-25 | Zu, C.S. | China | Chung-an hsien Hsien-fen-Lin, Fukien (Fujian?) |  |
| GEODE12753_Gomphidae_Lindenia_tetraphylla | 1987-07-15 |  | Greece | Thessaloniki, Lake Volvi, 37m |  |
| GEODE12755_Gomphidae_Perissogomphus_stevensi | 1986-06-02 |  | Nepal | Upper Mai Valley, Pualthola, 3300ft |  |
| GEODE17755_Libellulidae_Anatya_januaria | 1981-03-21 |  | Brazil | Sao Paulo; San Carlos | 1m |
| GEODE17909_Gomphidae_Zonophora_diversa | 1999-01-30 |  | Argentina | Misiones: Iguazo Department, Iguazo | 1F. IORI #: 00086255. Lot #: 000075. Ex Carl Cook Collection. Florida State Collection of Arthropods. Proc by: Bill Mauffray 5/5/2020 |
| GEODE17929_Libellulidae_Scapanea_frontalis | 1982-04-23 |  | Jamaica | Blue Mountain |  |
| GEODE2756_Euphaeidae_Heterophaea_barbata | 1997-04-02 |  | Philippines |  |  |
| GEODE7650_Libellulidae_Diplacina_spec | 1953-09-14 |  | Indonesia | Laiwui-Obi | RMNH.ins.1153160 |
| GEODE7653_Platycnemididae_Esme_mudiensis | 1963-05 |  | India | Madras State, Anamalai Hills, Kadamparai, 3500 feet |  |
| GEODE7655_Libellulidae_Diplacina_persephone | 1936-02-12/19 |  | Indonesia | North New Guinea, Hollandia, Humboldt Bay, Nonno mountains, 400 m | RMNH.ins.1153180 |
| GEODE7659_Libellulidae_Microtrigonia_gomphoides | 1931 |  | Indonesia | North New Guinea, Hollandia, Humboldt Bay, Cycloop mountains, 400 m | RMNH.ins.1153175 |
| GEODE7660_Platycnemididae_Nososticta_exul | 1951-09-12 |  | Indonesia | Isl. Halmahera, Akilamo 50-100m |  |
| GEODE7666_Chlorocyphidae_Heliocypha_bisignata | 1961-04 |  | India | Nilgiri Hills, Devala, 3200 ft, PSM |  |
| GEODE7672_Chlorocyphidae_Cyrano_unicolor | 1936-06-21 |  | Phillipines | Mount Makiling |  |
| GEODE7673_Platycnemididae_Calicnemia_erythromelas | 1998-04-21 |  | Thailand | Chiang Mai, Doi Inthanon, streams at checkpoint 2 trail, 1500m |  |
| GEODE7676_Libellulidae_Misagria_calverti | 1966-03-04 |  | Suriname | Compagnie Kreek | RMNH.ins.517922 |
| GEODE7686_Euphaeidae_Euphaea_cardinalis | 1963-05 |  | India | SOUTH INDIA, Madras state, Anamalai hills, Kadamparai, 3500 ft, P.S.N. |  |
| GEODE7694_Euphaeidae_Euphaea_refulgens | 1990-08-05 |  | Philippines | Philippines, Luzon Id, Laguna Prov., Pagasanjan Falls |  |
| GEODE7759_Libellulidae_Nannophya_australis |  |  | Australia | Stradbroke Island |  |
| GEODE7815_Libellulidae_Urothemis_assignata | 2010-03-13 |  | Gabon | 8 Km from Cap Esterias to Libreville; 0.586042, 9.335804 |  |
| GEODE7829_Coenagrionidae_Antiagrion_grinbergsi | 1988-01-01 |  | Chille |  |  |
| GEODE7841_Coenagrionidae_Peristicta_aeneoviridis | 2015-02-19 |  | Brazil | Rio Panela, 2.3 miles W of Camacan and east of large pond; 15 25.039S, 39 31.992W |  |
| OD1077_Mesopodagrionidae_Mesopodagrion_sp | 2015-05-31 |  | China | Yunnan, Lu Chun, parque do totem touro | 1884 m elevation |
| OD1091_Libellulidae_Brachythemis_contaminata |  |  | Vietnam |  |  |
| OD1094_Libellulidae_Nannophlebia_alexia |  |  | Indonesia |  |  |
| OD1101_Macromiidae_Epophthalmia_elegans |  |  | China | Hong Kong |  |
| OD1106_Libellulidae_Zygonyx_flavicosta |  |  | Senegal |  |  |
| OD1116_Coenagrionidae_Cyanallagma_sp |  |  | Argentina |  |  |
| OD1280_Priscagrionidae_Priscagrion_kiautai | 2016-05-27 |  | Vietnam | Tam Dao; 21.465 N 105.632 E |  |
| RMNH.INS.505717_Polythoridae_Chalcopteryx_rutilans | 2010 |  | Peru |  |  |
| SRR921569_Ephemeroptera_Baetis_sp | 2011-05-17 |  | Germany | North Rhine-Westphalia, Rhein-Sieg-Kreis, Donrath, Agger |  |
| SRR921591_Ephemeroptera_Ephemera_danica | 2011-05-17 |  | Germany | North Rhine-Westphalia, Rhein-Sieg-Kreis, Donrath, Agger |  |
| SRR921596_Ephemeroptera_Eurylophella_sp | 2012-02-19 |  | United States | Pennsylvania, Cumberland County, King's Gap State Park |  |
| SRR921608_Ephemeroptera_Isonychia_bicolor | 2011-10-31 |  | United States | New Jersey, Hunterton County, Muscanetcong River and North Mountain Road |  |
