# Supplementary material for: Soaring Systematics: An Evaluation of Biogeography and Flight Behavior in Dragonflies and Damselflies (Insecta: Odonata) Using Phylogenomics
Source: Syst Biol. 2026 Jan 22;75(5):984–1002. doi: 10.1093/sysbio/syag005 (PMC13445619; doi:10.1093/sysbio/syag005)
Supplement: Supplementary file 1 [file syag005_supplemental_files.zip › Supplemental Table 2 - Fossil Calibrations.docx]

| **Tree Node** | **Fossil species** | **Fossil Age/ Prior lower bound** | **Prior upper bound** | **Accession number** | **Publication** | **Phylogenetic justification** | **Age justification for the bed/fossil** |
| --- | --- | --- | --- | --- | --- | --- | --- |
| Paleoptera (Root) | *Erasipteroides valentini* | 319.9 | 412 | see Kohli et al. (2021) | see Kohli et al. (2021) | see Kohli et al. (2021) | see Kohli et al. (2021) |
| Odonata | *Triassolestodes asiaticus* | 228.4 | 319.9 | see Kohli et al. (2021) | see Kohli et al. (2021) | see Kohli et al. (2021) | see Kohli et al. (2021) |
| Epiprocta | *Liassophlebia sp.* | 199.3 | 319.9 | see Kohli et al. (2021) | see Kohli et al. (2021) | see Kohli et al. (2021) | see Kohli et al. (2021) |
| Anisoptera | *Sinacymatophlebia mongolica* | 158.1 | 319.9 | see Kohli et al. (2021) | see Kohli et al. (2021) | see Kohli et al. (2021) | see Kohli et al. (2021) |
| Zygoptera | *Mersituria ludmilae* | 71.9 | 319.9 | see Kohli et al. (2021) | see Kohli et al. (2021) | see Kohli et al. (2021) | see Kohli et al. (2021) |
| Aeshnidae | *Gomphaeschna inferna* | 71.9 | 319.9 | see Kohli et al. (2021) | see Kohli et al. (2021) | see Kohli et al. (2021) | see Kohli et al. (2021) |
| Anax | *Anax cryptus* | 5.6 | 319.9 | Monte Castellaro working Collection of Gentilini No. 1219 | Gentilini and Peters 1993, The Upper Miocene aeshnids of Monte Castellaro, | from the Getilini and Peters (1)anterior part of Arc shorter than its posterior segment ,(2)RP diverging near the midst of the anterior part of Arc, (3) MA-fork and the conjunction of its anterior branch with RP3-4 very distinct, (4)Mspl and Rspl deeply arched, (5)between IR2 and Rspl some distinct slanting rows of rarely less than 5cells,  (6) IR2 unforked (its pseudo fork starting beneath Pt embraces only 2 rows of cells),(7) RP distinctly curved towards Pt(Fig.4a),(8)A2 in hind wing running parallel to A3 | roveri et al (2008) - Recent advancements in the Messinian stratigraphy of Italy and their Mediterranean-scale implications Fossils are from the Gessoso-solfifera Formation |
| Gomphidae | *Proterogomphus renateae* | 150 | 319.9 | see Kohli et al. (2021) | see Kohli et al. (2021) | see Kohli et al. (2021) | see Kohli et al. (2021) |
| Lindeniinae sensu our tree | *Cratolindenia knuepfae* | 122.5 | 319.9 | SMNS 64414, an exoskeleton | G. Bechly. 2000. Two new fossil dragonfly species (Insecta: Odonata: Anisoptera: Araripegomphidae and Lindeniidae) from the Crato Limestone (Lower Cretaceous, Brazil). Stuttgarter Beiträge zur Naturkunde Serie B (Geologie und Paläontologie) 296:1-16 |  | lacustrine - large; lithified carbonate, Crato Formation of Brazil |
| Erpetogomphus + Nihonogomphus | *Erpetogomphus shii* | 23 | 319.9 | RX001, an exoskeleton | D. R. Zheng, A. Nel, E. A. Jarzembowski, S. C. Chang, H. C. Zhang and B. Wang. 2018. Exceptionally well-preserved dragonflies (Insecta: Odonata) in Mexican amber. Alcheringa 43:157-164 |  | Mexican/Chiapas amber |
| Petaluridae | *Argentinopetala* | 125 | 319.9 | MLP 12887, a wing | J. F. Petrulevičius and A. Nel. 2003. Oldest Petalurid dragonfly (Insecta: Odonata): a Lower Cretaceous specimen from south Patagonia, Argentina. Cretaceous Research 24:31-34 |  | Aptial lacustrine |
| Cavilabiata | *Juralibellula ningchengensis* | 158.1 | 319.9 | see Kohli et al. (2021) | see Kohli et al. (2021) | see Kohli et al. (2021) | see Kohli et al. (2021) |
| Chlorogomphidae + Cordulegasteridae | *Araripechlorogomphus muratai* | 112.6 | 319.9 | see Kohli et al. (2021) | see Kohli et al. (2021) | see Kohli et al. (2021) | see Kohli et al. (2021) |
| Libellulidae | *Tauriphila? cerestensis* | 29.2 | 319.9 | see Kohli et al. (2021) | see Kohli et al. (2021) | see Kohli et al. (2021) | see Kohli et al. (2021) |
| Libellulinae sensu our tree | Libellula requieniana | 28.1 | 319.9 | Fossil ID Number: 49440 | H. Nicolas. 1890. Insectes fossiles d'Aix - collection du Muséum Requien, à Avignon. Compte Rendus, Association Française pour l'Avancement des Sciences 18:424-432 |  | Chattian lacustrine - large limestone in the Niveau du gypse d'Aix Formation of France. |
| Sympetrum + Celithemis | Sympetrum bigoti n. sp | 28.1 | 319.9 | MNHN-LP-R.07452 | A. Nel and M. Papazian. 1985. Description d'une nouvelle espèce d'odonate fossile du Stampien d'Aix-en-Provence (Odon., Libellulidae). Entomologica Gallica 1:351-355 |  | Chattian lacustrine, 'Aix Formation of France. |
| Cordullidae | *Croatocordulia platyptera* | 12.7 | 319.9 | see Kohli et al. (2021) | see Kohli et al. (2021) | see Kohli et al. (2021) | see Kohli et al. (2021) |
| Libellulidae + Cordullidae | *Palaeolibellula zherikhini* | 89.5 | 319.9 | see Kohli et al. (2021) | see Kohli et al. (2021) | see Kohli et al. (2021) | see Kohli et al. (2021) |
| Macromiidae | *Epophtalmia biordinata* | 15.5 | 319.9 | see Kohli et al. (2021) | see Kohli et al. (2021) | see Kohli et al. (2021) | see Kohli et al. (2021) |
| Ephemeroptera - Baetis sp. | *Australiphemera revelata* | 112.6 | 319.9 | see Kohli et al. (2021) | see Kohli et al. (2021) | see Kohli et al. (2021) | see Kohli et al. (2021) |
| Calopterygidae + Chlorocyphidae | *Sinocalopteryx shangyongensis* | 47.8 | 319.9 | see Kohli et al. (2021) | see Kohli et al. (2021) | see Kohli et al. (2021) | see Kohli et al. (2021) |
| Calopterygid Complex | *Sapho' sannoisiensis* | 23 | 319.9 | see Kohli et al. (2021) | see Kohli et al. (2021) | see Kohli et al. (2021) | see Kohli et al. (2021) |
