# Supplementary material for: Soaring Systematics: An Evaluation of Biogeography and Flight Behavior in Dragonflies and Damselflies (Insecta: Odonata) Using Phylogenomics
Source: Syst Biol. 2026 Jan 22;75(5):984–1002. doi: 10.1093/sysbio/syag005 (PMC13445619; doi:10.1093/sysbio/syag005)
Supplement: Supplementary file 1 [file syag005_supplemental_files.zip › Supplemental Table 3 - Aspect Ratio Averages, Flight Behavior Designation, AHE Loci Recovery.docx]

| **Sample ID** | **Aspect Ratio, L/W (Hindwing)** | **Aspect Ratio, L/W (Forewing)** | **Flight Behavior** | **AHE Loci Recovered** |
| --- | --- | --- | --- | --- |
| **Outgroups** | | | | |
| SRR1695233_Ephemeroptera_Ecdyonurus_insignis | ? | ? | Flyer | 837 |
| SRR921569_Ephemeroptera_Baetis_sp. | ? | ? | Flyer | 831 |
| SRR921591_Ephemeroptera_Ephemera_danica | ? | ? | Flyer | 784 |
| SRR921596_Ephemeroptera_Eurylophella_sp. | ? | ? | Flyer | 840 |
| SRR921608_Ephemeroptera_Isonychia_bicolor | ? | ? | Flyer | 821 |
| **Aeschnosomatidae** | | | | |
| GEODE7682_Aeschnosomatidae_Aeschnosoma_forcipula | 3.06862745 | 4.02469136 | Flyer | 464 |
| OD1158_Aeschnosomatidae_Pentathemis_sp. | 3.00760456 | 3.87614679 | Flyer | 920 |
| **Aeshnidae** | | | | |
| GEODE17607_Aeshnidae_Andaeschna_rufipes | 3 | 3.61157025 | Flyer | 906 |
| GEODE17609_Aeshnidae_Epiaeschna_heros | 3.28551856 | 4.27299312 | Flyer | 921 |
| GEODE17610_Aeshnidae_Gomphaeschna_furcillata | 3.09114895 | 4.08643831 | Flyer | 898 |
| GEODE17614_Aeshnidae_Oreaeschna_dictatrix | 3.3037037 | 3.96774194 | Flyer | 433 |
| GEODE7669_Aeshnidae_Amphiaeschna_ampla | 3.26811594 | 4.12820513 | Flyer | 241 |
| GEODE7690_Aeshnidae_Plattycantha_cornuta | 3.42105263 | 4.1875 | Flyer | 868 |
| GEODE7691_Aeshnidae_Heliaeschna_crassa | 3.15217391 | 3.90973872 | Flyer | 823 |
| GEODE7740_Aeshnidae_Rhionaeschna_multicolor | 3.02463576 | 4.10236387 | Flyer | 928 |
| OD1303_Aeshnidae_Gynacanthaeschna_sikkima | 2.76521739 | 4.08235294 | Flyer | 908 |
| OD1316_Aeshnidae_Gynacantha_gracilis | 3.13251303 | 3.98538244 | Flyer | 911 |
| OD830_Aeshnidae_Anax_walsinghami | 3.14263946 | 4.20123894 | Flyer | 912 |
| RMNH.5011576_Aeshnidae_Austrogynacantha_heterogena | 2.82727273 | 3.79069767 | Flyer | 922 |
| RMNH.INS.502232_Aeshnidae_Gynacantha_victoriae | 3.13251303 | 3.98538244 | Flyer | 925 |
| RMNH.INS.504908_Aeshnidae_Telephlebia_godeffroyi | 3.0647821 | 3.92034139 | Flyer | 912 |
| RMNH.INS.504941_Aeshnidae_Notoaeschna_sagittata | 3.32380952 | 4.22727273 | Flyer | 927 |
| RMNH.INS.504965_Aeshnidae_Acanthaeschna_victoria | 3.3167 | 4.17 | Flyer | 922 |
| RMNH.INS.505016_Aeshnidae_Anax_imperator | 3.14263946 | 4.20123894 | Flyer | 922 |
| RMNH.INS.505017_Aeshnidae_Isoaeshna_isoceles | 2.977011494 | 3.591549296 | Flyer | 927 |
| RMNH.INS.506912_Aeshnidae_Indaeschna_grubaueri | 3.58091067 | 4.60354665 | Flyer | 910 |
| RMNH.INS.507402_Aeshnidae_Brachytron_pratense | 3.27956989 | 4.16455696 | Flyer | 937 |
| RMNH.INS.507528_Aeshnidae_Boyeria_cretensis | 3.02385021 | 3.94327694 | Flyer | 916 |
| RMNH.INS.509145_Aeshnidae_Cephalaeschna_obversa | 3.19683482 | 4.09069212 | Flyer | 930 |
| TM170_Aeshnidae_Remartinia_luteipennis | 3.29172714 | 4.26585366 | Flyer | 904 |
| TM392_Aeshnidae_Rhionaeschna_joannisi | 3.02463576 | 4.10236387 | Flyer | 906 |
| TM581_Aeshnidae_Andaeschna_occidentalis | 3 | 3.61157025 | Flyer | 885 |
| **Amanipodagrionidae** | | | | |
| OD1133_Amanipodagrionidae_Amanipodagrion_gilliesi | 4.175 | 4.658536585 | Percher | 924 |
| **Amphipterygidae** | | | | |
| OD1148_Amphipterygidae_Amphipteryx_agrioides | 5.17318355 | 5.41741123 | Percher | 920 |
| GEODE7678_Argiolestidae_Luzonargiolestes_baltazarae | 3.71134021 | 4.30681818 | Percher | 913 |
| GEODE7716_Argiolestidae_Griseargiolestes_albescens | 5.58536585 | 5.02272727 | Percher | 930 |
| GEODE7820_Argiolestidae_Archiargiolestes_pusillissimus | 4.2631579 | 4.28947368 | Percher | 889 |
| GEODE7821_Argiolestidae_Eoargiolestes_ochraceus | 4.409836066 | 5.107142857 | Percher | 920 |
| GEODE7822_Argiolestidae_Nesolestes_radama | 4.538461538 | 5.294117647 | Percher | 832 |
| OD1083_Argiolestidae_Austroargiolestes_icteromelas | 4.56 | 5.10416667 | Percher | 919 |
| OD1309_Argiolestidae_Nesolestes_sp. | 4.538461538 | 5.294117647 | Percher | 919 |
| OD162_Argiolestidae_Caledargiolestes_uniseries | 4.28070175 | 4.72222222 | Percher | 923 |
| RMNH.5008334_Argiolestidae_Podolestes_orientalis | 4.8 | 4.71428571 | Percher | 921 |
| RMNH.INS.500397_Argiolestidae_Allolestes_maclachlanii | 4.78571429 | 4.80952381 | Percher | 937 |
| RMNH.INS.500853_Argiolestidae_Eoargiolestes_ochraceus | 4.409836066 | 5.107142857 | Percher | 929 |
| RMNH.INS.500854_Argiolestidae_Trineuragrion_percostale | 5.56410256 | 5.45454546 | Percher | 939 |
| RMNH.INS.500862_Argiolestidae_Podopteryx_selysi | 3.773809524 | 4.15 | Percher | 924 |
| RMNH.INS.501973_Argiolestidae_Argiolestes_muller | 4.49748744 | 4.5 | Percher | 894 |
| RMNH.INS.501990_Argiolestidae_Pyrrhargiolestes_aulicus | 4.783783784 | 5.057142857 | Percher | 925 |
| RMNH.INS.502075_Argiolestidae_Metagrion_ornatum | 4.594594595 | 4.882352941 | Percher | 913 |
| RMNH.INS.502077_Argiolestidae_Miniargiolestes_minimus | 3.697183099 | 4.344827586 | Percher | 921 |
| RMNH.INS.502084_Argiolestidae_Archiargiolestes_parvulus | 4.2631579 | 4.28947368 | Percher | 917 |
| RMNH.INS.503407_Argiolestidae_Caledopteryx_sarasini | 4.28070175 | 4.72222222 | Percher | 930 |
| RMNH.INS.506145_Argiolestidae_Neurolestes_trinervis | 4.2 | 4.796610169 | Percher | 929 |
| RMNH.INS.543752_Argiolestidae_Wahnesia_kirbyi | 4.369565217 | 4.511111111 | Percher | 926 |
| **Austrocorduliidae** | | | | |
| GEODE17873_Austrocorduliidae_Lathrocordulia_metallica | 3.03888889 | 3.84137931 | Flyer | 852 |
| GEODE7744_Austrocorduliidae_Micromidia_convergens | 2.88059702 | 3.62545455 | Flyer | 928 |
| GEODE7791_Austrocorduliidae_Hesperocordulia_berthoudi | 3.06713781 | 3.75431035 | Flyer | 933 |
| OD1172_Austrocorduliidae_Austrocordulia_refracta | 2.70097508 | 3.89970502 | Flyer | 918 |
| OD1310_Austrocorduliidae_Cordulephya_pygmaea | 3.38071066 | 3.98342541 | Flyer | 925 |
| GEODE17604_Austropetaliidae_Austropetalia_patricia | 3.06734694 | 4.15196078 | Flyer | 907 |
| OD1112_Austropetaliidae_Phyllopetalia_apollo | 3.07142857 | 4.34444444 | Flyer | 900 |
| OD1115_Austropetaliidae_Hypopetalia_pestilens | 3.41 | 4.453488372 | Flyer | 904 |
| **Calopterygidae** | | | | |
| GEODE7705_Calopterygidae_Noguchiphaea_yoshikoae | 3.58888889 | 4.13953488 | Percher | 937 |
| GEODE7719_Calopterygidae_Calopteryx_aequabilis | 3.09200971 | 3.18772535 | Percher | 937 |
| GEODE7721_Calopterygidae_Neurobasis_chinensis | 2.36216216 | 2.99481865 | Percher | 934 |
| GEODE7748_Calopterygidae_Mnesarete_williamsoni | 4.6712609 | 4.44223916 | Percher | 928 |
| GEODE7779_Calopterygidae_Mnesarete_cupraea | 4.6712609 | 4.44223916 | Percher | 931 |
| GEODE7780_Calopterygidae_Hetaerina_caja | 4.2421347 | 4.23470057 | Percher | 932 |
| GEODE7823_Calopterygidae_Bryoplathanon_globifer | 6.40384615 | 4.46052632 | Percher | 919 |
| OD102_Calopterygidae_Matrona_basilaris | 5 | 4.73913044 | Percher | 927 |
| OD1125_Calopterygidae_Neurobasis_ianthinipennis | 2.36216216 | 2.99481865 | Percher | 922 |
| OD1170_Calopterygidae_Hetaerina_americana | 4.2421347 | 4.23470057 | Percher | 925 |
| OD126_Calopterygidae_Vestalis_apicalis | 3.76712329 | 3.32467533 | Percher | 919 |
| OD976_Calopterygidae_Ormenophlebia_imperatrix | 4.3255814 | 6.43137255 | Percher | 924 |
| RMNH.INS.501070_Calopterygidae_Echo_modesta | 4.75272 | 1.31859 | Percher | 932 |
| RMNH.INS.502054_Calopterygidae_Caliphaea_confusa | 4.13333333 | 5.19607843 | Percher | 929 |
| RMNH.INS.502132_Calopterygidae_Phaon_iridipennis | 3.14 | 3.60674157 | Percher | 908 |
| RMNH.INS.503116_Calopterygidae_Sapho_fumosa | 2.95192308 | 4.42666667 | Percher | 905 |
| RMNH.INS.505747_Calopterygidae_Vestalaria_smaragdina | ? | ? | Percher | 931 |
| RMNH.INS.505806_Calopterygidae_Psolodesmus_kuroiwae | 3.35643564 | 3.03738318 | Percher | 924 |
| RMNH.INS.507722_Calopterygidae_Matronoides_cyaneipennis | 2.5 | 2.740740741 | Percher | 936 |
| RMNH.INS.507799_Calopterygidae_Vestalis_amaryllis | 3.76712329 | 3.32467533 | Percher | 928 |
| RMNH.INS.508607_Calopterygidae_Mnais_mneme | 3.52380952 | 3.78313253 | Percher | 927 |
| RMNH.INS.508616_Calopterygidae_Atrocalopteryx_melli | 3.175 | 3.275 | Percher | 930 |
| RMNH.INS.508674_Calopterygidae_Archineura_incarnata | 3.12195122 | 3.583333333 | Percher | 926 |
| RMNH.INS.508844_Calopterygidae_Umma_longistigma | 3.34375 | 2.11392405 | Percher | 921 |
| **Chlorocyphidae** | | | | |
| GEODE7666_Chlorocyphidae_Heliocypha_bisignata | 5.35164835 | 5.47777778 | Percher | 707 |
| GEODE7667_Chlorocyphidae_Libellago_greeni | 5.08204604 | 5.40104104 | Percher | 241 |
| GEODE7672_Chlorocyphidae_Cyrano_unicolor | 5.13414634 | 6.10588235 | Percher | 885 |
| GEODE7675_Chlorocyphidae_Disparocypha_biedermanni | ? | 6 | Percher | 891 |
| GEODE7812_Chlorocyphidae_Heliocypha_perforata | 5.35164835 | 5.47777778 | Percher | 916 |
| OD120_Chlorocyphidae_Heliocypha_biseriata | 5.35164835 | 5.47777778 | Percher | 910 |
| OD134_Chlorocyphidae_Libellago_semiopaca | 5.08204604 | 5.40104104 | Percher | 913 |
| OD35_Chlorocyphidae_Rhinocypha_tincta | 4.72284237 | 5.18092519 | Percher | 898 |
| RMNH.INS.500989_Chlorocyphidae_Libellago_aurantiaca | 5.08204604 | 5.40104104 | Percher | 910 |
| RMNH.INS.501024_Chlorocyphidae_Sundacypha_petiolata | 5.51428571 | 5.58823529 | Percher | 917 |
| RMNH.INS.505345_Chlorocyphidae_Platycypha_caligata | 5.37837838 | 5.94594595 | Percher | 934 |
| RMNH.INS.505736_Chlorocyphidae_Aristocypha_fenestrella | 4.03311258 | 4.45683453 | Percher | 914 |
| RMNH.INS.506163_Chlorocyphidae_Africocypha_lacuselephantum | 4.79032258 | 5.27472528 | Percher | 918 |
| RMNH.INS.507707_Chlorocyphidae_Rhinoneura_villosipes | 5.941176471 | 5.977777778 | Percher | 918 |
| RMNH.INS.508833_Chlorocyphidae_Stenocypha_gracilis | 4.866666667 | 5.066666667 | Percher | 904 |
| GEODE7689_Chlorogomphidae_Chlorogomphus_spec | 3.21713442 | 3.93727915 | Flyer | 888 |
| OD1315_Chlorogomphidae_Chlorogomphus_auratus | 3.21713442 | 3.93727915 | Flyer | 916 |
| RMNH.INS.508528_Chlorogomphidae_Watanabeopetalia_usignata | 3.07377049 | 4.05699482 | Flyer | 926 |
| **Coenagrionidae** | | | | |
| GEODE2711_Coenagrionidae_Luzonobasis_glauca | 4.9 | 4.806451613 | Percher | 921 |
| GEODE2758_Coenagrionidae_Plagulibasis_ciliata | 5.018518519 | 5.185185185 | Percher | 882 |
| GEODE7697_Coenagrionidae_Protoneura_tenuis | 6.09858882 | 6.14693175 | Percher | 924 |
| GEODE7725_Coenagrionidae_Mecistogaster_linearis | 5.01721181 | 5.09584894 | Percher | 936 |
| GEODE7741_Coenagrionidae_Psaironeura_tenuissima | 5.8 | 6.32142857 | Percher | 934 |
| GEODE7749_Coenagrionidae_Amphiagrion_abbreviatum | 4.45709029 | 4.86942915 | Percher | 932 |
| GEODE7753_Coenagrionidae_Telebasis_salva | 4.51893154 | 4.77704847 | Percher | 926 |
| GEODE7758_Coenagrionidae_Acanthagrion_adustum | 5.00740769 | 5.200566 | Percher | 936 |
| GEODE7778_Coenagrionidae_Metaleptobasis_bicornis | 5.05128205 | 5.21008403 | Percher | 926 |
| GEODE7781_Coenagrionidae_Phasmoneura_exigua | 4.97142857 | 4.97368421 | Percher | 934 |
| GEODE7782_Coenagrionidae_Tuberculobasis_yanomami | 4.59499091 | 4.79134056 | Percher | 935 |
| GEODE7783_Coenagrionidae_Telebasis_demarara | 4.51893154 | 4.77704847 | Percher | 928 |
| GEODE7784_Coenagrionidae_Epipotoneura_nehalennia | 4.85714286 | 6.47826087 | Percher | 934 |
| GEODE7785_Coenagrionidae_Inpabasis_rosea | 4.65789474 | 4.72972973 | Percher | 932 |
| GEODE7786_Coenagrionidae_Acanthallagma_luteum | 3.097560976 | 3.536585366 | Percher | 933 |
| GEODE7794_Coenagrionidae_Microstigma_anomalum | 5.21875 | 3.85087719 | Percher | 928 |
| GEODE7819_Coenagrionidae_Aeolagrion_dorsale | 5.00740769 | 5.200566 | Percher | 939 |
| GEODE7826_Coenagrionidae_Aceratobasis_macilenta | 4.82352941 | 5.14285714 | Percher | 721 |
| GEODE7827_Coenagrionidae_Amphiallagma_parvum | 4.125 | 3.85185185 | Percher | 916 |
| GEODE7828_Coenagrionidae_Anisagrion_allopterum | 4.56816143 | 4.45938477 | Percher | 924 |
| GEODE7829_Coenagrionidae_Antiagrion_grinbergsi | 4.68 | 4.82 | Percher | 259 |
| GEODE7830_Coenagrionidae_Apanisagrion_lais | 4.1875 | 4.5625 | Percher | 930 |
| GEODE7832_Coenagrionidae_Caliagrion_billinghursti | 5.16 | 5.06 | Percher | 513 |
| GEODE7833_Coenagrionidae_Calvertagrion_mauffrayi | 4.27272727 | 4.26666667 | Percher | 898 |
| GEODE7835_Coenagrionidae_Cyanallagma_angelae | 4.853258949 | 4.970763881 | Percher | 926 |
| GEODE7836_Coenagrionidae_Diceratobasis_melanogaster | 3.26025 | 0.59409 | Percher | 770 |
| GEODE7839_Coenagrionidae_Telagrion_longum | 5.24242424 | 5.18181818 | Percher | 883 |
| GEODE7841_Coenagrionidae_Peristicta_aeneoviridis | 4.54545455 | 4.78787879 | Percher | 911 |
| GEODE7844_Coenagrionidae_Mesamphiagrion_demarmelsi | 4.76481578 | 4.94230104 | Percher | 929 |
| GEODE7848_Coenagrionidae_Oxyagrion_hermosae | 4.81818182 | 5.13333333 | Percher | 933 |
| GEODE7852_Coenagrionidae_Homeoura_lindneri | 4.11764706 | 4.41176471 | Percher | 933 |
| GEODE7854_Coenagrionidae_Leptobasis_melinogaster | 5.00437769 | 5.03012908 | Percher | 900 |
| GEODE7857_Coenagrionidae_Platystigma_astictum | 4.106870229 | 4.479674797 | Percher | 717 |
| GEODE7879_Coenagrionidae_Forcepsioneura_sancta | 6.625 | 5.75 | Percher | 900 |
| GEODE7880_Coenagrionidae_Denticulobasis_dunklei | 2.66616 | 0.59409 | Percher | 822 |
| GEODE7882_Coenagrionidae_Andinagrion_peterseni | 4.09375 | 4.54545455 | Percher | 908 |
| OD103_Coenagrionidae_Neoneura_mariana | 4.85860205 | 4.87945136 | Percher | 934 |
| OD105_Coenagrionidae_Protoneura_capillaris | 6.09858882 | 6.14693175 | Percher | 924 |
| OD1116_Coenagrionidae_Cyanallagma_sp. | 4.853258949 | 4.970763881 | Percher | 928 |
| OD136_Coenagrionidae_Papuagrion_occipitale | 4.71875 | 5.328125 | Percher | 928 |
| RMNH.5007797_Coenagrionidae_Azuragrion_nigridorsum | 3.87096774 | 4.22580645 | Percher | 936 |
| RMNH.5007810_Coenagrionidae_Africallagma_glaucum | 4.33333333 | 4.5 | Percher | 937 |
| RMNH.5010307_Coenagrionidae_Proischnura_polychromatica | 4.11111111 | 4.08823529 | Percher | 937 |
| RMNH.INS.229208_Coenagrionidae_Oreocnemis_phoenix | 5.3 | 6.34285714 | Percher | 928 |
| RMNH.INS.500398_Coenagrionidae_Leptocnemis_cyanops | 4.74 | 4.75 | Percher | 934 |
| RMNH.INS.501979_Coenagrionidae_Thaumatagrion_funereum | 3.465753425 | 4.084507042 | Percher | 932 |
| RMNH.INS.503604_Coenagrionidae_Archibasis_viola | 5.13953488 | 5.34117647 | Percher | 932 |
| RMNH.INS.503619_Coenagrionidae_Aciagrion_borneense | 4.5 | 4.85714286 | Percher | 932 |
| RMNH.INS.503951_Coenagrionidae_Argiocnemis_rubescens | 4.91891892 | 4.47222222 | Percher | 931 |
| RMNH.INS.504852_Coenagrionidae_Austrocoenagrion_lyelli | 4.957746479 | 4.753424658 | Percher | 930 |
| RMNH.INS.505254_Coenagrionidae_Austrocnemis_splendida | 4.2631579 | 4.75 | Percher | 929 |
| RMNH.INS.507703_Coenagrionidae_Stenagrion_dubium | 4.918367347 | 5.204081633 | Percher | 927 |
| RMNH.INS.509787_Coenagrionidae_Coenagriocnemis_rufipes | 5.86046512 | 4.97727273 | Percher | 937 |
| RMNH.INS.509800_Coenagrionidae_Coenagriocnemis_insularis | 5.86046512 | 4.97727273 | Percher | 935 |
| RMNH.INS.557636_Coenagrionidae_Pericnemis_stictica | 5.612155388 | 5.539057841 | Percher | 929 |
| TM305_Coenagrionidae_Acanthagrion_trilobatum | 5.00740769 | 5.200566 | Percher | 927 |
| TMCH16_Coenagrionidae_Protoneura_amatoria | 6.09858882 | 6.14693175 | Percher | 190 |
| **Cordulegastridae** | | | | |
| OD1164_Cordulegastridae_Cordulegaster_sp. | 3.47294783 | 4.53740914 | Flyer | 893 |
| OD1304_Cordulegastridae_Neallogaster_hermionae | 3.315789474 | 4.29787234 | Flyer | 902 |
| RMNH.INS.508536_Cordulegastridae_Anotogaster_sieboldii | 4.37799043 | 3.53629032 | Flyer | 916 |
| **Corduliidae** | | | | |
| GEODE17698_Corduliidae_Dorocordulia_lepida | 2.89611024 | 4.0037906 | Flyer | 933 |
| GEODE17699_Corduliidae_Dorocordulia_libera | 2.89611024 | 4.0037906 | Flyer | 930 |
| GEODE17703_Corduliidae_Epitheca_cynosura | 2.72884424 | 3.97652575 | Flyer | 932 |
| GEODE17704_Corduliidae_Epitheca_marginata | 2.72884424 | 3.97652575 | Flyer | 925 |
| GEODE17707_Corduliidae_Epitheca_semiaquea | 2.72884424 | 3.97652575 | Flyer | 929 |
| GEODE17711_Corduliidae_Guadalca_insularis | 2.6969697 | 3.90410959 | Flyer | 928 |
| GEODE17712_Corduliidae_Helocordulia_selysii | 3.191575 | 4.10196096 | Flyer | 925 |
| GEODE17713_Corduliidae_Helocordulia_uhleri | 3.191575 | 4.10196096 | Flyer | 922 |
| GEODE17717_Corduliidae_Hemicordulia_fidelis | 3.07543103 | 4.01891892 | Flyer | 929 |
| GEODE17720_Corduliidae_Hemicordulia_kalliste | 3.07543103 | 4.01891892 | Flyer | 929 |
| GEODE17779_Corduliidae_Navicordulia_errans | 3.148514851 | 4.012345679 | Flyer | 769 |
| GEODE17781_Corduliidae_Neurocordulia_virginiensis | 2.75956459 | 3.68368385 | Flyer | 874 |
| GEODE17783_Corduliidae_Neurocordulia_yamaskanensis | 2.75956459 | 3.68368385 | Flyer | 925 |
| GEODE17788_Corduliidae_Procordulia_jacksoniensis | 2.96994536 | 3.86006826 | Flyer | 907 |
| GEODE17789_Corduliidae_Procordulia_moroensis | 2.96994536 | 3.86006826 | Flyer | 918 |
| GEODE17793_Corduliidae_Neurocordulia_michaeli | 2.75956459 | 3.68368385 | Flyer | 920 |
| GEODE17832_Corduliidae_Somatochlora_cingulata | 3.10288889 | 4.09978786 | Flyer | 923 |
| GEODE7747_Corduliidae_Cordulia_shurtleffii | 2.92526281 | 3.92882638 | Flyer | 927 |
| GEODE7765_Corduliidae_Paracordulia_sericea | 2.81481482 | 3.83435583 | Flyer | 932 |
| OD1322_Corduliidae_Rialla_villosa | 3.10869565 | 4.08219178 | Flyer | 907 |
| OD146_Corduliidae_Cordulia_aenea | 2.92526281 | 3.92882638 | Flyer | 902 |
| OD83_Corduliidae_Hemicordulia_australiae | 3.07543103 | 4.01891892 | Flyer | 923 |
| RMNH.5009497_Corduliidae_Corduliochlora_borisi | 2.794871795 | 3.2 | Flyer | 920 |
| RMNH.5011578_Corduliidae_Metaphya_tillyardi | 3.158730159 | 3.99047619 | Flyer | 934 |
| RMNH.INS.500518_Corduliidae_Procordulia_leopoldi | 2.96994536 | 3.86006826 | Flyer | 933 |
| RMNH.INS.506657_Corduliidae_Heteronaias_heterodoxa | 2.95081967 | 4.11827957 | Flyer | 913 |
| **Devadattidae** | | | | |
| OD151_Devadattidae_Devadatta_podolestoides | 3.99924 | 1.02879 | Percher | 920 |
| RMNH.INS.501127_Devadattidae_Devadatta_argyoides | 3.99924 | 1.02879 | Percher | 934 |
| Dicteriadidae | | | | |
| GEODE7718_Dicteriadidae_Dicterias_atrosanguinea | 7.6797 | 0.21735 | Percher | 923 |
| GEODE7788_Dicteriadidae_Heliocharis_amazona | 4.58064516 | 4.88709677 | Percher | 927 |
| **Epiophlebiidae** | | | | |
| OD1076_Epiophlebiidae_Epiophlebia_superstes | 3.888 | 4.0078125 | Flyer | 912 |
| OD1302_Epiophlebiidae_Epiophlebia_laidlawi | 3.888 | 4.0078125 | Flyer | 907 |
| **Euphaeidae** | | | | |
| GEODE2756_Euphaeidae_Heterophaea_barbata | 5 | 4.982758621 | Percher | 872 |
| GEODE7683_Euphaeidae_Anisopleura_lestoides | 4.77586207 | 4.83076923 | Percher | 915 |
| GEODE7686_Euphaeidae_Euphaea_cardinalis | 3.37254902 | 3.65605096 | Percher | 872 |
| GEODE7687_Euphaeidae_Cyclophaea_cyanifrons | 4.618181818 | 4.872727273 | Percher | 918 |
| GEODE7694_Euphaeidae_Euphaea_refulgens | 3.37254902 | 3.65605096 | Percher | 931 |
| GEODE7858_Euphaeidae_Cryptophaea_saukra | 5.81034483 | 5.625 | Percher | 908 |
| OD132_Euphaeidae_Euphaea_ochracea | 3.37254902 | 3.65605096 | Percher | 902 |
| OD99_Euphaeidae_Bayadera_brevicauda | 4.62711864 | 5.74545455 | Percher | 903 |
| RMNH.5009494_Euphaeidae_Epallage_fatime | 3.98684211 | 3.7631579 | Percher | 930 |
| RMNH.INS.501089_Euphaeidae_Dysphaea_dimidiata | 5.02803 | 0.81144 | Percher | 938 |
| RMNH.INS.505757_Euphaeidae_Anisopleura_furcata | 4.77586207 | 4.83076923 | Percher | 917 |
| RMNH.INS.505817_Euphaeidae_Cryptophaea_vietnamensis | 5.75 | 5.79661017 | Percher | 926 |
| **Gomphidae** | | | | |
| GEODE12753_Gomphidae_Lindenia_tetraphylla | 2.99082569 | 4.3373494 | Percher | 691 |
| GEODE12754_Gomphidae_Mattigomphus_pinratani | 3.438596491 | 4.224489796 | Percher | 911 |
| GEODE12755_Gomphidae_Perissogomphus_stevensi | 3.309090909 | 4.131868132 | Percher | 766 |
| GEODE17903_Gomphidae_Anisogomphus_anderi | 3.07565012 | 4.29216868 | Percher | 919 |
| GEODE17904_Gomphidae_Arigomphus_furcifer | 3.26673081 | 4.18107748 | Percher | 931 |
| GEODE17909_Gomphidae_Zonophora_diversa | ?3.321428571 | 4.06122449 | Percher | 870 |
| GEODE17911_Gomphidae_Cacoides_latro | 3.66346154 | 4.3125 | Percher | 918 |
| GEODE17912_Gomphidae_Hagenius_brevistylus | 3.52124933 | 4.73995552 | Percher | 926 |
| GEODE17913_Gomphidae_Fukienogomphus_promineus | 3.32286996 | 4.42934783 | Percher | 917 |
| GEODE17914_Gomphidae_Shaogomphus_postocularis | 3 | 4.220930233 | Percher | 918 |
| GEODE17915_Gomphidae_Peruviogomphus_pearsoni | 3.34666667 | 4.36507937 | Percher | 779 |
| GEODE17917_Gomphidae_Octogomphus_specularis | 2.97762902 | 4.07693172 | Percher | 926 |
| GEODE17919_Gomphidae_Gastrogomphus_abdominalis | 3.20833333 | 3.94047619 | Percher | 917 |
| GEODE17922_Gomphidae_Melligomphus_ardens | 3.21546961 | 4.02884615 | Percher | 915 |
| GEODE17923_Gomphidae_Merogomphus_longistigma | 3.31468532 | 4.50638298 | Percher | 848 |
| GEODE17933_Gomphidae_Scalmogomphus_falcatus | 2.97777778 | 4.11347518 | Percher | 896 |
| GEODE17935_Gomphidae_Anormogomphus_kiritshenkoi | 2.5257732 | 3.48051948 | Percher | 462 |
| GEODE17936_Gomphidae_Phanogomphus_exilis | 3.04563132 | 4.00653972 | Percher | 918 |
| GEODE7634_Gomphidae_Sinogomphus_telamon | 2.978723404 | 4.052631579 | Percher | 206 |
| GEODE7681_Gomphidae_Phyllogomphoides_undulatus | 3.38519257 | 4.401727 | Percher | 617 |
| GEODE7692_Gomphidae_Phyllogomphoides_fuliginosus | 3.38519257 | 4.401727 | Percher | 742 |
| GEODE7710_Gomphidae_Ebegomphus_sp. | 3.4 | 3.655172414 | Percher | 931 |
| GEODE7731_Gomphidae_Ictinogomphus_pertinax | 3.3019802 | 4.31024096 | Percher | 923 |
| GEODE7732_Gomphidae_Gomphurus_externus | 3.10059141 | 4.18780785 | Percher | 932 |
| GEODE7733_Gomphidae_Tragogomphus_ellioti | ? | ? | Percher | 922 |
| GEODE7734_Gomphidae_Heliogomphus_scorpio | 3.29787234 | 4.25628141 | Percher | 917 |
| GEODE7736_Gomphidae_Erpetogomphus_compositus | 3.05787602 | 4.01137388 | Percher | 924 |
| GEODE7745_Gomphidae_Armagomphus_armiger | 3.10526316 | 4.25 | Percher | 932 |
| GEODE7754_Gomphidae_Stenogomphurus_rogersi | 3.52461526 | 4.35295149 | Percher | 930 |
| GEODE7763_Gomphidae_Archaeogomphus_nanus | 2.99539171 | 4.20108696 | Percher | 913 |
| GEODE7772_Gomphidae_Ebegomphus_minutus | 3.4 | 3.655172414 | Percher | 928 |
| GEODE7773_Gomphidae_Zonophora_batesi | 3.321428571 | 4.06122449 | Percher | 925 |
| GEODE7774_Gomphidae_Desmogomphus_tigrivensis | 3.01948052 | 4.216 | Percher | 927 |
| GEODE7775_Gomphidae_Agriogomphus_sylvicola | 2.99516908 | 3.99418605 | Percher | 919 |
| GEODE7790_Gomphidae_Antipodogomphus_acolythus | 3.0131579 | 4.08064516 | Percher | 938 |
| GEODE7818_Gomphidae_Aphylla_sp. | 3.41009693 | 4.34958561 | Percher | 920 |
| OD1314_Gomphidae_Ophiogomphus_morrisoni | 2.98475264 | 4.02416394 | Percher | 904 |
| OD69_Gomphidae_Ictinogomphus_australis | 3.3019802 | 4.31024096 | Percher | 601 |
| OD85_Gomphidae_Austrogomphus_amphiclitus | 3.06397306 | 4.08943089 | Percher | 772 |
| RMNH.5008090_Gomphidae_Ceratogomphus_pictus | 3.32170543 | 4.22685185 | Percher | 928 |
| RMNH.5009376_Gomphidae_Onychogomphus_styx | 3.05576208 | 4.17933492 | Percher | 924 |
| RMNH.5011399_Gomphidae_Stylurus_flavipes | 3.43879674 | 4.3333835 | Percher | 922 |
| RMNH.5011415_Gomphidae_Austroepigomphus_praeruptus | 2.96666667 | 3.95967742 | Percher | 933 |
| RMNH.5011429_Gomphidae_Hemigomphus_comitatus | 3.09698997 | 4.28333333 | Percher | 927 |
| RMNH.5011478_Gomphidae_Austrogomphus_prasinus | 3.223684211 | 4.174603175 | Percher | 923 |
| RMNH.INS.229080_Gomphidae_Macrogomphus_quadratus | 3.33628319 | 4.2924282 | Percher | 900 |
| RMNH.INS.501184_Gomphidae_Phaenandrogomphus_asthenes | 3.20289855 | 3.98412698 | Percher | 911 |
| RMNH.INS.501444_Gomphidae_Libyogomphus_christinae | 3.285714286 | 3.6 | Percher | 866 |
| RMNH.INS.501518_Gomphidae_Microgomphus_camerunensis | 3.12244898 | 4.46956522 | Percher | 907 |
| RMNH.INS.502394_Gomphidae_Lestinogomphus_congoensis | 3.07079646 | 3.67619048 | Percher | 931 |
| RMNH.INS.502555_Gomphidae_Gomphidia_gamblesi | 3.28697851 | 4.3250774 | Percher | 912 |
| RMNH.INS.502891_Gomphidae_Nepogomphoides_stuhlmanni | 2.77777778 | 3.57377049 | Percher | 912 |
| RMNH.INS.503108_Gomphidae_Diastatomma_gamblesi | 2.95575221 | 3.98333333 | Percher | 871 |
| RMNH.INS.503523_Gomphidae_Sieboldius_japponicus | 3.802325581 | 4.694444444 | Percher | 927 |
| RMNH.INS.503524_Gomphidae_Acrogomphus_jubilaris | 2.98019802 | 4.282051282 | Percher | 917 |
| RMNH.INS.504961_Gomphidae_Austrogomphus_ochraceus | 3.06397306 | 4.08943089 | Percher | 931 |
| RMNH.INS.505397_Gomphidae_Neurogomphus_cocytius | 3.33333333 | 4.71621622 | Percher | 922 |
| RMNH.INS.505653_Gomphidae_Notogomphus_praetorius | 3.23876405 | 4.3183391 | Percher | 937 |
| RMNH.INS.505754_Gomphidae_Amphigomphus_somnuki | 3.21052632 | 4.23566879 | Percher | 924 |
| RMNH.INS.506264_Gomphidae_Leptogomphus_coomansi | 3.15189873 | 4.38977636 | Percher | 926 |
| RMNH.INS.506367_Gomphidae_Megalogomphus_borneensis | 3.3628692 | 4.46842105 | Percher | 764 |
| RMNH.INS.507519_Gomphidae_Ophiogomphus_cecilia | 2.98475264 | 4.02416394 | Percher | 916 |
| RMNH.INS.507929_Gomphidae_Phyllogomphus_annulus | 3.081632653 | 4.051282051 | Percher | 924 |
| RMNH.INS.508012_Gomphidae_Paragomphus_genei | 3.25177305 | 3.62641509 | Percher | 909 |
| RMNH.INS.508545_Gomphidae_Trigomphus_beatus | 3.168674699 | 4.507462687 | Percher | 910 |
| RMNH.INS.508624_Gomphidae_Fukienogomphus_promineus | 3.32286996 | 4.42934783 | Percher | 925 |
| RMNH.INS.508661_Gomphidae_Lamelligomphus_camelus | 3.30625 | 4.08363636 | Percher | 913 |
| RMNH.INS.508662_Gomphidae_Melligomphus_sinicus | 3.30625 | 4.08363636 | Percher | 914 |
| RMNH.INS.508663_Gomphidae_Nihonogomphus_semanticus | 3.06145251 | 4.06122449 | Percher | 923 |
| RMNH.INS.508672_Gomphidae_Nepogomphus_walli | 2.83823529 | 4.05769231 | Percher | 902 |
| RMNH.INS.508691_Gomphidae_Asiagomphus_hainanensis | 3.45812808 | 4.03252033 | Percher | 922 |
| RMNH.INS.508700_Gomphidae_Labrogomphus_torvus | 3.36296296 | 4.41284404 | Percher | 926 |
| RMNH.INS.509134_Gomphidae_Euthygomphus_koxingai | 3.026548673 | 4.042553191 | Percher | 920 |
| RMNH.INS.509144_Gomphidae_Dubitogomphus_bicornutus | 2.64444444 | 3.9916318 | Percher | 928 |
| RMNH.INS.509146_Gomphidae_Sinictinogomphus_clavatus | 3.524752475 | 4.728395062 | Percher | 913 |
| RMNH.INS.509207_Gomphidae_Burmagomphus_divaricatus | 2.83850932 | 4.02419355 | Percher | 409 |
| RMNH.INS.509920_Gomphidae_Lamelligomphus_castor | 3.30625 | 4.08363636 | Percher | 920 |
| RMNH.INS.509971_Gomphidae_Orientogomphus_minor | 2.9625 | 4.81818182 | Percher | 923 |
| RMNH.INS.554426_Gomphidae_Mastigogomphus_chapini | ? | ? | Percher | 909 |
| RMNH.INS.554510_Gomphidae_Cornigomphus_guineensis | 3.297297297 | 4.194444444 | Percher | 896 |
| RMNH.INS.559802_Gomphidae_Crenigomphus_hartmanni | 3.01520913 | 4.03255814 | Percher | 908 |
| GEODE17894_Gomphomacromiidae_Gomphomacromia_nodisticta | 2.91988131 | 3.86891386 | Flyer | 669 |
| OD1169_Gomphomacromiidae_Gomphomacromia_paradoxa | 2.91988131 | 3.86891386 | Flyer | 907 |
| **Hemiphlebiidae** | | | | |
| OD1074_Hemiphlebiidae_Hemiphlebia_mirabilis | 1.43451 | 0.34776 | Percher | 877 |
| **Heteragrionidae** | | | | |
| GEODE7742_Heteragrionidae_Oxystigma_petiolatum | 6.24324324 | 5.24444444 | Percher | 927 |
| OD1126_Heteragrionidae_Dimeragrion_percubitale | 5.150943396 | 6.08 | Percher | 927 |
| TM155_Heteragrionidae_Heteragrion_tatama | 5.60174836 | 5.46935167 | Percher | 914 |
| TM404_Heteragrionidae_Heteragrion_erythrogastrum | 5.60174836 | 5.46935167 | Percher | 906 |
| **Hypolestidae** | | | | |
| OD1146_Hypolestidae_Hypolestes_sp | 6.525 | 4.96363636 | Percher | 923 |
| **Idionychidae** | | | | |
| GEODE17864_Idionychidae_Idionyx_claudia | 2.8 | 3.90429043 | Flyer | 927 |
| GEODE17870_Idionychidae_Idionyx_optatus | 2.8 | 3.90429043 | Flyer | 895 |
| RMNH.INS.506617_Idionychidae_Idionyx_philippa | 2.8 | 3.90429043 | Flyer | 918 |
| **Idomacromiidae** | | | | |
| GEODE17881_Idomacromiidae_Neocordulia_griphus | 2.855421687 | 3.833333333 | Flyer | 895 |
| NESOMASC_Idomacromiidae_Nesocordulia_mascarenica | 2.540816327 | 3 | Flyer | 687 |
| RMNH.5008153_Idomacromiidae_Syncordulia_gracilis | 2.90909091 | 3.8 | Flyer | 927 |
| RMNH.INS.503184_Idomacromiidae_Idomacromia_proavita | 2.93700787 | 3.70476191 | Flyer | 927 |
| RMNH.INS.507576_Idomacromiidae_Oxygastra_curtisii | 2.8995098 | 3.89776358 | Percher | 912 |
| RU139_Idomacromiidae_Neocordulia_volxemi | 2.855421687 | 3.833333333 | Flyer | 278 |
| TM41_Idomacromiidae_Neocordulia_batesi | 2.855421687 | 3.833333333 | Flyer | 916 |
| **Isostictidae** | | | | |
| GEODE7860_Isostictidae_Austrosticta_soror | 7.55 | 6 | Percher | 829 |
| OD1277_Isostictidae_Neosticta_fraseri | 5.68115942 | 5.26666667 | Percher | 920 |
| OD1298_Isostictidae_Cnemisticta_latilobata | 5.84782609 | 5.85416667 | Percher | 924 |
| OD1312_Isostictidae_Labidiosticta_vallisi | 4.89361702 | 5.61702128 | Percher | 917 |
| OD159_Isostictidae_Isosticta_robustior | 5.825 | 5.75609756 | Percher | 924 |
| OD55_Isostictidae_Rhadinosticta_simplex | 5.21621622 | 5.28571429 | Percher | 913 |
| RMNH.5011475_Isostictidae_Oristicta_filicicola | 5.64102564 | 5.67901235 | Percher | 925 |
| RMNH.5011582_Isostictidae_Tanymecosticta_leptalea | 7.06666667 | 6.87096774 | Percher | 916 |
| RMNH.INS.229243_Isostictidae_Selysioneura_capreola | 7.61764706 | 7.25714286 | Percher | 918 |
| **Lauromacromiidae** | | | | |
| GEODE7771_Lauromacromiidae_Lauromacromia_dubitalis | 3.166666667 | 3.829787234 | Flyer | 934 |
| RU123_Lauromacromiidae_Lauromacromia_bedei | 3.166666667 | 3.829787234 | Flyer | 608 |
| RU126_Lauromacromiidae_Lauromacromia_picinguaba | 3.166666667 | 3.829787234 | Flyer | 808 |
| **Lestidae** | | | | |
| GEODE7665_Lestidae_Indolestes_albicaudus | 5.13513514 | 5.15789474 | Percher | 786 |
| GEODE7728_Lestidae_Austrolestes_leda | 4.11111111 | 4.525 | Percher | 906 |
| GEODE7730_Lestidae_Archilestes_grandis | 4.97674211 | 5.00195116 | Percher | 920 |
| GEODE7813_Lestidae_Lestes_plagiatus | 4.6041491 | 4.60267429 | Percher | 909 |
| OD1127_Lestidae_Orolestes_octomaculatus | 4.257575758 | 5.107692308 | Percher | 912 |
| OD122_Lestidae_Sympecma_paedisca | 4.85 | 4.6 | Percher | 888 |
| OD1278_Lestidae_Indolestes_cyaneus | 5.13513514 | 5.15789474 | Percher | 908 |
| OD1321_Lestidae_Lestes_disjunctus | 4.6041491 | 4.60267429 | Percher | 907 |
| OD63_Lestidae_Austrolestes_annulosus | 4.11111111 | 4.525 | Percher | 851 |
| RMNH.INS.507448_Lestidae_Chalcolestes_viridis | 4.365853659 | 4.585365854 | Percher | 912 |
| **Lestoideidae** | | | | |
| OD157_Lestoideidae_Lestoidea_conjuncta | 5.780487805 | 5.697674419 | Percher | 922 |
| OD43_Lestoideidae_Diphlebia_coerulescens | 5.215384615 | 4.735294118 | Percher | 906 |
| RMNH.5011424_Lestoideidae_Diphlebia_euphoeoides | 5.215384615 | 4.735294118 | Percher | 933 |
| **Libellulidae** | | | | |
| GEODE12746_Libellulidae_Akrothemis_risi | 3.025862069 | 4.461538462 | Percher | 387 |
| GEODE12747_Libellulidae_Amphithemis_curvistyla | 2.608695652 | 3.222222222 | Percher | 905 |
| GEODE12748_Libellulidae_Celebophlebia_dactylogastra | 3.24 | 4.195121951 | Percher | 869 |
| GEODE12749_Libellulidae_Celebothemis_delecollei | 3.37593985 | 4.55 | Percher | 896 |
| GEODE17725_Libellulidae_Orthemis_macrostigma | 3.18752405 | 4.59889563 | Percher | 931 |
| GEODE17728_Libellulidae_Orthemis_sulphurata | 3.18752405 | 4.59889563 | Percher | 924 |
| GEODE17755_Libellulidae_Anatya_januaria | 3.569620253 | 4.362318841 | Percher | 882 |
| GEODE17756_Libellulidae_Antidythemis_trameiformis | 3.54237288 | 4.55445545 | Percher | 236 |
| GEODE17757_Libellulidae_Archaeophlebia_martini | 2.72916667 | 3.81333333 | Percher | 651 |
| GEODE17758_Libellulidae_Atoconeura_kenya | 2.97487437 | 4.27083333 | Percher | 392 |
| GEODE17761_Libellulidae_Boninthemis_insularis | 3.08433735 | 4.63492064 | Percher | 223 |
| GEODE17929_Libellulidae_Scapanea_frontalis | 3.163793103 | 4.111111111 | Percher | 808 |
| GEODE7612_Libellulidae_Cannaphila_insularis | 3.47955016 | 4.75537578 | Percher | 182 |
| GEODE7628_Libellulidae_Rhodopygia_hinei | 3.31146482 | 4.53146285 | Percher | 250 |
| GEODE7638_Libellulidae_Nannophlebia_axiagasta | 3.0562249 | 4.03773585 | Percher | 626 |
| GEODE7650_Libellulidae_Diplacina_spec | 3.10746269 | 4.19011407 | Percher | 851 |
| GEODE7655_Libellulidae_Diplacina_persephone | 3.10746269 | 4.19011407 | Percher | 470 |
| GEODE7659_Libellulidae_Microtrigonia_gomphoides | 2.92391304 | 3.98648649 | Percher | 812 |
| GEODE7676_Libellulidae_Misagria_calverti | 3.69887374 | 4.92970163 | Percher | 899 |
| GEODE7698_Libellulidae_Erythemis_credula | 3.13688237 | 4.19493393 | Percher | 930 |
| GEODE7699_Libellulidae_Erythrodiplax_umbrata | 2.96539276 | 3.98291632 | Percher | 932 |
| GEODE7700_Libellulidae_Oligoclada_sp | 2.7792045 | 4.05936065 | Percher | 928 |
| GEODE7701_Libellulidae_Agrionoptera_insignis | 3.77620397 | 4.7359736 | Percher | 927 |
| GEODE7702_Libellulidae_Uracis_fastigiata | 2.75723368 | 3.72006014 | Percher | 928 |
| GEODE7707_Libellulidae_Elga_sp | 3.07471623 | 3.9556441 | Percher | 930 |
| GEODE7708_Libellulidae_Plathemis_subornata | 2.79724774 | 3.76418041 | Percher | 933 |
| GEODE7709_Libellulidae_Rhyothemis_notata | 3.04658436 | 4.02277096 | Percher | 930 |
| GEODE7711_Libellulidae_Celithemis_eponina | 2.60681332 | 3.79407154 | Percher | 929 |
| GEODE7712_Libellulidae_Perithemis_sp | 2.54409106 | 3.34655979 | Percher | 924 |
| GEODE7714_Libellulidae_Brachythemis_leucosticta | 2.95053989 | 3.89423445 | Percher | 930 |
| GEODE7735_Libellulidae_Ladona_julia | 2.9808918 | 4.02311388 | Percher | 931 |
| GEODE7738_Libellulidae_Brechmorhoga_mendax | 3.01418332 | 4.27825719 | Percher | 932 |
| GEODE7739_Libellulidae_Perithemis_tenera | 2.54409106 | 3.34655979 | Percher | 920 |
| GEODE7746_Libellulidae_Paltothemis_lineatipes | 2.66335907 | 4.30100673 | Percher | 932 |
| GEODE7756_Libellulidae_Pachydiplax_longipennis | 2.94262678 | 4.06280557 | Percher | 933 |
| GEODE7759_Libellulidae_Nannophya_australis | 2.34615385 | 3.42574257 | Percher | 930 |
| GEODE7760_Libellulidae_Austrothemis_nigrescens | 3.314655172 | 4.451428571 | Percher | 932 |
| GEODE7761_Libellulidae_Tauriphila_argo | 2.7707949 | 4.14907466 | Flyer | 927 |
| GEODE7762_Libellulidae_Macrothemis_nobilis | 2.928154 | 4.14129019 | Percher | 930 |
| GEODE7764_Libellulidae_Miathyria_marcella | 2.58674413 | 4.25459364 | Flyer | 930 |
| GEODE7766_Libellulidae_Zenithoptera_fasciata | 2.66324126 | 3.17657591 | Percher | 933 |
| GEODE7767_Libellulidae_Nephepeltia_phryne | 3.21571095 | 4.08368419 | Percher | 926 |
| GEODE7768_Libellulidae_Elasmothemis_williamsoni | 3.90510519 | 4.58482123 | Percher | 932 |
| GEODE7769_Libellulidae_Argyrothemis_argentea | 3.485714286 | 3.96875 | Percher | 926 |
| GEODE7770_Libellulidae_Gynothemis_pumilia | 2.896825397 | 3.514851485 | Percher | 926 |
| GEODE7776_Libellulidae_Micrathyria_didyma | 3.03889894 | 4.23231737 | Percher | 933 |
| GEODE7777_Libellulidae_Dythemis_nigra | 2.98945067 | 4.29395073 | Percher | 934 |
| GEODE7798_Libellulidae_Palpopleura_lucia | 2.93181818 | 3.4717608 | Percher | 908 |
| GEODE7800_Libellulidae_Diplacodes_trivialis | 2.96063261 | 3.98633147 | Percher | 928 |
| GEODE7801_Libellulidae_Crocothemis_servilia | 3.10775333 | 4.27019123 | Percher | 926 |
| GEODE7802_Libellulidae_Aethriamanta_rezia | 2.81132076 | 4.14193548 | Percher | 924 |
| GEODE7803_Libellulidae_Diastatops_obscura | 2.31090937 | 3.07790585 | Percher | 930 |
| GEODE7804_Libellulidae_Tetrathemis_fraseri | 2.87027027 | 3.53012048 | Percher | 934 |
| GEODE7805_Libellulidae_Acisoma_trifidum | 2.92199522 | 3.64458013 | Percher | 920 |
| GEODE7806_Libellulidae_Orthetrum_julia | 3.930232558 | 4.581081081 | Percher | 926 |
| GEODE7807_Libellulidae_Tramea_transmarina | 2.42767702 | 4.20920122 | Flyer | 923 |
| GEODE7815_Libellulidae_Urothemis_assignata | 3.37837838 | 4.2875817 | Percher | 864 |
| OD107_Libellulidae_Pantala_flavescens | 2.5287276 | 4.17827552 | Flyer | 872 |
| OD1091_Libellulidae_Brachythemis_contaminata | 2.95053989 | 3.89423445 | Percher | 783 |
| OD1094_Libellulidae_Nannophlebia_alexia | 3.0562249 | 4.03773585 | Percher | 743 |
| OD1095_Libellulidae_Sympetrum_corruptum | 2.63194789 | 3.74345689 | Percher | 916 |
| OD1106_Libellulidae_Zygonyx_flavicosta | 3.22428571 | 4.23948812 | Percher | 839 |
| OD1319_Libellulidae_Macrodiplax_cora | 2.56924349 | 4.07435084 | Percher | 920 |
| OD77_Libellulidae_Rhyothemis_graphiptera | 3.04658436 | 4.02277096 | Percher | 843 |
| OD82_Libellulidae_Brachydiplax_denticauda | 3.02 | 4.23684211 | Percher | 925 |
| RMNH.5007803_Libellulidae_Atoconeura_biordinata | 2.97487437 | 4.27083333 | Percher | 936 |
| RMNH.5008306_Libellulidae_Neurothemis_fluctuans | 2.67073171 | 3.5664557 | Percher | 927 |
| RMNH.5011411_Libellulidae_Notolibellula_bicolor | 3.139534884 | 3.641025641 | Percher | 926 |
| RMNH.5011412_Libellulidae_Microtrigonia_curvata | 2.92391304 | 3.98648649 | Percher | 930 |
| RMNH.5011413_Libellulidae_Bironides_glochidion | 3.06993007 | 4.15254237 | Percher | 931 |
| RMNH.INS.228976_Libellulidae_Tetrathemis_irregularis | 2.87027027 | 3.53012048 | Percher | 926 |
| RMNH.INS.228999_Libellulidae_Raphismia_bispina | 3.333333333 | 4.357142857 | Percher | 928 |
| RMNH.INS.500592_Libellulidae_Huonia_epinephela | 3.11163896 | 4.14328358 | Percher | 924 |
| RMNH.INS.500644_Libellulidae_Protorthemis_coronata | 3.74747475 | 4.30434783 | Percher | 935 |
| RMNH.INS.501123_Libellulidae_Acisoma_panorpoides | 2.92199522 | 3.64458013 | Percher | 931 |
| RMNH.INS.501152_Libellulidae_Pseudothemis_jorina | 3.62430939 | 3.60655738 | Percher | 925 |
| RMNH.INS.501189_Libellulidae_Aethriamanta_brevipennis | 2.81132076 | 4.14193548 | Percher | 932 |
| RMNH.INS.501196_Libellulidae_Rhodothemis_rufa | 3.35555556 | 4.25675676 | Percher | 932 |
| RMNH.INS.501275_Libellulidae_Orchithemis_xanthosoma | 3.30232558 | 4.50943396 | Percher | 932 |
| RMNH.INS.501284_Libellulidae_Brachygonia_oculata | 3.43636364 | 4.40425532 | Percher | 923 |
| RMNH.INS.501296_Libellulidae_Tholymis_tillarga | 2.69152229 | 4.04713805 | Flyer | 926 |
| RMNH.INS.501373_Libellulidae_Chalybeothemis_fluviatilis | 3.0483871 | 4.08163265 | Percher | 926 |
| RMNH.INS.501377_Libellulidae_Zyxomma_petiolatum | 2.95372751 | 3.80392157 | Percher | 930 |
| RMNH.INS.501398_Libellulidae_Pornothemis_sp | 3.416666667 | 4.618421053 | Percher | 929 |
| RMNH.INS.501662_Libellulidae_Eleuthemis_buettikoferi | 2.526315789 | 3.970149254 | Percher | 917 |
| RMNH.INS.502121_Libellulidae_Oxythemis_phoenicosceles | 2.92380952 | 4.03781513 | Percher | 924 |
| RMNH.INS.502150_Libellulidae_Chalcostephia_flavifrons | 3.31081081 | 4.4 | Percher | 925 |
| RMNH.INS.502177_Libellulidae_Notiothemis_robertsi | 3.13846154 | 4.11320755 | Percher | 923 |
| RMNH.INS.502225_Libellulidae_Aethiothemis_erythromelas | 2.96978852 | 3.92481203 | Percher | 932 |
| RMNH.INS.502285_Libellulidae_Cyanothemis_simpsoni | 3.06617647 | 4.11607143 | Percher | 925 |
| RMNH.INS.502287_Libellulidae_Micromacromia_camerunica | 3 | 3.96682465 | Percher | 926 |
| RMNH.INS.502304_Libellulidae_Parazyxomma_flavicans | 2.583333333 | 3.137931034 | Percher | 934 |
| RMNH.INS.502305_Libellulidae_Hadrothemis_camarensis | 3.43106796 | 4.33180778 | Percher | 929 |
| RMNH.INS.502397_Libellulidae_Malgassophlebia_bispina | 2.91358025 | 3.95454546 | Percher | 922 |
| RMNH.INS.502400_Libellulidae_Nesciothemis_farinosa | 3.05183585 | 3.88802083 | Percher | 929 |
| RMNH.INS.502847_Libellulidae_Thermochoria_jeanneli | 3.15625 | 4.078740157 | Percher | 924 |
| RMNH.INS.503416_Libellulidae_Lyriothemis_cleis | 3.04761905 | 4.39184953 | Percher | 931 |
| RMNH.INS.503419_Libellulidae_Nesoxenia_lineata | 3.61437909 | 4.6171875 | Percher | 929 |
| RMNH.INS.503939_Libellulidae_Hydrobasileus_croceus | 3.224 | 3.96091205 | Percher | 932 |
| RMNH.INS.503943_Libellulidae_Indothemis_limbata | 3.15819209 | 4.23188406 | Percher | 913 |
| RMNH.INS.503957_Libellulidae_Potamarcha_congener | 2.915662651 | 3.808823529 | Percher | 924 |
| RMNH.INS.503963_Libellulidae_Lathrecista_asiatica | 3.809090909 | 4.547368421 | Percher | 917 |
| RMNH.INS.503964_Libellulidae_Urothemis_signata | 3.37837838 | 4.2875817 | Percher | 918 |
| RMNH.INS.504032_Libellulidae_Onychothemis_culminicola | 3.08539945 | 4.27075812 | Percher | 916 |
| RMNH.INS.504039_Libellulidae_Orchithemis_pulcherrima | 3.30232558 | 4.50943396 | Percher | 924 |
| RMNH.INS.504459_Libellulidae_Hemistigma_albipunctum | 3.03846154 | 3.77272727 | Percher | 936 |
| RMNH.INS.504999_Libellulidae_Orthetrum_cancellatum | 3.930232558 | 4.581081081 | Percher | 927 |
| RMNH.INS.505354_Libellulidae_Trithetrum_navasi | ? | ? | Percher | 926 |
| RMNH.INS.505362_Libellulidae_Crocothemis_erythraea | 3.10775333 | 4.27019123 | Percher | 933 |
| RMNH.INS.505380_Libellulidae_Olpogastra_lugubris | 3.36111111 | 4.36046512 | Percher | 932 |
| RMNH.INS.505547_Libellulidae_Porpax_risi | 3.34228188 | 4.05555556 | Percher | 926 |
| RMNH.INS.505588_Libellulidae_Diplacodes_lefebvrii | 2.96063261 | 3.98633147 | Percher | 927 |
| RMNH.INS.505652_Libellulidae_Bradinopyga_cornuta | 3.27891157 | 4.30567686 | Percher | 930 |
| RMNH.INS.505880_Libellulidae_Tapeinothemis_boharti | 3.606741573 | 4.986111111 | Percher | 936 |
| RMNH.INS.506254_Libellulidae_Camacinia_gigantea | 3.01020408 | 4.17525773 | Percher | 802 |
| RMNH.INS.506290_Libellulidae_Cratilla_lineata | 3.80579784 | 4.78231228 | Percher | 920 |
| RMNH.INS.507392_Libellulidae_Leucorrhinia_rubicunda | 2.9122135 | 3.81909169 | Percher | 934 |
| RMNH.INS.507400_Libellulidae_Libellula_depressa | 3.14477186 | 4.3500372 | Percher | 930 |
| RMNH.INS.507439_Libellulidae_Libellula_quadrimaculata | 3.14477186 | 4.3500372 | Percher | 931 |
| RMNH.INS.507465_Libellulidae_Sympetrum_danae | 2.63194789 | 3.74345689 | Percher | 929 |
| RMNH.INS.507492_Libellulidae_Nannodiplax_rubra | 2.755555556 | 3.5 | Percher | 921 |
| RMNH.INS.507529_Libellulidae_Trithemis_annulata | 2.76976744 | 4.14527027 | Percher | 921 |
| RMNH.INS.507551_Libellulidae_Selysiothemis_nigra | 2.65591398 | 3.44444444 | Percher | 929 |
| RMNH.INS.507746_Libellulidae_Hylaeothemis_clementia | 3.11026616 | 4.15492958 | Percher | 932 |
| RMNH.INS.507831_Libellulidae_Tyriobapta_torrida | 2.74324324 | 3.92982456 | Percher | 932 |
| RMNH.INS.508001_Libellulidae_Zygonyx_torridus | 3.22428571 | 4.23948812 | Percher | 926 |
| RMNH.INS.508447_Libellulidae_Palpopleura_sexmaculata | 2.93181818 | 3.4717608 | Percher | 921 |
| RMNH.INS.508515_Libellulidae_Nannophyopsis_clara | 2.03030303 | 3.35294118 | Percher | 927 |
| RMNH.INS.508797_Libellulidae_Neodythemis_afra | 3.24200913 | 4.24719101 | Percher | 926 |
| RMNH.INS.509795_Libellulidae_Thalassothemis_marchali | 3.39047619 | 4.641025641 | Percher | 926 |
| RMNH.INS.559789_Libellulidae_Zygonoides_fuelleborni | 3.206349206 | 4.168421053 | Percher | 927 |
| TM207_Libellulidae_Brechmorhoga_vivax | 3.01418332 | 4.27825719 | Percher | 925 |
| TM332_Libellulidae_Cannaphila_vibex | 3.47955016 | 4.75537578 | Percher | 912 |
| TM342_Libellulidae_Macrothemis_pseudimitans | 2.928154 | 4.14129019 | Percher | 921 |
| TM409_Libellulidae_Rhodopygia_hinei | 3.31146482 | 4.53146285 | Percher | 918 |
| TM532_Libellulidae_Erythemis_plebeja | 3.13688237 | 4.19493393 | Percher | 910 |
| **Macromidiidae** | | | | |
| GEODE17874_Macromidiidae_Macromidia_genialis | 3.01522843 | 4.21052632 | Flyer | 930 |
| GEODE17876_Macromidiidae_Macromidia_kelloggi | 3.01522843 | 4.21052632 | Flyer | 930 |
| RMNH.INS.506247_Macromidiidae_Macromidia_fulva | 3.01522843 | 4.21052632 | Flyer | 930 |
| **Macromiidae** | | | | |
| GEODE17622_Macromiidae_Macromia_astarte | 3.41770018 | 4.41804763 | Flyer | 903 |
| GEODE7715_Macromiidae_Phyllomacromia_picta | 3.38243626 | 4.26086957 | Flyer | 927 |
| OD1101_Macromiidae_Epophthalmia_elegans | 3.58130081 | 4.387471 | Flyer | 903 |
| RMNH.INS.507575_Macromiidae_Macromia_splendens | 3.41770018 | 4.41804763 | Flyer | 843 |
| **Megapodagrionidae** | | | | |
| GEODE7863_Megapodagrionidae_Allopodagrion_contortum | 3.42307692 | 4.55769231 | Percher | 845 |
| OD1281_Megapodagrionidae_Teinopodagrion_meridionale | 4.575 | 4.02272727 | Percher | 918 |
| **Mesagrionidae** | | | | |
| OD1183_Mesagrionidae_Mesagrion_leucorrhinum | 4.5945946 | 6.16666667 | Percher | 927 |
| **Mesopodagrionidae** | | | | |
| OD1077_Mesopodagrionidae_Mesopodagrion_sp | 3.39705882 | 4.35714286 | Percher | 914 |
| **Neopetaliidae** | | | | |
| OD1113_Neopetaliidae_Neopetalia_punctata | 3.578947368 | 3.865384615 | Flyer | 931 |
| **Neophyidae** | | | | |
| RMNH.INS.502324_Neophyidae_Neophya_rutherfordi | 2.99137931 | 3.453608247 | Flyer | 903 |
| **Pentaphlebiidae** | | | | |
| OD1139_Pentaphlebiidae_Pentaphlebia_mangana | 4.276923077 | 5.111111111 | Percher | 909 |
| RMNH.INS.500093_Pentaphlebiidae_Pentaphlebia_stahli | 3.00760456 | 3.87614679 | Percher | 927 |
| **Perilestidae** | | | | |
| OD1120_Perilestidae_Perissolestes_remotus | 5.2 | 5.142857143 | Percher | 903 |
| **Petaluridae** | | | | |
| GEODE17940_Petaluridae_Petalura_litorea | 3.513513514 | 4.620689655 | Percher | 890 |
| GEODE17943_Petaluridae_Uropetala_chiltoni | 3.67460318 | 4.50691244 | Percher | 840 |
| GEODE7743_Petaluridae_Tanypteryx_hageni | 2.96820319 | 4.11517538 | Percher | 925 |
| OD1318_Petaluridae_Petalura_gigantea | 3.513513514 | 4.620689655 | Percher | 894 |
| PD166_Petaluridae_Tanypteryx_pryeri | 2.96820319 | 4.11517538 | Percher | 825 |
| **Philogangidae** | | | | |
| OD1079_Philogangidae_Philoganga_vetusta | 4.91358025 | 6.15789474 | Percher | 927 |
| **Philgeniidae** | | | | |
| OD1124_Philogeniidae_Philogenia_carrillica | 4.03961039 | 4.721171802 | Percher | 909 |
| TM201_Philogeniidae_Philogenia_martae | 4.03961039 | 4.721171802 | Percher | 916 |
| **Philosinidae** | | | | |
| OD1119_Philosinidae_Rhinagrion_sp | 5.083333333 | 5.03125 | Percher | 928 |
| OD1130_Philosinidae_Philosina_buchi | 4.262135922 | 4.712871287 | Percher | 924 |
| RMNH.INS.506835_Philosinidae_Rhinagrion_macrocephalum | 5.083333333 | 5.03125 | Percher | 937 |
| **Platycnemididae** | | | | |
| GEODE2652_Platycnemididae_Asthenocnemis_stephanodera | 4.03030303 | 4.48795181 | Percher | 682 |
| GEODE7624_Platycnemididae_Disparoneura_apicalis | 3.12984 | 0.62307 | Percher | 253 |
| GEODE7653_Platycnemididae_Esme_mudiensis | 6.42857143 | 5.11111111 | Percher | 740 |
| GEODE7656_Platycnemididae_Nososticta_selysii | 5.575757576 | 4.571428571 | Percher | 525 |
| GEODE7660_Platycnemididae_Nososticta_exul | 5.575757576 | 4.571428571 | Percher | 469 |
| GEODE7668_Platycnemididae_Calicnemia_pulverulans | 4.1682243 | 4.7184466 | Percher | 892 |
| GEODE7673_Platycnemididae_Calicnemia_erythromelas | 4.1682243 | 4.7184466 | Percher | 926 |
| GEODE7724_Platycnemididae_Coeliccia_cyanomelas | 5.24705882 | 5.51162791 | Percher | 927 |
| GEODE7727_Platycnemididae_Prodasineura_autumnalis | 5.54074074 | 5.78832117 | Percher | 923 |
| GEODE7750_Platycnemididae_Copera_nyansana | 4.06 | 4.25 | Percher | 924 |
| GEODE7796_Platycnemididae_Elattoneura_acuta | 2.52126 | 0.49266 | Percher | 914 |
| GEODE7811_Platycnemididae_Copera_marginipes | 4.06 | 4.25 | Percher | 916 |
| GEODE7864_Platycnemididae_Hylaeargia_magnifica | 4.72 | 5.163265306 | Percher | 880 |
| GEODE7866_Platycnemididae_Lieftinckia_malaitae | 4.221052632 | 4.935483871 | Percher | 887 |
| GEODE7868_Platycnemididae_Rhyacocnemis_prothoracica | 4.75609756 | 4.22222222 | Percher | 922 |
| OD1122_Platycnemididae_Prodasineura_croconota | 5.54074074 | 5.78832117 | Percher | 912 |
| OD1123_Platycnemididae_Coeliccia_pyriformis | 5.24705882 | 5.51162791 | Percher | 915 |
| OD1132_Platycnemididae_Onychargia_atrocyana | 3.34883721 | 3.63333333 | Percher | 917 |
| OD1143_Platycnemididae_Mesocnemis_singularis | 4.476190476 | 4.731707317 | Percher | 926 |
| OD1306_Platycnemididae_Elattoneura_glauca | 2.52126 | 0.49266 | Percher | 913 |
| OD1317_Platycnemididae_Nososticta_solida | 5.575757576 | 4.571428571 | Percher | 924 |
| OD148_Platycnemididae_Platycnemis_pennipes | 4.74045802 | 4.89230769 | Percher | 905 |
| RMNH.5007870_Platycnemididae_Metacnemis_valida | ? | 4.851851852 | Percher | 907 |
| RMNH.5011406_Platycnemididae_Macrocnemis_gracilis | 4.120689655 | 4.767857143 | Percher | 925 |
| RMNH.INS.229282_Platycnemididae_Paracnemis_alluaudi | 4.218181818 | 4.592592593 | Percher | 929 |
| RMNH.INS.229291_Platycnemididae_Torrenticnemis_filicornis | 6.20930233 | 5.15384615 | Percher | 934 |
| RMNH.INS.500162_Platycnemididae_Allocnemis_contraria | 4.09803922 | 5 | Percher | 917 |
| RMNH.INS.500513_Platycnemididae_Lochmaeocnemis_malacodora | 6.525 | 5.02 | Percher | 923 |
| RMNH.INS.500580_Platycnemididae_Palaiargia_charmosyna | 4.125 | 4.37777778 | Percher | 919 |
| RMNH.INS.500639_Platycnemididae_Idiocnemis_obliterata | 4.92 | 5.040816327 | Percher | 918 |
| RMNH.INS.501977_Platycnemididae_Arrhenocnemis_parvibullis | ? | 5.535714286 | Percher | 908 |
| RMNH.INS.501995_Platycnemididae_Cyanocnemis_aureofrons | 4.081632653 | 4.595744681 | Percher | 914 |
| RMNH.INS.503929_Platycnemididae_Calicnemia_chaseni | 4.1682243 | 4.7184466 | Percher | 884 |
| RMNH.INS.503944_Platycnemididae_Pseudocopera_ciliata | 4.93023256 | 3.30434783 | Percher | 760 |
| RMNH.INS.505846_Platycnemididae_Arabicnemis_caerulea | ? | 4.381818182 | Percher | 865 |
| RMNH.INS.506232_Platycnemididae_Matticnemis_doi | 5.681818182 | 5.489795918 | Percher | 889 |
| RMNH.INS.508201_Platycnemididae_Arabineura_khalidi | ? | ? | Percher | 919 |
| RMNH.INS.508637_Platycnemididae_Indocnemis_orang | 4.07079646 | 4.626168224 | Percher | 928 |
| RMNH.INS.508794_Platycnemididae_Stenocnemis_pachystigma | 3.941176471 | 4.56 | Percher | 907 |
| RMNH.INS.559644_Platycnemididae_Spesbona_angusta | 4.804347826 | 5.363636364 | Percher | 908 |
| **Platystictidae** | | | | |
| GEODE7722_Platystictidae_Protosticta_satoi | 5.7 | 5.71052632 | Percher | 909 |
| GEODE7787_Platystictidae_Palaemnema_brevignoni | 4.83728743 | 3.07290622 | Percher | 914 |
| GEODE7871_Platystictidae_Sinosticta_sylvatica | 5.5 | 4.94230769 | Percher | 899 |
| GEODE7876_Platystictidae_Ceylonosticta_lankanensis | 4.790697674 | 5.023255814 | Percher | 912 |
| RMNH.5008397_Platystictidae_Telosticta_ulubaram | 5.542857143 | 5.764705882 | Percher | 890 |
| RMNH.INS.506261_Platystictidae_Telosticta_bidayuh | 5.542857143 | 5.764705882 | Percher | 886 |
| RMNH.INS.506492_Platystictidae_Sinosticta_ogatai | 5.5 | 4.94230769 | Percher | 916 |
| RMNH.INS.507659_Platystictidae_Drepanosticta_actaeon | 3.72393 | 0.68103 | Percher | 911 |
| **Polythoridae** | | | | |
| ANCH188_Polythoridae_Miocora_peraltica | 3.431034483 | 3.929824561 | Percher | 920 |
| BOY03_Polythoridae_Cora_xanthostoma | 4.189655172 | 2.983333333 | Percher | 919 |
| PP330_Polythoridae_Polythore_procera | 3.417556 | 3.48697946 | Percher | 929 |
| RMNH.INS.505717_Polythoridae_Chalcopteryx_rutilans | 3 | 3.12765957 | Percher | 920 |
| TM154_Polythoridae_Polythore_gigantea | 3.417556 | 3.48697946 | Percher | 916 |
| UAE1_Polythoridae_Euthore_fassli | 2.99409503 | 3.32047069 | Percher | 921 |
| **Priscagrionidae** | | | | |
| GEODE7872_Priscagrionidae_Sinocnemis_henanensis | 5.32142857 | 5.12962963 | Percher | 919 |
| OD1280_Priscagrionidae_Priscagrion_kiautai | 6.044776119 | 5.56 | Percher | 913 |
| **Protolestidae** | | | | |
| OD1136_Protolestidae_Protolestes_fickei | 4.547169811 | 4.959183673 | Percher | 923 |
| OD1308_Protolestidae_Protolestes_kerckhoffae | 4.547169811 | 4.959183673 | Percher | 922 |
| **Pseudocorduliidae** | | | | |
| GEODE17883_Pseudocorduliidae_Pseudocordulia_circularis | 2.94387755 | 3.875 | Flyer | 882 |
| GEODE17884_Pseudocorduliidae_Pseudocordulia_elliptica | 2.94387755 | 3.875 | Flyer | 920 |
| **Pseudolestidae** | | | | |
| OD1283_Pseudolestidae_Pseudolestes_mirabilis | 4.86538462 | 3.37931035 | Percher | 924 |
| **Rhipidolestidae** | | | | |
| OD1085_Rhipidolestidae_Agriomorpha_xinglongensis | 4.127272727 | 4.482142857 | Percher | 916 |
| OD1121_Rhipidolestidae_Agriomorpha_fusca | 4.127272727 | 4.482142857 | Percher | 920 |
| OD96_Rhipidolestidae_Rhipidolestes_aculeatus | 3.98184739 | 4.32721569 | Percher | 908 |
| RMNH.INS.502064_Rhipidolestidae_Rhipidolestes_truncatidens | 3.98184739 | 4.32721569 | Percher | 929 |
| **Rimanellidae** | | | | |
| GEODE7704_Rimanellidae_Rimanella_arcana | 5.56410256 | 5.18258258 | Percher | 926 |
| **Synlestidae** | | | | |
| GEODE7723_Synlestidae_Chlorolestes_elegans | 4.295081967 | 5.35483871 | Percher | 926 |
| GEODE7755_Synlestidae_Episynlestes_albicauda | 4.4 | 4.62962963 | Percher | 921 |
| GEODE7873_Synlestidae_Phylolestes_ethelae | 5.41071429 | 5.27586207 | Percher | 350 |
| OD1305_Synlestidae_Chlorolestes_umbratus | 4.295081967 | 5.35483871 | Percher | 913 |
| OD81_Synlestidae_Synlestes_weyersii | 5.11627907 | 5.3125 | Percher | 919 |
| RMNH.5007835_Synlestidae_Ecchlorolestes_nylephtha | 3.88332 | 0.78246 | Percher | 911 |
| RMNH.5011485_Synlestidae_Chorismagrion_risi | 4.47826087 | 4.63829787 | Percher | 912 |
| RMNH.INS.508632_Synlestidae_Megalestes_heros | 4.8030303 | 5.08695652 | Percher | 919 |
| RMNH.INS.508746_Synlestidae_Nubiolestes_diotima | 5.68888889 | 5.63829787 | Percher | 917 |
| **Synthemistidae** | | | | |
| GEODE17885_Synthemistidae_Archaeosynthemis_leachii | 3.0560166 | 3.84343434 | Flyer | 773 |
| GEODE17887_Synthemistidae_Archaeosynthemis_orientalis | 3.0560166 | 3.84343434 | Flyer | 895 |
| GEODE17888_Synthemistidae_Archaeosynthemis_spiniger | 3.0560166 | 3.84343434 | Flyer | 875 |
| GEODE17889_Synthemistidae_Austrosynthemis_cyanitincta | 3.09265176 | 3.89189189 | Flyer | 834 |
| GEODE17891_Synthemistidae_Eusynthemis_guttata | 3.11174242 | 4.05437352 | Flyer | 918 |
| GEODE17896_Synthemistidae_Calesynthemis_campioni | 2.98701299 | 3.92434211 | Flyer | 926 |
| GEODE17901_Synthemistidae_Synthemis_tasmanica | 2.98701299 | 3.92434211 | Flyer | 872 |
| GEODE17902_Synthemistidae_Tonyosynthemis_ofarrelli | 2.88852459 | 3.85294118 | Flyer | 354 |
| GEODE7643_Synthemistidae_Palaeosynthemis_gracilenta | 3.41237113 | 4.05882353 | Flyer | 338 |
| GEODE7680_Synthemistidae_Palaeosynthemis_cervula | 3.41237113 | 4.05882353 | Flyer | 662 |
| GEODE7737_Synthemistidae_Choristhemis_flavoterminata | 2.92328042 | 3.89768977 | Flyer | 927 |
| GEODE7792_Synthemistidae_Eusynthemis_nigra | 3.11174242 | 4.05437352 | Flyer | 930 |
| OD1311_Synthemistidae_Parasynthemis_regina | 3.501930502 | 4.213270142 | Flyer | 908 |
| RMNH.5011432_Synthemistidae_Tonyosynthemis_claviculata | 2.88852459 | 3.85294118 | Flyer | 929 |
| RMNH.INS.500525_Synthemistidae_Palaeosynthemis_primigenia | 3.41237113 | 4.05882353 | Flyer | 925 |
| RMNH.INS.504910_Synthemistidae_Synthemis_eustalacta | 2.98701299 | 3.92434211 | Flyer | 923 |
| **Tatocnemididae** | | | | |
| OD1137_Tatocnemididae_Tatocnemis_malgassica | 5.33333333 | 3.92982456 | Percher | 927 |
| **Thaumatoneuridae** | | | | |
| GEODE7874_Thaumatoneuridae_Paraphlebia_duodecima | 3.55128205 | 4.69333333 | Percher | 932 |
| OD1135_Thaumatoneuridae_Paraphlebia_sp. | 3.55128205 | 4.69333333 | Percher | 926 |
| OD1141_Thaumatoneuridae_Thaumatoneura_inopinata | 3.333333333 | 3.611570248 | Percher | 937 |
