# Supplementary material for: Soaring Systematics: An Evaluation of Biogeography and Flight Behavior in Dragonflies and Damselflies (Insecta: Odonata) Using Phylogenomics
Source: Syst Biol. 2026 Jan 22;75(5):984–1002. doi: 10.1093/sysbio/syag005 (PMC13445619; doi:10.1093/sysbio/syag005)
Supplement: Supplementary file 1 [file syag005_supplemental_files.zip › Supplemental Table 4 - Wing Scans per Genus.docx]

| ***Genus*** | **Number of Scans** |
| --- | --- |
| *Acanthallagma* | 1 |
| *Acisoma* | 5 |
| *Acrogomphus* | 1 |
| *Aeschnophlebia* | 2 |
| *Aeschnosoma* | 1 |
| *Aeshna* | 133 |
| *Aethiothemis* | 4 |
| *Aethriamanta* | 3 |
| *Agriogomphus* | 3 |
| *Agriomorpha* | 1 |
| *Agrionoptera* | 4 |
| *Agyrtacantha* | 6 |
| *Akrothemis* | 1 |
| *Allopetalia* | 1 |
| *Amanipodagrion* | 1 |
| *Amphiaeschna* | 1 |
| *Amphigomphus* | 4 |
| *Amphithemis* | 1 |
| *Anaciaeschna* | 19 |
| *Anax* | 40 |
| *Andaeschna* | 1 |
| *Anisogomphus* | 4 |
| *Anormogomphus* | 1 |
| *Anotogaster* | 4 |
| *Antidythemis* | 1 |
| *Antiopodophlebia* | 1 |
| *Antipodochlora* | 1 |
| *Antipodogomphus* | 1 |
| *Aphylla* | 34 |
| *Arabicnemis* | 1 |
| *Archaeogomphus* | 4 |
| *Archaeophlebia* | 1 |
| *Archaeophya* | 2 |
| *Archaeosynthemis* | 5 |
| *Archilestes* | 1 |
| *Archineura* | 1 |
| *Argirothemis* | 1 |
| *Arigomphus* | 81 |
| *Armagomphus* | 1 |
| *Arrhenocnemis* | 1 |
| *Asiagomphus* | 8 |
| *Atocorneura* | 4 |
| *Atrocalopteryx* | 1 |
| *Austroaeschna* | 9 |
| *Austroceonagrion* | 1 |
| *Austrocordulia* | 9 |
| *Austroepigomphus* | 4 |
| *Austrogomphus* | 8 |
| *Austrogynacantha* | 1 |
| *Austropetalia* | 5 |
| *Austrophlebia* | 1 |
| *Austrophya* | 1 |
| *Austrosynthemis* | 4 |
| *Austrothemis* | 1 |
| *Basiaeschna* | 27 |
| *Bironides* | 2 |
| *Boyeria* | 11 |
| *Brachydiplax* | 2 |
| *Brachygonia* | 2 |
| *Brachymesia* | 22 |
| *Brachythemis* | 5 |
| *Brachytron* | 1 |
| *Bradinopyga* | 5 |
| *Brechmorhoga* | 5 |
| *Burmagomphus* | 4 |
| *Cacoides* | 1 |
| *Camacinia* | 3 |
| *Cannaphila* | 4 |
| *Castoraeschna* | 1 |
| *Celebophlebia* | 1 |
| *Celithemis* | 150 |
| *Cephalaeschna* | 8 |
| *Ceratogomphus* | 3 |
| *Ceylonosticta* | 1 |
| *Chalcolestes* | 1 |
| *Chalcostephia* | 1 |
| *Chalybeothemis* | 2 |
| *Chlorogomphus* | 9 |
| *Choristhemis* | 4 |
| *Copera* | 1 |
| *Cordulegaster* | 90 |
| *Cordulephya* | 3 |
| *Cordulia* | 8 |
| *Corduliochlora* | 1 |
| *Cornigomphus* | 1 |
| *Coryphaeschna* | 25 |
| *Cratilla* | 3 |
| *Crenigomphus* | 4 |
| *Crocothemis* | 3 |
| *Cyanocnemis* | 1 |
| *Cyanogomphus* | 2 |
| *Cyclogomphus* | 1 |
| *Cyclophaea* | 1 |
| *Dasythemis* | 2 |
| *Davidius* | 4 |
| *Dendroaeschna* | 1 |
| *Diaphlebia* | 1 |
| *Diastatomma* | 2 |
| *Diastatops* | 13 |
| *Didymops* | 6 |
| *Diplacina* | 4 |
| *Diplacodes* | 5 |
| *Disparocypha* | 1 |
| *Dorocordulia* | 17 |
| *Dromaeschna* | 4 |
| *Dromogomphus* | 45 |
| *Dythemis* | 21 |
| *Ebegomphus* | 1 |
| *Elasmothemis* | 11 |
| *Eleuthemis* | 1 |
| *Elga* | 3 |
| *Epiaeschna* | 22 |
| *Epiophlebia* | 2 |
| *Epitheca* | 130 |
| *Epophthalmia* | 4 |
| *Erpetogomphus* | 57 |
| *Erythemis* | 175 |
| *Erythrodiplax* | 107 |
| *Eusynthemis* | 5 |
| *Euthygomphus* | 1 |
| *Fukienoogomphus* | 2 |
| *Fylgia* | 1 |
| *Gatrogomphus* | 1 |
| *Gomphaeschna* | 17 |
| *Gomphidia* | 7 |
| *Gomphidictinus* | 2 |
| *Gomphoides* | 2 |
| *Gomphomacromia* | 4 |
| *Gomphurus* | 172 |
| *Guadalca* | 1 |
| *Gynacantha* | 30 |
| *Gynacanthaeschna* | 1 |
| *Gynothemis* | 2 |
| *Hadrothemis* | 5 |
| *Hagenius* | 17 |
| *Heliaeschna* | 8 |
| *Heliogomphus* | 6 |
| *Helocordulia* | 13 |
| *Hemicordulia* | 5 |
| *Hemigomphus* | 8 |
| *Hemistigma* | 1 |
| *Hesperocordulia* | 2 |
| *Heteronaias* | 1 |
| *Heterophaea* | 1 |
| *Huonia* | 5 |
| *Hydrobasileus* | 6 |
| *Hylaeargia* | 1 |
| *Hylaeothemis* | 3 |
| *Hylogomphus* | 62 |
| *Hypothemis* | 1 |
| *Ictinogomphus* | 4 |
| *Idiataphe* | 1 |
| *Idiataphe* | 1 |
| *Idionyx* | 8 |
| *Idomacromia* | 2 |
| *Indaeschna* | 2 |
| *Indothemis* | 2 |
| *Isoaeshna* | 1 |
| *Isomma* | 1 |
| *Labrogomphus* | 1 |
| *Ladona* | 55 |
| *Lamelligomphus* | 6 |
| *Lanthanusa* | 2 |
| *Lanthrecista* | 1 |
| *Lanthus* | 21 |
| *Lathrocordulia* | 2 |
| *Lauromacromia* | 1 |
| *Leptocnemis* | 1 |
| *Leptogomphus* | 8 |
| *Lestinogomphus* | 2 |
| *Leucorrhinia* | 70 |
| *Libellula* | 343 |
| *Libyogomphus* | 1 |
| *Lindenia* | 1 |
| *Luzonobasis* | 1 |
| *Lyriothemis* | 8 |
| *Macrocnemis* | 1 |
| *Macrodiplax* | 9 |
| *Macrogomphus* | 8 |
| *Macromia* | 39 |
| *Macromidia* | 8 |
| *Macrothemis* | 43 |
| *Malgassophlebia* | 2 |
| *Matronoides* | 1 |
| *Mattigomphus* | 1 |
| *Megalogomphus* | 4 |
| *Melligomphus* | 4 |
| *Merogomphus* | 3 |
| *Mesocnemis* | 1 |
| *Metacnemis* | 1 |
| *Metagrion* | 1 |
| *Metaphya* | 1 |
| *Miathryia* | 16 |
| *Micrathyria* | 84 |
| *Microgomphus* | 2 |
| *Micromacromia* | 3 |
| *Micromidia* | 8 |
| *Microtrigonia* | 1 |
| *Miniargiolestes* | 1 |
| *Miocora* | 1 |
| *Misagria* | 2 |
| *Nannodiplax* | 1 |
| *Nannophlebia* | 8 |
| *Nannophya* | 5 |
| *Nannophyopsis* | 1 |
| *Nannothemis* | 20 |
| *Nasiaeschna* | 14 |
| *Navicordulia* | 1 |
| *Neallogaster* | 1 |
| *Nehalennia* | 1 |
| *Neocordulia* | 1 |
| *Neodythemis* | 6 |
| *Neogomphus* | 3 |
| *Neopetalia* | 1 |
| *Neophya* | 1 |
| *Nephepeltia* | 7 |
| *Nepogomphoides* | 1 |
| *Nepogomphus* | 1 |
| *Nesciothemis* | 5 |
| *Nesobasis* | 1 |
| *Nesocordulia* | 1 |
| *Nesogonia* | 1 |
| *Nesolestes* | 1 |
| *Nesoxenia* | 4 |
| *Neuraeschna* | 4 |
| *Neurocordulia* | 7 |
| *Neurogomphus* | 6 |
| *Neurothemis* | 5 |
| *Nihonogomphus* | 4 |
| *Notiothemis* | 4 |
| *Notoaeschna* | 1 |
| *Notogomphus* | 8 |
| *Notolibellula* | 1 |
| *Nychogomphus* | 11 |
| *Octogomphus* | 9 |
| *Oligoaeschna* | 7 |
| *Oligoclada* | 5 |
| *Olpogastra* | 1 |
| *Ophiogomphus* | 88 |
| *Oplonaeschna* | 2 |
| *Orchithemis* | 4 |
| *Oreaeschna* | 1 |
| *Orientogomphus* | 1 |
| *Orthemis* | 36 |
| *Orthetrum* | 1 |
| *Oxygastra* | 4 |
| *Oxythemis* | 4 |
| *Pachydiplax* | 68 |
| *Pacificothemis* | 2 |
| *Palaeosynthemis* | 2 |
| *Palpopleura* | 5 |
| *Paltothemis* | 14 |
| *Pantala* | 86 |
| *Paracnemis* | 1 |
| *Paracordulia* | 2 |
| *Paragomphus* | 4 |
| *Parasynthemis* | 1 |
| *Parazyxomma* | 1 |
| *Pentaphlebia* | 1 |
| *Pentathemis* | 3 |
| *Periaeschna* | 6 |
| *Pericnemis* | 2 |
| *Perigomphus* | 1 |
| *Perissogomphus* | 1 |
| *Perissolestes* | 1 |
| *Perithemis* | 79 |
| *Peruviogomphus* | 1 |
| *Phaenandrogomphus* | 4 |
| *Phanogomphus* | 234 |
| *Phenes* | 1 |
| *Philoganga* | 1 |
| *Philogenia* | 2 |
| *Philosina* | 1 |
| *Phyllocycla* | 17 |
| *Phyllogomphoides* | 29 |
| *Phyllogomphus* | 1 |
| *Phyllomacromia* | 2 |
| *Phyllopetalia* | 2 |
| *Pinheyschna* | 1 |
| *Plagulibasis* | 1 |
| *Planaeschna* | 8 |
| *Planiplax* | 2 |
| *Plathemis* | 88 |
| *Plattycantha* | 2 |
| *Platystigma* | 1 |
| *Pleiogomphus* | 1 |
| *Podopteryx* | 1 |
| *Polycanthagyna* | 7 |
| *Pornothemis* | 1 |
| *Porpax* | 4 |
| *Potamarcha* | 1 |
| *Procordulia* | 7 |
| *Progomphus* | 62 |
| *Protolestes* | 1 |
| *Protorthemis* | 1 |
| *Pseudocordulia* | 2 |
| *Pseudoleon* | 22 |
| *Pseudothemis* | 4 |
| *Pyrrhargiolestes* | 1 |
| *Raphismia* | 1 |
| *Remartinia* | 1 |
| *Rhinagrion* | 1 |
| *Rhinoneura* | 1 |
| *Rhionaeschna* | 4 |
| *Rhipidolestes* | 1 |
| *Rhodopygia* | 3 |
| *Rhodothemis* | 4 |
| *Rhyacocnemis* | 1 |
| *Rhyothemis* | 5 |
| *Rialla* | 1 |
| *Rimanella* | 1 |
| *Saraeschna* | 1 |
| *Scalmogomphus* | 2 |
| *Scapanea* | 1 |
| *Selysiothemis* | 1 |
| *Shaogomphus* | 1 |
| *Sieboldius* | 1 |
| *Sinictinogomphus* | 1 |
| *Sinogomphus* | 1 |
| *Somatochlora* | 55 |
| *Spesbona* | 1 |
| *Spinaeschna* | 1 |
| *Staurophlebia* | 1 |
| *Stenagrion* | 1 |
| *Stenocnemis* | 1 |
| *Stenocypha* | 2 |
| *Stenogomphurus* | 7 |
| *Stylogomphus* | 18 |
| *Stylurus* | 38 |
| *Sympeca* | 1 |
| *Sympetrum* | 151 |
| *Syncordulia* | 2 |
| *Synlestes* | 1 |
| *Synthemiopsis* | 2 |
| *Synthemis* | 7 |
| *Tachopteryx* | 10 |
| *Tanypteryx* | 3 |
| *Tapeinothemis* | 1 |
| *Tauriphila* | 21 |
| *Telephlebia* | 7 |
| *Telosticta* | 1 |
| *Tetracanthagyna* | 1 |
| *Tetrathemis* | 3 |
| *Thalassothemis* | 1 |
| *Thaumatagrion* | 1 |
| *Thaumatonerua* | 1 |
| *Tholymis* | 9 |
| *Tonyosynthemis* | 3 |
| *Tramea* | 85 |
| *Tricanthagyna* | 13 |
| *Trigomphus* | 1 |
| *Trithemis* | 5 |
| *Tyriobapta* | 2 |
| *Uracis* | 9 |
| *Uropetala* | 2 |
| *Urothemis* | 4 |
| *Wahnesia* | 1 |
| *Watanabeopetalia* | 2 |
| *Williamsonia* | 2 |
| *Xanthocnemis* | 1 |
| *Zenithoptera* | 4 |
| *Zygonyx* | 5 |
| *Zyxomma* | 5 |
