# Supplementary material for: Soaring Systematics: An Evaluation of Biogeography and Flight Behavior in Dragonflies and Damselflies (Insecta: Odonata) Using Phylogenomics
Source: Syst Biol. 2026 Jan 22;75(5):984–1002. doi: 10.1093/sysbio/syag005 (PMC13445619; doi:10.1093/sysbio/syag005)
Supplement: Supplementary file 1 [file syag005_supplemental_files.zip › Supplemental Table 5 - Biogeographic Realm Designations.docx]

| **Sample ID** | **Nearctic** | **Neotropics** | **Palaearctic** | **Afrotropics** | **Madagascar & Seychelles** | **Indo-**  **Malayan** | **Wallacea** | **Australasia** | **Oceania** |
| --- | --- | --- | --- | --- | --- | --- | --- | --- | --- |
| GEODE7682_Aeschnosomatidae_Aeschnosoma_forcipula |  | x |  |  |  |  |  |  |  |
| OD1158_Aeschnosomatidae_Pentathemis_sp |  |  |  |  |  |  |  | x |  |
| GEODE17607_Aeshnidae_Andaeschna_rufipes |  | x |  |  |  |  |  |  |  |
| GEODE17609_Aeshnidae_Epiaeschna_heros | x |  |  |  |  |  |  |  |  |
| GEODE17610_Aeshnidae_Gomphaeschna_furcillata | x |  |  |  |  |  |  |  |  |
| GEODE17614_Aeshnidae_Oreaeschna_dictatrix |  |  |  |  |  |  |  | x | x |
| GEODE7669_Aeshnidae_Amphiaeschna_ampla |  |  |  |  |  | x |  |  |  |
| GEODE7690_Aeshnidae_Plattycantha_cornuta |  |  |  |  |  |  |  | x |  |
| GEODE7691_Aeshnidae_Heliaeschna_crassa |  |  |  | x |  | x | x |  |  |
| GEODE7740_Aeshnidae_Rhionaeschna_multicolor | x | x |  |  |  |  |  |  |  |
| OD1303_Aeshnidae_Gynacanthaeschna_sikkima |  |  |  |  |  | x |  |  |  |
| OD1316_Aeshnidae_Gynacantha_gracilis | x | x | x | x | x | x | x | x | x |
| OD830_Aeshnidae_Anax_walsinghami | x | x | x | x | x | x | x | x | x |
| RMNH.5011576_Aeshnidae_Austrogynacantha_heterogena |  |  |  |  |  |  |  | x | x |
| RMNH.INS.502232_Aeshnidae_Gynacantha_victoriae | x | x | x | x | x | x | x | x | x |
| RMNH.INS.504908_Aeshnidae_Telephlebia_godeffroyi |  |  |  |  |  |  |  | x |  |
| RMNH.INS.504941_Aeshnidae_Notoaeschna_sagittata |  |  |  |  |  |  |  | x |  |
| RMNH.INS.504965_Aeshnidae_Acanthaeschna_victoria |  |  |  |  |  |  |  | x |  |
| RMNH.INS.505016_Aeshnidae_Anax_imperator | x | x | x | x | x | x | x | x | x |
| RMNH.INS.505017_Aeshnidae_Isoaeshna_isoceles |  |  | x |  |  |  |  |  |  |
| RMNH.INS.506912_Aeshnidae_Indaeschna_grubaueri |  |  | x |  |  | x | x |  |  |
| RMNH.INS.507402_Aeshnidae_Brachytron_pratense |  |  | x |  |  | x |  |  |  |
| RMNH.INS.507528_Aeshnidae_Boyeria_cretensis | x |  | x |  |  | x |  |  |  |
| RMNH.INS.509145_Aeshnidae_Cephalaeschna_obversa |  |  |  |  |  | x |  |  |  |
| TM170_Aeshnidae_Remartinia_luteipennis | x | x |  |  |  |  |  |  |  |
| TM392_Aeshnidae_Rhionaeschna_joannisi | x | x |  |  |  |  |  |  |  |
| TM581_Aeshnidae_Andaeschna_occidentalis |  | x |  |  |  |  |  |  |  |
| OD1133_Amanipodagrionidae_Amanipodagrion_gilliesi |  |  |  | x |  |  |  |  |  |
| OD1148_Amphipterygidae_Amphipteryx_agrioides |  | x |  |  |  |  |  |  |  |
| GEODE7678_Argiolestidae_Luzonargiolestes_baltazarae |  |  |  |  |  |  | x |  |  |
| GEODE7716_Argiolestidae_Griseargiolestes_albescens |  |  |  |  |  |  |  | x |  |
| GEODE7820_Argiolestidae_Archiargiolestes_pusillissimus |  |  |  |  |  |  |  | x |  |
| GEODE7821_Argiolestidae_Eoargiolestes_ochraceus |  |  |  |  |  |  |  | x |  |
| GEODE7822_Argiolestidae_Nesolestes_radama |  |  |  |  | x |  |  |  |  |
| OD1083_Argiolestidae_Austroargiolestes_icteromelas |  |  |  |  |  |  |  | x |  |
| OD1309_Argiolestidae_Nesolestes_sp |  |  |  |  | x |  |  |  |  |
| OD162_Argiolestidae_Caledargiolestes_uniseries |  |  |  |  |  |  |  | x |  |
| RMNH.5008334_Argiolestidae_Podolestes_orientalis |  |  |  |  |  | x |  |  |  |
| RMNH.INS.500397_Argiolestidae_Allolestes_maclachlanii |  |  |  |  | x |  |  |  |  |
| RMNH.INS.500853_Argiolestidae_Eoargiolestes_ochraceus |  |  |  |  |  |  |  | x |  |
| RMNH.INS.500854_Argiolestidae_Trineuragrion_percostale |  |  |  |  |  |  |  | x |  |
| RMNH.INS.500862_Argiolestidae_Podopteryx_selysi |  |  |  |  |  |  |  | x |  |
| RMNH.INS.501973_Argiolestidae_Argiolestes_muller |  |  |  |  |  |  | x | x |  |
| RMNH.INS.501990_Argiolestidae_Pyrrhargiolestes_aulicus |  |  |  |  |  |  |  | x |  |
| RMNH.INS.502075_Argiolestidae_Metagrion_ornatum |  |  |  |  |  |  |  | x |  |
| RMNH.INS.502077_Argiolestidae_Miniargiolestes_minimus |  |  |  |  |  |  |  | x |  |
| RMNH.INS.502084_Argiolestidae_Archiargiolestes_parvulus |  |  |  |  |  |  |  | x |  |
| RMNH.INS.503407_Argiolestidae_Caledopteryx_sarasini |  |  |  |  |  |  |  | x |  |
| RMNH.INS.506145_Argiolestidae_Neurolestes_trinervis |  |  |  | x |  |  |  |  |  |
| RMNH.INS.543752_Argiolestidae_Wahnesia_kirbyi |  |  |  |  |  |  |  | x |  |
| GEODE17873_Austrocorduliidae_Lathrocordulia_metallica |  |  |  |  |  |  |  | x |  |
| GEODE7744_Austrocorduliidae_Micromidia_convergens |  |  |  |  |  |  |  | x |  |
| GEODE7791_Austrocorduliidae_Hesperocordulia_berthoudi |  |  |  |  |  |  |  | x |  |
| OD1172_Austrocorduliidae_Austrocordulia_refracta |  |  |  |  |  |  |  | x |  |
| OD1310_Austrocorduliidae_Cordulephya_pygmaea |  |  |  |  |  |  |  | x |  |
| GEODE17604_Austropetaliidae_Austropetalia_patricia |  |  |  |  |  |  |  | x |  |
| OD1112_Austropetaliidae_Phyllopetalia_apollo |  | x |  |  |  |  |  |  |  |
| OD1115_Austropetaliidae_Hypopetalia_pestilens |  | x |  |  |  |  |  |  |  |
| GEODE7705_Calopterygidae_Noguchiphaea_yoshikoae |  |  |  |  |  | x |  |  |  |
| GEODE7719_Calopterygidae_Calopteryx_aequabilis | x |  | x |  |  |  |  |  |  |
| GEODE7721_Calopterygidae_Neurobasis_chinensis |  |  |  |  |  | x | x | x |  |
| GEODE7748_Calopterygidae_Mnesarete_williamsoni |  | x |  |  |  |  |  |  |  |
| GEODE7779_Calopterygidae_Mnesarete_cupraea |  | x |  |  |  |  |  |  |  |
| GEODE7780_Calopterygidae_Hetaerina_caja | x | x |  |  |  |  |  |  |  |
| GEODE7823_Calopterygidae_Bryoplathanon_globifer |  | x |  |  |  |  |  |  |  |
| OD102_Calopterygidae_Matrona_basilaris |  |  | x |  |  | x |  |  |  |
| OD1125_Calopterygidae_Neurobasis_ianthinipennis |  |  |  |  |  | x | x | x |  |
| OD1170_Calopterygidae_Hetaerina_americana | x | x |  |  |  |  |  |  |  |
| OD126_Calopterygidae_Vestalis_apicalis |  |  |  |  |  | x | x |  |  |
| OD976_Calopterygidae_Ormenophlebia_imperatrix |  | x |  |  |  |  |  |  |  |
| RMNH.INS.501070_Calopterygidae_Echo_modesta |  |  |  |  |  | x |  |  |  |
| RMNH.INS.502054_Calopterygidae_Caliphaea_confusa |  |  |  |  |  | x |  |  |  |
| RMNH.INS.502132_Calopterygidae_Phaon_iridipennis |  |  |  | x | x |  |  |  |  |
| RMNH.INS.503116_Calopterygidae_Sapho_fumosa |  |  |  | x |  |  |  |  |  |
| RMNH.INS.505747_Calopterygidae_Vestalaria_smaragdina |  |  |  |  |  | x |  |  |  |
| RMNH.INS.505806_Calopterygidae_Psolodesmus_kuroiwae |  |  | x |  |  | x |  |  |  |
| RMNH.INS.507722_Calopterygidae_Matronoides_cyaneipennis |  |  |  |  |  | x |  |  |  |
| RMNH.INS.507799_Calopterygidae_Vestalis_amaryllis |  |  |  |  |  | x | x |  |  |
| RMNH.INS.508607_Calopterygidae_Mnais_mneme |  |  | x |  |  | x |  |  |  |
| RMNH.INS.508616_Calopterygidae_Atrocalopteryx_melli |  |  | x |  |  | x |  |  |  |
| RMNH.INS.508674_Calopterygidae_Archineura_incarnata |  |  |  |  |  | x |  |  |  |
| RMNH.INS.508844_Calopterygidae_Umma_longistigma |  |  |  | x |  |  |  |  |  |
| GEODE7666_Chlorocyphidae_Heliocypha_bisignata |  |  |  |  |  | x |  |  |  |
| GEODE7667_Chlorocyphidae_Libellago_greeni |  |  |  |  |  | x | x | x |  |
| GEODE7672_Chlorocyphidae_Cyrano_unicolor |  |  |  |  |  |  | x |  |  |
| GEODE7675_Chlorocyphidae_Disparocypha_biedermanni |  |  |  |  |  |  | x |  |  |
| GEODE7812_Chlorocyphidae_Heliocypha_perforata |  |  |  |  |  | x |  |  |  |
| OD120_Chlorocyphidae_Heliocypha_biseriata |  |  |  |  |  | x |  |  |  |
| OD134_Chlorocyphidae_Libellago_semiopaca |  |  |  |  |  | x | x | x |  |
| OD35_Chlorocyphidae_Rhinocypha_tincta |  |  |  |  |  | x | x | x |  |
| RMNH.INS.500989_Chlorocyphidae_Libellago_aurantiaca |  |  |  |  |  | x | x | x |  |
| RMNH.INS.501024_Chlorocyphidae_Sundacypha_petiolata |  |  |  |  |  | x |  |  |  |
| RMNH.INS.505345_Chlorocyphidae_Platycypha_caligata |  |  |  | x |  |  |  |  |  |
| RMNH.INS.505736_Chlorocyphidae_Aristocypha_fenestrella |  |  |  |  |  | x |  |  |  |
| RMNH.INS.506163_Chlorocyphidae_Africocypha_lacuselephantum |  |  |  | x |  |  |  |  |  |
| RMNH.INS.507707_Chlorocyphidae_Rhinoneura_villosipes |  |  |  |  |  | x |  |  |  |
| RMNH.INS.508833_Chlorocyphidae_Stenocypha_gracilis |  |  |  | x |  |  |  |  |  |
| GEODE7689_Chlorogomphidae_Chlorogomphus_spec |  |  | x |  |  | x | x |  |  |
| OD1315_Chlorogomphidae_Chlorogomphus_auratus |  |  | x |  |  | x | x |  |  |
| RMNH.INS.508528_Chlorogomphidae_Watanabeopetalia_usignata |  |  |  |  |  | x |  |  |  |
| GEODE2711_Coenagrionidae_Luzonobasis_glauca |  |  |  |  |  |  | x |  |  |
| GEODE2758_Coenagrionidae_Plagulibasis_ciliata |  |  |  |  |  |  |  | x |  |
| GEODE7697_Coenagrionidae_Protoneura_tenuis |  | x |  |  |  |  |  |  |  |
| GEODE7725_Coenagrionidae_Mecistogaster_linearis | x | x |  |  |  |  |  |  |  |
| GEODE7741_Coenagrionidae_Psaironeura_tenuissima |  | x |  |  |  |  |  |  |  |
| GEODE7749_Coenagrionidae_Amphiagrion_abbreviatum | x |  |  |  |  |  |  |  |  |
| GEODE7753_Coenagrionidae_Telebasis_salva | x | x |  |  |  |  |  |  |  |
| GEODE7758_Coenagrionidae_Acanthagrion_adustum | x | x |  |  |  |  |  |  |  |
| GEODE7778_Coenagrionidae_Metaleptobasis_bicornis |  | x |  |  |  |  |  |  |  |
| GEODE7781_Coenagrionidae_Phasmoneura_exigua |  | x |  |  |  |  |  |  |  |
| GEODE7782_Coenagrionidae_Tuberculobasis_yanomami |  | x |  |  |  |  |  |  |  |
| GEODE7783_Coenagrionidae_Telebasis_demarara | x | x |  |  |  |  |  |  |  |
| GEODE7784_Coenagrionidae_Epipotoneura_nehalennia |  | x |  |  |  |  |  |  |  |
| GEODE7785_Coenagrionidae_Inpabasis_rosea |  | x |  |  |  |  |  |  |  |
| GEODE7786_Coenagrionidae_Acanthallagma_luteum |  | x |  |  |  |  |  |  |  |
| GEODE7794_Coenagrionidae_Microstigma_anomalum |  | x |  |  |  |  |  |  |  |
| GEODE7819_Coenagrionidae_Aeolagrion_dorsale |  | x |  |  |  |  |  |  |  |
| GEODE7826_Coenagrionidae_Aceratobasis_macilenta |  | x |  |  |  |  |  |  |  |
| GEODE7827_Coenagrionidae_Amphiallagma_parvum |  |  |  |  |  | x |  |  |  |
| GEODE7828_Coenagrionidae_Anisagrion_allopterum |  | x |  |  |  |  |  |  |  |
| GEODE7829_Coenagrionidae_Antiagrion_grinbergsi |  | x |  |  |  |  |  |  |  |
| GEODE7830_Coenagrionidae_Apanisagrion_lais | x | x |  |  |  |  |  |  |  |
| GEODE7832_Coenagrionidae_Caliagrion_billinghursti |  |  |  |  |  |  |  | x |  |
| GEODE7833_Coenagrionidae_Calvertagrion_mauffrayi |  | x |  |  |  |  |  |  |  |
| GEODE7835_Coenagrionidae_Cyanallagma_angelae |  | x |  |  |  |  |  |  |  |
| GEODE7836_Coenagrionidae_Diceratobasis_melanogaster |  | x |  |  |  |  |  |  |  |
| GEODE7839_Coenagrionidae_Telagrion_longum |  | x |  |  |  |  |  |  |  |
| GEODE7841_Coenagrionidae_Peristicta_aeneoviridis |  | x |  |  |  |  |  |  |  |
| GEODE7844_Coenagrionidae_Mesamphiagrion_demarmelsi |  | x |  |  |  |  |  |  |  |
| GEODE7848_Coenagrionidae_Oxyagrion_hermosae |  | x |  |  |  |  |  |  |  |
| GEODE7852_Coenagrionidae_Homeoura_lindneri |  | x |  |  |  |  |  |  |  |
| GEODE7854_Coenagrionidae_Leptobasis_melinogaster | x | x |  |  |  |  |  |  |  |
| GEODE7857_Coenagrionidae_Platystigma_astictum |  | x |  |  |  |  |  |  |  |
| GEODE7879_Coenagrionidae_Forcepsioneura_sancta |  | x |  |  |  |  |  |  |  |
| GEODE7880_Coenagrionidae_Denticulobasis_dunklei |  | x |  |  |  |  |  |  |  |
| GEODE7882_Coenagrionidae_Andinagrion_peterseni |  | x |  |  |  |  |  |  |  |
| OD103_Coenagrionidae_Neoneura_mariana | x | x |  |  |  |  |  |  |  |
| OD105_Coenagrionidae_Protoneura_capillaris |  | x |  |  |  |  |  |  |  |
| OD1116_Coenagrionidae_Cyanallagma_sp |  | x |  |  |  |  |  |  |  |
| OD136_Coenagrionidae_Papuagrion_occipitale |  |  |  |  |  |  |  | x |  |
| RMNH.5007797_Coenagrionidae_Azuragrion_nigridorsum |  |  | x | x | x |  |  |  |  |
| RMNH.5007810_Coenagrionidae_Africallagma_glaucum |  |  |  | x | x |  |  |  |  |
| RMNH.5010307_Coenagrionidae_Proischnura_polychromatica |  |  |  | x |  |  |  |  |  |
| RMNH.INS.229208_Coenagrionidae_Oreocnemis_phoenix |  |  |  | x |  |  |  |  |  |
| RMNH.INS.500398_Coenagrionidae_Leptocnemis_cyanops |  |  |  |  | x |  |  |  |  |
| RMNH.INS.501979_Coenagrionidae_Thaumatagrion_funereum |  |  |  |  |  |  |  | x |  |
| RMNH.INS.503604_Coenagrionidae_Archibasis_viola |  |  |  |  |  | x | x | x |  |
| RMNH.INS.503619_Coenagrionidae_Aciagrion_borneense |  |  | x | x |  | x | x | x |  |
| RMNH.INS.503951_Coenagrionidae_Argiocnemis_rubescens |  |  |  |  | x | x | x | x |  |
| RMNH.INS.504852_Coenagrionidae_Austrocoenagrion_lyelli |  |  |  |  |  |  |  | x |  |
| RMNH.INS.505254_Coenagrionidae_Austrocnemis_splendida |  |  |  |  |  |  |  | x |  |
| RMNH.INS.507703_Coenagrionidae_Stenagrion_dubium |  |  |  |  |  | x | x |  |  |
| RMNH.INS.509787_Coenagrionidae_Coenagriocnemis_rufipes |  |  |  |  | x |  |  |  |  |
| RMNH.INS.509800_Coenagrionidae_Coenagriocnemis_insularis |  |  |  |  | x |  |  |  |  |
| RMNH.INS.557636_Coenagrionidae_Pericnemis_stictica |  |  |  |  |  | x | x |  |  |
| TM305_Coenagrionidae_Acanthagrion_trilobatum | x | x |  |  |  |  |  |  |  |
| TMCH16_Coenagrionidae_Protoneura_amatoria |  | x |  |  |  |  |  |  |  |
| OD1164_Cordulegastridae_Cordulegaster_sp | x | x | x |  |  | x |  |  |  |
| OD1304_Cordulegastridae_Neallogaster_hermionae |  |  | x |  |  | x |  |  |  |
| RMNH.INS.508536_Cordulegastridae_Anotogaster_sieboldii |  |  | x |  |  | x |  |  |  |
| GEODE17698_Corduliidae_Dorocordulia_lepida | x |  |  |  |  |  |  |  |  |
| GEODE17699_Corduliidae_Dorocordulia_libera | x |  |  |  |  |  |  |  |  |
| GEODE17703_Corduliidae_Epitheca_cynosura | x |  | x |  |  |  |  |  |  |
| GEODE17704_Corduliidae_Epitheca_marginata | x |  | x |  |  |  |  |  |  |
| GEODE17707_Corduliidae_Epitheca_semiaquea | x |  | x |  |  |  |  |  |  |
| GEODE17711_Corduliidae_Guadalca_insularis |  |  |  |  |  |  |  | x | x |
| GEODE17712_Corduliidae_Helocordulia_selysii | x |  |  |  |  |  |  |  |  |
| GEODE17713_Corduliidae_Helocordulia_uhleri | x |  |  |  |  |  |  |  |  |
| GEODE17717_Corduliidae_Hemicordulia_fidelis |  |  | x | x | x | x | x | x | x |
| GEODE17720_Corduliidae_Hemicordulia_kalliste |  |  | x | x | x | x | x | x | x |
| GEODE17779_Corduliidae_Navicordulia_errans |  | x |  |  |  |  |  |  |  |
| GEODE17781_Corduliidae_Neurocordulia_virginiensis | x |  |  |  |  |  |  |  |  |
| GEODE17783_Corduliidae_Neurocordulia_yamaskanensis | x |  |  |  |  |  |  |  |  |
| GEODE17788_Corduliidae_Procordulia_jacksoniensis |  |  |  |  |  | x | x | x | x |
| GEODE17789_Corduliidae_Procordulia_moroensis |  |  |  |  |  | x | x | x | x |
| GEODE17793_Corduliidae_Neurocordulia_michaeli | x |  |  |  |  |  |  |  |  |
| GEODE17832_Corduliidae_Somatochlora_cingulata | x |  | x |  |  | x |  |  |  |
| GEODE7747_Corduliidae_Cordulia_shurtleffii | x |  | x |  |  |  |  |  |  |
| GEODE7765_Corduliidae_Paracordulia_sericea |  | x |  |  |  |  |  |  |  |
| OD1322_Corduliidae_Rialla_villosa |  | x |  |  |  |  |  |  |  |
| OD146_Corduliidae_Cordulia_aenea | x |  | x |  |  |  |  |  |  |
| OD83_Corduliidae_Hemicordulia_australiae |  |  | x | x | x | x | x | x | x |
| RMNH.5009497_Corduliidae_Corduliochlora_borisi |  |  | x |  |  |  |  |  |  |
| RMNH.5011578_Corduliidae_Metaphya_tillyardi |  |  |  |  |  | x |  | x |  |
| RMNH.INS.500518_Corduliidae_Procordulia_leopoldi |  |  |  |  |  | x | x | x | x |
| RMNH.INS.506657_Corduliidae_Heteronaias_heterodoxa |  |  |  |  |  |  | x |  |  |
| OD151_Devadattidae_Devadatta_podolestoides |  |  |  |  |  | x | x |  |  |
| RMNH.INS.501127_Devadattidae_Devadatta_argyoides |  |  |  |  |  | x | x |  |  |
| GEODE7718_Dicteriadidae_Dicterias_atrosanguinea |  | x |  |  |  |  |  |  |  |
| GEODE7788_Dicteriadidae_Heliocharis_amazona |  | x |  |  |  |  |  |  |  |
| SRR1695233_Ephemeroptera_Ecdyonurus_insignis | x | x | x |  |  |  |  |  |  |
| SRR921569_Ephemeroptera_Baetis_sp | x | x | x | x | x | x |  | x |  |
| SRR921591_Ephemeroptera_Ephemera_danica | x | x | x | x |  | x |  |  |  |
| SRR921596_Ephemeroptera_Eurylophella_sp | x |  | x |  | x |  |  |  |  |
| SRR921608_Ephemeroptera_Isonychia_bicolor | x | x | x |  |  | x |  |  |  |
| OD1076_Epiophlebiidae_Epiophlebia_superstes |  |  | x |  |  | x |  |  |  |
| OD1302_Epiophlebiidae_Epiophlebia_laidlawi |  |  | x |  |  | x |  |  |  |
| GEODE2756_Euphaeidae_Heterophaea_barbata |  |  |  |  |  |  | x |  |  |
| GEODE7683_Euphaeidae_Anisopleura_lestoides |  |  |  |  |  | x |  |  |  |
| GEODE7686_Euphaeidae_Euphaea_cardinalis |  |  | x |  |  | x | x |  |  |
| GEODE7687_Euphaeidae_Cyclophaea_cyanifrons |  |  |  |  |  |  | x |  |  |
| GEODE7694_Euphaeidae_Euphaea_refulgens |  |  | x |  |  | x | x |  |  |
| GEODE7858_Euphaeidae_Cryptophaea_saukra |  |  |  |  |  | x |  |  |  |
| OD132_Euphaeidae_Euphaea_ochracea |  |  | x |  |  | x | x |  |  |
| OD99_Euphaeidae_Bayadera_brevicauda |  |  | x |  |  | x |  |  |  |
| RMNH.5009494_Euphaeidae_Epallage_fatime |  |  | x |  |  | x |  |  |  |
| RMNH.INS.501089_Euphaeidae_Dysphaea_dimidiata |  |  |  |  |  | x | x |  |  |
| RMNH.INS.505757_Euphaeidae_Anisopleura_furcata |  |  |  |  |  | x |  |  |  |
| RMNH.INS.505817_Euphaeidae_Cryptophaea_vietnamensis |  |  |  |  |  | x |  |  |  |
| GEODE12753_Gomphidae_Lindenia_tetraphylla |  |  | x |  |  |  |  |  |  |
| GEODE12754_Gomphidae_Mattigomphus_pinratani |  |  |  |  |  | x |  |  |  |
| GEODE12755_Gomphidae_Perissogomphus_stevensi |  |  |  |  |  | x |  |  |  |
| GEODE17903_Gomphidae_Anisogomphus_anderi |  |  | x |  |  | x |  |  |  |
| GEODE17904_Gomphidae_Arigomphus_furcifer | x |  |  |  |  |  |  |  |  |
| GEODE17909_Gomphidae_Zonophora_diversa |  | x |  |  |  |  |  |  |  |
| GEODE17911_Gomphidae_Cacoides_latro |  | x |  |  |  |  |  |  |  |
| GEODE17912_Gomphidae_Hagenius_brevistylus | x |  |  |  |  |  |  |  |  |
| GEODE17913_Gomphidae_Fukienogomphus_promineus |  |  |  |  |  | x |  |  |  |
| GEODE17914_Gomphidae_Shaogomphus_postocularis |  |  | x |  |  | x |  |  |  |
| GEODE17915_Gomphidae_Peruviogomphus_pearsoni | x |  |  |  |  |  |  |  |  |
| GEODE17917_Gomphidae_Octogomphus_specularis | x |  |  |  |  |  |  |  |  |
| GEODE17919_Gomphidae_Gastrogomphus_abdominalis |  |  | x |  |  | x |  |  |  |
| GEODE17922_Gomphidae_Melligomphus_ardens |  |  | x |  |  | x |  |  |  |
| GEODE17923_Gomphidae_Merogomphus_longistigma |  |  |  |  |  | x |  |  |  |
| GEODE17933_Gomphidae_Scalmogomphus_falcatus |  |  |  |  |  | x |  |  |  |
| GEODE17935_Gomphidae_Anormogomphus_kiritshenkoi |  |  | x |  |  | x |  |  |  |
| GEODE17936_Gomphidae_Phanogomphus_exilis | x |  |  |  |  |  |  |  |  |
| GEODE7634_Gomphidae_Sinogomphus_telamon |  |  | x |  |  | x |  |  |  |
| GEODE7681_Gomphidae_Phyllogomphoides_undulatus | x | x |  |  |  |  |  |  |  |
| GEODE7692_Gomphidae_Phyllogomphoides_fuliginosus | x | x |  |  |  |  |  |  |  |
| GEODE7710_Gomphidae_Ebegomphus_sp |  | x |  |  |  |  |  |  |  |
| GEODE7731_Gomphidae_Ictinogomphus_pertinax |  |  | x | x |  | x | x | x | x |
| GEODE7732_Gomphidae_Gomphurus_externus | x |  |  |  |  |  |  |  |  |
| GEODE7733_Gomphidae_Tragogomphus_ellioti |  |  |  | x |  |  |  |  |  |
| GEODE7734_Gomphidae_Heliogomphus_scorpio |  |  |  |  |  | x | x |  |  |
| GEODE7736_Gomphidae_Erpetogomphus_compositus | x | x |  |  |  |  |  |  |  |
| GEODE7745_Gomphidae_Armagomphus_armiger |  |  |  |  |  |  |  | x |  |
| GEODE7754_Gomphidae_Stenogomphurus_rogersi | x |  |  |  |  | x |  |  |  |
| GEODE7763_Gomphidae_Archaeogomphus_nanus |  | x |  |  |  |  |  |  |  |
| GEODE7772_Gomphidae_Ebegomphus_minutus |  | x |  |  |  |  |  |  |  |
| GEODE7773_Gomphidae_Zonophora_batesi |  | x |  |  |  |  |  |  |  |
| GEODE7774_Gomphidae_Desmogomphus_tigrivensis |  | x |  |  |  |  |  |  |  |
| GEODE7775_Gomphidae_Agriogomphus_sylvicola |  | x |  |  |  |  |  |  |  |
| GEODE7790_Gomphidae_Antipodogomphus_acolythus |  |  |  |  |  |  |  | x |  |
| GEODE7818_Gomphidae_Aphylla_sp | x | x |  |  |  |  |  |  |  |
| OD1314_Gomphidae_Ophiogomphus_morrisoni | x |  | x |  |  | x |  |  |  |
| OD69_Gomphidae_Ictinogomphus_australis |  |  | x | x |  | x | x | x | x |
| OD85_Gomphidae_Austrogomphus_amphiclitus |  |  |  |  |  |  |  | x |  |
| RMNH.5008090_Gomphidae_Ceratogomphus_pictus |  |  |  | x |  |  |  |  |  |
| RMNH.5009376_Gomphidae_Onychogomphus_styx |  |  | x | x | x | x |  |  |  |
| RMNH.5011399_Gomphidae_Stylurus_flavipes | x | x | x |  |  | x |  |  |  |
| RMNH.5011415_Gomphidae_Austroepigomphus_praeruptus |  |  |  |  |  |  |  | x |  |
| RMNH.5011429_Gomphidae_Hemigomphus_comitatus |  |  |  |  |  |  |  | x |  |
| RMNH.5011478_Gomphidae_Austrogomphus_prasinus |  |  |  |  |  |  | H |  |  |
| RMNH.INS.229080_Gomphidae_Macrogomphus_quadratus |  |  |  |  |  | x |  |  |  |
| RMNH.INS.501184_Gomphidae_Phaenandrogomphus_asthenes |  |  |  |  |  | x | x |  |  |
| RMNH.INS.501444_Gomphidae_Libyogomphus_christinae |  |  |  | x |  |  |  |  |  |
| RMNH.INS.501518_Gomphidae_Microgomphus_camerunensis |  |  |  | x |  | x | x |  |  |
| RMNH.INS.502394_Gomphidae_Lestinogomphus_congoensis |  |  |  | x |  |  |  |  |  |
| RMNH.INS.502555_Gomphidae_Gomphidia_gamblesi |  |  | x | x |  | x | x |  |  |
| RMNH.INS.502891_Gomphidae_Nepogomphoides_stuhlmanni |  |  |  | x |  |  |  |  |  |
| RMNH.INS.503108_Gomphidae_Diastatomma_gamblesi |  |  |  | x |  |  |  |  |  |
| RMNH.INS.503523_Gomphidae_Sieboldius_japponicus |  |  | x |  |  | x |  |  |  |
| RMNH.INS.503524_Gomphidae_Acrogomphus_jubilaris |  |  |  |  |  | x | x |  |  |
| RMNH.INS.504961_Gomphidae_Austrogomphus_ochraceus |  |  |  |  |  |  |  | x |  |
| RMNH.INS.505397_Gomphidae_Neurogomphus_cocytius |  |  |  | x |  |  |  |  |  |
| RMNH.INS.505653_Gomphidae_Notogomphus_praetorius |  |  |  | x |  |  |  |  |  |
| RMNH.INS.505754_Gomphidae_Amphigomphus_somnuki |  |  |  |  |  | x |  |  |  |
| RMNH.INS.506264_Gomphidae_Leptogomphus_coomansi |  |  | x |  |  | x | x |  |  |
| RMNH.INS.506367_Gomphidae_Megalogomphus_borneensis |  |  |  |  |  | x |  |  |  |
| RMNH.INS.507519_Gomphidae_Ophiogomphus_cecilia | x |  | x |  |  | x |  |  |  |
| RMNH.INS.507929_Gomphidae_Phyllogomphus_annulus |  |  |  | x |  |  |  |  |  |
| RMNH.INS.508012_Gomphidae_Paragomphus_genei |  |  | x | x | x | x | x |  |  |
| RMNH.INS.508545_Gomphidae_Trigomphus_beatus |  |  | x |  |  | x |  |  |  |
| RMNH.INS.508624_Gomphidae_Fukienogomphus_promineus |  |  |  |  |  | x |  |  |  |
| RMNH.INS.508661_Gomphidae_Lamelligomphus_camelus |  |  | x |  |  | x |  |  |  |
| RMNH.INS.508662_Gomphidae_Melligomphus_sinicus |  |  | x |  |  | x |  |  |  |
| RMNH.INS.508663_Gomphidae_Nihonogomphus_semanticus |  |  | x |  |  | x |  |  |  |
| RMNH.INS.508672_Gomphidae_Nepogomphus_walli |  |  |  |  |  | x | x |  |  |
| RMNH.INS.508691_Gomphidae_Asiagomphus_hainanensis |  |  | x |  |  | x |  |  |  |
| RMNH.INS.508700_Gomphidae_Labrogomphus_torvus |  |  |  |  |  | x |  |  |  |
| RMNH.INS.509134_Gomphidae_Euthygomphus_koxingai |  |  |  |  |  | x |  |  |  |
| RMNH.INS.509144_Gomphidae_Dubitogomphus_bicornutus |  |  | x |  |  | x |  |  |  |
| RMNH.INS.509146_Gomphidae_Sinictinogomphus_clavatus |  |  | x |  |  | x |  |  |  |
| RMNH.INS.509207_Gomphidae_Burmagomphus_divaricatus |  |  | x |  |  | x | x |  |  |
| RMNH.INS.509920_Gomphidae_Lamelligomphus_castor |  |  | x |  |  | x |  |  |  |
| RMNH.INS.509971_Gomphidae_Orientogomphus_minor |  |  |  |  |  | x |  |  |  |
| RMNH.INS.554426_Gomphidae_Mastigogomphus_chapini |  |  |  | x |  |  |  |  |  |
| RMNH.INS.554510_Gomphidae_Cornigomphus_guineensis |  |  |  | x |  |  |  |  |  |
| RMNH.INS.559802_Gomphidae_Crenigomphus_hartmanni |  |  |  | x |  |  |  |  |  |
| GEODE17894_Gomphomacromiidae_Gomphomacromia_nodisticta |  | x |  |  |  |  |  |  |  |
| OD1169_Gomphomacromiidae_Gomphomacromia_paradoxa |  | x |  |  |  |  |  |  |  |
| OD1074_Hemiphlebiidae_Hemiphlebia_mirabilis |  |  |  |  |  |  |  | x |  |
| GEODE7742_Heteragrionidae_Oxystigma_petiolatum |  | x |  |  |  |  |  |  |  |
| OD1126_Heteragrionidae_Dimeragrion_percubitale |  | x |  |  |  |  |  |  |  |
| TM155_Heteragrionidae_Heteragrion_tatama |  | x |  |  |  |  |  |  |  |
| TM404_Heteragrionidae_Heteragrion_erythrogastrum |  | x |  |  |  |  |  |  |  |
| OD1146_Hypolestidae_Hypolestes_sp |  | x |  |  |  |  |  |  |  |
| GEODE17864_Idionychidae_Idionyx_claudia |  |  |  |  |  | x | x |  |  |
| GEODE17870_Idionychidae_Idionyx_optatus |  |  |  |  |  | x | x |  |  |
| RMNH.INS.506617_Idionychidae_Idionyx_philippa |  |  |  |  |  | x | x |  |  |
| GEODE17881_Idomacromiidae_Neocordulia_griphus |  | x |  |  |  |  |  |  |  |
| NESOMASC_Idomacromiidae_Nesocordulia_mascarenica |  |  |  |  | x |  |  |  |  |
| RMNH.5008153_Idomacromiidae_Syncordulia_gracilis |  |  |  | x |  |  |  |  |  |
| RMNH.INS.503184_Idomacromiidae_Idomacromia_proavita |  |  |  | x |  |  |  |  |  |
| RMNH.INS.507576_Idomacromiidae_Oxygastra_curtisii |  |  | x |  |  |  |  |  |  |
| RU139_Idomacromiidae_Neocordulia_volxemi |  | x |  |  |  |  |  |  |  |
| TM41_Idomacromiidae_Neocordulia_batesi |  | x |  |  |  |  |  |  |  |
| GEODE7860_Isostictidae_Austrosticta_soror |  |  |  |  |  |  |  | x |  |
| OD1277_Isostictidae_Neosticta_fraseri |  |  |  |  |  |  |  | x |  |
| OD1298_Isostictidae_Cnemisticta_latilobata |  |  |  |  |  |  |  | x |  |
| OD1312_Isostictidae_Labidiosticta_vallisi |  |  |  |  |  |  |  | x |  |
| OD159_Isostictidae_Isosticta_robustior |  |  |  |  |  |  |  | x |  |
| OD55_Isostictidae_Rhadinosticta_simplex |  |  |  |  |  |  |  | x |  |
| RMNH.5011475_Isostictidae_Oristicta_filicicola |  |  |  |  |  |  |  | x |  |
| RMNH.5011582_Isostictidae_Tanymecosticta_leptalea |  |  |  |  |  |  | x | x |  |
| RMNH.INS.229243_Isostictidae_Selysioneura_capreola |  |  |  |  |  |  | x | x |  |
| GEODE7771_Lauromacromiidae_Lauromacromia_dubitalis |  | x |  |  |  |  |  |  |  |
| RU123_Lauromacromiidae_Lauromacromia_bedei |  | x |  |  |  |  |  |  |  |
| RU126_Lauromacromiidae_Lauromacromia_picinguaba |  | x |  |  |  |  |  |  |  |
| GEODE7665_Lestidae_Indolestes_albicaudus |  |  | x |  |  | x | x | x | x |
| GEODE7728_Lestidae_Austrolestes_leda |  |  |  |  |  |  |  | x | x |
| GEODE7730_Lestidae_Archilestes_grandis | x | x |  |  |  |  |  |  |  |
| GEODE7813_Lestidae_Lestes_plagiatus | x | x | x | x | x | x | x | x | x |
| OD1127_Lestidae_Orolestes_octomaculatus |  |  |  |  |  | x |  |  |  |
| OD122_Lestidae_Sympecma_paedisca |  |  | x |  |  | x |  |  |  |
| OD1278_Lestidae_Indolestes_cyaneus |  |  | x |  |  | x | x | x | x |
| OD1321_Lestidae_Lestes_disjunctus | x | x | x | x | x | x | x | x | x |
| OD63_Lestidae_Austrolestes_annulosus |  |  |  |  |  |  |  | x | x |
| RMNH.INS.507448_Lestidae_Chalcolestes_viridis |  |  | x |  |  |  |  |  |  |
| OD157_Lestoideidae_Lestoidea_conjuncta |  |  |  |  |  |  |  | x |  |
| OD43_Lestoideidae_Diphlebia_coerulescens |  |  |  |  |  |  |  | x |  |
| RMNH.5011424_Lestoideidae_Diphlebia_euphoeoides |  |  |  |  |  |  |  | x |  |
| GEODE12746_Libellulidae_Akrothemis_risi |  |  |  |  |  |  |  | x |  |
| GEODE12747_Libellulidae_Amphithemis_curvistyla |  |  |  |  |  | x |  |  |  |
| GEODE12748_Libellulidae_Celebophlebia_dactylogastra |  |  |  |  |  |  | x |  |  |
| GEODE12749_Libellulidae_Celebothemis_delecollei |  |  |  |  |  |  | x |  |  |
| GEODE17725_Libellulidae_Orthemis_macrostigma | x | x |  |  |  |  |  |  | x |
| GEODE17728_Libellulidae_Orthemis_sulphurata | x | x |  |  |  |  |  |  | x |
| GEODE17755_Libellulidae_Anatya_januaria |  | x |  |  |  |  |  |  |  |
| GEODE17756_Libellulidae_Antidythemis_trameiformis |  | x |  |  |  |  |  |  |  |
| GEODE17757_Libellulidae_Archaeophlebia_martini |  |  |  |  | x |  |  |  |  |
| GEODE17758_Libellulidae_Atoconeura_kenya |  |  |  | x |  |  |  |  |  |
| GEODE17761_Libellulidae_Boninthemis_insularis |  |  |  |  |  |  |  |  | x |
| GEODE17929_Libellulidae_Scapanea_frontalis |  | x |  |  |  |  |  |  |  |
| GEODE7612_Libellulidae_Cannaphila_insularis | x | x |  |  |  |  |  |  |  |
| GEODE7628_Libellulidae_Rhodopygia_hinei |  | x |  |  |  |  |  |  |  |
| GEODE7638_Libellulidae_Nannophlebia_axiagasta |  |  |  |  |  |  | x | x |  |
| GEODE7650_Libellulidae_Diplacina_spec |  |  |  |  |  |  | x | x |  |
| GEODE7655_Libellulidae_Diplacina_persephone |  |  |  |  |  |  | x | x |  |
| GEODE7659_Libellulidae_Microtrigonia_gomphoides |  |  |  |  |  |  |  | x |  |
| GEODE7676_Libellulidae_Misagria_calverti |  | x |  |  |  |  |  |  |  |
| GEODE7698_Libellulidae_Erythemis_credula | x | x |  |  |  |  |  |  |  |
| GEODE7699_Libellulidae_Erythrodiplax_umbrata | x | x |  |  |  |  |  |  |  |
| GEODE7700_Libellulidae_Oligoclada_sp |  | x |  |  |  |  |  |  |  |
| GEODE7701_Libellulidae_Agrionoptera_insignis |  |  | x |  |  | x | x | x | x |
| GEODE7702_Libellulidae_Uracis_fastigiata |  | x |  |  |  |  |  |  |  |
| GEODE7707_Libellulidae_Elga_sp |  | x |  |  |  |  |  |  |  |
| GEODE7708_Libellulidae_Plathemis_subornata | x |  |  |  |  |  |  |  |  |
| GEODE7709_Libellulidae_Rhyothemis_notata |  |  | x | x | x | x | x | x | x |
| GEODE7711_Libellulidae_Celithemis_eponina | x |  |  |  |  |  |  |  |  |
| GEODE7712_Libellulidae_Perithemis_sp | x | x |  |  |  |  |  |  |  |
| GEODE7714_Libellulidae_Brachythemis_leucosticta |  |  | x | x | x | x | x |  |  |
| GEODE7735_Libellulidae_Ladona_julia | x |  |  |  |  |  |  |  |  |
| GEODE7738_Libellulidae_Brechmorhoga_mendax | x | x |  |  |  |  |  |  |  |
| GEODE7739_Libellulidae_Perithemis_tenera | x | x |  |  |  |  |  |  |  |
| GEODE7746_Libellulidae_Paltothemis_lineatipes | x |  |  |  |  |  |  |  |  |
| GEODE7756_Libellulidae_Pachydiplax_longipennis | x |  |  |  |  |  |  |  |  |
| GEODE7759_Libellulidae_Nannophya_australis |  |  | x |  |  | x |  | x | x |
| GEODE7760_Libellulidae_Austrothemis_nigrescens |  |  |  |  |  |  |  | x |  |
| GEODE7761_Libellulidae_Tauriphila_argo | x | x |  |  |  |  |  |  |  |
| GEODE7762_Libellulidae_Macrothemis_nobilis | x | x |  |  |  |  |  |  |  |
| GEODE7764_Libellulidae_Miathyria_marcella | x | x |  |  |  |  |  |  |  |
| GEODE7766_Libellulidae_Zenithoptera_fasciata |  | x |  |  |  |  |  |  |  |
| GEODE7767_Libellulidae_Nephepeltia_phryne |  | x |  |  |  |  |  |  |  |
| GEODE7768_Libellulidae_Elasmothemis_williamsoni |  | x |  |  |  |  |  |  |  |
| GEODE7769_Libellulidae_Argyrothemis_argentea |  | x |  |  |  |  |  |  |  |
| GEODE7770_Libellulidae_Gynothemis_pumilia |  | x |  |  |  |  |  |  |  |
| GEODE7776_Libellulidae_Micrathyria_didyma | x | x |  |  |  |  |  |  |  |
| GEODE7777_Libellulidae_Dythemis_nigra | x | x |  |  |  |  |  |  |  |
| GEODE7798_Libellulidae_Palpopleura_lucia |  |  | x | x | x | x |  |  |  |
| GEODE7800_Libellulidae_Diplacodes_trivialis |  |  | x | x | x | x | x | x | x |
| GEODE7801_Libellulidae_Crocothemis_servilia | x | x | x | x | x | x | x | x | x |
| GEODE7802_Libellulidae_Aethriamanta_rezia |  |  |  | x | x | x | x | x | x |
| GEODE7803_Libellulidae_Diastatops_obscura |  | x |  |  |  |  |  |  |  |
| GEODE7804_Libellulidae_Tetrathemis_fraseri |  |  |  | x | x | x | x | x |  |
| GEODE7805_Libellulidae_Acisoma_trifidum |  |  | x | x | x | x | x |  |  |
| GEODE7806_Libellulidae_Orthetrum_julia |  |  | x | x | x | x | x | x | x |
| GEODE7807_Libellulidae_Tramea_transmarina | x | x | x | x | x | x | x | x | x |
| GEODE7815_Libellulidae_Urothemis_assignata |  |  | x | x | x | x | x | x |  |
| OD107_Libellulidae_Pantala_flavescens | x | x | x | x | x | x | x | x | x |
| OD1091_Libellulidae_Brachythemis_contaminata |  |  | x | x | x | x | x |  |  |
| OD1094_Libellulidae_Nannophlebia_alexia |  |  |  |  |  |  | x | x |  |
| OD1095_Libellulidae_Sympetrum_corruptum | x | x | x | x | x | x |  |  |  |
| OD1106_Libellulidae_Zygonyx_flavicosta |  |  | x | x | x | x | x |  |  |
| OD1319_Libellulidae_Macrodiplax_cora | x | x | x | x | x | x | x | x | x |
| OD77_Libellulidae_Rhyothemis_graphiptera |  |  | x | x | x | x | x | x | x |
| OD82_Libellulidae_Brachydiplax_denticauda |  |  | x |  |  | x | x | x | x |
| RMNH.5007803_Libellulidae_Atoconeura_biordinata |  |  |  | x |  |  |  |  |  |
| RMNH.5008306_Libellulidae_Neurothemis_fluctuans |  |  | x |  |  | x | x | x | x |
| RMNH.5011411_Libellulidae_Notolibellula_bicolor |  |  |  |  |  |  |  | x |  |
| RMNH.5011412_Libellulidae_Microtrigonia_curvata |  |  |  |  |  |  |  | x |  |
| RMNH.5011413_Libellulidae_Bironides_glochidion |  |  |  |  |  |  |  | x |  |
| RMNH.INS.228976_Libellulidae_Tetrathemis_irregularis |  |  |  | x | x | x | x | x |  |
| RMNH.INS.228999_Libellulidae_Raphismia_bispina |  |  |  |  |  | x | x | x |  |
| RMNH.INS.500592_Libellulidae_Huonia_epinephela |  |  |  |  |  |  | x | x |  |
| RMNH.INS.500644_Libellulidae_Protorthemis_coronata |  |  |  |  |  |  | x |  | x |
| RMNH.INS.501123_Libellulidae_Acisoma_panorpoides |  |  | x | x | x | x | x |  |  |
| RMNH.INS.501152_Libellulidae_Pseudothemis_jorina |  |  | x |  |  | x |  |  |  |
| RMNH.INS.501189_Libellulidae_Aethriamanta_brevipennis |  |  |  | x | x | x | x | x | x |
| RMNH.INS.501196_Libellulidae_Rhodothemis_rufa |  |  |  |  |  |  | x | x | x |
| RMNH.INS.501275_Libellulidae_Orchithemis_xanthosoma |  |  |  |  |  | x | x |  |  |
| RMNH.INS.501284_Libellulidae_Brachygonia_oculata |  |  |  |  |  | x | x |  |  |
| RMNH.INS.501296_Libellulidae_Tholymis_tillarga | x | x | x | x | x | x | x | x | x |
| RMNH.INS.501373_Libellulidae_Chalybeothemis_fluviatilis |  |  |  |  |  | x |  |  |  |
| RMNH.INS.501377_Libellulidae_Zyxomma_petiolatum |  |  | x | x | x | x | x | x | x |
| RMNH.INS.501398_Libellulidae_Pornothemis_sp |  |  |  |  |  | x | x |  |  |
| RMNH.INS.501662_Libellulidae_Eleuthemis_buettikoferi |  |  |  | x |  |  |  |  |  |
| RMNH.INS.502121_Libellulidae_Oxythemis_phoenicosceles |  |  |  | x |  |  |  |  |  |
| RMNH.INS.502150_Libellulidae_Chalcostephia_flavifrons |  |  |  | x | x |  |  |  |  |
| RMNH.INS.502177_Libellulidae_Notiothemis_robertsi |  |  |  | x |  |  |  |  |  |
| RMNH.INS.502225_Libellulidae_Aethiothemis_erythromelas |  |  |  | x | x |  |  |  |  |
| RMNH.INS.502285_Libellulidae_Cyanothemis_simpsoni |  |  |  | x |  |  |  |  |  |
| RMNH.INS.502287_Libellulidae_Micromacromia_camerunica |  |  |  | x |  |  |  |  |  |
| RMNH.INS.502304_Libellulidae_Parazyxomma_flavicans |  |  |  | x | x |  |  |  |  |
| RMNH.INS.502305_Libellulidae_Hadrothemis_camarensis |  |  |  | x |  |  |  |  |  |
| RMNH.INS.502397_Libellulidae_Malgassophlebia_bispina |  |  |  | x | x |  |  |  |  |
| RMNH.INS.502400_Libellulidae_Nesciothemis_farinosa |  |  | x | x |  |  |  |  |  |
| RMNH.INS.502847_Libellulidae_Thermochoria_jeanneli |  |  |  | x |  |  |  |  |  |
| RMNH.INS.503416_Libellulidae_Lyriothemis_cleis |  |  | x |  |  | x | x | x |  |
| RMNH.INS.503419_Libellulidae_Nesoxenia_lineata |  |  |  |  |  | x | x | x | x |
| RMNH.INS.503939_Libellulidae_Hydrobasileus_croceus |  |  | x |  |  | x | x | x | x |
| RMNH.INS.503943_Libellulidae_Indothemis_limbata |  |  |  |  |  | x |  |  |  |
| RMNH.INS.503957_Libellulidae_Potamarcha_congener |  |  |  |  |  | x | x | x |  |
| RMNH.INS.503963_Libellulidae_Lathrecista_asiatica |  |  | x |  |  | x | x | x | x |
| RMNH.INS.503964_Libellulidae_Urothemis_signata |  |  | x | x | x | x | x | x |  |
| RMNH.INS.504032_Libellulidae_Onychothemis_culminicola |  |  |  |  |  | x | x |  |  |
| RMNH.INS.504039_Libellulidae_Orchithemis_pulcherrima |  |  |  |  |  | x | x |  |  |
| RMNH.INS.504459_Libellulidae_Hemistigma_albipunctum |  |  |  | x | x |  |  |  |  |
| RMNH.INS.504999_Libellulidae_Orthetrum_cancellatum |  |  | x | x | x | x | x | x | x |
| RMNH.INS.505354_Libellulidae_Trithetrum_navasi |  |  |  | x |  |  |  |  |  |
| RMNH.INS.505362_Libellulidae_Crocothemis_erythraea | x | x | x | x | x | x | x | x | x |
| RMNH.INS.505380_Libellulidae_Olpogastra_lugubris |  |  |  | x | x |  |  |  |  |
| RMNH.INS.505547_Libellulidae_Porpax_risi |  |  |  | x |  |  |  |  |  |
| RMNH.INS.505588_Libellulidae_Diplacodes_lefebvrii |  |  | x | x | x | x | x | x | x |
| RMNH.INS.505652_Libellulidae_Bradinopyga_cornuta |  |  |  | x |  | x |  |  |  |
| RMNH.INS.505880_Libellulidae_Tapeinothemis_boharti |  |  |  |  |  |  |  | x | x |
| RMNH.INS.506254_Libellulidae_Camacinia_gigantea |  |  |  |  |  | x | x | x | x |
| RMNH.INS.506290_Libellulidae_Cratilla_lineata |  |  |  |  |  | x | x | x |  |
| RMNH.INS.507392_Libellulidae_Leucorrhinia_rubicunda | x |  | x |  |  |  |  |  |  |
| RMNH.INS.507400_Libellulidae_Libellula_depressa | x | x | x |  |  | x |  |  |  |
| RMNH.INS.507439_Libellulidae_Libellula_quadrimaculata | x | x | x |  |  | x |  |  |  |
| RMNH.INS.507465_Libellulidae_Sympetrum_danae | x | x | x | x | x | x |  |  |  |
| RMNH.INS.507492_Libellulidae_Nannodiplax_rubra |  |  |  |  |  |  |  | x |  |
| RMNH.INS.507529_Libellulidae_Trithemis_annulata |  |  | x | x | x | x | x | x |  |
| RMNH.INS.507551_Libellulidae_Selysiothemis_nigra |  |  | x |  |  | x |  |  |  |
| RMNH.INS.507746_Libellulidae_Hylaeothemis_clementia |  |  |  |  |  | x |  |  |  |
| RMNH.INS.507831_Libellulidae_Tyriobapta_torrida |  |  |  |  |  | x |  |  |  |
| RMNH.INS.508001_Libellulidae_Zygonyx_torridus |  |  | x | x | x | x | x |  |  |
| RMNH.INS.508447_Libellulidae_Palpopleura_sexmaculata |  |  | x | x | x | x |  |  |  |
| RMNH.INS.508515_Libellulidae_Nannophyopsis_clara |  |  |  |  |  | x |  |  |  |
| RMNH.INS.508797_Libellulidae_Neodythemis_afra |  |  |  | x | x |  |  |  |  |
| RMNH.INS.509795_Libellulidae_Thalassothemis_marchali |  |  |  |  | x |  |  |  |  |
| RMNH.INS.559789_Libellulidae_Zygonoides_fuelleborni |  |  |  | x | x |  |  |  |  |
| TM207_Libellulidae_Brechmorhoga_vivax | x | x |  |  |  |  |  |  |  |
| TM332_Libellulidae_Cannaphila_vibex | x | x |  |  |  |  |  |  |  |
| TM342_Libellulidae_Macrothemis_pseudimitans | x | x |  |  |  |  |  |  |  |
| TM409_Libellulidae_Rhodopygia_hinei |  | x |  |  |  |  |  |  |  |
| TM532_Libellulidae_Erythemis_plebeja | x | x |  |  |  |  |  |  |  |
| GEODE17874_Macromidiidae_Macromidia_genialis |  |  | x |  |  | x | x |  |  |
| GEODE17876_Macromidiidae_Macromidia_kelloggi |  |  | x |  |  | x | x |  |  |
| RMNH.INS.506247_Macromidiidae_Macromidia_fulva |  |  | x |  |  | x | x |  |  |
| GEODE17622_Macromiidae_Macromia_astarte | x |  | x |  |  | x | x | x |  |
| GEODE7715_Macromiidae_Phyllomacromia_picta |  |  |  | x | x |  |  |  |  |
| OD1101_Macromiidae_Epophthalmia_elegans |  |  | x |  |  | x | x |  |  |
| RMNH.INS.507575_Macromiidae_Macromia_splendens | x |  | x |  |  | x | x | x |  |
| GEODE7863_Megapodagrionidae_Allopodagrion_contortum |  | x |  |  |  |  |  |  |  |
| OD1281_Megapodagrionidae_Teinopodagrion_meridionale |  | x |  |  |  |  |  |  |  |
| OD1183_Mesagrionidae_Mesagrion_leucorrhinum |  | x |  |  |  |  |  |  |  |
| OD1077_Mesopodagrionidae_Mesopodagrion_sp |  |  |  |  |  | x |  |  |  |
| OD1113_Neopetaliidae_Neopetalia_punctata |  | x |  |  |  |  |  |  |  |
| RMNH.INS.502324_Neophyidae_Neophya_rutherfordi |  |  |  | x |  |  |  |  |  |
| OD1139_Pentaphlebiidae_Pentaphlebia_mangana |  |  |  | x |  |  |  |  |  |
| RMNH.INS.500093_Pentaphlebiidae_Pentaphlebia_stahli |  |  |  | x |  |  |  |  |  |
| OD1120_Perilestidae_Perissolestes_remotus |  | x |  |  |  |  |  |  |  |
| GEODE17940_Petaluridae_Petalura_litorea |  |  |  |  |  |  |  | x |  |
| GEODE17943_Petaluridae_Uropetala_chiltoni |  |  |  |  |  |  |  | x | x |
| GEODE7743_Petaluridae_Tanypteryx_hageni | x |  | x |  |  |  |  |  |  |
| OD1318_Petaluridae_Petalura_gigantea |  |  |  |  |  |  |  | x |  |
| PD166_Petaluridae_Tanypteryx_pryeri | x |  | x |  |  |  |  |  |  |
| OD1079_Philogangidae_Philoganga_vetusta |  |  |  |  |  | x |  |  |  |
| OD1124_Philogeniidae_Philogenia_carrillica |  | x |  |  |  |  |  |  |  |
| TM201_Philogeniidae_Philogenia_martae |  | x |  |  |  |  |  |  |  |
| OD1119_Philosinidae_Rhinagrion_sp |  |  |  |  |  | x | x |  |  |
| OD1130_Philosinidae_Philosina_buchi |  |  |  |  |  | x |  |  |  |
| RMNH.INS.506835_Philosinidae_Rhinagrion_macrocephalum |  |  |  |  |  | x | x |  |  |
| GEODE2652_Platycnemididae_Asthenocnemis_stephanodera |  |  |  |  |  |  | x |  |  |
| GEODE7624_Platycnemididae_Disparoneura_apicalis |  |  |  |  |  | x |  |  |  |
| GEODE7653_Platycnemididae_Esme_mudiensis |  |  |  |  |  | x |  |  |  |
| GEODE7656_Platycnemididae_Nososticta_selysii |  |  |  |  |  | x | x | x |  |
| GEODE7660_Platycnemididae_Nososticta_exul |  |  |  |  |  | x | x | x |  |
| GEODE7668_Platycnemididae_Calicnemia_pulverulans |  |  |  |  |  | x |  |  |  |
| GEODE7673_Platycnemididae_Calicnemia_erythromelas |  |  |  |  |  | x |  |  |  |
| GEODE7724_Platycnemididae_Coeliccia_cyanomelas |  |  | x |  |  | x | x |  |  |
| GEODE7727_Platycnemididae_Prodasineura_autumnalis |  |  |  |  |  | x | x |  |  |
| GEODE7750_Platycnemididae_Copera_nyansana |  |  |  | x |  | x | x |  |  |
| GEODE7796_Platycnemididae_Elattoneura_acuta |  |  |  | x |  | x |  |  |  |
| GEODE7811_Platycnemididae_Copera_marginipes |  |  |  | x |  | x | x |  |  |
| GEODE7864_Platycnemididae_Hylaeargia_magnifica |  |  |  |  |  |  |  | x |  |
| GEODE7866_Platycnemididae_Lieftinckia_malaitae |  |  |  |  |  |  |  | x | x |
| GEODE7868_Platycnemididae_Rhyacocnemis_prothoracica |  |  |  |  |  |  |  | x |  |
| OD1122_Platycnemididae_Prodasineura_croconota |  |  |  |  |  | x | x |  |  |
| OD1123_Platycnemididae_Coeliccia_pyriformis |  |  | x |  |  | x | x |  |  |
| OD1132_Platycnemididae_Onychargia_atrocyana |  |  |  |  |  | x | x |  |  |
| OD1143_Platycnemididae_Mesocnemis_singularis |  |  | x | x |  |  |  |  |  |
| OD1306_Platycnemididae_Elattoneura_glauca |  |  |  | x |  | x |  |  |  |
| OD1317_Platycnemididae_Nososticta_solida |  |  |  |  |  | x | x | x |  |
| OD148_Platycnemididae_Platycnemis_pennipes |  |  | x |  |  | x |  |  |  |
| RMNH.5007870_Platycnemididae_Metacnemis_valida |  |  |  | x |  |  |  |  |  |
| RMNH.5011406_Platycnemididae_Macrocnemis_gracilis |  |  |  |  |  |  |  | x |  |
| RMNH.INS.229282_Platycnemididae_Paracnemis_alluaudi |  |  |  |  | x |  |  |  |  |
| RMNH.INS.229291_Platycnemididae_Torrenticnemis_filicornis |  |  |  |  |  |  |  | x |  |
| RMNH.INS.500162_Platycnemididae_Allocnemis_contraria |  |  |  | x |  |  |  |  |  |
| RMNH.INS.500513_Platycnemididae_Lochmaeocnemis_malacodora |  |  |  |  |  |  |  | x |  |
| RMNH.INS.500580_Platycnemididae_Palaiargia_charmosyna |  |  |  |  |  |  | x | x |  |
| RMNH.INS.500639_Platycnemididae_Idiocnemis_obliterata |  |  |  |  |  |  |  | x |  |
| RMNH.INS.501977_Platycnemididae_Arrhenocnemis_parvibullis |  |  |  |  |  |  |  | x |  |
| RMNH.INS.501995_Platycnemididae_Cyanocnemis_aureofrons |  |  |  |  |  |  |  | x |  |
| RMNH.INS.503929_Platycnemididae_Calicnemia_chaseni |  |  |  |  |  | x |  |  |  |
| RMNH.INS.503944_Platycnemididae_Pseudocopera_ciliata |  |  | x |  |  | x |  |  |  |
| RMNH.INS.505846_Platycnemididae_Arabicnemis_caerulea |  |  | x |  |  |  |  |  |  |
| RMNH.INS.506232_Platycnemididae_Matticnemis_doi |  |  |  |  |  | x |  |  |  |
| RMNH.INS.508201_Platycnemididae_Arabineura_khalidi |  |  | x |  |  |  |  |  |  |
| RMNH.INS.508637_Platycnemididae_Indocnemis_orang |  |  |  |  |  | x |  |  |  |
| RMNH.INS.508794_Platycnemididae_Stenocnemis_pachystigma |  |  |  | x |  |  |  |  |  |
| RMNH.INS.559644_Platycnemididae_Spesbona_angusta |  |  |  | x |  |  |  |  |  |
| GEODE7722_Platystictidae_Protosticta_satoi |  |  |  |  |  | x | x |  |  |
| GEODE7787_Platystictidae_Palaemnema_brevignoni | x | x |  |  |  |  |  |  |  |
| GEODE7871_Platystictidae_Sinosticta_sylvatica |  |  |  |  |  | x |  |  |  |
| GEODE7876_Platystictidae_Ceylonosticta_lankanensis |  |  |  |  |  | x |  |  |  |
| RMNH.5008397_Platystictidae_Telosticta_ulubaram |  |  |  |  |  | x | x |  |  |
| RMNH.INS.506261_Platystictidae_Telosticta_bidayuh |  |  |  |  |  | x | x |  |  |
| RMNH.INS.506492_Platystictidae_Sinosticta_ogatai |  |  |  |  |  | x |  |  |  |
| RMNH.INS.507659_Platystictidae_Drepanosticta_actaeon |  |  |  |  |  | x | x | x | x |
| ANCH188_Polythoridae_Miocora_peraltica |  | x |  |  |  |  |  |  |  |
| BOY03_Polythoridae_Cora_xanthostoma |  | x |  |  |  |  |  |  |  |
| PP330_Polythoridae_Polythore_procera |  | x |  |  |  |  |  |  |  |
| RMNH.INS.505717_Polythoridae_Chalcopteryx_rutilans |  | x |  |  |  |  |  |  |  |
| TM154_Polythoridae_Polythore_gigantea |  | x |  |  |  |  |  |  |  |
| UAE1_Polythoridae_Euthore_fassli |  | x |  |  |  |  |  |  |  |
| GEODE7872_Priscagrionidae_Sinocnemis_henanensis |  |  |  |  |  | x |  |  |  |
| OD1280_Priscagrionidae_Priscagrion_kiautai |  |  |  |  |  | x |  |  |  |
| OD1136_Protolestidae_Protolestes_fickei |  |  |  |  | x |  |  |  |  |
| OD1308_Protolestidae_Protolestes_kerckhoffae |  |  |  |  | x |  |  |  |  |
| GEODE17883_Pseudocorduliidae_Pseudocordulia_circularis |  |  |  |  |  |  |  | x |  |
| GEODE17884_Pseudocorduliidae_Pseudocordulia_elliptica |  |  |  |  |  |  |  | x |  |
| OD1283_Pseudolestidae_Pseudolestes_mirabilis |  |  |  |  |  | x |  |  |  |
| OD1085_Rhipidolestidae_Agriomorpha_xinglongensis |  |  |  |  |  | x |  |  |  |
| OD1121_Rhipidolestidae_Agriomorpha_fusca |  |  |  |  |  | x |  |  |  |
| OD96_Rhipidolestidae_Rhipidolestes_aculeatus |  |  | x |  |  | x |  |  |  |
| RMNH.INS.502064_Rhipidolestidae_Rhipidolestes_truncatidens |  |  | x |  |  | x |  |  |  |
| GEODE7704_Rimanellidae_Rimanella_arcana |  | x |  |  |  |  |  |  |  |
| GEODE7723_Synlestidae_Chlorolestes_elegans |  |  |  | x |  |  |  |  |  |
| GEODE7755_Synlestidae_Episynlestes_albicauda |  |  |  |  |  |  |  | x |  |
| GEODE7873_Synlestidae_Phylolestes_ethelae |  | x |  |  |  |  |  |  |  |
| OD1305_Synlestidae_Chlorolestes_umbratus |  |  |  | x |  |  |  |  |  |
| OD81_Synlestidae_Synlestes_weyersii |  |  |  |  |  |  |  | x |  |
| RMNH.5007835_Synlestidae_Ecchlorolestes_nylephtha |  |  |  | x |  |  |  |  |  |
| RMNH.5011485_Synlestidae_Chorismagrion_risi |  |  |  |  |  |  |  | x |  |
| RMNH.INS.508632_Synlestidae_Megalestes_heros |  |  |  |  |  | x |  |  |  |
| RMNH.INS.508746_Synlestidae_Nubiolestes_diotima |  |  |  | x |  |  |  |  |  |
| GEODE17885_Synthemistidae_Archaeosynthemis_leachii |  |  |  |  |  |  |  | x |  |
| GEODE17887_Synthemistidae_Archaeosynthemis_orientalis |  |  |  |  |  |  |  | x |  |
| GEODE17888_Synthemistidae_Archaeosynthemis_spiniger |  |  |  |  |  |  |  | x |  |
| GEODE17889_Synthemistidae_Austrosynthemis_cyanitincta |  |  |  |  |  |  |  | x |  |
| GEODE17891_Synthemistidae_Eusynthemis_guttata |  |  |  |  |  |  |  | x |  |
| GEODE17896_Synthemistidae_Calesynthemis_campioni |  |  |  |  |  |  |  | x |  |
| GEODE17901_Synthemistidae_Synthemis_tasmanica |  |  |  |  |  |  |  | x |  |
| GEODE17902_Synthemistidae_Tonyosynthemis_ofarrelli |  |  |  |  |  |  |  | x |  |
| GEODE7643_Synthemistidae_Palaeosynthemis_gracilenta |  |  |  |  |  |  | x | x |  |
| GEODE7680_Synthemistidae_Palaeosynthemis_cervula |  |  |  |  |  |  | x | x |  |
| GEODE7737_Synthemistidae_Choristhemis_flavoterminata |  |  |  |  |  |  |  | x |  |
| GEODE7792_Synthemistidae_Eusynthemis_nigra |  |  |  |  |  |  |  | x |  |
| OD1311_Synthemistidae_Parasynthemis_regina |  |  |  |  |  |  |  | x |  |
| RMNH.5011432_Synthemistidae_Tonyosynthemis_claviculata |  |  |  |  |  |  |  | x |  |
| RMNH.INS.500525_Synthemistidae_Palaeosynthemis_primigenia |  |  |  |  |  |  | x | x |  |
| RMNH.INS.504910_Synthemistidae_Synthemis_eustalacta |  |  |  |  |  |  |  | x |  |
| OD1137_Tatocnemididae_Tatocnemis_malgassica |  |  |  |  | x |  |  |  |  |
| GEODE7874_Thaumatoneuridae_Paraphlebia_duodecima |  | x |  |  |  |  |  |  |  |
| OD1135_Thaumatoneuridae_Paraphlebia_sp |  | x |  |  |  |  |  |  |  |
| OD1141_Thaumatoneuridae_Thaumatoneura_inopinata |  | x |  |  |  |  |  |  |  |
