# Supplementary material for: Soaring Systematics: An Evaluation of Biogeography and Flight Behavior in Dragonflies and Damselflies (Insecta: Odonata) Using Phylogenomics
Source: Syst Biol. 2026 Jan 22;75(5):984–1002. doi: 10.1093/sysbio/syag005 (PMC13445619; doi:10.1093/sysbio/syag005)
Supplement: Supplementary file 1 [file syag005_supplemental_files.zip › Supplemental Table 6 - Divergence time estimates, 95% HPD CI, and Geological Period.docx]

| **CrownNodes** | **Age (Ma)** | **95% HPD CI** | **Geological Time Period** |
| --- | --- | --- | --- |
| Paleoptera | 316.8 | (292.59, 331.78) | Carboniferous |
| Odonata | 313.73 | (290.78, 325.19) | Carboniferous |
| Zygoptera | 278.35 | (233.23, 319.18) | Permian |
| "Calopterygoidea"+Coenagrionoidea | 212.98 | (173.53, 244.67) | Triassic |
| Coenagrionoidea | 160.53 | (127.24, 188.54) | Jurassic |
| Lestoidea | 149.15 | (73.23, 231.38) | Jurassic |
| Argiolestidae | 84.78 | (63.64, 104.84) | Cretaceous |
| Calopterygidae | 96.46 | (70.51, 116.61) | Cretaceous |
| Chlorocyphidae | 91.84 | (70.14, 112.96) | Cretaceous |
| Coenagrionidae | 122.6 | (81.42, 143.29) | Cretaceous |
| Devadattidae | 19.52 | (6.36, 41.23) | Miocene |
| Dicteriadidae | 32.58 | (14.70, 50.24) | Oligocene |
| Euphaeidae | 59.77 | (43.26, 80.06) | Palaeocene |
| Heteragrionidae | 35.07 | (17.53, 57.84) | Oligocene |
| Isostictidae | 53.14 | (29.25, 87.43) | Palaeocene |
| Lestidae | 89.25 | (54.93, 148.33) | Cretaceous |
| Lestoideidae | 39.17 | (18.84, 65.96) | Eocene |
| Megapodagrionidae | 27.88 | (7.09, 55.81) | Oligocene |
| Pentaphlebiidae | 26.21 | (8.68, 45.40) | Oligocene |
| Philogeniidae | 22.64 | (7.64, 42.60) | Miocene |
| Philosinidae | 34.69 | (12.55, 62.44) | Eocene |
| Platycnemididae | 106.63 | (84.49, 132.32) | Cretaceous |
| Platystictidae | 133.61 | (48.93, 228.96) | Cretaceous |
| Platystictoidea | 133.61 | (48.93, 228.96) | Cretaceous |
| Polythoridae | 48.88 | (31.86, 81.42) | Eocene |
| Priscagrionidae | 38.6 | (7.48, 90.72) | Eocene |
| Protolestidae | 28.43 | (9.01, 52.70) | Eocene |
| Rhipidolestidae | 40.62 | (24.29, 66.34) | Eocene |
| Synlestidae | 76.77 | (41.41, 130.72) | Cretaceous |
| Thaumatoneuridae | 26.6 | (13.66, 44.54) | Oligocene |
| Epiprocta | 290.08 | (259.83, 312.93) | Permian |
| Epiophlebiidae | 32.95 | (6.35, 79.93) | Oligocene |
| Anisoptera | 274.07 | (244.76, 299.84) | Permian |
| Aeschnosomiidae | 17.12 | (5.96, 37.16) | Miocene |
| Aeshnidae | 164.75 | (113.45, 225.06) | Jurassic |
| Aeshnoidea | 229.82 | (163.04, 282.77) | Triassic |
| Austrocorduliidae | 91.35 | (34.75, 183.86) | Cretaceous |
| Austropetaliidae | 59.95 | (15.15, 145.87) | Palaeocene |
| Chlorogomphidae | 52.79 | (18.88, 111.62) | Palaeocene |
| Cordulegastridae | 43.37 | (23.30, 69.72) | Eocene |
| Cordulegastroidea | 137.25 | (89.66, 196.70) | Cretaceous |
| Corduliidae | 112.87 | (81.93, 163.87) | Cretaceous |
| Gomphoidea | 233.99 | (214.51, 256.32) | Triassic |
| Gomphomacromiidae | 69.77 | (13.14, 144.68) | Cretaceous |
| Idionychidae | 43.89 | (14.86, 77.24) | Eocene |
| Idomacromiidae | 73.68 | (34.64, 117.92) | Cretaceous |
| Lauromacromiidae | 51.3 | (15.76, 98.64) | Eocene |
| Libellulidae | 106.96 | (78.82, 142.27) | Cretaceous |
| Libelluloidea | 220.3 | (185.96, 251.63) | Triassic |
| Macromidiaiidae | 45.84 | (14.62, 88.40) | Eocene |
| Macromiidae | 39.64 | (18.82, 59.19) | Oligocene |
| Petaluroidea | 152.8 | (114.93, 205.79) | Cretaceous |
| Pseudocorduliidae | 25.47 | (6.84, 54.02) | Miocene |
| Synthemistidae | 116.51 | (69.24, 156.75) | Cretaceous |
|  |  |  |  |
| **StemNodes** | **Age (Ma)** | **95% HPD CI** | **Geological Time Period** |
| Amanipodagrionidae+Mesopodagrionidae | 45.75 | (10.94, 97.54) | Eocene |
| Amphipterygidae+Devadattidae | 29.47 | (10.43, 51.24) | Miocene |
| Hypolestidae+Dicteriadidae | 18.25 | (7.52, 32.00) | Miocene |
| Mesagrionidae+(Hypolestidae+Dicteriadidae) | 48.8 | (23.42, 80.20) | Eocene |
| Philogangidae+Philosinidae | 48.64 | (20.57, 81.30) | Eocene |
| Pseudolestidae+(Lestoideidae+Euphaeidae) | 84.22 | (67.37, 100.33) | Cretaceous |
| Rimanellidae+Polythoridae | 94.38 | (56.44, 141.34) | Cretaceous |
| Tatocnemididae+(Pentaphlebiidae+Protolestidae) | 53.65 | (23.96, 85.94) | Eocene |
| Neopetaliidae+Cordulegastridae | 85.75 | (35.82, 143.33) | Cretaceous |
| Hemiphlebiidae+((Perilestidae+Synlestidae)+Lestidae) | 149.15 | (73.23, 231.38) | Jurassic |
| Perilestidae+Synlestidae | 91.59 | (49.03, 146.13) | Cretaceous |
| Neophyidae+((Libellulidae+Aeschnosomiidae)+Corduliidae) | 143.6 | (125.77, 181.59) | Jurassic |
