# Supplementary material for: Soaring Systematics: An Evaluation of Biogeography and Flight Behavior in Dragonflies and Damselflies (Insecta: Odonata) Using Phylogenomics
Source: Syst Biol. 2026 Jan 22;75(5):984–1002. doi: 10.1093/sysbio/syag005 (PMC13445619; doi:10.1093/sysbio/syag005)
Supplement: Supplementary file 1 [file syag005_supplemental_files.zip › Supplemental Table 7 - Vicariance & Dispersal Events (Full Version).docx]

| **Lineage** | **Vicariance Events** | **Dispersal Events** | | | | | | | **Ratio Vicariance / Dispersal** |
| --- | --- | --- | --- | --- | --- | --- | --- | --- | --- |
|  |  |  | | | | | | |  |
|  |  | **1-4** | **5** | **6** | **7** | **8** | **9** | **Total** |  |
| **Zygoptera** | **34** | **68** | **2** | **-** | **-** | **-** | **2** | **72** | **32/68** |
| ***Lestoidea*** | **5** | **2** | **1** | - | - | - | **2** | **5** | **50/50** |
| Hemiphlebidae | - | - | - | - | - | - | - | **-** |  |
| Lestidae | 1 | 1 | 1 |  |  |  | 2 | **4** | **20/80** |
| Perilestidae | - | - | - | - | - | - | - | **-** |  |
| Synlestidae | 3 | 3 | - | - | - | - | - | **3** | **50/50** |
| ***Platystictoidea*** | 1 | 3 | - | - | - | - | - | **3** | **25/75** |
| Platystictictidae | 1 | 3 | - | - | - | - | - | **3** | **25/75** |
| ***"Calopterygoidea"*** | **18** | **15** | - | - | - | - | - | **15** | **55/45** |
| ***Group 1*** | - | - | - | - | - | - | - | **-** |  |
| Priscagrionidae | - | - | - | - | - | - | - | **-** |  |
| ***Group 2*** | **2** | **2** | - | - | - | - | - | **2** | **50/50** |
| Tatocnemididae | - | - | - | - | - | - | - | **-** |  |
| Pentaphlebidae | - | - | - | - | - | - | - | **-** |  |
| Protolestidae | - | - | - | - | - | - | - | **-** |  |
| Rimanellidae | - | - | - | - | - | - | - | **-** |  |
| Polythoridae | - | - | - | - | - | - | - | **-** |  |
| Mesagrionidae | - | - | - | - | - | - | - | **-** |  |
| Dicteriadidae | - | - | - | - | - | - | - | **-** |  |
| Hypolestidae | - | - | - | - | - | - | - | **-** |  |
| Philogeniidae | - | - | - | - | - | - | - | **-** |  |
| ***Group 3*** | **7** | **10** | - | - | - | - | - | **10** | **41/59** |
| Aminopodagrionidae | - | - | - | - | - | - | - | **-** |  |
| Mesopodagrionidae | - | - | - | - | - | - | - | - |  |
| Amphipterygidae | 1 | - | - | - | - | - | - | - |  |
| Devadattidae | - | - | - | - | - | - | - | **-** |  |
| Thaumatoneuridae | - | - | - | - | - | - | - | **-** |  |
| Rhipolestidae | - | 1 | - | - | - | - | - | **1** |  |
| Lestoideidae | - | - | - | - | - | - | - | **-** |  |
| Euphaeidae | 2 | 6 | - | - | - | - | - | **6** | **25/75** |
| ***Group 4*** | **9** | **26** | - | - | - | - | - | **26** | **26/74** |
| Philogangidae | - | - | - | - | - | - | - | **-** |  |
| Philosinidae | - | 1 | - | - | - | - | - | **1** |  |
| Megapodagrionidae | - | - | - | - | - | - | - | **-** |  |
| Argiolestidae | 5 | 5 | - | - | - | - | - | **5** | **50/50** |
| Chlorophycidae | 1 | 5 | - | - | - | - | - | **5** | **17/83** |
| Calopterygidae | 2 | 11 | - | - | - | - | - | **11** | **15/85** |
| ***Coenagrionoidea*** | **12** | **48** | **1** | - | - | - | - | **49** | **20/80** |
| Isostictidae | - | 2 | 1 | - | - | - | - | **3** |  |
| Platycnecmidae | 4 | 19 | - | - | - | - | - | **19** | **17/83** |
| Coenagrionidae | 8 | 26 | - | - | - | - | - | **26** | **24/76** |
| **Epiprocta** | **43** | 189 | 18 | 8 | 8 | 1 | 4 | **228** | **16/84** |
| ***Epiophlebioidea*** | - | 1 | - | - | - | - | - | **1** |  |
| Epiophlebidae | - | 1 | - | - | - | - | - | **1** |  |
| ***Aeshnoidea*** | **5** | **12** | **3** | **1** | **1** | **-** | **2** | **19** | **21/79** |
| Austropetalidae | 1 |  | - | - | - | - | - | **0** | **100/0** |
| Aeshnidae | 4 | 10 | 3 | 1 | 1 | - | 2 | **17** | **19/81** |
| ***Petaluroidea*** | **1** | **2** | - | - | - | - | - | **2** | **33/67** |
| Petaluridae | 1 | 2 | - | - | - | - | - | **2** | **33/67** |
| ***Gomphoidea*** | **11** | **49** | **-** | **-** | **1** | **-** | - | **50** | **18/82** |
| Gomphidae | 11 | 49 | - | - | 1 | - | - | **50** | **18/82** |
| ***Cordulegastroidea*** | - | **5** | - | - | - | - | - | **5** |  |
| Chlorogomphidae | - | 1 | - | - | - | - | - | **1** |  |
| Cordulegastridae | - | 2 | - | - | - | - | - | **2** |  |
| Neopetalidae | - | - | - | - | - | - | - | **0** |  |
| ***Libelluloidea*** | **26** | **120** | **15** | **7** | **6** | **1** | **2** | **151** | **15/85** |
| Pseduocordulidae | - |  |  |  |  |  |  | **0** |  |
| Gomphomacromiidae | - |  |  |  |  |  |  | **0** |  |
| Synthemistidae | 4 | 1 |  |  |  |  |  | **1** | **80/20** |
| Austrocordulidae | - |  |  |  |  |  |  | **0** |  |
| Idionychidae | - | 1 |  |  |  |  |  | **1** |  |
| Macromiidae | 1 | 1 | 2 |  |  |  |  | **3** | **25/75** |
| Idomacromiidae | 4 | 3 |  |  |  |  |  | **3** | **57/43** |
| Lauromacromiidae | - |  |  |  |  |  |  | **0** |  |
| Macromidiidae | - |  |  |  |  |  |  | **0** |  |
| Neophyidea | - |  |  |  |  |  |  | **0** |  |
| Corduliidae | 3 | 10 |  |  | 2 |  |  | **12** | **20/80** |
| Aeshnosomatidae | 1 | 1 |  |  |  |  |  | **1** | **50/50** |
| Libellulidae | 13 | 103 | 13 | 7 | 4 | 1 | 2 | **130** | **9/91** |
