# Supplementary material for: Soaring Systematics: An Evaluation of Biogeography and Flight Behavior in Dragonflies and Damselflies (Insecta: Odonata) Using Phylogenomics
Source: Syst Biol. 2026 Jan 22;75(5):984–1002. doi: 10.1093/sysbio/syag005 (PMC13445619; doi:10.1093/sysbio/syag005)
Supplement: Supplementary file 1 [file syag005_supplemental_files.zip › Supplemental_Material.pdf]

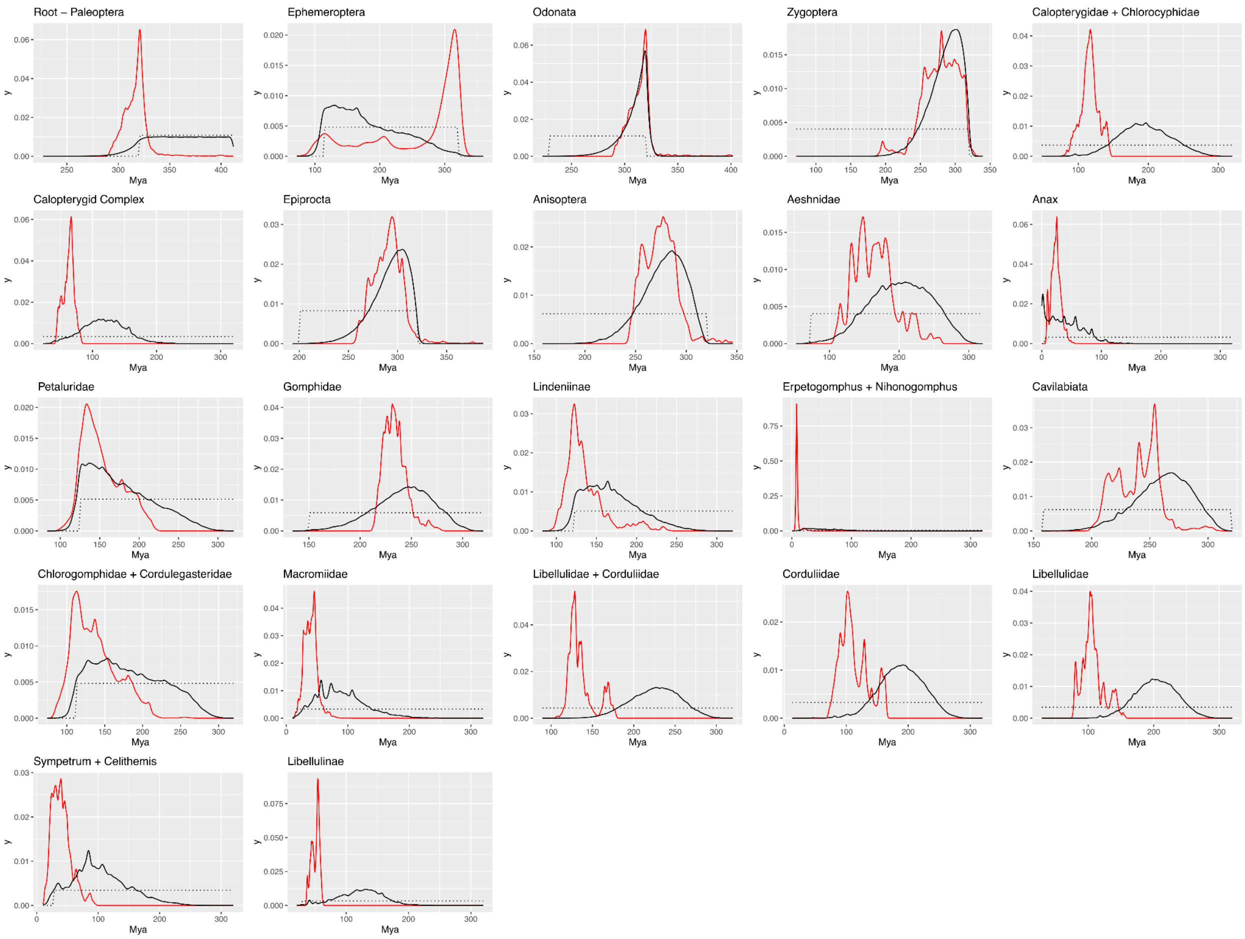

Supplemental Figure 1. Visualization of the specified and effective priors and posteriors in the divergence time estimation analysis. The dotted black line depicts the specified prior, the solid black line depicts the effective prior, and the solid red line depicts the posterior.

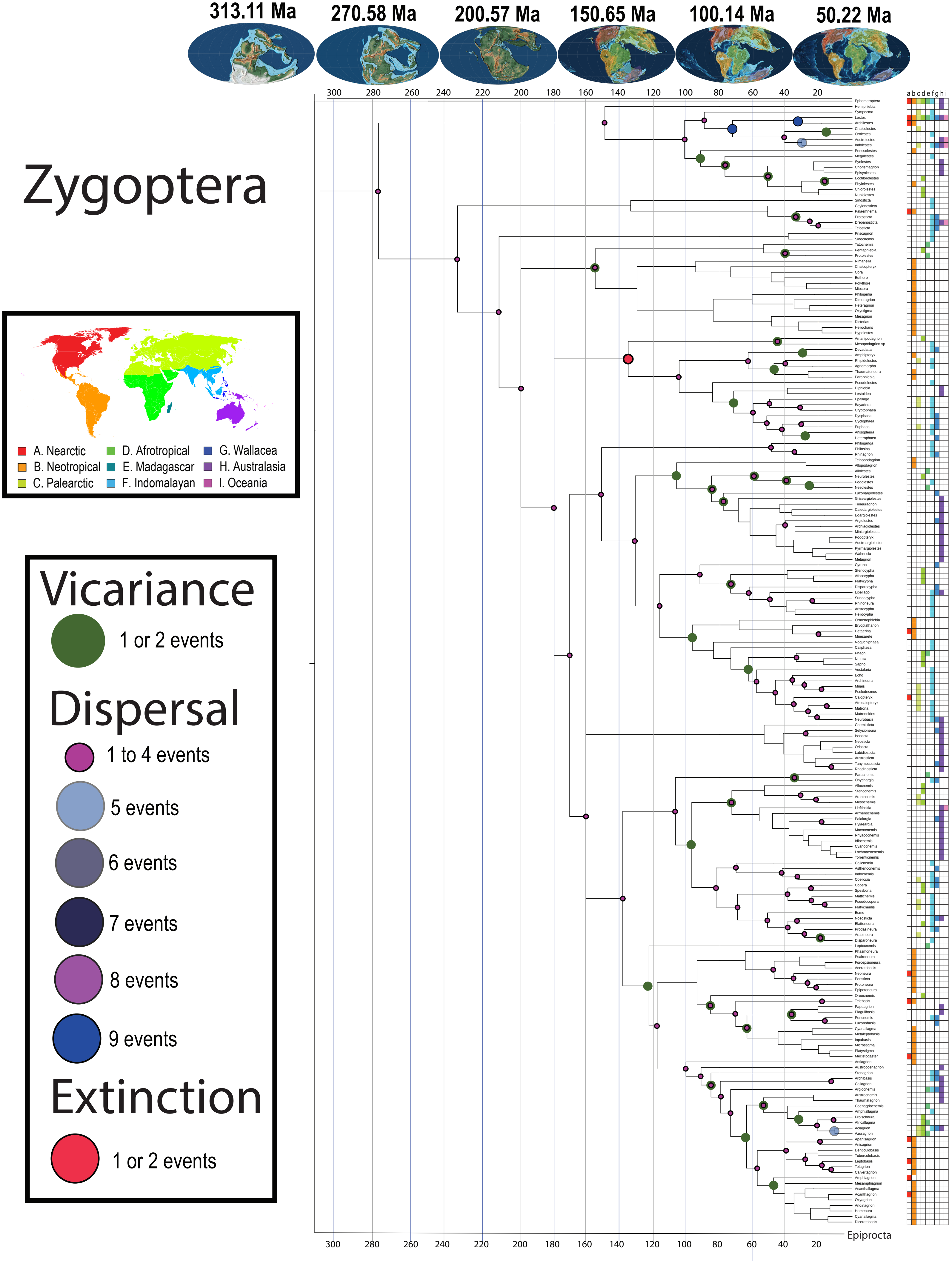

313.11 Ma
270.58 Ma
200.57 Ma
150.65 Ma
100.14 Ma
50.22 Ma
300 280 260 240 220 200 180 160 140 120 100 80 60 40 20
Zygoptera
A. Nearctic
B. Neotropical
C. Palearctic
D. Afrotropical
E. Madagascar
F. Indomalayan
G. Wallacea
H. Australasia
I. Oceania
Vicariance
1 or 2 events
Dispersal
1 to 4 events
5 events
6 events
7 events
8 events
9 events
Extinction
1 or 2 events
a b c d e f g h i
Epiprocta

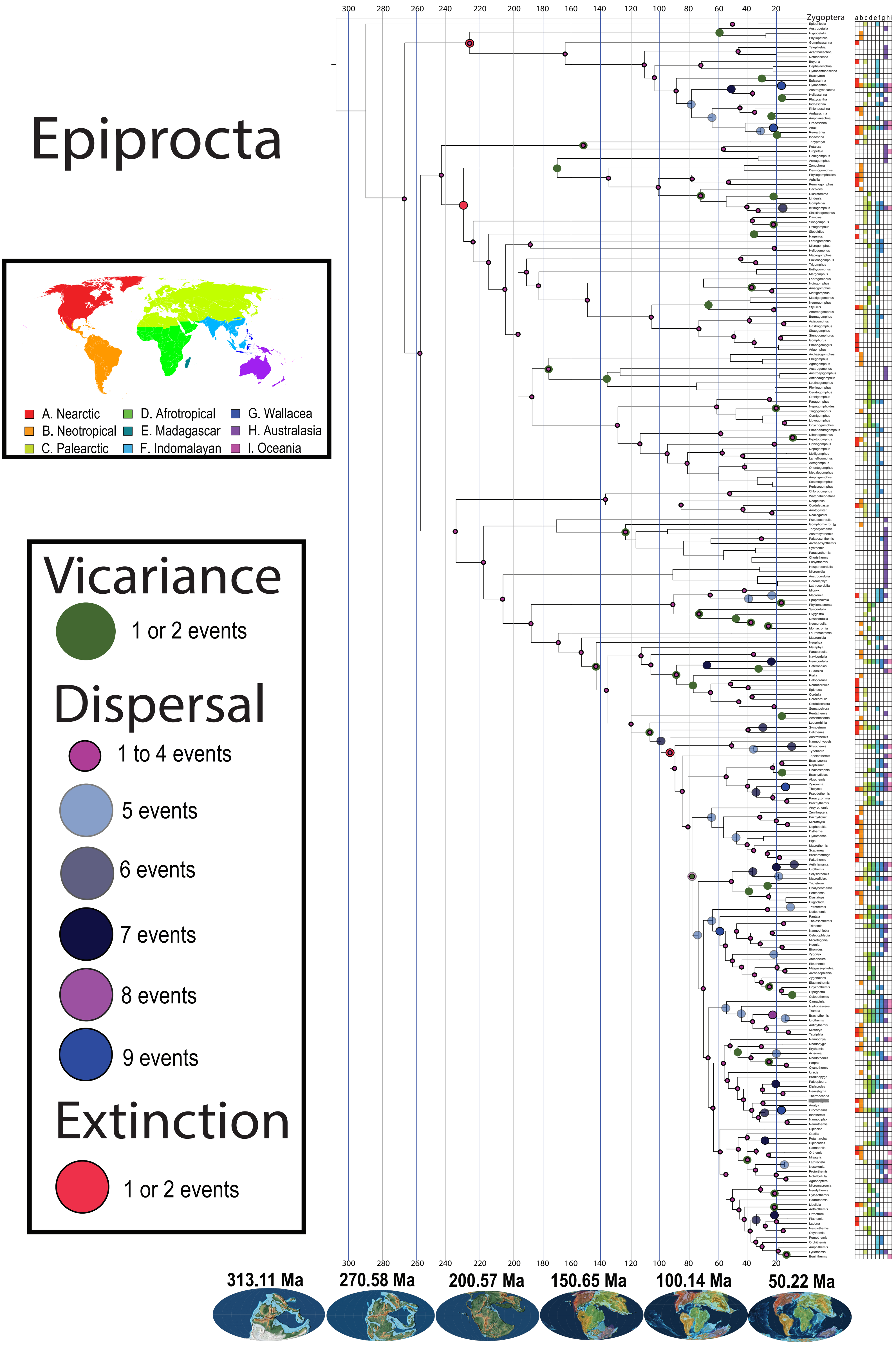
Epiprocta
Zygoptera
a b c d e f g h i
300 280 260 240 220 200 180 160 140 120 100 80 60 40 20
A. Nearctic
B. Neotropical
C. Palearctic
D. Afrotropical
E. Madagascar
F. Indomalayan
G. Wallacea
H. Australasia
I. Oceania
Vicariance
1 or 2 events
Dispersal
1 to 4 events
5 events
6 events
7 events
8 events
9 events
Extinction
1 or 2 events
313.11 Ma
270.58 Ma
200.57 Ma
150.65 Ma
100.14 Ma
50.22 Ma

Supplemental Figure 2. Time-calibrated phylogeny of **Zygoptera (2a)** and **Epiprocta (2b)** with nodes annotated for dispersal, vicariance, and extinction events, shown in temporal context alongside paleogeographic reconstructions PALEOMAPS in GPlates 2.4 (Müller et al., 2024). Dispersal frequency is indicated by contrasting purple/blue hues depending on the number of events. Vicariance events (1–2) are shown in green, and extinction events (1–2) in red.

Sample	Date (yyyy-mm-dd)	Collector	Country	Locality (if Coordinates, separate with a ";")	Other details (notes, other)
RMNH.INS.501973_Argiolestidae_Argiolestes_muller	2011		Papua New Guinea		
RMNH.INS.503407_Argiolestidae_Caledopteryx_sarasini	2010-10-12		New Caledonia	Kompwara R. Below, Refuge Blaffart; -20.624, 164.782	
RMNH.INS.507519_Gomphidae_Ophiogomphus_cecilia	2009-08-04		Netherlands	Paarlo; 351, 200	
RMNH.INS.508536_Cordulegastridae_Anotogaster_sieboldii	2010-09-28		China	Guangdong, Shaoguan City, Nanling National reserve;	
RMNH.INS.508545_Gomphidae_Trigomphus_beatus	2011-04-15		China	Guangdong, Conghua City, Weidong;	
RMNH.INS.508624_Gomphidae_Fukienogomphus_promineus	2010-06-05		China	Guangdong, Huizhou City, Nankunshan;	
GEODE17901_Synthemistidae_Synthemis_tasmanica	1954-01-24	Angel, S.	Australia	Tasmania Lake Dobson 3400ft	
TM155_Heteragrionidae_Heteragrion_tatama	2016-06-29	Ayala, D., A. Orejuela & C. Bota	Colombia	Parque Nacional Natural Tatamá, Vereda Monte Bello	
GEODE7876_Platystictidae_Ceylonosticta_lankanensis	2009-05-21	Bedjanic, M.	Sri Lanka	Sri Lanka, Kegalla district, small stream in secondary forest 1 km S of bridge near Plantation Hotel in Kithulgala, loc. 52, 430 m asl., 6°59.063'N 80°24.821'E, 21 May 2009, leg. M. Bedjanic; 6°59.063'N 80°24.821'E,	
GEODE7681_Gomphidae_Phyllogomphoides_undulatus	1961-10-28	Belle, J.	Suriname	Para River (upper part)	RMNH.ins.516366

GEODE7682_Aeschnosomatidae_Aeschnosoma_forcipula	1959-01-31	Belle, J.	Suriname	Suriname, Zanderij, Pontjikreek	RMNH.ins.516376
GEODE7692_Gomphidae_Phyllogomphoides_fuliginosus	1963-09-03	Belle, J.	Suriname	Kabalebo River	RMNH.ins.516246
GEODE2652_Platycnemididae_Asthenocnemis_stephanodera	1992-05-22	Borromeo, T.	Philippines	Palawan Ad, Quezon, Magmuni steam, 1000ft	
TM170_Aeshnidae_Remartinia_luteipennis	2014-04-16	Bota, C.	Colombia	Parque Nacional Natural Tatamá, Vereda Monte Bello; 5.22855; -76.09826667	
TM201_Philogeniidae_Philogenia_martae	2016-08-17	Bota, C.	Colombia	Parque Nacional Natural Tatamá, Vereda Monte Bello; 5.22855; -76.09826668	
TM332_Libellulidae_Cannaphila_vibex	2016-08-26	Bota, C.	Colombia	Parque Nacional Natural Tatamá, Vereda Monte Bello; 5.2149; -76.0857	
TM342_Libellulidae_Macrothemis_pseudimitans	2016-09-17	Bota, C.	Colombia	Risaralda, Pueblo Rico, Corregimiento Santa Cecilia; 5.3283; -76.1487	
TM392_Aeshnidae_Rhionaeschna_joannisi	2016-07-28	Bota, C.	Colombia	Parque Nacional Natural Tatamá, Vereda Monte Bello; 5.22855; -76.09826673	
TM404_Heteragrionidae_Heteragrion_erythrogastrum		Bota, C.	Colombia	Parque Nacional Natural Tatamá, Vereda Monte Bello; Bochoroma, 5.22855; -76.09826674	
TM409_Libellulidae_Rhodopygia_hinei		Bota, C.	Colombia	Risaralda, Pueblo Rico, Corregimiento Santa Cecilia; 5.3283; -76.1487	
TM41_Idomacromiidae_Neocordulia_batesi		Bota, C.	Colombia	Parque Nacional Natural Tatamá, Vereda Monte Bello;	

				5.22855; -76.09826676	
TM532_Libellulidae_Erythemis_plebeja		Bota, C.	Colombia	Parque Nacional Natural Tatamá, Vereda Monte Bello; 5.22855; -76.09826677	
TM581_Aeshnidae_Andaeschna_occidentalis		Bota, C.	Colombia	Parque Nacional Natural Tatamá, Vereda Monte Bello; 5.22855; -76.09826678	
TMCH16_Coenagrionidae_Protoneura_amatoria		Bota, C.	Colombia	Risaralda, Rio Sucio	
UAE1_Polythoridae_Euthore_fassli		Bota, C.	Colombia	Risaralda, Parque Nacional Natural Tatamá, Vereda Monte Bello, Quebrada Los Chorros; 5,25496° N; 76,11093° W	
RMNH.INS.508201_Platycnemididae_Arabineura_khalidi	2015-03-30	Boudot, J.-P.	Oman	Hadjar, Hawqayn; 23.52797, 57.32791	
GEODE17698_Corduliidae_Dorocordulia_lepida	2011-07-17	Bridgehouse, D.W.	Canada	Halifax Drysdale Bog, Big Indian & Pipeline Rd.	
GEODE17699_Corduliidae_Dorocordulia_libera	2012-06-02	Bridgehouse, D.W.	Canada	Nova Scotia Cumberland 8.5 NNE of Amherst	
GEODE17703_Corduliidae_Epitheca_cynosura	2012-06-30	Bridgehouse, D.W.	Canada	Nova Scotia Halifax Pockwock Watershed	
GEODE17912_Gomphidae_Hagenius_brevistylus	2010-07-05	Bridgehouse, D.W.	Canada	Nova Scotia, Hants County. Mt Uniacke, DND bog/fen	
RMNH.5009494_Euphaeidae_Epallage_fatime	2015-05-08	Brochard, C.	Bulgaria	Strandzha NP, Varovnik; 42.22322, 27.19295	
RMNH.5009497_Corduliidae_Corduliochlora_borisi	2015-05-08	Brochard, C.	Bulgaria	Strandzha NP, Varovnik; 42.22322, 27.19295	
RMNH.INS.507528_Aeshnidae_Boyeria_cretensis	2012-06-19	Brochard, C. & E.	Greece	Crete, Filaki, river 200 m NE of,;	

		van der Ploeg			
RMNH.INS.507529_Libellulidae_Trithemis_annulata	2012-06-19	Brochard, C. & E. van der Ploeg	Greece	Crete, Greece, Crete, Kaloniktis, river 2 km NW of;	
RMNH.INS.507551_Libellulidae_Selysiothemis_nigra	2012-05-09	Brochard, C. & E. van der Ploeg	Greece	Corfu, Greece, Corfu, Kavos, 1.3 km SW of,;	
GEODE7723_Synlestidae_Chlorolestes_elegans	2013-03-12	Bybee, S.M.	South Africa	Kruger National Park	
GEODE7753_Coenagrionidae_Telebasis_salva	2016-05-28	Bybee, S.M.	United States	Placerville; Gold Trail Park; Kathy Lane; 38.752413 -120.844354	
GEODE7813_Lestidae_Lestes_plagiatus	2013-03-12	Bybee, S.M.	South Africa	Kruger National Park	
OD1315_Chlorogomphidae_Chlorogomphus_auratus	2016-06-21	Bybee, S.M.	Vietnam	NE Region Ninh Binh Provience Cuc Phuong National Park 20°16.745N 105°40.757E	
OD1322_Corduliidae_Rialla_villosa	2015-11-01	Bybee, S.M.	Argentina		
OD830_Aeshnidae_Anax_walsinghami	2015	Bybee, S.M.	United States	Utah	
GEODE7733_Gomphidae_Tragogomphus_ellioti	2019-02-02	Bybee, S.M. & G. Powell	Gabon	Ivindo Nat. Pk. ; 0.5116N, 12.8032	
GEODE7807_Libellulidae_Tramea_transmarina	2018-05-14	Bybee, S.M. & G.S. Powell	Vanuatu	Unpongkor; -18.771033, 169.013417	
OD1112_Austropetaliidae_Phyllopetalia_apollo	2015-11-19	Bybee, S.M. & Pessacq P.	Argentina	Puelo lake, Puerto Patriada, Chubut Province; 42°08'17'' S 71°31'56'' W	
OD1113_Neopetaliidae_Neopetalia_punctata	2015-11-19	Bybee, S.M. & Pessacq P.	Argentina	Pil Pil stream, Neuquén Province; 40°11'05'' S 71°23'27'' W	

OD1115_Austropetaliidae_Hypopetalia_pestilens	2015-11-19	Bybee, S.M. & Pessacq P.	Argentina	Puelo lake, Puerto Patriada, Chubut Province; 42°08'17'' S 71°31'56'' W	
GEODE7709_Libellulidae_Rhyothemis_notata	2018-02-21	Bybee, S.M. & Y. M. Pacheco	Gabon	Stream at bottom of ravine; S02.36927, E11.16140	
GEODE7805_Libellulidae_Acisoma_trifidum	2018-02-17	Bybee, S.M. & Y. M. Pacheco	Gabon	Coastal forest S. of Gamba; 2.8355, 10.096683	
GEODE7714_Libellulidae_Brachythemis_leucosticta	2013-05-12	Bybee, S.M., G. Svenson, N. Hardy	Rwanda	Akagera National Park, Swamp/wetland by entrance gate; 1.928251S 30.674073E	
GEODE7715_Macromiidae_Phyllomacromia_picta	2013-05-07	Bybee, S.M., G. Svenson, N. Hardy	Rwanda	N. U. of Rwanda University Arboretum; S2.612869, E29.75995	
GEODE7722_Platystictidae_Protosticta_satoi	2016-06-21	Bybee, S.M., M. F. Whiting, T. H. Ogden	Vietnam	Cuc Phuong National Park; 20.20995N 103.35608E	
GEODE7731_Gomphidae_Ictinogomphus_pertinax	2016-06-21	Bybee, S.M., M. F. Whiting, T. H. Ogden	Vietnam	Cuc Phuong National Park; 20 20.995N 105 35.608E	
GEODE7734_Gomphidae_Heliogomphus_scorpio	2016-06-10	Bybee, S.M., M. F. Whiting, T. H. Ogden	Vietnam	Tav Yen Tu Nature Reserve; 21 11.029N 106 43.720E	
GEODE7705_Calopterygidae_Noguchiphaea_yoshikoae	2013-09-12	Bybee, S.M., N. P. Lord	Vietnam	Tam Dao National Park; N21.45255 S105.64282	

GEODE7698_Libellulidae_ Erythemis_credula	2017-06-09	Bybee, S.M., R. A. Clement, S. Smith & R. Ferreira	Brazil	Manaus; S3.230439, W60.64309	
GEODE7699_Libellulidae_ Erythrodiplax_umbrata	2017-06-15	Bybee, S.M., R. A. Clement, S. Smith & R. Ferreira	Brazil	Agropalma; S2.543239, W48.798426	
GEODE7700_Libellulidae_ Oligoclada_sp	2017-06-15	Bybee, S.M., R. A. Clement, S. Smith & R. Ferreira	Brazil	Agropalma; S2.543239, W48.798426	
GEODE7702_Libellulidae_ Uracis_fastigiata	2017-06-10	Bybee, S.M., R. A. Clement, S. Smith & R. Ferreira	Brazil	Manaus; S3.230439, W60.64309	
GEODE7707_Libellulidae_ Elga_sp	2017-06-15	Bybee, S.M., R. A. Clement, S. Smith & R. Ferreira	Brazil	Agropalma; S2.543239, W48.798426	
GEODE7710_Gomphidae_ Ebegomphus_sp	2017-06-15	Bybee, S.M., R. A. Clement, S. Smith & R. Ferreira	Brazil	Agropalma; S2.543239, W48.798426	
GEODE7712_Libellulidae_ Perithemis_sp	2017-06-14	Bybee, S.M., R. A. Clement,	Brazil	Agropalma; S2.521002, W48.855789	

		S. Smith & R. Ferreira			
GEODE7741_Coenagrionidae_Psaironeura_tenuissima	2017-06-09	Bybee, S.M., R. A. Clement, S. Smith & R. Ferreira	Brazil	Manaus; 2.833831S 60.931534W	
GEODE7742_Heteragrionidae_Oxystigma_petiolatum	2017-06-14	Bybee, S.M., R. A. Clement, S. Smith & R. Ferreira	Brazil	Agropalma; 2.521002S 48.855789W	
GEODE7748_Calopterygidae_Mnesarete_williamsoni	2017-06-15	Bybee, S.M., R. A. Clement, S. Smith & R. Ferreira	Brazil	Agropalma; 2.543239S 48.798426W	
GEODE7704_Rimanellidae_Rimanella_arcana	2017-06-11	Bybee, S.M., R. A. Clement, S. Smith, J. F. Lontchi	Brazil	Manaus; 1.992748S 59.557015W	
GEODE7718_Dicteriadidae_Dicterias_atrosanguinea	2017-06-10	Bybee, S.M., R. A. Clement, S. Smith, J. F. Lontchi	Brazil	Manaus; S2.846025, W60.916522	
GEODE7725_Coenagrionidae_Mecistogaster_linearis	2017-06-15	Bybee, S.M., R. A. Clement, S. Smith, J. F. Lontchi	Brazil	Agropalma; 2.605046S 48.738174E	

GEODE7750_Platycnemididae_Copera_nyansana	2017-07-28	Bybee, S.M., R. A. Clement, S. Smith, J. F. Lontchi	Cameroon	Bouamir Swampy Trail; 3.18965N 12.82299E	
GEODE7758_Coenagrionidae_Acanthagrion_adustum	2017-06-09	Bybee, S.M., R. A. Clement, S. Smith, J. F. Lontchi	Brazil	Manaus; 3.230439S 60.64309W	
GEODE7796_Platycnemididae_Elattoneura_acuta	2017-07-27	Bybee, S.M., R. A. Clement, S. Smith, J. F. Lontchi	Cameroon	Bouamir Tree Fall Stream; 3.18880N 12.83396E	
GEODE7798_Libellulidae_Palpopleura_lucia	2017-07-30	Bybee, S.M., R. A. Clement, S. Smith, J. F. Lontchi	Cameroon	Somalomo River crossing; 3.37148N 12.73367E	
RMNH.INS.506617_Idionychidae_Idionyx_philippa	2012-06-11	Cahilog, H.	The Philippines	Mindanao, Mindanao - Dispatcheng, Purok 5, Aliwagwag, Cateel, Davao Oriental;	
RMNH.INS.506657_Corduliidae_Heteronaias_heterodoxa	2012-06-13	Cahilog, H.	The Philippines	Mindanao, Mindanao - Marahan, Marilog district, Davao City;	
TM305_Coenagrionidae_Acanthagrion_trilobatum	2017-01-01	Cárdenas, B., J. Sandoval & C. Bota	Colombia	Risaralda, Pueblo Rico, Corregimiento Santa Cecilia, Vereda El Silencio; 5.347297; -76.134167	
RMNH.5009376_Gomphidae_Onychogomphus_styx	2014-07-23	Clausnitzer, V.	Uganda	Kibale National Park, around Field station; 0.55389, 30.36365	
GEODE17720_Corduliidae_Hemicordulia_kalliste	2005-01-17	Cleminson, G.	Australia	Queensland Ningi	

GEODE17917_Gomphidae_Octogomphus_specularis	1994-07-10	Cole, J.	United States	California: Tuolumne Co., North Fork of the Tuolumne River at Lair of the Bear, Camp Blue, 2 miles southwest of Pinecrest Lake, Stanislaus national Forest.	M. Elevation 1700 meters.
GEODE17610_Aeshnidae_Gomphaeschna_furcillata	2002-04-01	Cook, C.	Florida	Okaloosa County, Niceville Robert Lake at rd #190	
OD1074_Hemiphlebiidae_Hemiphlebia_mirabilis		Cordero Rivera, A.	Australia	Nelson, Victoria; 141.137474, -38.110307	Long swamp, WGS84, 11
OD1083_Argiolestidae_Austroargiolestes_icteromelas	2013-12-09	Cordero Rivera, A.	Australia	Victoria, Coral Bank, Kiewa river; 147.093135, -36.583770	266 m elevation
OD1085_Rhipidolestidae_Agriomorpha_xinglongensis	2014-05-29	Cordero Rivera, A.	China	Hainan, Lingshui, Diaoluoshan, stream near road; 109.879155, 18.685422	574 m elevation
OD1283_Pseudolestidae_Pseudolestes_mirabilis		Cordero Rivera, A.	China	Diaoluoshan National Park, Lingshui, Hainan; 109.880018, 18.690587	ACR-01154, WGS84, 601
GEODE17728_Libellulidae_Orthemis_sulphurata	20122-08-02	Daigle, J.J.	Aruba	Aruba, ditch east of church, Calbas road, Noord	2M. 900am. IORI #: 000094011. EX Jerrel J. Daigle Collection. Florida State Collection of Arthropods. Processed by: Bill Mauffray 12/5/2021
GEODE17781_Corduliidae_Neurocordulia_virginiensis	1981-05-21	Daigle, J.J.	United States	Arkansas Montgomery Little Missouri River at Camp	

GEODE17873_Austrocorduliidae_Lathrocordulia_metallica	1979-12-15	Davies, D.A.L.	Australia	Western Australia 120km S of Perth, Waroona, Nanga Brook	
GEODE17885_Synthemistidae_Archaeosynthemis_leachii	1979-12-09	Davies, D.A.L.	Australia	Western Australia Nanga Brook, Waroona 120km S. of Perth	
GEODE17888_Synthemistidae_Archaeosynthemis_spiniger	1979-12-12	Davies, D.A.L.	Australia	Western Australia Nanga Brook, Waroona 120km S. of Perth	
GEODE17889_Synthemistidae_Austrosynthemis_cyanitincta	1979-11-30	Davies, D.A.L.	Australia	Western Australia Drake's Drain, Waroona	
NESOMASC_Idomacromiidae_Nesocordulia_mascarenica	2007	Dijkstra, K.-D.B.	Madagascar		
OD1305_Synlestidae_Chlorolestes_umbratus	2015-12-23	Dijkstra, K.-D.B.	Madagascar		
OD1306_Platycnemididae_Elattoneura_glauca	2015-12-23	Dijkstra, K.-D.B.	Madagascar		
OD1308_Protolestidae_Protolestes_kerckhoffae	2016-01-13	Dijkstra, K.-D.B.	Madagascar		
OD1309_Argiolestidae_Nesolestes_sp	2016-01-14	Dijkstra, K.-D.B.	Madagascar		
RMNH.5007797_Coenagrionidae_Azuragrion_nigridorsum	2013-12-06	Dijkstra, K.-D.B.	Zimbabwe	Manicaland, 21 km SSE of Chipinge, Mt Selinda-Chipinge road, Ruwadzano	
RMNH.5010307_Coenagrionidae_Proischnura_polychromatica	2015-02-14	Dijkstra, K.-D.B.	South Africa	Western Cape, 22 km NE of Citrusdal, Cederberg, Driehoek; -32.45, 19.18	
RMNH.INS.229208_Coenagrionidae_Oreocnemis_phoenix	2000-11-16	Dijkstra, K.-D.B.	Malawi	Mt Mulanje, Dzole stream;	
RMNH.INS.501444_Gomphidae_Libyogomphus_christinae	2011-01-10	Dijkstra, K.-D.B.	Liberia	Nimba County, East Nimba Nature Reserve, Yiti valley; 7.48, -8.57	

RMNH.INS.501662_Libellulidae_Eleuthemis_buettikoferi	2011-02-25	Dijkstra, K.-D.B.	Sierra Leone	Eastern Province, 7 km south of Kenema, Moa River at Tilorma; 7.81236, -11.17794	
RMNH.INS.502121_Libellulidae_Oxythemis_phoenicosceles	2010-05-23	Dijkstra, K.-D.B.	Democratic Republic of Congo	Province Orientale, Lower Aruwimi, Yayofa (=Yaofa); 1.267, 23.68	
RMNH.INS.502132_Calopterygidae_Phaon_iridipennis	2010-05-23	Dijkstra, K.-D.B.	Democratic Republic of Congo	Province Orientale, Lower Aruwimi, Yayofa (=Yaofa); 1.267, 23.68	
RMNH.INS.502150_Libellulidae_Chalcostephia_flavifrons	2010-05-28	Dijkstra, K.-D.B.	Democratic Republic of Congo	Province Orientale, Lower Lomami, Iloko stream on Lieki-Lileke path; 0.6548, 24.2685	
RMNH.INS.502177_Libellulidae_Notiothemis_robertsi	2010-06-03	Dijkstra, K.-D.B.	Democratic Republic of Congo	Province Orientale, Lower Lomami, Yendega, Bosega & Bogunu streams in forest west of Djabir & Yaengo; 0.51, 24.16	
RMNH.INS.502225_Libellulidae_Aethiothemis_erythromelas	2010-05-02	Dijkstra, K.-D.B.	Democratic Republic of Congo	Province Orientale, Yaekela; 0.81, 24.28	
RMNH.INS.502232_Aeshnidae_Gynacantha_victoriae	2010-05-02	Dijkstra, K.-D.B.	Democratic Republic of Congo	Province Orientale, Yaekela; 0.81, 24.28	
RMNH.INS.502285_Libellulidae_Cyanothemis_simpsoni	2010-05-04	Dijkstra, K.-D.B.	Democratic Republic of Congo	Province Orientale, Lilanda & Baombo streams at and upstream of Lilanda & Yafake villages; 0.83, 24.34	
RMNH.INS.502287_Libellulidae_Micromacromia_camerunica	2010-05-04	Dijkstra, K.-D.B.	Democratic Republic of Congo	Province Orientale, Lilanda & Baombo streams at and upstream of Lilanda & Yafake villages; 0.83, 24.34	
RMNH.INS.502304_Libellulidae_Parazyxomma_flavicans	2010-05-11	Dijkstra, K.-D.B.	Democratic Republic of Congo	Province Equateur, Lower Itimbiri, Kona camp; 2.033, 22.787	

RMNH.INS.502305_Libellulidae_Hadrothemis_camarensis	2010-05-07	Dijkstra, K.-D.B.	Democratic Republic of Congo	Province Orientale, Lilanda & Baombo streams at and upstream of Lilanda & Yafake villages; 0.83, 24.34	
RMNH.INS.502324_Neophyidae_Neophya_rutherfordi	2010-05-04	Dijkstra, K.-D.B.	Democratic Republic of Congo	Province Orientale, Lilanda & Baombo streams at and upstream of Lilanda & Yafake villages; 0.83, 24.34	
RMNH.INS.502394_Gomphidae_Lestinogomphus_congoensis	2010-06-09	Dijkstra, K.-D.B.	Democratic Republic of Congo	Province Orientale, Old Buta Road, PK 13 (Point Kilometre from Kisangani); 0.59193, 25.25786	
RMNH.INS.502397_Libellulidae_Malgassophlebia_bispina	2010-06-08	Dijkstra, K.-D.B.	Democratic Republic of Congo	Province Orientale, Ubundu Road, Yoko Forest, Mokonoka Stream; 0.29402, 25.2888	
RMNH.INS.502400_Libellulidae_Nesciothemis_farinosa	2010-06-10	Dijkstra, K.-D.B.	Democratic Republic of Congo	Province Orientale, Kisangani, Scolasticat; 0.545, 25.215	
RMNH.INS.502847_Libellulidae_Thermochoria_jeanneli	2009-08-24	Dijkstra, K.-D.B.	Tanzania	Pemba North Region, 13km N of Wete, Pemba Island, Ngezi Forest; -4.93, 39.7	
RMNH.INS.502891_Gomphidae_Nepogomphoides_stuhlmanni	2009-08-26	Dijkstra, K.-D.B.	Tanzania	Tanga Region, Amani Nature Reserve, Zigi; -5.10009, 38.65206	
RMNH.INS.504459_Libellulidae_Hemistigma_albipunctum	2011-11-25	Dijkstra, K.-D.B.	Democratic Republic of Congo	Katanga, Kundelungu National Park, 2 km ESE of Katwe; -10.57, 27.88	
RMNH.INS.505345_Chlorocyphidae_Platycypha_caligata	2011-11-06	Dijkstra, K.-D.B.	Democratic Republic of Congo	Katanga, Kiubo; -9.52, 27.05	
RMNH.INS.505354_Libellulidae_Trithetrum_navasi	2011-11-06	Dijkstra, K.-D.B.	Democratic Republic of Congo	Katanga, Kiubo; -9.52, 27.05	

RMNH.INS.505362_Libellulidae_Crocothemis_erythraea	2011-11-06	Dijkstra, K.-D.B.	Democratic Republic of Congo	Katanga, Kiubo; -9.52, 27.05	
RMNH.INS.505380_Libellulidae_Olpogastra_lugubris	2011-11-07	Dijkstra, K.-D.B.	Democratic Republic of Congo	Katanga, Kiubo; -9.52, 27.05	
RMNH.INS.505397_Gomphidae_Neurogomphus_cocytius	2011-11-07	Dijkstra, K.-D.B.	Democratic Republic of Congo	Katanga, Kiubo; -9.52, 27.05	
RMNH.INS.505547_Libellulidae_Porpax_risi	2011-11-15	Dijkstra, K.-D.B.	Democratic Republic of Congo	Katanga, Upemba National Park, Kabwekanono, source area of Kafwi; -8.937, 27.166	
RMNH.INS.505588_Libellulidae_Diplacodes_lefebvrii	2011-11-17	Dijkstra, K.-D.B.	Democratic Republic of Congo	Katanga, Upemba National Park, Mukana, west of Lusinga; -8.91, 27.18	
RMNH.INS.505652_Libellulidae_Bradinopyga_cornuta	2011-11-22	Dijkstra, K.-D.B.	Democratic Republic of Congo	Katanga, Kundelungu National Park, Masansa; -10.532, 27.978	
RMNH.INS.505653_Gomphidae_Notogomphus_praetorius	2011-11-22	Dijkstra, K.-D.B.	Democratic Republic of Congo	Katanga, Kundelungu National Park, Lutshipuka; -10.557, 27.958	
RMNH.INS.559644_Platycnemididae_Spesbona_angusta	2013-10-27	Dijkstra, K.-D.B.	South Africa	Western Cape, 6 km SE of Franschhoek, Hottentots-Holland Nature Reserve, Purgatory; -33.97, 19.175	
RMNH.INS.559789_Libellulidae_Zygonoides_fuelleborni	2013-11-30	Dijkstra, K.-D.B.	Zimbabwe	Manicaland, 62 km S of Mutare, Umvumvumvu river below Mutambara; -19.528, 32.622	
RMNH.INS.559802_Gomphidae_Crenigomphus_hartmanni	2013-11-30	Dijkstra, K.-D.B.	Zimbabwe	Manicaland, 62 km S of Mutare, Umvumvumvu river below Mutambara; -19.528, 32.622	

RMNH.INS.501518_Gomphidae_Microgomphus_camerunensis	2011-01-22	Dijkstra, K.-D.B. & A. Dayeker	Liberia	Grand Gedeh County, Putu Iron Ore Mining concession, Biodiversity Camp; 5.65921, -8.20411	
RMNH.INS.503108_Gomphidae_Diastatomma_gamblesi	2011-03-01	Dijkstra, K.-D.B. & A. Dayeker	Sierra Leone	Eastern Province, Gola Forest, Wibwa (Wegbua) stream at Mayengema; 7.64262, -10.78986	
RMNH.INS.503116_Calopterygidae_Sapho_fumosa	2011-03-02	Dijkstra, K.-D.B. & A. Dayeker	Sierra Leone	Eastern Province, Gola Forest, Gagbe stream, 1 km west of Mogbaima; 7.65743, -10.77889	
RMNH.INS.503184_Idomacromiidae_Idomacromia_proavita	2011-03-11	Dijkstra, K.-D.B. & A. Dayeker	Sierra Leone	Eastern Province, Gola Forest, Sembehun (abandoned village); 7.7598, -10.71747	
RMNH.5007835_Synlestidae_Ecchlorolestes_nylephtha	2014-01-06	Dijkstra, K.-D.B. & F.I. Grundlingh	South Africa	Eastern Cape, 49 km ENE of Plettenberg Bay, Garden Route National Park (Tsitsikamma Section), Big Tree off N2 at Stormrivier; -33.96667, 23.89519	
RMNH.5007870_Platycnemididae_Metacnemis_valida	2014-01-09	Dijkstra, K.-D.B. & F.I. Grundlingh	South Africa	Eastern Cape, 4 km SE of Stutterheim, Kubusi river at railway bridge; -32.59899, 27.45191	
RMNH.5008090_Gomphidae_Ceratogomphus_pictus	2014-01-10	Dijkstra, K.-D.B. & F.I. Grundlingh	South Africa	Eastern Cape, 19 km SSW of Dordrecht, R392 just N of Bankies and Zixinene; -31.53109, 26.96422	
RMNH.5008153_Idomacromiidae_Syncordulia_gracilis	2014-01-19	Dijkstra, K.-D.B. & F.I. Grundlingh	South Africa	Western Cape, between Kleinmond and Betty's Bay, Kogelberg Nature Reserve, Palmiet River at Oudebosch; -34.31542, 18.96283	

RMNH.5007803_Libellulidae_Atoconeura_biordinata	2013-12-08	Dijkstra, K.-D.B. & U. Bjelke	Zimbabwe	Manicaland, 9 km NE of Nyanga village, northside of Nyanga National Park, Troutbeck; -18.18311, 32.80346	
RMNH.5007810_Coenagrionidae_Africallagma_glaucum	2013-12-08	Dijkstra, K.-D.B. & U. Bjelke	Zimbabwe	Manicaland, 9 km NE of Nyanga village, northside of Nyanga National Park, Troutbeck; -18.18311, 32.80346	
RMNH.INS.502555_Gomphidae_Gomphidia_gamblesi	2010-02-10	Dijkstra, K.-D.B., N. Mézière & C. Vanappelghem	Gabon	Haut-Ogooué, 50km N of Franceville, Franceville-Okondja Road, Léconi River at bridge N of Onvouri-Olounga; -1.17758, 13.53886	
GEODE17622_Macromiidae_Macromia_astarte	1972-10-25	Donnelly, N.	Papua New Guinea	Morobe Dist, stream at Gurakor, Wau Rd.	500 m
GEODE17711_Corduliidae_Guadalca_insularis	1987-02-13/24	Donnelly, N.	Solomon Islands	Guadalcanal Honiara Rove Cr. At Reservoir	
GEODE17883_Pseudocorduliidae_Pseudocordulia_circularis	1972-11-27	Donnelly, N.	Australia	Queensland Tchooratippa Cr., 30mi W. of Innisfail 2000ft	
GEODE17887_Synthemistidae_Archaeosynthemis_orientalis	1972-12-30	Donnelly, N.	Australia	A.C.T (Australian Capital Territory) Ginini Flats at Mt. Ginini 5200ft	
GEODE7866_Platycnemididae_Lieftinckia_malaitae	1987-02-19	Donnelly, N.	Solomon Islands		
GEODE17607_Aeshnidae_Andaeschna_rufipes	1997-01-15	Donnelly, T.W.	United States	Argentina, Jujuy; P.N. Calilegua; Ao. Tres Cruces (23°41.73'S 64°52.18'W; 1250m) 97x023	
GEODE17870_Idionychidae_Idionyx_optatus	1998-07-21/22	Donnelly, T.W.	Thailand	Chieng Mai Siriphum waterfall, nr Doi Inthanon 1000m	
GEODE17874_Macromidiidae_Macromidia_genialis	1994-07-23/24	Donnelly, T.W.	Thailand	Chantaburi Khao Soi Dao N.P.	

GEODE17881_Idomacromiidae_Neocordulia_griphus	1976-08-24	Donnelly, T.W.	Guatemala	Zacapa Quebrada La Ceiba, 2km W of Juan de Paz 100m	
GEODE7827_Coenagrionidae_Amphiallagma_parvum	2001-06-18	Donnelly, T.W.	Myanmar	Kandawgyi Botanical Garden; 21.093N, 96.470167E	
GEODE7863_Megapodagrionidae_Allopodagrion_contortum	1997-01-23	Donnelly, T.W.	Argentina	25°37.48'S 54°35.19'W	
GEODE7874_Thaumatoneuridae_Paraphlebia_duodecima	2007-06-11	Donnelly, T.W.	Guatemala	near Riachuelo Colorado tiny waterfall; 15°12.98'N 90°13.27'W	
GEODE17717_Corduliidae_Hemicordulia_fidelis	2013-04-14	Donnelly, T.W. & J. Michalski	New Caledonia	Nord Province Highway RP5 (Sarramea - Canala Rd) stream and picnic area 310m	
GEODE17896_Synthemistidae_Calesynthemis_campioni	0203-04-04	Donnelly, T.W. & J. Michalski	New Caledonia	Sud highway RP5 (Sarramea-Canala road) small stream; 330m	
GEODE7821_Argiolestidae_Eoargiolestes_ochraceus	2013-04-05	Donnelly, T.W. & J. Michalski	New Caledonia	highway RP5 (Sarramea-Canala Road), stream and picnic area; S21.5641, E165, 8319	
RMNH.5008306_Libellulidae_Neurothemis_fluctuans	2014-03-19	Dow, R.A.	Malaysia	Sarawak, Bintulu, Samarakan - T2N - stream with buffer near nursery;	
RMNH.5008334_Argiolestidae_Podolestes_orientalis	2014-03-18	Dow, R.A.	Malaysia	Sarawak, Bintulu, Samarakan - Sg Philip;	
RMNH.5008397_Platystictidae_Telosticta_ulubaram	2014-08-25	Dow, R.A.	Malaysia	Sarawak, Miri Division, Ulu Moh;	
RMNH.INS.228976_Libellulidae_Tetrathemis_irregularis	2008-01-02	Dow, R.A.	Malaysia	Sarawak, Miri Division, Gunung Mulu NP, Swamp on trail to Deer Cave;	
RMNH.INS.228999_Libellulidae_Raphismia_bispina	2008-02-02	Dow, R.A.	Malaysia	Sarawak, Bintulu Division, Similajau NP,;	

RMNH.INS.500989_Chlorocyphidae_Libellago_aurantiaca	2010-11-04	Dow, R.A.	Malaysia	Sarawak, Miri Division, Lambir Hills area, Simon La'ang's farm; 4.2362166667, 113.98548333	
RMNH.INS.501024_Chlorocyphidae_Sundacypha_petiolata	2010-11-20	Dow, R.A.	Malaysia	Sarawak, Bintulu Division, Anap Muput, Sg Sebelalang; 2.3433833333, 112.80668333	
RMNH.INS.501070_Calopterygidae_Echo_modesta	2010-12-11	Dow, R.A.	Malaysia	Pahang, Kuala Tahan, Tributary 3 to Sg Tembeling; 4.38155, 102.42205	
RMNH.INS.501089_Euphaeidae_Dysphaea_dimidiata	2010-12-10	Dow, R.A.	Malaysia	Pahang, Kuala Tahan,;	
RMNH.INS.501123_Libellulidae_Acisoma_panorpoides	2010-12-17	Dow, R.A.	Malaysia	Pahang, Gelanggi Caves; 3.8998, 102.47331667	
RMNH.INS.501127_Devadattidae_Devadatta_argyoides	2010-12-09	Dow, R.A.	Malaysia	Pahang, Kuala Tahan, Tributary 1 to Sg Tembeling; 4.39755, 102.4307	
RMNH.INS.501152_Libellulidae_Pseudothemis_jorina	2010-12-14	Dow, R.A.	Malaysia	Pahang, Kuala Tahan,; 4.3719833333, 102.40291667	
RMNH.INS.501184_Gomphidae_Phaenandrogomphus_asthenes	2010-12-12	Dow, R.A.	Malaysia	Pahang, Kuala Tahan, Tributary 1 to Sg Tembeling; 4.39755, 102.4307	
RMNH.INS.501189_Libellulidae_Aethriamanta_brevipennis	2010-12-17	Dow, R.A.	Malaysia	Pahang, Gelanggi Caves; 3.8998, 102.47331667	
RMNH.INS.501196_Libellulidae_Rhodothemis_rufa	2010-12-18	Dow, R.A.	Malaysia	Pahang, Gelanggi Caves; 3.8998, 102.47331667	
RMNH.INS.501275_Libellulidae_Orchithemis_xanthosoma	2010-05-28	Dow, R.A.	Malaysia	Sarawak, Kuching, Matang Rd, remnant peatswamp/rubber; 1.5736, 110.27875	
RMNH.INS.501284_Libellulidae_Brachygonia_oculata	2010-05-10	Dow, R.A.	Malaysia	Sarawak, Sibu, Sg. Teku area; 2.3503, 111.86538333	

RMNH.INS.501296_Libellulidae_Tholymis_tillarga	2010-06-19	Dow, R.A.	Malaysia	Sarawak, Bintulu, Camp C; 2.72445, 113.3492	
RMNH.INS.501373_Libellulidae_Chalybeothemis_fluviatilis	2009-09-21	Dow, R.A.	Malaysia	Pahang, Sg Bebar; 3.3116, 103.23533333	
RMNH.INS.501377_Libellulidae_Zyxomma_petiolatum	2009-09-21	Dow, R.A.	Malaysia	Pahang, Sg Bebar, swamp downstream; 3.2608333333, 103.24478333	
RMNH.INS.501398_Libellulidae_Pornothemis_sp	2009-09-22	Dow, R.A.	Malaysia	Pahang, Sg Bebar, swamp upstream; 3.3116, 103.23533333	
RMNH.INS.503416_Libellulidae_Lyriothemis_cleis	2010-06-25	Dow, R.A.	Malaysia	Sarawak, Bintulu, Camp C waterpipe path; 2.7192, 113.34625	
RMNH.INS.503419_Libellulidae_Nesoxenia_lineata	2010-06-30	Dow, R.A.	Malaysia	Sarawak, Bintulu, Kakus nursery, swamp near stream; 2.7701166667, 113.21476667	
RMNH.INS.503523_Gomphidae_Sieboldius_japponicus	2010-05-07	Dow, R.A.	Malaysia	Sarawak, Miri, Mulu, sg Melinau Paku upstream of w/f; 4.0475666667, 114.83643333	
RMNH.INS.503524_Gomphidae_Acrogomphus_jubilaris	2010-05-24	Dow, R.A.	Malaysia	Sarawak, Kapit, Hose range, perched at side of tractor path in morning, ca 900m; 2.07965, 113.69446667	
RMNH.INS.503619_Coenagrionidae_Aciagrion_borneense	2010-06-19	Dow, R.A.	Malaysia	Sarawak, Bintulu, Camp C; 2.72445, 113.3492	
RMNH.INS.503929_Platycnemididae_Calicnemia_chaseni	2011-08-21	Dow, R.A.	Malaysia	Terengganu, Small stream near Sg Lasir;	
RMNH.INS.503939_Libellulidae_Hydrobasileus_croceus	2011-08-22	Dow, R.A.	Malaysia	Terengganu, Pond just outside Sekayu Rec Forest;	

RMNH.INS.503943_Libellulidae_Indothemis_limbata	2011-08-22	Dow, R.A.	Malaysia	Terengganu, Pond just outside Sekayu Rec Forest;	
RMNH.INS.503944_Platycnemididae_Pseudocopera_ciliata	2011-08-22	Dow, R.A.	Malaysia	Terengganu, Open stream just outside Sekayu Rec Forest;	
RMNH.INS.503951_Coenagrionidae_Argiocnemis_rubescens	2011-08-23	Dow, R.A.	Malaysia	Terengganu, Bukit Kesing Recreational forest, muddy trail pool;	
RMNH.INS.503957_Libellulidae_Potamarcha_congener	2011-08-24	Dow, R.A.	Malaysia	Terengganu, Marshy area by Sg Peres just outside Sekayu Rec Forest;	
RMNH.INS.503963_Libellulidae_Lathrecista_asiatica	2011-08-24	Dow, R.A.	Malaysia	Terengganu, Marshy area by Sg Peres just outside Sekayu Rec Forest;	
RMNH.INS.503964_Libellulidae_Urothemis_signata	2011-08-24	Dow, R.A.	Malaysia	Terengganu, Pond near Sekayu Rec Forest;	
RMNH.INS.504032_Libellulidae_Onychothemis_culminicola	2011-09-14	Dow, R.A.	Malaysia	Sarawak, Bintulu, T1C stream, SPFP;	
RMNH.INS.504039_Libellulidae_Orchithemis_pulcherrima	2011-09-19	Dow, R.A.	Malaysia	Sarawak, Bintulu, T1?, small stream in acacia, upstream of road;	
RMNH.INS.506247_Macromidiidae_Macromidia_fulva	2012-03-23	Dow, R.A.	Malaysia	Sarawak, Bintulu, Camp stream;	
RMNH.INS.506254_Libellulidae_Camacinia_gigantea	2012-03-27	Dow, R.A.	Malaysia	Sarawak, Bintulu, Bukit Kana/Bukit Naong - logging track on lower slopes;	
RMNH.INS.506261_Platystictidae_Telosticta_bidayuh	2012-03-30	Dow, R.A.	Malaysia	Sarawak, Kuching, Kubah, high stream;	
RMNH.INS.506264_Gomphidae_Leptogomphus_coomansi	2012-04-01	Dow, R.A.	Malaysia	Sarawak, Kuching, MWC - Sg Rayu;	
RMNH.INS.506290_Libellulidae_Cratilla_lineata	2012-04-06	Dow, R.A.	Malaysia	Sarawak, Miri, Outside Jaman's lodge, ditches etc;	

RMNH.INS.506367_Gomphidae_Megalogomphus_borneensis	2012-05-13	Dow, R.A.	Malaysia	Sarawak, Miri, Mulu - BO stream;	
RMNH.INS.506835_Philosinidae_Rhinagrion_macrocephalum	2012-08-02	Dow, R.A.	Malaysia	Johor, Gn Belumut - backwater & trib to main stream;	
RMNH.INS.506912_Aeshnidae_Indaeschna_grubaueri	2012-09-30	Dow, R.A.	Malaysia	Sarawak, Kuching, Kubah - frog pond;	
RMNH.INS.507659_Platystictidae_Drepanosticta_actaeon	2012-09-10	Dow, R.A.	Malaysia	Sabah, Poring - stream running into Bamboo garden;	
RMNH.INS.507703_Coenagrionidae_Stenagrion_dubium	2012-09-13	Dow, R.A.	Malaysia	Sabah, Liwagu at side;	
RMNH.INS.507707_Chlorocyphidae_Rhinoneura_villosipes	2012-09-13	Dow, R.A.	Malaysia	Sabah, Kinabalu HQ - Silau-Silau;	
RMNH.INS.507722_Calopterygidae_Matronoides_cyaneipennis	2012-09-14	Dow, R.A.	Malaysia	Sabah, Sayap - sg Kemantis;	
RMNH.INS.507746_Libellulidae_Hylaeothemis_clementia	2012-09-17	Dow, R.A.	Malaysia	Sabah, Sayap - drain by road;	
RMNH.INS.507799_Calopterygidae_Vestalis_amaryllis	2012-09-29	Dow, R.A.	Malaysia	Sarawak, Kuching, Kubah - stream behind rest house;	
RMNH.INS.507831_Libellulidae_Tyriobapta_torrida	2012-10-05	Dow, R.A.	Malaysia	Sarawak, Kuching, Kpg Sebako - slow stream & swamp area;	
RMNH.INS.557636_Coenagrionidae_Pericnemis_sticticа	2013-08-07	Dow, R.A.	Malaysia	Sarawak, Bintulu, Bukit Sarang - swamp and small streams on trail to clearings;	
GEODE17832_Corduliidae_Somatochlora_cingulata	2000-08-01	Dunkle, S.W.	Canada	British Columbia Solco Lake & Fen, E of Okanagan Falls 5600ft	
GEODE17891_Synthemistidae_Eusynthemis_guttata	2003-01-07	Dunkle, S.W.	Australia	Victoria Eurobin Creek at entrance to Buffalo National Park	

GEODE7836_Coenagrionidae_Diceratobasis_melanogaster	1989-06-01	Dunkle, S.W.	Dominican Republic	stream on road to Constanza, 8.4 km from Autopista Duarte	
OD146_Corduliidae_Cordulia_aenea	1999-12-12	Ekestubbe, K.	Sweden	Sverige, So, Sodertalje Kn 656016 159643	male
OD1314_Gomphidae_Ophiogomphus_morrisoni	2016-10-20	Erickson, R.J.	United States	Utah Beaver County Beaver River, Hanging Rock Recreation Site N38.21437° W112.86496°	
GEODE7800_Libellulidae_Diplacodes_trivialis	2018-07-16	Erickson, R.J. , Y. M. Pacheco & S. M. Bybee	Vietnam	Near checkpoint station; 22.45518N 105.67502E	
GEODE7801_Libellulidae_Crocothemis_servilia	2018-07-16	Erickson, R.J. , Y. M. Pacheco & S. M. Bybee	Vietnam	Stream outside of Ba Be N.P.; 22.42314N 105.64756E	
GEODE7811_Platycnemididae_Copera_marginipes	2018-07-17	Erickson, R.J., Y. M. Pacheco & S. M. Bybee	Vietnam	Cuc Phuong N.P., Hotel; N20.2501, E105.71429	
GEODE7721_Calopterygidae_Neurobasis_chinensis	2018-07-15	Erickson, R.J., Y. M. Pacheco, S. M. Bybee	Vietnam	Ba Be N.P, trail to waterfall; N22.42055, E105.63834	
GEODE7724_Platycnemididae_Coeliccia_cyanomelas	2018-07-12	Erickson, R.J., Y. M. Pacheco, S. M. Bybee	Vietnam	Pia Oac Nature Reserve	
GEODE7727_Platycnemididae_Prodasineura_autumnalis	2018-07-16	Erickson, R.J., Y. M.	Vietnam	Stream outside of Ba Be N.P; N22.42314, E105.64756	

		Pacheco, S. M. Bybee			
GEODE7812_Chlorocyphidae_Heliocypha_perforata	2018-07-16	Erickson, R.J., Y. M. Pacheco, S. M. Bybee	Vietnam	Stream outside of Ba Be N.P; N22.42314, E105.64756	
GEODE7802_Libellulidae_Aethriamanta_rezia	2020-12-20	Fomekong Lontchi, J.	Cameroon	Mbalmayo 4; N3.4848 E11.5010	
GEODE7804_Libellulidae_Tetrathemis_fraseri	2020-12-20	Fomekong Lontchi, J.	Cameroon	Mbalmayo 1; N3.4741 E11.4006	
GEODE7806_Libellulidae_Orthetrum_julia	2020-07-27	Fomekong Lontchi, J.	Cameroon	Mbalmayo 6; N3.4697 E11.5004	
GEODE7624_Platycnemididae_Disparoneura_apicalis	1923-05-06	Fraser, F.C.	India	S.India, Cauvery River, Fraserpet, Coorg	
GEODE7873_Synlestidae_Phylolestes_ethelae	1980-08-25	Garrison, R.W.	Dominican Republic	small stream on Constaza rd. 14 km E of Autopista Duarte	
GEODE7879_Coenagrionidae_Forcepsioneura_sancta	2000-12-02	Garrison, R.W.	Brazil	Ilha de Marambaia, Gruta da Santa; 23 3'35" S, 43 57'56" W	
RMNH.INS.500397_Argiolestidae_Allolestes_maclachlanii	2008-12-12	Gerlach, J.	Seychelles	Mahe,;	
RMNH.INS.500398_Coenagrionidae_Leptocnemis_cyanops	2008-12-12	Gerlach, J.	Seychelles	Mahe,;	
GEODE7628_Libellulidae_Rhodopygia_hinei	1936-03-19	Gertsch, W.J.	Panama	Barro Colorado Island	
GEODE17757_Libellulidae_Archaeophlebia_martini	1963-01-16/20	Gould	Madagascar	Perinet	
GEODE7669_Aeshnidae_Amphiaeschna_ampla	1941-05-16	Groenendael, J.M.A.	Indonesia	West Java, Gunung Gedeh	RMNH.ins.1153207
OD132_Euphaeidae_Euphaea_ochracea		Grummel	Thailand	Chiay Mai Prov., Doi Sutheds Matl Parks	

TM154_Polythoridae_Polythore_gigantea	2016-08-23	Guzmán, C., J. Sandoval & C. Bota	Colombia	Parque Nacional Natural Tatamá, Vereda Monte Bello, La Clarita	
GEODE12747_Libellulidae_Amphithemis_curvistyla	1999-10-20	Hamalainen, M.	Thailand	Kanchanaburi, Thong Pha Phum area, Nam Tok Nang Kroan	
GEODE12754_Gomphidae_Mattigomphus_pinratani	1999-05-19	Hamalainen, M.	Thailand	Chiang Mai, Doi Inthanon, 1300 m, Siriphum wf.	
RMNH.INS.502054_Calopterygidae_Caliphaea_confusa	2009-05-20	Hamalainen, M.	Vietnam	Lao Cai, Sapa, Hoang Lien, small streams between 22°20'59"N 103°46'18"E and 22°20'51"N 103°46'10"E; 22.35, 103.772	
RMNH.INS.505736_Chlorocyphidae_Aristocypha_fenestrella	2009-01-15	Hamalainen, M.	Thailand	Ranong, Khlong Nakha, Khlong Bangmun;	
RMNH.INS.505747_Calopterygidae_Vestalaria_smaragdina	2009-01-08	Hamalainen, M.	Thailand	Chiang Mai, Near the Headquarters Garden, Doi Suthep, Stream below Pau Pau w/f;	
RMNH.INS.505754_Gomphidae_Amphigomphus_somnuki	2009-06-04	Hamalainen, M.	Thailand	Chiang Mai, Doi Suthep, Pau Pau w/f;	
RMNH.INS.505757_Euphaeidae_Anisopleura_furcata	2002-05-20	Hamalainen, M.	Thailand	Chiang Mai, Doi Inthanon,;	
RMNH.INS.505806_Calopterygidae_Psolodesmus_kuroiwae			Japan	Okinawa	
RMNH.INS.505817_Euphaeidae_Cryptophaea_vietnamensis	2009-04-28	Hamalainen, M.	Laos	Lak Sao;	
RMNH.INS.506232_Platycnemididae_Matticnemis_doi	2010-06-21	Hamalainen, M.	Vietnam	Lang Son, Huu Lien, Tan Lai village, Hang Chau stream;	
OD122_Lestidae_Sympecma_paedisca	2002-08-27	Jarvis & Taylor	Russia	Altaiskii Krai, Barnaul City, SE side, 2 km from Ob River	

SRR1695233_Ephemeroptera_Ecdyonurus_insignis	2013-06-05	Jeanne Wilbrandt, Tanja Ziesmann, Oliver Niehuis, Ralph Peters	Germany	North Rhine-Westphalia, Donrath	
OD1172_Austrocorduliidae_Austrocordulia_refracta		JLW	Australia		
OD1183_Mesagrionidae_Mesagrion_leucorrhinum		JLW	Colombia		
GEODE7767_Libellulidae_Nephepeltia_phryne	2016-11-12	Juillerat, J. & N. Mézière	French Guiana	Sinnamary, 3.611356, -53.174503	121116-6, male
GEODE7785_Coenagrionidae_Inpabasis_rosea	2016-11-04	Juillerat, J. & N. Mézière	French Guiana	Saül; 3.603129, -53.208978	041116-33, male
GEODE7786_Coenagrionidae_Acanthallagma_luteum	2016-11-02	Juillerat, J. & N. Mézière	French Guiana	Saül; 3.611356, -53.174503	
GEODE12746_Libellulidae_Akrothemis_risi	2009-07-25	Kaize, J.	Indonesia	Province Papua, Kab. Asmat, Vrinedschap R.; S05 20 20, E138 52 72	
GEODE2758_Coenagrionidae_Plagulibasis_ciliata	2009-07-12	Kaize, J.	Indonesia	Prov Papua, Kab. Mappi-Katan, S06'40,60 E139'14.87	
RMNH.INS.502075_Argiolestidae_Metagrion_ornatum	2009-08-18	Kaize, J.	Indonesia	Papua Barat, Kabupaten Wondama,, Mioswaar, Pantai Barat, Waprak;	
OD1126_Heteragrionidae_Dimeragrion_percubitale		Kalkman, V.J.	Venezuela		
OD1130_Philosinidae_Philosina_buchi		Kalkman, V.J.	China		
OD1132_Platycnemididae_Onychargia_atrocyana		Kalkman, V.J.	Malaysia		
OD1133_Amanipodagrionidae_Amanipodagrion_gilliesi		Kalkman, V.J.	Tanzania		
OD1135_Thaumatoneuridae_Paraphlebia_sp		Kalkman, V.J.	Mexico		

OD1136_Protolestidae_Protolestes_fickei		Kalkman, V.J.	Madagascar		
OD1137_Tatocnemididae_Tatocnemis_malgassica		Kalkman, V.J.	Madagascar		
OD1139_Pentaphlebiidae_Pentaphlebia_mangana		Kalkman, V.J.	Gabon		
OD1141_Thaumatoneuridae_Thaumatoneura_inopinata		Kalkman, V.J.	Costa Rica		
OD1143_Platycnemididae_Mesocnemis_singularis		Kalkman, V.J.	South Africa		
OD1146_Hypolestidae_Hypolestes_sp		Kalkman, V.J.	Dominican Republic		
OD1148_Amphipterygidae_Amphipteryx_agrioides		Kalkman, V.J.	Dominican Republic		
OD1278_Lestidae_Indolestes_cyaneus	2016-08-13	Kalkman, V.J.	Bhutan		
OD1302_Epiophlebiidae_Epiophlebia_laidlawi		Kalkman, V.J.	Bhutan		RMNH.5011536
OD1303_Aeshnidae_Gynacanthaeschna_sikkima		Kalkman, V.J.	Bhutan	Chirang, Kikhorthang	RMNH.5015076
OD1304_Cordulegastridae_Neallogaster_hermionae		Kalkman, V.J.	Bhutan		
RMNH.5011399_Gomphidae_Stylurus_flavipes	2014-08-27	Kalkman, V.J.	Netherlands	Noord-Brabant, Klundert - Kleine Ton;	
RMNH.INS.229243_Isostictidae_Selysioneura_capreola	2006-08-02	Kalkman, V.J.	Indonesia	Papua, Abepura,; -2.582, 140.651	
RMNH.INS.229291_Platycnemididae_Torrenticnemis_filicornis	2008-10-27	Kalkman, V.J.	Indonesia	Papua, Walmak (Nipsan),; -4.117, 139.633	
RMNH.INS.500513_Platycnemididae_Lochmaeocnemis_malacodora	2006-07-28	Kalkman, V.J.	Indonesia	Papua, Star mountains, Borme; -4.396, 140.434	
RMNH.INS.500518_Corduliidae_Procordulia_leopoldi	2006-07-29	Kalkman, V.J.	Indonesia	Papua, a few km from Borme village, Star mountains, Borme; -4.392, 140.428	
RMNH.INS.500525_Synthemistidae_Palaeosynthemis_primigenia	2006-07-27	Kalkman, V.J.	Indonesia	Papua, Star mountains, Borme; -4.396, 140.434	

RMNH.INS.500580_Platycnemididae_Palaiargia_charmosyna	2006-08-02	Kalkman, V.J.	Indonesia	Papua, Abepura,; -2.582, 140.651	
RMNH.INS.500592_Libellulidae_Huonia_epinephela	2006-07-11	Kalkman, V.J.	Indonesia	Papua, Yapen, Ambaidiru village; -1.763, 136.179	
RMNH.INS.500639_Platycnemididae_Idiocnemis_obliterata	2008-10-25	Kalkman, V.J.	Indonesia	Papua, Lelambo,; -4.033, 139.787	
RMNH.INS.500644_Libellulidae_Protorthemis_coronata	2008-10-25	Kalkman, V.J.	Indonesia	Papua, Lelambo,; -4.033, 139.787	
RMNH.INS.501977_Platycnemididae_Arrhenocnemis_parvibullis	2009	Kalkman, V.J.	Papua New Guinea		
RMNH.INS.504852_Coenagrionidae_Austrocoenagrion_lyelli	2011-12-21	Kalkman, V.J.	Australia	NSW, Guyra, Mother of Duck Lagoon, SE corner,; -30.2466, 151.6667	
RMNH.INS.504908_Aeshnidae_Telephlebia_godeffroyi	2011-12-30	Kalkman, V.J.	Australia	NSW, Blue Mountains, Blackheath, Govetts Leap Brook,; -33.6328, 150.313	
RMNH.INS.504910_Synthemistidae_Synthemis_eustalacta	2011-12-31	Kalkman, V.J.	Australia	NSW, Kanangra Boyd NP, Boyd River Campground,; -33.9702, 150.057	
RMNH.INS.504941_Aeshnidae_Notoaeschna_sagittata	2012-01-01	Kalkman, V.J.	Australia	NSW, Blue Mountains, Blackheath, Govetts Leap Brook,; -33.6328, 150.313	
RMNH.INS.504961_Gomphidae_Austrogomphus_ochraceus	2012-01-04	Kalkman, V.J.	Australia	NSW, Blue Mountains, Glenbrook, Bruce Road,; -33.7788, 150.6184	
RMNH.INS.504999_Libellulidae_Orthetrum_cancellatum	2012-07-24	Kalkman, V.J.	Netherlands	Meinweg; 51.15, 6.12	
RMNH.INS.505016_Aeshnidae_Anax_imperator	2012-06-02	Kalkman, V.J.	Netherlands	Leidse Hout; 52.17, 4.47	

RMNH.INS.505017_Aeshnidae_Isoaeshna_isoceles	2012-06-02	Kalkman, V.J.	Netherlands	Leidse Hout; 52.17, 4.47	
RMNH.INS.505254_Coenagrionidae_Austrocnemis_splendida	2011-12-09	Kalkman, V.J.	Australia	Queensland, Mapleton, Mapleton, Lilyponds park,; -26.622, 152.8643	
RMNH.INS.507392_Libellulidae_Leucorrhinia_rubicunda	2012-05-18	Kalkman, V.J.	Netherlands	Bosbeek, Meinweg; 51.16, 6.09	
RMNH.INS.507400_Libellulidae_Libellula_depressa	2012-05-19	Kalkman, V.J.	Netherlands	Meinweg; 51.156, 6.137	
RMNH.INS.507402_Aeshnidae_Brachytron_pratense	2012-05-19	Kalkman, V.J.	Netherlands	Meinweg; 51.151, 6.125	
RMNH.INS.507439_Libellulidae_Libellula_quadrimaculata	2012-05-26	Kalkman, V.J.	Netherlands	Leidse Hout; 52.17, 4.47	
RMNH.INS.507448_Lestidae_Chalcolestes_viridis	2012-08-01	Kalkman, V.J.	Netherlands	De Wolden; 533, 224	
RMNH.INS.507465_Libellulidae_Sympetrum_danae	2012-08-18	Kalkman, V.J.	Netherlands	Balloerveld; 558, 239	
RMNH.INS.507492_Libellulidae_Nannodiplax_rubra	2012-10-13	Kalkman, V.J.	Australia	Northern Territory, Howard Spring, old swimming pool; -12.4565, 131.0522	
OD1277_Isostictidae_Neosticta_fraseri	2015-01-10	Kalkman, V.J. & G. Theischinger	Australia	Queensland, Wooroonooran Park, Gooligans creek, stream; -17.6048 S 145.7622 E	
RMNH.5011424_Lestoideidae_Diphlebia_euphoeoides	2014-12-31	Kalkman, V.J. & G. Theischinger	Australia	Queensland, Station Creek, west of Mt Molloy; 145.2226, -16.6121	
RMNH.5011429_Gomphidae_Hemigomphus_comitatus	2014-12-31	Kalkman, V.J. & G. Theischinger	Australia	Queensland, Station Creek, west of Mt Molloy; 145.2226, -16.6121	
RMNH.5011432_Synthemistidae_Tonyosynthemis_claviculata	2014-12-31	Kalkman, V.J. & G. Theischinger	Australia	Queensland, Station Creek, west of Mt Molloy; 145.2226, -16.6121	

RMNH.5011475_Isostictidae_Oristicta_filicicola	2015-01-07	Kalkman, V.J., G. Theischinger & S. Richards	Australia	Queensland, Wooroonooran Park, Josephine Falls; 145.8594, -17.4363	
RMNH.5011478_Gomphidae_Austrogomphus_prasinus	2015-01-08	Kalkman, V.J., G. Theischinger & S. Richards	Australia	Queensland, Little Mulgrave, Gillies Hw; 145.7232, -17.1405	
RMNH.5011485_Synlestidae_Chorismagrion_risi	2015-01-09	Kalkman, V.J., G. Theischinger & S. Richards	Australia	Queensland, Paluma Range NP, Paluma Crystal Creek, first small creek; 146.2645, -190163	
OD1076_Epiophlebiidae_Epiophlebia_superstes	2013-05-06	Karube, H.	Japan	Sagamihara city, Shiroyama tawn, Amefurasshi	
RMNH.INS.507575_Macromiidae_Macromia_splendens	2012-06-18	Keijl, G.O.	France	Mialet; 44.106, 3.948	
RMNH.INS.507576_Idomacromiidae_Oxygastra_curtisii	2012-06-18	Keijl, G.O.	France	Mialet; 44.106, 3.948	
GEODE17756_Libellulidae_Antidythemis_trameiformis	1963-01	Kesselring, J.	Brazil	Pará, Belém	
RMNH.INS.507929_Gomphidae_Phyllogomphus_annulus	2012-05-19	Kipping, J.	Angola	Cuando-Cubango, Cuanavale River NE of Cuito Cuanavale,; -15.13569, 19.19571	
RMNH.INS.508001_Libellulidae_Zygonyx_torridus	2010-03-08	Kipping, J.	Zambia	Livingstone, Livingstone, Maramba River Lodge; -17.887817, 25.854138	
RMNH.INS.508012_Gomphidae_Paragomphus_genei	2010-01-12	Kipping, J.	Botswana	Ngamiland, Meno-a-Kwena Camp, Boteti; -20.32262, 24.31857	
RMNH.INS.509207_Gomphidae_Burmagomphus_divaricatus	2011-08-23	Kosterin, O.	Cambodia	Koh Kong,;	
RMNH.INS.509920_Gomphidae_Lamelligomphus_castor		Kosterin, O.	Cambodia		

RMNH.INS.509971_Gomphidae_Orientogomphus_minor	2013-06-02	Kosterin, O.	Cambodia	Rattanakiri, O'Sieng Lei waterfalls;	
GEODE7691_Aeshnidae_Heliaeschna_crassa	1936-09-26	Kuiper, F.J.	Indonesia	W Billiton, Tandjong Pandan	RMNH.ins.1153186
GEODE17761_Libellulidae_Boninthemis_insularis	1977-06-18	Kuwahara	Japan	Okimura-Mt. Funeki Hahashima, Ogasawara-Is Tokyo-To	
GEODE7854_Coenagrionidae_Leptobasis_melinogaster	2006-10-16	Langshied, T.	United States	Santa Gertrudis Creek, King Ranch; 27°35.4'N, 98°2.6'W	
GEODE17935_Gomphidae_Anormogomphus_kiritshenkoi	1935-06-10	Lazar, Y.	Iraq	Baghdad	Carl Cook Collection
GEODE7839_Coenagrionidae_Telagrion_longum	1998-04-10	Lencioni, A.A.	Brazil	Fazenda Santana do Rio Abaixo - Jacarei - SP; 23°14'52"S E, 46°59'58" W	
GEODE7835_Coenagrionidae_Cyanallagma_angelae	1999-03-14	Lencioni, F.A.A.	Brazil	-23.5975S, -45.7280556W	
GEODE7665_Lestidae_Indolestes_albicaudus	1948-08-09	Lieftinck, M.A.	Indonesia	NW New Guinea, Sorong	
GEODE7667_Chlorocyphidae_Libellago_greeni	1938-09-16	Lieftinck, M.A.	Sri Lanka	Ceylon, C.P.,Haragama, 500m	
GEODE7683_Euphaeidae_Anisopleura_lestoides	1978-10-20	Lieftinck, M.A.	India	NW India, UP, Kumaon, 1500m, Sat Tal	
OD1298_Isostictidae_Cnemisticta_latilobata	2015-09-11	Marinov, M.	Solomon Islands	Rap-Guadalcanal, 2015 Valevahalo base camp; 9.6468° S 160.0447° E	810 m a.s.l.
RMNH.INS.500853_Argiolestidae_Eoargiolestes_ochraceus	2009-01-11	Marinov, M.	New Caledonia	;	
RMNH.INS.500854_Argiolestidae_Trineuragrion_percostale	2009-01-18	Marinov, M.	New Caledonia	;	
RMNH.INS.505880_Libellulidae_Tapeinothemis_boharti	2012-04-28	Marinov, M.	Solomon Islands	Salapuka River in the Honiara Botanical Garden, Guadalcanal Island; -9.4363, 159.9417	

OD55_Isostictidae_Rhadinosticta_simplex	2000-02-21	Mathieson, M.	Australia	Queensland, Barakula State Forest 302; 26 20' S. 150 58' E.	
OD69_Gomphidae_Ictinogomphus_australis	2000-01-20	Mathieson, M.	Australia	Queensland, Tuchekoi, Area near Gympie: Grassy edges and pools around Chinaman's creek; 26 24'43" S. 152 45'44" E.	
OD1312_Isostictidae_Labidiosticta_vallisi	2000-02-05	Mathieson, M., J. Skevington & A. Skevington	Australia	Queensland, Brisbane, Forest Park; Scrub Creek; 27°25'05"S 152°50'13"E	
OD1317_Platycnemididae_Nososticta_solida	2000-01-03	Mathieson, M., J. Skevington & A. Skevington	Australia	Queensland Slanthorpe Along River 28°39'54"S 151°56'26"E	
OD1319_Libellulidae_Macrodiplax_cora	2000-03-04	Mathieson, M., J. Skevington & A. Skevington	Australia	Queensland Lake Clarendon 27°31'04"S 152°21'22"E	
OD43_Lestoideidae_Diphlebia_coerulescens	2000-02-05	Mathieson, M., J. Skevington & A. Skevington	Australia	Queensland, Brisbane, Forest Park; Scrub Creek; 27 25'05" S, 152 50'13"E	
OD85_Gomphidae_Austrogomphus_amphiclitus	2000-02-05	Mathieson, M., J. Skevington & A. Skevington	Australia	Brisbane, Forest Park: Scrub Creek, 27 25 05 S. 152 50'13"	
GEODE7777_Libellulidae_Dythemis_nigra	2020-05-20	Mauffray, B.	Panama	Oeste Province; capira district; el carmen, CA, 4KM west off of via el Caco	

				de Capira, small, intermittent stream	
GEODE7833_Coenagrionidae_Calvertagrion_mauffrayi	1995-06-12	Mauffray, B.	Ecuador	borrow pit nr. Rio Savaleto, km marker 31; -0.47, -76.85	
GEODE7852_Coenagrionidae_Homeoura_lindneri	2019-12-17	Mauffray, B.	Paraguay	San Antonio,,Rio Parana,; -27.3913, -56.8367	
GEODE7857_Coenagrionidae_Platystigma_astictum	1998-11-12	Mauffray, B.	Bolivia		
GEODE7761_Libellulidae_Tauriphila_argo	2015-11-28	Mézière, N.	French Guiana	Sinnamary; 5.1233611, -52.9553055	
GEODE7762_Libellulidae_Macrothemis_nobilis	2016-06-24	Mézière, N.	French Guiana	Sinnamary; 5.1233611, -52.9553055	
GEODE7763_Gomphidae_Archaeogomphus_nanus	2016-07-03	Mézière, N.	French Guiana	Sinnamary; 5.1233611, -52.9553055	
GEODE7764_Libellulidae_Miathyria_marcella	2017-06-28	Mézière, N.	French Guiana	Kourou; 5.148015, -52.635704	280617-1, male
GEODE7766_Libellulidae_Zenithoptera_fasciata	2015-12-01	Mézière, N.	French Guiana	Sinnamary; 5.1233611, -52.9553056	011215-1, male
GEODE7768_Libellulidae_Elasmothemis_williamsoni	2015-11-28	Mézière, N.	French Guiana	Sinnamary; 5.1233611, -52.9553055	
GEODE7770_Libellulidae_Gynothemis_pumilia	2016-06-17	Mézière, N.	French Guiana	Sinnamary; 5.1233611, -52.9553055	
GEODE7771_Lauromacromiidae_Lauromacromia_dubitalis	2016-01-16	Mézière, N.	French Guiana	Sinnamary; 5.1233611, -52.9553055	
GEODE7772_Gomphidae_Ebegomphus_minutus	2016-01-26	Mézière, N.	French Guiana	Sinnamary; 5.1233611, -52.9553055	
GEODE7773_Gomphidae_Zonophora_batesi	2015-12-01	Mézière, N.	French Guiana	Sinnamary; 5.1233611, -52.9553055	
GEODE7774_Gomphidae_Desmogomphus_tigrivensis	2016-07-03	Mézière, N.	French Guiana	Sinnamary; 5.1233611, -52.9553055	

GEODE7775_Gomphidae_Agriogomphus_sylvicola	2015-12-01	Mézière, N.	French Guiana	Sinnamary; 5.1233611, -52.9553055	
GEODE7776_Libellulidae_Micrathyria_didyma	2015-12-05	Mézière, N.	French Guiana	Kourou	
GEODE7778_Coenagrionidae_Metaleptobasis_bicornis	2016-10-24	Mézière, N.	French Guiana	Iracoubo; 5.489229, -53.303506	
GEODE7779_Calopterygidae_Mnesarete_cupraea	2016-07-16	Mézière, N.	French Guiana	Nouragues; 4.03804, -52.67727	
GEODE7780_Calopterygidae_Hetaerina_caja	2016-06-24	Mézière, N.	French Guiana	Sinnamary; 5.1233611, -52.9553055	
GEODE7781_Coenagrionidae_Phasmoneura_exigua	2016-02-09	Mézière, N.	French Guiana	Sinnamary; 5.1233611, -52.9553055	
GEODE7782_Coenagrionidae_Tuberculobasis_yanomami	2016-07-06	Mézière, N.	French Guiana	Iracoubo; 5.489229, -53.303506	
GEODE7783_Coenagrionidae_Telebasis_demarara	2016-07-06	Mézière, N.	French Guiana	Iracoubo	
GEODE7784_Coenagrionidae_Epipotoneura_nehalennia	2016-07-14	Mézière, N.	French Guiana	Nouragues; 4.083041, -52.679985	
GEODE7788_Dicteriadidae_Heliocharis_amazona	2016-09-25	Mézière, N.	French Guiana	Sinnamary; 5.1233611, -52.9553056	G1-025, male
GEODE7818_Gomphidae_Aphylla_sp	2018-10-17	Mézière, N.	French Guiana	Sinnamary; 5,1233611, -52,95530556	
GEODE7819_Coenagrionidae_Aeolagrion_dorsale	2015-06-26	Mézière, N.	French Guiana	Sinnamary; 5,4176111, -53,04366667	
RMNH.INS.506145_Argiolestidae_Neurolestes_trinervis	2012-02-09	Mézière, N.	Gabon	Ogoué-Lolo, route Koulamoutou-Pana,; -1.30447, 12.50123	
RMNH.INS.506163_Chlorocyphidae_Africocypha_lacuselephantum	2012-04-07	Mézière, N.	Gabon	NGOUNIE, route Mouila-Mimongo,; -1.72887, 11.30718	
RMNH.INS.508794_Platycnemididae_Stenocnemis_pachystigma	2013-03-24	Mézière, N.	Gabon	NGOUNIE, route de Koulamoutou-Mimongo, MAVANGA; -1.41428, 14.14632	

RMNH.INS.508797_Libellulidae_Neodythemis_afra	2013-04-03	Mézière, N.	Gabon	Haut-Ogooué, route Moanda-Francevile, concession Rougier, 2ème Barière, 2ème pont, Moyabi; -1.70565, 13.25595	
RMNH.INS.508833_Chlorocyphidae_Stenocypha_gracilis	2013-09-03	Mézière, N.	Gabon	NGOUNIE, Mbigou, route Mbigou-Moukabou 14km après Mbigou; -1.812, 11.85424	
RMNH.INS.508844_Calopterygidae_Umma_longistigma	2013-09-08	Mézière, N.	Gabon	NGOUNIE, Parc National des monts Birougou, La Louetsie; -1.89434, 12.22619	
RMNH.INS.554426_Gomphidae_Mastigogomphus_chapini	2012-09-29	Mézière, N.	Gabon	Haut-Ogooué, le Clao-centre de loisir, devant l'hotel, OSSKAMA; -1.11259, 13.87533	
RMNH.INS.554510_Gomphidae_Cornigomphus_guineensis	2012-11-18	Mézière, N.	Gabon	Haut-Ogooué, piste à droite avant africa n°1 (direction franceville), Moyabi; -1.68773, 13.31128	
RMNH.INS.508746_Synlestidae_Nubiolestes_diotima	2012-12-31	Mézière, N. & J. Lekogooelle	Gabon	WOLEU-NTEM, route de Kougouleu_Medouneu (Monts de Cristal), ASSSOK; 0.62771, 10.30423	
GEODE7858_Euphaeidae_Cryptophaea_saukra	1992-06-30	Michalski, J.	Thailand	Pi Pau Pau waterfall	
GEODE7864_Platycnemididae_Hylaeargia_magnifica	1994-08-02	Michalski, J.	New Guinea		
OD1316_Aeshnidae_Gynacantha_gracilis	2004-02-23	Miller, K. B.	Costa Rica	Heredia near Puerto Viejo La Selva Biological Station 10°25'N 84°00'W	
GEODE17789_Corduliidae_Procordulia_moroensis	1995-03-25/30	Mohagan, D.	Philippines	Mindanao island/Bukidnon prov. Mt. Katanglad, Impulatao 750-900m	

GEODE7689_Chlorogomphidae_Chlorogomphus_spec	1994-05-02/20	Mohagan, N.	Philippines	Mindoro, Mount Halcon, 1000-1500m	RMNH.ins.1153162
GEODE17911_Gomphidae_Cacoides_latro	2017-08-23	Morris & Wappes	French Guiana	Amazone Nature Lodge, Kaw Rd. #6; 4.550330, -52.170310	
GEODE7687_Euphaeidae_Cyclophaea_cyanifrons	1991-06-05	Muller, R.A.	Philippines	PHILIPPINES, Palawan Id, Iwahig, Balsahan	
GEODE7711_Libellulidae_Celithemis_eponina	2018-07-21	Myrup, A.R.	United States	Lake Champlain, Shelburne Bay S of Burlington; 44.40038, -73.23533	
GEODE7719_Calopterygidae_Calopteryx_aequabilis	2018-07-20	Myrup, A.R.	United States	Poultney River, Cogman Road; 43.62525, -73.37101	
GEODE7730_Lestidae_Archilestes_grandis	2019-09-14	Myrup, A.R.	United States	Kanab Creek, near Hog Canyon; 37.07898 -112.53918	
GEODE7732_Gomphidae_Gomphurus_externus	2015-07-14	Myrup, A.R.	United States	Mona Reservoir below Dam	
GEODE7735_Libellulidae_Ladona_julia	2015-06-26	Myrup, A.R.	United States	Ten Acre Pond; Scotia Range Road; west of State College; 40.80125 -77.94308	
GEODE7736_Gomphidae_Erpetogomphus_compositus	2017-07-27	Myrup, A.R.	United States	Trout Creek; Whitehorse Ranch Road; southeast of Fields (SR 205); 42.16182 -118.43738	
GEODE7738_Libellulidae_Brechmorhoga_mendax	2010-09-12	Myrup, A.R.	United States	North Creek; Kolob Terrace Road; 4.6 miles north of Virgin; 40.35661 -111.57479	
GEODE7740_Aeshnidae_Rhionaeschna_multicolor	2017-07-21	Myrup, A.R.	United States	Kolob Reservoir; north of Virgin; 37.44433 -113.04946	
GEODE7746_Libellulidae_Paltothemis_lineatipes	2017-07-07	Myrup, A.R.	United States	Zion National Park; Kolob Canyon Area; Taylor Creek; 37.463097 -113.198280	

GEODE7747_Corduliidae_Cordulia_shurtleffii	2018-07-13	Myrup, A.R.	United States	Uinta Mountains; pond near Teapot Lake (SR 150); 40.68161 -110.94016	
GEODE7749_Coenagrionidae_Amphiagrion_abbreviatum	2018-06-09	Myrup, A.R.	United States	Kanosh Canyon near mouth (FR106); Cork Creek; 38.77154 -112.39673	
GEODE7754_Gomphidae_Stenogomphurus_rogersi	2015-06-26	Myrup, A.R.	United States	Rothrock State Forest; Beaver Dam Meadows off Pine Swamp Road; south of State College (off SR 26) ; 40.71184 -77.88047	
OD1321_Lestidae_Lestes_disjunctus	2015-07-21	Myrup, A.R.	United States	Utah, Wastach County, Jordanelle Wetlands below dam (SR 32), N of Heber; N40.58161° W111.42781°	
GEODE7708_Libellulidae_Plathemis_subornata	2015-06-09	Myrup, A.R. & M. Clark	United States	UT Hwy 196, 10 miles S of I-80; N40.61401, W112.70823	
GEODE17923_Gomphidae_Merogomphus_longistigma	1981-05-01	Nathan, T.R.S.	India	Mysore State, Agumbe Ghat, Shimoga District	Elevation 2000 ft
GEODE2711_Coenagrionidae_Luzonobasis_glauca	1997-04-20	Nazareno, C.M.	Philippines	Luzon, Apayao Prov. Flora, Santa Maria, Lumon River area	
GEODE7678_Argiolestidae_Luzonargiolestes_baltazarae	1997-05-30	Nazareno, C.M.	Philippines	Luzon, Cagayan prov, Claveria, Brgy Union, 100-500m,	
GEODE17894_Gomphomacromiidae_Gomphomacromia_nodisticta	1974-01-27	Neff, J.L.	Argentina	Catamarca Seep near Minas Capillitas	
GEODE7697_Coenagrionidae_Protoneura_tenuis	2003-12-06	Ogden et al	Peru	Tambopata N.R. Explorer's Inn Tower River Junction	
GEODE7739_Libellulidae_Perithemis_tenera	2003-12-14	Ogden, T.H.	Peru	Puerto Maldonado; 12.401901S, 69.14020	

GEODE7794_Coenagrionidae_Microstigma_anomalum	2003-12-06	Ogden, T.H.	Peru	Tambopata N.R. Explorer's Inn Tower River Junction	
GEODE7868_Platycnemididae_Rhyacocnemis_prothoracica	2004-06-05	Oppel, S.	Papua New Guinea	S 06.72° E 145.08°	
GEODE17604_Austropetaliidae_Austropetalia_patricia	1998-12-10	Paulson, D.R.	Australia	Bridal veil falls area, 2-3km E Blackhealth, 2800-3000'. New South Wales	
GEODE17609_Aeshnidae_Epiaeschna_heros	2005-04-22	Paulson, D.R.	Florida	Liberty County, Sweetwater Creek at Hwy 270	
GEODE17940_Petaluridae_Petalura_litorea	1998-12-20	Paulson, D.R.	Australia	South Queensland, North Stradbroke Island Tortoise Lagoon	Ware et al. 2014
GEODE7832_Coenagrionidae_Caliagrion_billinghursti	1998-12-12	Paulson, D.R.	Australia	Kangaroo Creek, S. of Grays Point	
GEODE7880_Coenagrionidae_Denticulobasis_dunklei	1988-12-26	Paulson, D.R.	Peru	near Shimigay Lake, varzea forest	
GEODE17788_Corduliidae_Procordulia_jacksoniensis	1998-12-09	Paulson, D.R. & N. Smith	Australia	New South Wales Boggy Creek, tributaries and marshy ponds associated with creek on Boggy Creek Road, 9km N Jenolan caves 3600m	
GEODE17884_Pseudocorduliidae_Pseudocordulia_elliptica	1998-12-25	Paulson, D.R. & N. Smith	Australia	Queensland streams on Mount Lewis, NW Julatten 3000ft	
GEODE7701_Libellulidae_Agrionoptera_insignis	1998-12-24	Paulson, D.R. & N. Smith	Australia	Queensland, Swamp 2.1 rd km N Daintree River Crossing on cape tribulation rd.	
GEODE17915_Gomphidae_Peruviogomphus_pearsoni	1971-08-25	Pearson, D.L.	Ecuador	Province Napo, Limoncocha	Lake edge
GEODE17758_Libellulidae_Atoconeura_kenya	1949-06	Pinhey, E.	Kenya	Kenya, Nyeri	
RU123_Lauromacromiidae_Lauromacromia_bedei	2001-09	Pinto, A.	Brazil	MG, P. E. Rio Preto	
RU126_Lauromacromiidae_Lauromacromia_picinguaba	2004-12	Pinto, A.	Brazil	PR, Pontal do Paraná	

RU139_Idomacromiidae_Neocordulia_volxemi	1998-11-01/15	Pinto, A.	Brazil	GO, Mineiros	
GEODE7826_Coenagrionidae_Aceratobasis_macilenta	29495	Plaumann, F.	Brazil		
RMNH.INS.229080_Gomphidae_Macrogomphus_quadratus	2008-03-02	Ragai, R.	Malaysia	Sarawak, Bintulu Division, SPFP, Samarakan, Sg Philip, Samarakan Conservation Area (disturbed and secondary forest);	
RMNH.INS.506492_Platystictidae_Sinosticta_ogatai	2012-05-31	Reels, G.T.	China	Hong Kong, Hong Kong - Ng Tung Chai;	
OD148_Platycnemididae_Platycnemis_pennipes		Rehn A.C.	Europe		male
OD151_Devadattidae_Devadatta_podolestoides		Rehn A.C.	Thailand		male
OD157_Lestoideidae_Lestoidea_conjuncta		Rehn A.C.	Australia		male
OD159_Isostictidae_Isosticta_robustior		Rehn A.C.	New Caledonia		
OD162_Argiolestidae_Caledargiolestes_uniseries		Rehn A.C.	New Caledonia		male
OD1125_Calopterygidae_Neurobasis_ianthinipennis		Rehn, A.	Papua New Guinea		
GEODE17943_Petaluridae_Uropetala_chiltoni	1986-01-24	Reichel, J.	New Zealand	South Island, Canterbury, Lewis Pass, 1000m	Ware et al. 2014
GEODE7765_Corduliidae_Paracordulia_sericea	2016-11-01	Renoult, J.	French Guiana	SA (5) Crique Roche; 3.62672, -53.22751	
GEODE7787_Platystictidae_Palaemnema_brevignoni	2016-07-15	Renoult, J.	French Guiana	Nouragues	
RMNH.5011406_Platycnemididae_Macrocnemis_gracilis	2013-02-11	Richards, S.J.	Papua New Guinea	Bilbilokabip;	
RMNH.5011412_Libellulidae_Microtrigonia_curvata	2013-02-08	Richards, S.J.	Papua New Guinea	Yukfon Creek;	
RMNH.5011413_Libellulidae_Bironides_glochidion	2012-06-29	Richards, S.J.	Papua New Guinea	Purari Camp 9;	

RMNH.5011576_Aeshnidae_Austrogynacantha_heterogena	2012-04-95	Richards, S.J.	Australia	Western Australia, Kununurra Airport;	
RMNH.5011578_Synthemistidae_Metaphya_tillyardi	2011-03-14	Richards, S.J.	Papua New Guinea	near Bat Cave;	
RMNH.5011582_Isostictidae_Tanymecosticta_leptalea	2011-04-13	Richards, S.J.	Papua New Guinea	Tapos Creek;	
RMNH.INS.501979_Coenagrionidae_Thaumatagrion_funereum	2010	Richards, S.J.	Papua New Guinea		
RMNH.INS.501990_Argiolestidae_Pyrrhargiolestes_aulicus	2009	Richards, S.J.	Papua New Guinea		
RMNH.INS.501995_Platycnemididae_Cyanocnemis_aureofrons	2009	Richards, S.J.	Papua New Guinea	Mine Expedition, secret locality;	
RMNH.INS.543752_Argiolestidae_Wahnesia_kirbyi		Richards, S.J.	Papua New Guinea		
GEODE7820_Argiolestidae_Archiargiolestes_pusillissimus	1999-11-28	S. Dunkle	Australia	brushy swamp W of Denmark, and E of the Kent River	
ANCH188_Polythoridae_Miocora_peraltica	2020-06-02	Sanchez Herrera, M.	Colombia	Anchicayá-PNNFarrallones-"La Riqueza"; 3.6094167, -76.8845	
BOY03_Polythoridae_Cora_xanthostoma	2006-09-10	Sanchez Herrera, M.	Colombia	Cundinamarca, Guayabetal, Chirajara. 4.208611; -73.800556	
PP330_Polythoridae_Polythore_procera		Sanchez Herrera, M.	Ecuador	Imbabura	
TM207_Libellulidae_Brechmorhoga_vivax	2016-08-17	Sandoval, J. & C. Bota	Colombia	Parque Nacional Natural Tatamá, Vereda Monte Bello; 5.22855; -76.09826669	
RMNH.INS.505846_Platycnemididae_Arabicnemis_caerulea	2005-05-31	Schneider, W.	Yemen	Wadi Dau'an System, Wadi Mara; 15.143, 48.449	

RMNH.INS.229282_Platycnemididae_Paracnemis_alluaudi	2011	Schuette, K.	Madagascar		
RMNH.INS.500093_Pentaphlebiidae_Pentaphlebia_stahli	2008-05-24	Schuette, K.	Cameroon	Southwest Province, 13 km NW of Loum, Mt Kupe, Nyasoso; 4.8306, 9.6794	
RMNH.INS.500162_Platycnemididae_Allocnemis_contraria	2008-06-13	Schuette, K.	Cameroon	South Province, 26 km E of Campo, Campo-Ma'an National Park (buffer zone), Bitandé River 1km E of Nkoélon; 2.394, 10.054	
GEODE12749_Libellulidae_Celebothemis_delecollei	1983-10-11	Scipio S Parmiono	Sulawesi	Sulawesi, Biseang Labboro	
GEODE17707_Corduliidae_Epitheca_semiaquea	2016-04-02	Shiley, S.	United States	Louisiana Natchitoches Par., Kishatchie National Forest, Anderson Pond,	
GEODE7756_Libellulidae_Pachydiplax_longipennis	2015-06-13	Shuler, B. & A.R. Myrup	United States	Salem; Salem Pond; north end; across UT Hwy 198; 40.05423 -111.67869	
GEODE17713_Corduliidae_Helocordulia_uhleri	2003-06-24	Sibley, F.C.	United States	New York Fulton Town of Johnstown, Peck Lake Outlet 1350ft	
GEODE17725_Libellulidae_Orthemis_macrostigma	2016-02-19	Sibley, F.C.	United States	Virgin Islands: Saint Croix, Southwest sub-district, just Northwest of Subway, small pond; 17 42.569N 64 50.368W	1F. 130'. IORI #: 00082440. Lot #: 000073. EX Fred C Sibley Collection. Florida State Collection of Arthropods. Proc by: Bill Mauffray 4/4/2019

GEODE17904_Gomphidae_Arigomphus_furcifer	2000-06-10	Sibley, F.C.	United States	New York, Orange County, Sterling Forest Lake; lat: 41 10 30N long: 74 16 30W	9:30-12AM. clear, 80's. One of two along edge of small pond. Yale tag 6422. EX International Odonata Research Institute. Florida State Collection of Arthropods. Processed by: Bill Mauffray 2008-12-26. IORI #000043422
GEODE7823_Calopterygidae_Bryoplathanon_globifer	2015-02-20	Sibley, F.C.	Brazil	6.5 miles NW of Camacan. Overflow seep from water tank near end of tower road; -15.384267S, -39.572183W	
GEODE7828_Coenagrionidae_Anisagrion_allopterum	2009-03-26	Sibley, F.C.	Costa Rica	University of Georgia field station, 3 miles SE of Santa Elaena; 10.287833N, -84.7905W	
GEODE7743_Petaluridae_Tanypteryx_hageni	2003-06-21	Skevington, J.	United States	Butté Co., Butter Creek near Cherry Hill Campground; 40.0556N, 121.2947W	Ware et al. 2014
GEODE7792_Synthemistidae_Eusynthemis_nigra	2000-01-01	Skevington, J.	Australia		male
OD63_Lestidae_Austrolestes_annulosus	1999-12-17	Skevington, J.	Australia	WA, Stirling, Range National Park Talyuberlup Peak Hilltop; 34 24' S. 117 57' E.	
GEODE7791_Austrocorduliidae_Hesperocordulia_berthoudi	1999-12-21	Skevington, J. & A.	Australia	Near Wellington Dam; 33.2404S, 115.5723E	

OD1318_Petaluridae_Petalura_gigantea	2000-01-23	Skevington, J. & A.	Australia	New South Wales Washpool NationalPark Wetlan 1.3 km west of campground , entrance road 29°29'57"S 152°19'09"E	
OD77_Libellulidae_Rhyothemis_graphiptera	2000-01-16	Skevington, J. & A.	Australia	Queensland, Bribie Island	
OD81_Synlestidae_Synlestes_weyersii	2000-01-22	Skevington, J. & A.	Australia	New South Wales, Gibralter Range National Park, Suveyors creek; 29 32'17" S. 152 18'38" E.	
OD82_Libellulidae_Brachydiplax_denticauda	2000-01-16	Skevington, J. & A.	Australia	Queensland, Bribie Island	
GEODE7728_Lestidae_Austrolestes_leda	2000-02-03	Skevington, J. & A. & M. Mathieson	Australia	Along River; -28.665 151.940556	
GEODE7737_Synthemistidae_Choristhemis_flavoterminata	2000-02-03	Skevington, J. & A. & M. Mathieson	Australia	Stanthorpe, Along River; 28.3954S, 151.5626E	
GEODE7744_Austrocorduliidae_Micromidia_convergens	2000-02-05	Skevington, J. & A. & M. Mathieson	Australia	Brisbane Forest Park; Scrub Creek; 27.2505S,152.5013E	
GEODE7745_Gomphidae_Armagomphus_armiger	1999-12-21	Skevington, J. & A. & M. Mathieson	Australia	Honeymoon Pool Near Wellington Dam; 33.2246S 115.5613E	
GEODE7755_Synlestidae_Episynlestes_albicauda	2000-03-26	Skevington, J. & A. & M. Mathieson	Australia	Undercliffe Falls; -28.508889 152.172222	

GEODE7790_Gomphidae_Antipodogomphus_acolythus	2000-03-26	Skevington, J. & A. & M. Mathieson	Australia	Clarence River; 28.3807S 152.1700 E	
GEODE7716_Argiolestidae_Griseargiolestes_albescens	2000-01-29	Skevington, J. & D. Reeves	Australia	Stradbroke Island Capembah Creek; S27.28, E153.25	
OD83_Corduliidae_Hemicordulia_australiae	2000-01-26	Skevington, J. & D. Reeves	Australia	Queensland, Stradbroke Island	
OD1310_Austrocorduliidae_Cordulephya_pygmaea	2000-04-02	Skevington, J. & M. Mathieson	Australia	Queensland, Brisbane, Forest Park; Northbrook Creek; 21°18'S 152°41'E	
OD1311_Synthemistidae_Parasynthemis_regina	2000-02-27	Skevington, J. & M. Mathieson	Australia	Queensland, Brisbane, Karawtha Forest	
RMNH.INS.509787_Coenagrionidae_Coenagriocnemis_rufipes	2010-11-09	Skinner, A.	Mauritius		
RMNH.INS.509795_Libellulidae_Thalassothemis_marchali	2010-11-16	Skinner, A.	Mauritius		
RMNH.INS.509800_Coenagrionidae_Coenagriocnemis_insularis	2010-12-20	Skinner, A.	Mauritius		
OD1119_Philosinidae_Rhinagrion_sp		SMB	Vietnam		
OD1120_Perilestidae_Perissolestes_remotus		SMB	Costa Rica		
OD1121_Rhipidolestidae_Agriomorpha_fusca		SMB	Vietnam		
OD1122_Platycnemididae_Prodasineura_croconota		SMB	Vietnam		
OD1123_Platycnemididae_Coeliccia_pyriformis		SMB	Vietnam		
OD1124_Philogeniidae_Philogenia_carrillica		SMB	Costa Rica		

OD1127_Lestidae_Orolestes_octomaculatus		SMB	Vietnam		
OD1169_Gomphomacromiidae_Gomphomacromia_paradoxa	2015-11-29	SMB	Argentina		
OD1170_Calopterygidae_Hetaerina_americana		SMB	United States	Utah, Washington county, Leeds, Leeds creek	
GEODE7769_Libellulidae_Argyrothemis_argentea	1986-10-05	Spangler, P. & O.S. Flint	Brazil	Para, Rio Xingu Camp, CA 60Km, S of Altamira (52.22W, 3.39.S), 1st jungle stream, trail 1, malaise trap	
GEODE7803_Libellulidae_Diastatops_obscura	1913-04	Steinbach, J.	Bolivia	Santa Cruz, Sara province	male
GEODE7822_Argiolestidae_Nesolestes_radama	1990-03-06	Steiner, W.E.	Madagascar	7 km W Ranomafana	
GEODE7638_Libellulidae_Nannophlebia_axiagasta	1970-06-27	Straatman, R.	Indonesia	Joma, Mambare River, 300 m	RMNH.ins.1153178
GEODE17779_Corduliidae_Navicordulia_errans	1980-01-21	Strickman, L.	Paraguay	Itapua San Benito/Pastoreo, flaoting in lake	
GEODE17614_Aeshnidae_Oreaeschna_dictatrix	1935-08-31	Stuber, W.	New Guinea	Cycloop	1000 m
GEODE7643_Synthemistidae_Palaeosynthemis_gracilenta	1931-06	Stüber, W.	Indonesia	NN Guinea 19 Hollandia Humboldt Baai	RMNH.ins.1153203
GEODE7680_Synthemistidae_Palaeosynthemis_cervula	1937-03-06	Stüber, W.	Indonesia	Hollandia, Humboldt Bay, Bewani Hills, Uskwar, 500 m	RMNH.ins.1153205
GEODE7690_Aeshnidae_Plattycantha_cornuta	1932-09	Stüber, W.	Indonesia	Hollandia, Humboldt Bay, Cycloop mountains, 1000m	RMNH.ins.1153206
OD126_Calopterygidae_Vestalis_apicalis	2003-05-30	Svenson, G.	India	Karnataha, UDUPL-Somershkear, Evergreen Forest	
RMNH.INS.503604_Coenagrionidae_Archibasis_viola		Tateh, O.	Malaysia	Sarawak, Bintulu, Tubau, 'new' stream area, swampy section by stream; 3.1277833333, 113.66448333	O. Tateh

RMNH.INS.502077_Argiolestidae_Miniargiolestes_minimus	2010-01-12	Taylor, J.	Australia	Western Australia, Gin Gin,;	
RMNH.INS.502084_Argiolestidae_Archiargiolestes_parvulus	2010-01-12	Taylor, J.	Australia	Western Australia, Gin Gin,;	
GEODE17712_Corduliidae_Helocordulia_selysii	2003-05-01	Tennessen, K.J.	United States	Mississippi Tishomingo Cripple Deer Creek, W of Allsboro, AL nr. Limestone quarry	
GEODE17783_Corduliidae_Neurocordulia_yamaskanensis	2009-07-07	Tennessen, K.J.	United States	Wisconsin Wood Wisconsin River, boat launch off Biron Drive	
GEODE17793_Corduliidae_Neurocordulia_michaeli	2005-07-10	Tennessen, K.J.	Canada	Ontario Renfrew Petawala River, Hwy. 17, Petawawa	
GEODE17936_Gomphidae_Phanogomphus_exilis	1993-04-18	Tennessen, K.J.	United States	Alabama, Clarke County. Choctaw Creek	Hwy. 84, Gosport
GEODE7830_Coenagrionidae_Apanisagrion_lais	2013-05-23	Tennessen, K.J.	United States	Aravaipa Creek; N32.8986, W110.42084	
GEODE7848_Coenagrionidae_Oxyagrion_hermosae	2015-11-25	Tennessen, K.J.	Peru	Junin, Canaan Road to Tina de Perdra,, small stream; 11.46938, -74.55766	
OD976_Calopterygidae_Ormenophlebia_imperatrix	2005-09-21	Tennessen, K.J.	Ecuador	Pastaza Province, Triutary of Rio Anzu, north of Mera; 1 25'5"S 78 3'9"W	1190 m elevation
GEODE7844_Coenagrionidae_Mesamphiagrion_demarmelsi	2014-10-14	Tennessen, K.J. & W. Mauffray	Colombia	Cundinamarca: Sesquilé SE on road to Laguna de Guatavita, , small vegetated farm pond; 04.9934°W, -73.77805°W	
PD166_Petaluridae_Tanypteryx_pryeri	2008	Theischinger, G.	Japan		Ware et al. 2014
RMNH.5011411_Libellulidae_Notolibellula_bicolor	2014-02-01	Theischinger, G.	Australia	Queensland, tributary Isabella Ck, nr. Isabella Falls;	

RMNH.5011415_Gomphidae_Austroepigomphus_praeruptus		Theischinger, G.	Australia	New South Wales,;	
RMNH.INS.504965_Aeshnidae_Acanthaeschna_victoria		Theischinger, G.	Australia	;	
GEODE17902_Synthemistidae_Tonyosynthemis_ofarrelli	1986-01-11	Theischinger, G. & L. Müller	Australia	Queensland Booloumba Creek	
RMNH.INS.500862_Argiolestidae_Podopteryx_selysi	1997-04-06	Theischinger, G. & L. Müller	Australia	Queensland, Palmerston N.P., Along Palmerston Hwy,;	
OD103_Coenagrionidae_Neoneura_mariana		Trapero, A.	Cuba		male
OD105_Coenagrionidae_Protoneura_capillaris		Trapero, A.	Cuba		male
GEODE12748_Libellulidae_Celebophlebia_dactylogastra	1991-05-02	Van Tol, J.	Indonesia	Sulawesi, c. 15 km NNE Malili, c. 150 m asl., 2 32'S 121 12'E	
GEODE7675_Chlorocyphidae_Disparocypha_biedermanni	1993-10-24	Van Tol, J.	Indonesia	Indonesia, C Sulawesi, 40 km N of Woutu: just N of Batas. Small stream nr bridge road Wotu-Tentena at km 4. Distrirbed primary forest; w=2-4m, d=01=0.3, shade. Boulders. 1200m; 2 13'30"S 120 46'30"E	
GEODE7668_Platycnemididae_Calicnemia_pulverulans	1985-08-04	Vick, G.	Nepal	Tarang Marang	
GEODE7882_Coenagrionidae_Andinagrion_peterseni	2006-12-29	Von Ellenrieder, N.	Argentina	Cachi, rio Calchaqui; 25 6'0" S, 66 10'60"W	
OD1281_Megapodagrionidae_Teinopodagrion_meridionale	2012-01-28	Von Ellenrieder, N. & R.W. Garrison	Argentina	Salta, Lesser, Arroyo de piedras sin nombre, along marshy/rocky runnel; 24°40'56"S 65°28'33"W	1426 m elevation

OD102_Calopterygidae_Matrona_basilaris	2003-05-12	Wang, L.J.	Taiwan	Taping	male
OD96_Rhipidolestidae_Rhipidolestes_aculeatus	2003-05-12	Wang, L.J.	Taiwan	Taipei Co., Wanli, Taping	
OD99_Euphaeidae_Bayadera_brevicauda	2003-05-12	Wang, L.J.	Taiwan	Taipei Co., Wanli, Taping	
GEODE7612_Libellulidae_Cannaphila_insularis		Ware, J.	Guyana		
OD1095_Libellulidae_Sympetrum_corruptum		Ware, J.	United States	New Jersey	
OD1158_Aeschnosomatidae_Pentathemis_sp		Ware, JL	Australia		
OD1164_Cordulegastridae_Cordulegaster_sp		Ware, JL	United States		
OD107_Libellulidae_Pantala_flavescens	2003-08-20	Warrick, G. & R. Larsen	United States	New Mexico, Chaves Co., Pecos River east of the scout camp Bitter lake National Wildlife Refuge	male
GEODE7656_Platycnemididae_Nososticta_selysii	1957-03-27	Wegner, AMR	Indonesia	Annanese, Kupang	
GEODE7760_Libellulidae_Austrothemis_nigrescens	1988-02-11	Westfall, M.J. & M.J. Beech	Australia	SE Queensland, North Stradbroke Island	
GEODE7860_Isostictidae_Austrosticta_soror	1997-01-25	Whitesmith, G.	Australia		
OD35_Chlorocyphidae_Rhinocypha_tincta	2001-07-05	Whiting	Papua New Guinea	Chimbu Province, Haia village, Crater Mountain Research area; S06 42.35' E145 016	
OD136_Coenagrionidae_Papuagrion_occipitale	2000-09-21	Whiting, A.S. & M.F.	Papua New Guinea	Sandaun Province, Menakmofib Village; S 04 49.786' E 141 38.126'	male
OD120_Chlorocyphidae_Heliocypha_biseriata	2003-08-16	Whiting, Svenson, Bybee	Malaysia	Sabah, Crocker Range N.P., Inabong substation turnoff @ Penam Pang River	

OD134_Chlorocyphidae_Libellago_semiopaca	2003-08-16	Whiting, Svenson, Bybee	Malaysia	Sabah, Crocher Range N. P., Inabong substation turnoff @ Penepang River	
GEODE17864_Idionychidae_Idionyx_claudia	2013-07-30	Zhang, H.	China	Zhejiang Fuvecun, Jingning co. Lishui City	
GEODE17876_Macromidiidae_Macromidia_kelloggi	2013-07-30	Zhang, H.	China	Zhejiang Fuyecun, Lishui, Jingning County	
GEODE17903_Gomphidae_Anisogomphus_anderi	2009-08-13	Zhang, H.	China	Qinshuigu in Nanling National reserve, Ruyuan County, Shaoguan City, N. Guangdong Province; 24° 55'N 113° 01'E	Zhang Haomiao world odonata collection. 750 m
GEODE17913_Gomphidae_Fukienogomphus_promineus	2010-05-15	Zhang, H.	China	Yadongxi, Conghua City, Guangdong Province, China; 23°41'-23°42' N 113°43'-113°49' E	1 Male. 500 m. Zhang Haomiao World Odonata Collection
GEODE17914_Gomphidae_Shaogomphus_postocularis	2006-06-19	Zhang, H.	China	Mudanjiang City, Heillongjiang Province, Lowland Marsh Ponds	Zhang Haomiao World Odonata Collection
GEODE17919_Gomphidae_Gastrogomphus_abdominalis	2003-04-15	Zhang, H.	China	Anhui Province. Nanyanhu, Hefei City	
GEODE17922_Gomphidae_Melligomphus_ardens	2009-05-15	Zhang, H.	China	Yadongxi, Conghua City, Guangdong Province; 23 41' N, 113 43' E	
GEODE17933_Gomphidae_Scalmogomphus_falcatus	2013-06-17	Zhang, H.	China	Yunnan Province, Yangbi County, Dali, Yangbijiang River	Ex Haomiao Zhang's Odonata Collection. Carl Cook Collection. Florida State Collection of Arthropods
GEODE7871_Platystictidae_Sinosticta_sylvatica	2009-04-27	Zhang, H.	China		

GEODE7872_Priscagrionidae_Sinocnemis_henanensis	2012-07-20	Zhang, H.	China	Shennonjia National Reserve	
OD1079_Philogangidae_Philoganga_vetusta	2014-06-20	Zhang, H.	China	Hainan, Wuzhishan, Shuiman, stream; 109.661628, 18.850304	730 m elevation
RMNH.INS.502064_Rhipidolestidae_Rhipidolestes_truncatidens	2009-05-21	Zhang, H.	China	N. Guadong, Nanling Reserve,;	
RMNH.INS.508447_Libellulidae_Palpopleura_sexmaculata	2011-10-07	Zhang, H.	China	Guangdong, Conghua city, Weidong;	
RMNH.INS.508515_Libellulidae_Nannophyopsis_clara	2010-05-04	Zhang, H.	China	Guangdong, Huizhou City, Ruhu;	
RMNH.INS.508528_Chlorogomphidae_Watanabeopetalia_usignata	2011-07-22	Zhang, H.	China	Guizhou, Guiyang City, Xiangzhigou;	
RMNH.INS.508607_Calopterygidae_Mnais_mneme	2011-05-18	Zhang, H.	China	Guangdong, Conghua City, Foot of Nankunshan, Conghua village;	
RMNH.INS.508616_Calopterygidae_Atrocalopteryx_melli	2011-08-08	Zhang, H.	China	Guangdong, Shaoguan City, Shixing County, Chebaling Reserve;	
RMNH.INS.508632_Synlestidae_Megalestes_heros	2009-05-21	Zhang, H.	China	Guangdong, Shaoguan City, Ruyuan, Nanling Reserve;	
RMNH.INS.508637_Platycnemididae_Indocnemis_orang	2009-09-17	Zhang, H.	China	Guangdong, Shaoguan City, Ruyuan, Nanling Reserve;	
RMNH.INS.508661_Gomphidae_Lamelligomphus_camelus	2009-07-27	Zhang, H.	China	Guangdong, Huizhou City, Nankunshan;	
RMNH.INS.508662_Gomphidae_Melligomphus_sinicus	2011-06-20	Zhang, H.	China	Guangdong, Huizhou City, Nankunshan;	
RMNH.INS.508663_Gomphidae_Nihonogomphus_semanticus	2011-05-27	Zhang, H.	China	Guangdong, Huizhou City, Nankunshan;	

RMNH.INS.508672_Gomphidae_Nepogomphus_walli	2009-09-01	Zhang, H.	China	Yunnan, Xishbuangbanna, Mengla, Buguzhai;	
RMNH.INS.508674_Calopterygidae_Archineura_incarnata	2011-05-12	Zhang, H.	China	Guangdong, Huizhou City, Nankunshan;	
RMNH.INS.508691_Gomphidae_Asiagomphus_hainanensis	2011-5-20	Zhang, H.	China	Guangdong, Conghua City, Yingcun, foot of Nankunshan;	
RMNH.INS.508700_Gomphidae_Labrogomphus_torvus	2011-08-07	Zhang, H.	China	Guangdong, Conghua City, Yadongxi;	
RMNH.INS.509134_Gomphidae_Euthygomphus_koxingai	2009-04-18	Zhang, H.	China	Hainan, Xinglong County, Nanling;	
RMNH.INS.509144_Gomphidae_Dubitogomphus_bicornutus	2006-08-27	Zhang, H.	China	Beijing, Beijing, Yunmenshan;	
RMNH.INS.509145_Aeshnidae_Cephalaeschna_obversa	2011-08-02	Zhang, H.	China	Guizhou, Guiyang City, Xiangzhigou;	
RMNH.INS.509146_Gomphidae_Sinictinogomphus_clavatus	2011-08-03	Zhang, H.	China	Guizhou, Guiyang City, Qianlingshan;	
GEODE17704_Corduliidae_Epitheca_marginata	2004-04-15	Zhaohui, L.	China	Jiangsu Mt. Yousi, Goochin	
GEODE7634_Gomphidae_Sinogomphus_telamon	1943-07-25	Zu, C.S.	China	Chung-an hsien Hsien-fen-Lin, Fukien (Fujian?)	
GEODE12753_Gomphidae_Lindenia_tetraphylla	1987-07-15		Greece	Thessaloniki, Lake Volvi, 37m	
GEODE12755_Gomphidae_Perissogomphus_stevensi	1986-06-02		Nepal	Upper Mai Valley, Pualthola, 3300ft	
GEODE17755_Libellulidae_Anatya_januaria	1981-03-21		Brazil	Sao Paulo; San Carlos	1m
GEODE17909_Gomphidae_Zonophora_diversa	1999-01-30		Argentina	Misiones: Iguazo Department, Iguazo	1F. IORI #: 00086255. Lot #: 000075. Ex Carl Cook Collection. Florida State Collection of Arthropods. Proc by: Bill

					Mauffray 5/5/2020
GEODE17929_Libellulidae_Scapanea_frontalis	1982-04-23		Jamaica	Blue Mountain	
GEODE2756_Euphaeidae_Heterophaea_barbata	1997-04-02		Philippines		
GEODE7650_Libellulidae_Diplacina_spec	1953-09-14		Indonesia	Laiwui-Obi	RMNH.ins.1153160
GEODE7653_Platycnemididae_Esme_mudiensis	1963-05		India	Madras State, Anamalai Hills, Kadamparai, 3500 feet	
GEODE7655_Libellulidae_Diplacina_persephone	1936-02-12/19		Indonesia	North New Guinea, Hollandia, Humboldt Bay, Nonno mountains, 400 m	RMNH.ins.1153180
GEODE7659_Libellulidae_Microtrigonia_gomphoides	1931		Indonesia	North New Guinea, Hollandia, Humboldt Bay, Cycloop mountains, 400 m	RMNH.ins.1153175
GEODE7660_Platycnemididae_Nososticta_exul	1951-09-12		Indonesia	Isl. Halmahera, Akilamo 50-100m	
GEODE7666_Chlorocyphidae_Heliocypha_bisignata	1961-04		India	Nilgiri Hills, Devala, 3200 ft, PSM	
GEODE7672_Chlorocyphidae_Cyrano_unicolor	1936-06-21		Phillipines	Mount Makiling	
GEODE7673_Platycnemididae_Calicnemia_erythromelas	1998-04-21		Thailand	Chiang Mai, Doi Inthanon, streams at checkpoint 2 trail, 1500m	
GEODE7676_Libellulidae_Misagria_calverti	1966-03-04		Suriname	Compagnie Kreek	RMNH.ins.517922
GEODE7686_Euphaeidae_Euphaea_cardinalis	1963-05		India	SOUTH INDIA, Madras state, Anamalai hills, Kadamparai, 3500 ft, P.S.N.	
GEODE7694_Euphaeidae_Euphaea_refulgens	1990-08-05		Philippines	Philippines, Luzon Id, Laguna Prov., Pagasanjan Falls	
GEODE7759_Libellulidae_Nannophya_australis			Australia	Stradbroke Island	

GEODE7815_Libellulidae_ Urothemis_assignata	2010-03-13		Gabon	8 Km from Cap Esterias to Libreville; 0.586042, 9.335804	
GEODE7829_Coenagrionidae_Antiagrion_grinbergsi	1988-01-01		Chille		
GEODE7841_Coenagrionidae_Peristicta_aeneoviridis	2015-02-19		Brazil	Rio Panela, 2.3 miles W of Camacan and east of large pond; 15 25.039S, 39 31.992W	
OD1077_Mesopodagrionidae_Mesopodagrion_sp	2015-05-31		China	Yunnan, Lu Chun, parque do totem touro	1884 m elevation
OD1091_Libellulidae_Brachythemis_contaminata			Vietnam		
OD1094_Libellulidae_Nannophlebia_alexia			Indonesia		
OD1101_Macromiidae_Epophthalmia_elegans			China	Hong Kong	
OD1106_Libellulidae_Zygonyx_flavicosta			Senegal		
OD1116_Coenagrionidae_Cyanallagma_sp			Argentina		
OD1280_Priscagrionidae_Priscagrion_kiautai	2016-05-27		Vietnam	Tam Dao; 21.465 N 105.632 E	
RMNH.INS.505717_Polythoridae_Chalcopteryx_rutilans	2010		Peru		
SRR921569_Ephemeroptera_Baetis_sp	2011-05-17		Germany	North Rhine-Westphalia, Rhein-Sieg-Kreis, Donrath, Agger	
SRR921591_Ephemeroptera_Ephemera_danica	2011-05-17		Germany	North Rhine-Westphalia, Rhein-Sieg-Kreis, Donrath, Agger	
SRR921596_Ephemeroptera_Eurylophella_sp	2012-02-19		United States	Pennsylvania, Cumberland County, King's Gap State Park	
SRR921608_Ephemeroptera_Isonychia_bicolor	2011-10-31		United States	New Jersey, Hunterton County, Muscanetcong River and North Mountain Road	

Supplemental Table 1. Locality information for the taxa included in this study.

Tree Node	Fossil species	Fossil Age/ Prior lower bound	Prior upper bound	Accession number	Publication	Phylogenetic justification	Age justification for the bed/fossil
Paleoptera (Root)	*Erasipteroides valentini*	319.9	412	see Kohli et al. (2021)	see Kohli et al. (2021)	see Kohli et al. (2021)	see Kohli et al. (2021)
Odonata	*Triassolestodes asiaticus*	228.4	319.9	see Kohli et al. (2021)	see Kohli et al. (2021)	see Kohli et al. (2021)	see Kohli et al. (2021)
Epiprocta	*Liassophlebia sp.*	199.3	319.9	see Kohli et al. (2021)	see Kohli et al. (2021)	see Kohli et al. (2021)	see Kohli et al. (2021)
Anisoptera	*Sinacymatophlebia mongolica*	158.1	319.9	see Kohli et al. (2021)	see Kohli et al. (2021)	see Kohli et al. (2021)	see Kohli et al. (2021)
Zygoptera	*Mersituria ludmilae*	71.9	319.9	see Kohli et al. (2021)	see Kohli et al. (2021)	see Kohli et al. (2021)	see Kohli et al. (2021)
Aeshnidae	*Gomphaeschna inferna*	71.9	319.9	see Kohli et al. (2021)	see Kohli et al. (2021)	see Kohli et al. (2021)	see Kohli et al. (2021)

Anax	*Anax cryptus*	5.6	319.9	Monte Castellaro working Collection of Gentilini No. 1219	Gentilini and Peters 1993, The Upper Miocene aeshnids of Monte Castellaro,	from the Getilini and Peters (1)anterior part of Arc shorter than its posterior segment ,(2)RP diverging near the midst of the anterior part of Arc, (3) MA-fork and the conjunction of its anterior branch with RP3-4 very distinct, (4)Mspl and Rspl deeply arched, (5)between IR2 and Rspl some distinct slanting rows of rarely less than 5cells, (6) IR2 unforked (its pseudo fork starting beneath Pt embraces only 2 rows of cells),(7) RP distinctly curved towards Pt(Fig.4a),(8) A2 in hind wing running parallel to A3	roveri et al (2008) - Recent advancements in the Messinian stratigraphy of Italy and their Mediterranean-scale implications Fossils are from the Gessoso-solfifera Formation

Gomphidae	*Proterogomphus renateae*	150	319.9	see Kohli et al. (2021)	see Kohli et al. (2021)	see Kohli et al. (2021)	see Kohli et al. (2021)
Lindeniinae sensu our tree	*Cratolindenia knuepfae*	122.5	319.9	SMNS 64414, an exoskeleton	G. Bechly. 2000. Two new fossil dragonfly species (Insecta: Odonata: Anisoptera: Araripegomphidae and Lindeniidae) from the Crato Limestone (Lower Cretaceous, Brazil). Stuttgarter Beiträge zur Naturkunde Serie B (Geologie und Paläontologie) 296:1-16		lacustrine - large; lithified carbonate, Crato Formation of Brazil
Erpetogomphus + Nihonogomphus	*Erpetogomphus shii*	23	319.9	RX001, an exoskeleton	D. R. Zheng, A. Nel, E. A. Jarzembowski, S. C. Chang, H. C. Zhang and B. Wang. 2018. Exceptionally well-preserved dragonflies (Insecta: Odonata) in		Mexican/Chiapas amber

					Mexican amber. Alcheringa 43:157-164		
Petaluridae	*Argentinopetala*	125	319.9	MLP 12887, a wing	J. F. Petrulevičius and A. Nel. 2003. Oldest Petalurid dragonfly (Insecta: Odonata): a Lower Cretaceous specimen from south Patagonia, Argentina. Cretaceous Research 24:31-34		Aptial lacustrine
Cavilabiata	*Juralibellula ningchengensis*	158.1	319.9	see Kohli et al. (2021)	see Kohli et al. (2021)	see Kohli et al. (2021)	see Kohli et al. (2021)
Chlorogomphidae + Cordulegasteridae	*Araripechlorogomphus muratai*	112.6	319.9	see Kohli et al. (2021)	see Kohli et al. (2021)	see Kohli et al. (2021)	see Kohli et al. (2021)
Libellulidae	*Tauriphila? cerestensis*	29.2	319.9	see Kohli et al. (2021)	see Kohli et al. (2021)	see Kohli et al. (2021)	see Kohli et al. (2021)

Libellulinae sensu our tree	Libellula requieniana	28.1	319.9	Fossil ID Number: 49440	H. Nicolas. 1890. Insectes fossiles d'Aix - collection du Muséum Requien, à Avignon. Compte Rendus, Association Française pour l'Avancement des Sciences 18:424-432		Chattian lacustrine - large limestone in the Niveau du gypse d'Aix Formation of France.
Sympetrum + Celithemis	Sympetrum bigoti n. sp	28.1	319.9	MNHN-LP-R.07452	A. Nel and M. Papazian. 1985. Description d'une nouvelle espèce d'odonate fossile du Stampien d'Aix-en-Provence (Odon., Libellulidae). Entomologica Gallica 1:351-355		Chattian lacustrine, 'Aix Formation of France.
Cordullidae	*Croatocordulia platyptera*	12.7	319.9	see Kohli et al. (2021)	see Kohli et al. (2021)	see Kohli et al. (2021)	see Kohli et al. (2021)
Libellulidae + Cordullidae	*Palaeolibellula zherikhini*	89.5	319.9	see Kohli et al. (2021)	see Kohli et al. (2021)	see Kohli et al. (2021)	see Kohli et al. (2021)

Macromiidae	*Epophtalmia biordinata*	15.5	319.9	see Kohli et al. (2021)	see Kohli et al. (2021)	see Kohli et al. (2021)	see Kohli et al. (2021)
Ephemeroptera - Baetis sp.	*Australiphemera revelata*	112.6	319.9	see Kohli et al. (2021)	see Kohli et al. (2021)	see Kohli et al. (2021)	see Kohli et al. (2021)
Calopterygidae + Chlorocyphidae	*Sinocalopteryx shangyongensis*	47.8	319.9	see Kohli et al. (2021)	see Kohli et al. (2021)	see Kohli et al. (2021)	see Kohli et al. (2021)
Calopterygid Complex	*Sapho' sannoisiensis*	23	319.9	see Kohli et al. (2021)	see Kohli et al. (2021)	see Kohli et al. (2021)	see Kohli et al. (2021)

Supplemental Table 2. Fossil calibrations for our divergence time estimation analysis.

Sample ID	Aspect Ratio, L/W (Hindwing)	Aspect Ratio, L/W (Forewing)	Flight Behavior	AHE Loci Recovered
**Outgroups**				
SRR1695233_Ephemeroptera_Ecdyonurus_insignis	?	?	Flyer	837
SRR921569_Ephemeroptera_Baetis_sp.	?	?	Flyer	831
SRR921591_Ephemeroptera_Ephemera_danica	?	?	Flyer	784
SRR921596_Ephemeroptera_Eurylophella_sp.	?	?	Flyer	840
SRR921608_Ephemeroptera_Isonychia_bicolor	?	?	Flyer	821
**Aeschnosomatidae**				
GEODE7682_Aeschnosomatidae_Aeschnosoma_forcipula	3.06862745	4.02469136	Flyer	464
OD1158_Aeschnosomatidae_Pentathemis_sp.	3.00760456	3.87614679	Flyer	920
**Aeshnidae**				
GEODE17607_Aeshnidae_Andaeschna_rufipes	3	3.61157025	Flyer	906
GEODE17609_Aeshnidae_Epiaeschna_heros	3.28551856	4.27299312	Flyer	921
GEODE17610_Aeshnidae_Gomphaeschna_furcillata	3.09114895	4.08643831	Flyer	898
GEODE17614_Aeshnidae_Oreaeschna_dictatrix	3.3037037	3.96774194	Flyer	433
GEODE7669_Aeshnidae_Amphiaeschna_ampla	3.26811594	4.12820513	Flyer	241
GEODE7690_Aeshnidae_Plattycantha_cornuta	3.42105263	4.1875	Flyer	868

GEODE7691_Aeshnidae_Heliaeschna_crassa	3.15217391	3.90973872	Flyer	823
GEODE7740_Aeshnidae_Rhionaeschna_multicolor	3.02463576	4.10236387	Flyer	928
OD1303_Aeshnidae_Gynacanthaeschna_sikkima	2.76521739	4.08235294	Flyer	908
OD1316_Aeshnidae_Gynacantha_gracilis	3.13251303	3.98538244	Flyer	911
OD830_Aeshnidae_Anax_walsinghami	3.14263946	4.20123894	Flyer	912
RMNH.5011576_Aeshnidae_Austrogynacantha_heterogena	2.82727273	3.79069767	Flyer	922
RMNH.INS.502232_Aeshnidae_Gynacantha_victoriae	3.13251303	3.98538244	Flyer	925
RMNH.INS.504908_Aeshnidae_Telephlebia_godeffroyi	3.0647821	3.92034139	Flyer	912
RMNH.INS.504941_Aeshnidae_Notoaeschna_sagittata	3.32380952	4.22727273	Flyer	927
RMNH.INS.504965_Aeshnidae_Acanthaeschna_victoria	3.3167	4.17	Flyer	922
RMNH.INS.505016_Aeshnidae_Anax_imperator	3.14263946	4.20123894	Flyer	922
RMNH.INS.505017_Aeshnidae_Isoaeshna_isoceles	2.977011494	3.591549296	Flyer	927
RMNH.INS.506912_Aeshnidae_Indaeschna_grubaueri	3.58091067	4.60354665	Flyer	910
RMNH.INS.507402_Aeshnidae_Brachytron_pratense	3.27956989	4.16455696	Flyer	937
RMNH.INS.507528_Aeshnidae_Boyeria_cretensis	3.02385021	3.94327694	Flyer	916
RMNH.INS.509145_Aeshnidae_Cephalaeschna_obversa	3.19683482	4.09069212	Flyer	930
TM170_Aeshnidae_Remartinia_luteipennis	3.29172714	4.26585366	Flyer	904
TM392_Aeshnidae_Rhionaeschna_joannisi	3.02463576	4.10236387	Flyer	906
TM581_Aeshnidae_Andaeschna_occidentalis	3	3.61157025	Flyer	885
**Amanipodagrionidae**				
OD1133_Amanipodagrionidae_Amanipodagrion_gilliesi	4.175	4.658536585	Percher	924
**Amphipterygidae**				
OD1148_Amphipterygidae_Amphipteryx_agrioides	5.17318355	5.41741123	Percher	920
GEODE7678_Argiolestidae_Luzonargiolestes_baltazarae	3.71134021	4.30681818	Percher	913
GEODE7716_Argiolestidae_Griseargiolestes_albescens	5.58536585	5.02272727	Percher	930

GEODE7820_Argiolestidae_Archiargiolestes_pusillissimus	4.2631579	4.28947368	Percher	889
GEODE7821_Argiolestidae_Eoargiolestes_ochraceus	4.409836066	5.107142857	Percher	920
GEODE7822_Argiolestidae_Nesolestes_radama	4.538461538	5.294117647	Percher	832
OD1083_Argiolestidae_Austroargiolestes_icteromelas	4.56	5.10416667	Percher	919
OD1309_Argiolestidae_Nesolestes_sp.	4.538461538	5.294117647	Percher	919
OD162_Argiolestidae_Caledargiolestes_uniseries	4.28070175	4.72222222	Percher	923
RMNH.5008334_Argiolestidae_Podolestes_orientalis	4.8	4.71428571	Percher	921
RMNH.INS.500397_Argiolestidae_Allolestes_maclachlanii	4.78571429	4.80952381	Percher	937
RMNH.INS.500853_Argiolestidae_Eoargiolestes_ochraceus	4.409836066	5.107142857	Percher	929
RMNH.INS.500854_Argiolestidae_Trineuragrion_percostale	5.56410256	5.45454546	Percher	939
RMNH.INS.500862_Argiolestidae_Podopteryx_selysi	3.773809524	4.15	Percher	924
RMNH.INS.501973_Argiolestidae_Argiolestes_muller	4.49748744	4.5	Percher	894
RMNH.INS.501990_Argiolestidae_Pyrrhargiolestes_aulicus	4.783783784	5.057142857	Percher	925
RMNH.INS.502075_Argiolestidae_Metagrion_ornatum	4.594594595	4.882352941	Percher	913
RMNH.INS.502077_Argiolestidae_Miniargiolestes_minimus	3.697183099	4.344827586	Percher	921
RMNH.INS.502084_Argiolestidae_Archiargiolestes_parvulus	4.2631579	4.28947368	Percher	917
RMNH.INS.503407_Argiolestidae_Caledopteryx_sarasini	4.28070175	4.72222222	Percher	930
RMNH.INS.506145_Argiolestidae_Neurolestes_trinervis	4.2	4.796610169	Percher	929
RMNH.INS.543752_Argiolestidae_Wahnesia_kirbyi	4.369565217	4.511111111	Percher	926
**Austrocorduliidae**				
GEODE17873_Austrocorduliidae_Lathrocordulia_metallica	3.03888889	3.84137931	Flyer	852
GEODE7744_Austrocorduliidae_Micromidia_convergens	2.88059702	3.62545455	Flyer	928

GEODE7791_Austrocorduliidae_Hesperocordulia_berthoudi	3.06713781	3.75431035	Flyer	933
OD1172_Austrocorduliidae_Austrocordulia_refracta	2.70097508	3.89970502	Flyer	918
OD1310_Austrocorduliidae_Cordulephya_pygmaea	3.38071066	3.98342541	Flyer	925
GEODE17604_Austropetaliidae_Austropetalia_patricia	3.06734694	4.15196078	Flyer	907
OD1112_Austropetaliidae_Phyllopetalia_apollo	3.07142857	4.34444444	Flyer	900
OD1115_Austropetaliidae_Hypopetalia_pestilens	3.41	4.453488372	Flyer	904
**Calopterygidae**				
GEODE7705_Calopterygidae_Noguchiphaea_yoshikoae	3.58888889	4.13953488	Percher	937
GEODE7719_Calopterygidae_Calopteryx_aequabilis	3.09200971	3.18772535	Percher	937
GEODE7721_Calopterygidae_Neurobasis_chinensis	2.36216216	2.99481865	Percher	934
GEODE7748_Calopterygidae_Mnesarete_williamsoni	4.6712609	4.44223916	Percher	928
GEODE7779_Calopterygidae_Mnesarete_cupraea	4.6712609	4.44223916	Percher	931
GEODE7780_Calopterygidae_Hetaerina_caja	4.2421347	4.23470057	Percher	932
GEODE7823_Calopterygidae_Bryoplathanon_globifer	6.40384615	4.46052632	Percher	919
OD102_Calopterygidae_Matrona_basilaris	5	4.73913044	Percher	927
OD1125_Calopterygidae_Neurobasis_ianthinipennis	2.36216216	2.99481865	Percher	922
OD1170_Calopterygidae_Hetaerina_americana	4.2421347	4.23470057	Percher	925
OD126_Calopterygidae_Vestalis_apicalis	3.76712329	3.32467533	Percher	919
OD976_Calopterygidae_Ormenophlebia_imperatrix	4.3255814	6.43137255	Percher	924
RMNH.INS.501070_Calopterygidae_Echo_modesta	4.75272	1.31859	Percher	932
RMNH.INS.502054_Calopterygidae_Caliphaea_confusa	4.13333333	5.19607843	Percher	929
RMNH.INS.502132_Calopterygidae_Phaon_iridipennis	3.14	3.60674157	Percher	908
RMNH.INS.503116_Calopterygidae_Sapho_fumosa	2.95192308	4.42666667	Percher	905
RMNH.INS.505747_Calopterygidae_Vestalaria_smaragdina	?	?	Percher	931
RMNH.INS.505806_Calopterygidae_Psolodesmus_kuroiwae	3.35643564	3.03738318	Percher	924

RMNH.INS.507722_Calopterygidae_Matronoides_cyaneipennis	2.5	2.740740741	Percher	936
RMNH.INS.507799_Calopterygidae_Vestalis_amaryllis	3.76712329	3.32467533	Percher	928
RMNH.INS.508607_Calopterygidae_Mnais_mneme	3.52380952	3.78313253	Percher	927
RMNH.INS.508616_Calopterygidae_Atrocalopteryx_melli	3.175	3.275	Percher	930
RMNH.INS.508674_Calopterygidae_Archineura_incarnata	3.12195122	3.583333333	Percher	926
RMNH.INS.508844_Calopterygidae_Umma_longistigma	3.34375	2.11392405	Percher	921
**Chlorocyphidae**				
GEODE7666_Chlorocyphidae_Heliocypha_bisignata	5.35164835	5.47777778	Percher	707
GEODE7667_Chlorocyphidae_Libellago_greeni	5.08204604	5.40104104	Percher	241
GEODE7672_Chlorocyphidae_Cyrano_unicolor	5.13414634	6.10588235	Percher	885
GEODE7675_Chlorocyphidae_Disparocypha_biedermanni	?	6	Percher	891
GEODE7812_Chlorocyphidae_Heliocypha_perforata	5.35164835	5.47777778	Percher	916
OD120_Chlorocyphidae_Heliocypha_biseriata	5.35164835	5.47777778	Percher	910
OD134_Chlorocyphidae_Libellago_semiopaca	5.08204604	5.40104104	Percher	913
OD35_Chlorocyphidae_Rhinocypha_tincta	4.72284237	5.18092519	Percher	898
RMNH.INS.500989_Chlorocyphidae_Libellago_aurantiaca	5.08204604	5.40104104	Percher	910
RMNH.INS.501024_Chlorocyphidae_Sundacypha_petiolata	5.51428571	5.58823529	Percher	917
RMNH.INS.505345_Chlorocyphidae_Platycypha_caligata	5.37837838	5.94594595	Percher	934
RMNH.INS.505736_Chlorocyphidae_Aristocypha_fenestrella	4.03311258	4.45683453	Percher	914
RMNH.INS.506163_Chlorocyphidae_Africocypha_lacuselephantum	4.79032258	5.27472528	Percher	918
RMNH.INS.507707_Chlorocyphidae_Rhinoneura_villosipes	5.941176471	5.977777778	Percher	918
RMNH.INS.508833_Chlorocyphidae_Stenocypha_gracilis	4.866666667	5.066666667	Percher	904
GEODE7689_Chlorogomphidae_Chlorogomphus_spec	3.21713442	3.93727915	Flyer	888

OD1315_Chlorogomphidae_Chlorogomphus_auratus	3.21713442	3.93727915	Flyer	916
RMNH.INS.508528_Chlorogomphidae_Watanabeopetalia_usignata	3.07377049	4.05699482	Flyer	926
**Coenagrionidae**				
GEODE2711_Coenagrionidae_Luzonobasis_glauca	4.9	4.806451613	Percher	921
GEODE2758_Coenagrionidae_Plagulibasis_ciliata	5.018518519	5.185185185	Percher	882
GEODE7697_Coenagrionidae_Protoneura_tenuis	6.09858882	6.14693175	Percher	924
GEODE7725_Coenagrionidae_Mecistogaster_linearis	5.01721181	5.09584894	Percher	936
GEODE7741_Coenagrionidae_Psaironeura_tenuissima	5.8	6.32142857	Percher	934
GEODE7749_Coenagrionidae_Amphiagrion_abbreviatum	4.45709029	4.86942915	Percher	932
GEODE7753_Coenagrionidae_Telebasis_salva	4.51893154	4.77704847	Percher	926
GEODE7758_Coenagrionidae_Acanthagrion_adustum	5.00740769	5.200566	Percher	936
GEODE7778_Coenagrionidae_Metaleptobasis_bicornis	5.05128205	5.21008403	Percher	926
GEODE7781_Coenagrionidae_Phasmoneura_exigua	4.97142857	4.97368421	Percher	934
GEODE7782_Coenagrionidae_Tuberculobasis_yanomami	4.59499091	4.79134056	Percher	935
GEODE7783_Coenagrionidae_Telebasis_demarara	4.51893154	4.77704847	Percher	928
GEODE7784_Coenagrionidae_Epipotoneura_nehalennia	4.85714286	6.47826087	Percher	934
GEODE7785_Coenagrionidae_Inpabasis_rosea	4.65789474	4.72972973	Percher	932
GEODE7786_Coenagrionidae_Acanthallagma_luteum	3.097560976	3.536585366	Percher	933
GEODE7794_Coenagrionidae_Microstigma_anomalum	5.21875	3.85087719	Percher	928
GEODE7819_Coenagrionidae_Aeolagrion_dorsale	5.00740769	5.200566	Percher	939
GEODE7826_Coenagrionidae_Aceratobasis_macilenta	4.82352941	5.14285714	Percher	721
GEODE7827_Coenagrionidae_Amphiallagma_parvum	4.125	3.85185185	Percher	916

GEODE7828_Coenagrionidae_Anisagrion_allopterum	4.56816143	4.45938477	Percher	924
GEODE7829_Coenagrionidae_Antiagrion_grinbergsi	4.68	4.82	Percher	259
GEODE7830_Coenagrionidae_Apanisagrion_lais	4.1875	4.5625	Percher	930
GEODE7832_Coenagrionidae_Caliagrion_billinghursti	5.16	5.06	Percher	513
GEODE7833_Coenagrionidae_Calvertagrion_mauffrayi	4.27272727	4.26666667	Percher	898
GEODE7835_Coenagrionidae_Cyanallagma_angelae	4.853258949	4.970763881	Percher	926
GEODE7836_Coenagrionidae_Diceratobasis_melanogaster	3.26025	0.59409	Percher	770
GEODE7839_Coenagrionidae_Telagrion_longum	5.24242424	5.18181818	Percher	883
GEODE7841_Coenagrionidae_Peristicta_aeneoviridis	4.54545455	4.78787879	Percher	911
GEODE7844_Coenagrionidae_Mesamphiagrion_demarmelsi	4.76481578	4.94230104	Percher	929
GEODE7848_Coenagrionidae_Oxyagrion_hermosae	4.81818182	5.13333333	Percher	933
GEODE7852_Coenagrionidae_Homeoura_lindneri	4.11764706	4.41176471	Percher	933
GEODE7854_Coenagrionidae_Leptobasis_melinogaster	5.00437769	5.03012908	Percher	900
GEODE7857_Coenagrionidae_Platystigma_astictum	4.106870229	4.479674797	Percher	717
GEODE7879_Coenagrionidae_Forcepsioneura_sancta	6.625	5.75	Percher	900
GEODE7880_Coenagrionidae_Denticulobasis_dunklei	2.66616	0.59409	Percher	822
GEODE7882_Coenagrionidae_Andinagrion_peterseni	4.09375	4.54545455	Percher	908
OD103_Coenagrionidae_Neoneura_mariana	4.85860205	4.87945136	Percher	934
OD105_Coenagrionidae_Protoneura_capillaris	6.09858882	6.14693175	Percher	924
OD1116_Coenagrionidae_Cyanallagma_sp.	4.853258949	4.970763881	Percher	928
OD136_Coenagrionidae_Papuagrion_occipitale	4.71875	5.328125	Percher	928
RMNH.5007797_Coenagrionidae_Azuragrion_nigridorsum	3.87096774	4.22580645	Percher	936
RMNH.5007810_Coenagrionidae_Africallagma_glaucum	4.33333333	4.5	Percher	937

RMNH.5010307_Coenagrionidae_Proischnura_polychromatica	4.11111111	4.08823529	Percher	937
RMNH.INS.229208_Coenagrionidae_Oreocnemis_phoenix	5.3	6.34285714	Percher	928
RMNH.INS.500398_Coenagrionidae_Leptocnemis_cyanops	4.74	4.75	Percher	934
RMNH.INS.501979_Coenagrionidae_Thaumatagrion_funereum	3.465753425	4.084507042	Percher	932
RMNH.INS.503604_Coenagrionidae_Archibasis_viola	5.13953488	5.34117647	Percher	932
RMNH.INS.503619_Coenagrionidae_Aciagrion_borneense	4.5	4.85714286	Percher	932
RMNH.INS.503951_Coenagrionidae_Argiocnemis_rubescens	4.91891892	4.47222222	Percher	931
RMNH.INS.504852_Coenagrionidae_Austrocoenagrion_lyelli	4.957746479	4.753424658	Percher	930
RMNH.INS.505254_Coenagrionidae_Austrocnemis_splendida	4.2631579	4.75	Percher	929
RMNH.INS.507703_Coenagrionidae_Stenagrion_dubium	4.918367347	5.204081633	Percher	927
RMNH.INS.509787_Coenagrionidae_Coenagriocnemis_rufipes	5.86046512	4.97727273	Percher	937
RMNH.INS.509800_Coenagrionidae_Coenagriocnemis_insularis	5.86046512	4.97727273	Percher	935
RMNH.INS.557636_Coenagrionidae_Pericnemis_stictica	5.612155388	5.539057841	Percher	929
TM305_Coenagrionidae_Acanthagrion_trilobatum	5.00740769	5.200566	Percher	927
TMCH16_Coenagrionidae_Protoneura_amatoria	6.09858882	6.14693175	Percher	190
**Cordulegastridae**				
OD1164_Cordulegastridae_Cordulegaster_sp.	3.47294783	4.53740914	Flyer	893
OD1304_Cordulegastridae_Neallogaster_hermionae	3.315789474	4.29787234	Flyer	902
RMNH.INS.508536_Cordulegastridae_Anotogaster_sieboldii	4.37799043	3.53629032	Flyer	916
**Corduliidae**				
GEODE17698_Corduliidae_Dorocordulia_lepida	2.89611024	4.0037906	Flyer	933
GEODE17699_Corduliidae_Dorocordulia_libera	2.89611024	4.0037906	Flyer	930
GEODE17703_Corduliidae_Epitheca_cynosura	2.72884424	3.97652575	Flyer	932
GEODE17704_Corduliidae_Epitheca_marginata	2.72884424	3.97652575	Flyer	925

GEODE17707_Corduliidae_Epitheca_semiaquea	2.72884424	3.97652575	Flyer	929
GEODE17711_Corduliidae_Guadalca_insularis	2.6969697	3.90410959	Flyer	928
GEODE17712_Corduliidae_Helocordulia_selysii	3.191575	4.10196096	Flyer	925
GEODE17713_Corduliidae_Helocordulia_uhleri	3.191575	4.10196096	Flyer	922
GEODE17717_Corduliidae_Hemicordulia_fidelis	3.07543103	4.01891892	Flyer	929
GEODE17720_Corduliidae_Hemicordulia_kalliste	3.07543103	4.01891892	Flyer	929
GEODE17779_Corduliidae_Navicordulia_errans	3.148514851	4.012345679	Flyer	769
GEODE17781_Corduliidae_Neurocordulia_virginiensis	2.75956459	3.68368385	Flyer	874
GEODE17783_Corduliidae_Neurocordulia_yamaskanensis	2.75956459	3.68368385	Flyer	925
GEODE17788_Corduliidae_Procordulia_jacksoniensis	2.96994536	3.86006826	Flyer	907
GEODE17789_Corduliidae_Procordulia_moroensis	2.96994536	3.86006826	Flyer	918
GEODE17793_Corduliidae_Neurocordulia_michaeli	2.75956459	3.68368385	Flyer	920
GEODE17832_Corduliidae_Somatochlora_cingulata	3.10288889	4.09978786	Flyer	923
GEODE7747_Corduliidae_Cordulia_shurtleffii	2.92526281	3.92882638	Flyer	927
GEODE7765_Corduliidae_Paracordulia_sericea	2.81481482	3.83435583	Flyer	932
OD1322_Corduliidae_Rialla_villosa	3.10869565	4.08219178	Flyer	907
OD146_Corduliidae_Cordulia_aenea	2.92526281	3.92882638	Flyer	902
OD83_Corduliidae_Hemicordulia_australiae	3.07543103	4.01891892	Flyer	923
RMNH.5009497_Corduliidae_Corduliochlora_borisi	2.794871795	3.2	Flyer	920
RMNH.5011578_Corduliidae_Metaphya_tillyardi	3.158730159	3.99047619	Flyer	934
RMNH.INS.500518_Corduliidae_Procordulia_leopoldi	2.96994536	3.86006826	Flyer	933
RMNH.INS.506657_Corduliidae_Heteronaias_heterodoxa	2.95081967	4.11827957	Flyer	913
**Devadattidae**				
OD151_Devadattidae_Devadatta_podolestoides	3.99924	1.02879	Percher	920
RMNH.INS.501127_Devadattidae_Devadatta_argyoides	3.99924	1.02879	Percher	934
Dicteriadidae				
GEODE7718_Dicteriadidae_Dicterias_atrosanguinea	7.6797	0.21735	Percher	923
GEODE7788_Dicteriadidae_Heliocharis_amazona	4.58064516	4.88709677	Percher	927

**Epiophlebiidae**				
OD1076_Epiophlebiidae_Epiophlebia_superstes	3.888	4.0078125	Flyer	912
OD1302_Epiophlebiidae_Epiophlebia_laidlawi	3.888	4.0078125	Flyer	907
**Euphaeidae**				
GEODE2756_Euphaeidae_Heterophaea_barbata	5	4.982758621	Percher	872
GEODE7683_Euphaeidae_Anisopleura_lestoides	4.77586207	4.83076923	Percher	915
GEODE7686_Euphaeidae_Euphaea_cardinalis	3.37254902	3.65605096	Percher	872
GEODE7687_Euphaeidae_Cyclophaea_cyanifrons	4.618181818	4.872727273	Percher	918
GEODE7694_Euphaeidae_Euphaea_refulgens	3.37254902	3.65605096	Percher	931
GEODE7858_Euphaeidae_Cryptophaea_saukra	5.81034483	5.625	Percher	908
OD132_Euphaeidae_Euphaea_ochracea	3.37254902	3.65605096	Percher	902
OD99_Euphaeidae_Bayadera_brevicauda	4.62711864	5.74545455	Percher	903
RMNH.5009494_Euphaeidae_Epallage_fatime	3.98684211	3.7631579	Percher	930
RMNH.INS.501089_Euphaeidae_Dysphaea_dimidiata	5.02803	0.81144	Percher	938
RMNH.INS.505757_Euphaeidae_Anisopleura_furcata	4.77586207	4.83076923	Percher	917
RMNH.INS.505817_Euphaeidae_Cryptophaea_vietnamensis	5.75	5.79661017	Percher	926
**Gomphidae**				
GEODE12753_Gomphidae_Lindenia_tetraphylla	2.99082569	4.3373494	Percher	691
GEODE12754_Gomphidae_Mattigomphus_pinratani	3.438596491	4.224489796	Percher	911
GEODE12755_Gomphidae_Perissogomphus_stevensi	3.309090909	4.131868132	Percher	766
GEODE17903_Gomphidae_Anisogomphus_anderi	3.07565012	4.29216868	Percher	919
GEODE17904_Gomphidae_Arigomphus_furcifer	3.26673081	4.18107748	Percher	931
GEODE17909_Gomphidae_Zonophora_diversa	?3.321428571	4.06122449	Percher	870
GEODE17911_Gomphidae_Cacoides_latro	3.66346154	4.3125	Percher	918
GEODE17912_Gomphidae_Hagenius_brevistylus	3.52124933	4.73995552	Percher	926
GEODE17913_Gomphidae_Fukienogomphus_promineus	3.32286996	4.42934783	Percher	917
GEODE17914_Gomphidae_Shaogomphus_postocularis	3	4.220930233	Percher	918
GEODE17915_Gomphidae_Peruviogomphus_pearsoni	3.34666667	4.36507937	Percher	779

GEODE17917_Gomphidae_Octogomphus_specularis	2.97762902	4.07693172	Percher	926
GEODE17919_Gomphidae_Gastrogomphus_abdominalis	3.20833333	3.94047619	Percher	917
GEODE17922_Gomphidae_Melligomphus_ardens	3.21546961	4.02884615	Percher	915
GEODE17923_Gomphidae_Merogomphus_longistigma	3.31468532	4.50638298	Percher	848
GEODE17933_Gomphidae_Scalmogomphus_falcatus	2.97777778	4.11347518	Percher	896
GEODE17935_Gomphidae_Anormogomphus_kiritshenkoi	2.5257732	3.48051948	Percher	462
GEODE17936_Gomphidae_Phanogomphus_exilis	3.04563132	4.00653972	Percher	918
GEODE7634_Gomphidae_Sinogomphus_telamon	2.978723404	4.052631579	Percher	206
GEODE7681_Gomphidae_Phyllogomphoides_undulatus	3.38519257	4.401727	Percher	617
GEODE7692_Gomphidae_Phyllogomphoides_fuliginosus	3.38519257	4.401727	Percher	742
GEODE7710_Gomphidae_Ebegomphus_sp.	3.4	3.655172414	Percher	931
GEODE7731_Gomphidae_Ictinogomphus_pertinax	3.3019802	4.31024096	Percher	923
GEODE7732_Gomphidae_Gomphurus_externus	3.10059141	4.18780785	Percher	932
GEODE7733_Gomphidae_Tragogomphus_ellioti	?	?	Percher	922
GEODE7734_Gomphidae_Heliogomphus_scorpio	3.29787234	4.25628141	Percher	917
GEODE7736_Gomphidae_Erpetogomphus_compositus	3.05787602	4.01137388	Percher	924
GEODE7745_Gomphidae_Armagomphus_armiger	3.10526316	4.25	Percher	932
GEODE7754_Gomphidae_Stenogomphurus_rogersi	3.52461526	4.35295149	Percher	930
GEODE7763_Gomphidae_Archaeogomphus_nanus	2.99539171	4.20108696	Percher	913
GEODE7772_Gomphidae_Ebegomphus_minutus	3.4	3.655172414	Percher	928
GEODE7773_Gomphidae_Zonophora_batesi	3.321428571	4.06122449	Percher	925
GEODE7774_Gomphidae_Desmogomphus_tigrivensis	3.01948052	4.216	Percher	927
GEODE7775_Gomphidae_Agriogomphus_sylvicola	2.99516908	3.99418605	Percher	919
GEODE7790_Gomphidae_Antipodogomphus_acolythus	3.0131579	4.08064516	Percher	938
GEODE7818_Gomphidae_Aphylla_sp.	3.41009693	4.34958561	Percher	920
OD1314_Gomphidae_Ophiogomphus_morrisoni	2.98475264	4.02416394	Percher	904

OD69_Gomphidae_Ictinogomphus_australis	3.3019802	4.31024096	Percher	601
OD85_Gomphidae_Austrogomphus_amphiclitus	3.06397306	4.08943089	Percher	772
RMNH.5008090_Gomphidae_Ceratogomphus_pictus	3.32170543	4.22685185	Percher	928
RMNH.5009376_Gomphidae_Onychogomphus_styx	3.05576208	4.17933492	Percher	924
RMNH.5011399_Gomphidae_Stylurus_flavipes	3.43879674	4.3333835	Percher	922
RMNH.5011415_Gomphidae_Austroepigomphus_praeruptus	2.96666667	3.95967742	Percher	933
RMNH.5011429_Gomphidae_Hemigomphus_comitatus	3.09698997	4.28333333	Percher	927
RMNH.5011478_Gomphidae_Austrogomphus_prasinus	3.223684211	4.174603175	Percher	923
RMNH.INS.229080_Gomphidae_Macrogomphus_quadratus	3.33628319	4.2924282	Percher	900
RMNH.INS.501184_Gomphidae_Phaenandrogomphus_asthenes	3.20289855	3.98412698	Percher	911
RMNH.INS.501444_Gomphidae_Libyogomphus_christinae	3.285714286	3.6	Percher	866
RMNH.INS.501518_Gomphidae_Microgomphus_camerunensis	3.12244898	4.46956522	Percher	907
RMNH.INS.502394_Gomphidae_Lestinogomphus_congoensis	3.07079646	3.67619048	Percher	931
RMNH.INS.502555_Gomphidae_Gomphidia_gamblesi	3.28697851	4.3250774	Percher	912
RMNH.INS.502891_Gomphidae_Nepogomphoides_stuhlmanni	2.77777778	3.57377049	Percher	912
RMNH.INS.503108_Gomphidae_Diastatomma_gamblesi	2.95575221	3.98333333	Percher	871
RMNH.INS.503523_Gomphidae_Sieboldius_japponicus	3.802325581	4.694444444	Percher	927
RMNH.INS.503524_Gomphidae_Acrogomphus_jubilaris	2.98019802	4.282051282	Percher	917
RMNH.INS.504961_Gomphidae_Austrogomphus_ochraceus	3.06397306	4.08943089	Percher	931
RMNH.INS.505397_Gomphidae_Neurogomphus_cocytius	3.33333333	4.71621622	Percher	922
RMNH.INS.505653_Gomphidae_Notogomphus_praetorius	3.23876405	4.3183391	Percher	937

RMNH.INS.505754_Gomphidae_Amphigomphus_somnuki	3.21052632	4.23566879	Percher	924
RMNH.INS.506264_Gomphidae_Leptogomphus_coomansi	3.15189873	4.38977636	Percher	926
RMNH.INS.506367_Gomphidae_Megalogomphus_borneensis	3.3628692	4.46842105	Percher	764
RMNH.INS.507519_Gomphidae_Ophiogomphus_cecilia	2.98475264	4.02416394	Percher	916
RMNH.INS.507929_Gomphidae_Phyllogomphus_annulus	3.081632653	4.051282051	Percher	924
RMNH.INS.508012_Gomphidae_Paragomphus_genei	3.25177305	3.62641509	Percher	909
RMNH.INS.508545_Gomphidae_Trigomphus_beatus	3.168674699	4.507462687	Percher	910
RMNH.INS.508624_Gomphidae_Fukienogomphus_promineus	3.32286996	4.42934783	Percher	925
RMNH.INS.508661_Gomphidae_Lamelligomphus_camelus	3.30625	4.08363636	Percher	913
RMNH.INS.508662_Gomphidae_Melligomphus_sinicus	3.30625	4.08363636	Percher	914
RMNH.INS.508663_Gomphidae_Nihonogomphus_semanticus	3.06145251	4.06122449	Percher	923
RMNH.INS.508672_Gomphidae_Nepogomphus_walli	2.83823529	4.05769231	Percher	902
RMNH.INS.508691_Gomphidae_Asiagomphus_hainanensis	3.45812808	4.03252033	Percher	922
RMNH.INS.508700_Gomphidae_Labrogomphus_torvus	3.36296296	4.41284404	Percher	926
RMNH.INS.509134_Gomphidae_Euthygomphus_koxingai	3.026548673	4.042553191	Percher	920
RMNH.INS.509144_Gomphidae_Dubitogomphus_bicornutus	2.64444444	3.9916318	Percher	928
RMNH.INS.509146_Gomphidae_Sinictinogomphus_clavatus	3.524752475	4.728395062	Percher	913
RMNH.INS.509207_Gomphidae_Burmagomphus_divaricatus	2.83850932	4.02419355	Percher	409
RMNH.INS.509920_Gomphidae_Lamelligomphus_castor	3.30625	4.08363636	Percher	920
RMNH.INS.509971_Gomphidae_Orientogomphus_minor	2.9625	4.81818182	Percher	923

RMNH.INS.554426_Gomphidae_Mastigogomphus_chapini	?	?	Percher	909
RMNH.INS.554510_Gomphidae_Cornigomphus_guineensis	3.297297297	4.194444444	Percher	896
RMNH.INS.559802_Gomphidae_Crenigomphus_hartmanni	3.01520913	4.03255814	Percher	908
GEODE17894_Gomphomacromiidae_Gomphomacromia_nodisticta	2.91988131	3.86891386	Flyer	669
OD1169_Gomphomacromiidae_Gomphomacromia_paradoxa	2.91988131	3.86891386	Flyer	907
**Hemiphlebiidae**				
OD1074_Hemiphlebiidae_Hemiphlebia_mirabilis	1.43451	0.34776	Percher	877
**Heteragrionidae**				
GEODE7742_Heteragrionidae_Oxystigma_petiolatum	6.24324324	5.24444444	Percher	927
OD1126_Heteragrionidae_Dimeragrion_percubitale	5.150943396	6.08	Percher	927
TM155_Heteragrionidae_Heteragrion_tatama	5.60174836	5.46935167	Percher	914
TM404_Heteragrionidae_Heteragrion_erythrogastrum	5.60174836	5.46935167	Percher	906
**Hypolestidae**				
OD1146_Hypolestidae_Hypolestes_sp	6.525	4.96363636	Percher	923
**Idionychidae**				
GEODE17864_Idionychidae_Idionyx_claudia	2.8	3.90429043	Flyer	927
GEODE17870_Idionychidae_Idionyx_optatus	2.8	3.90429043	Flyer	895
RMNH.INS.506617_Idionychidae_Idionyx_philippa	2.8	3.90429043	Flyer	918
**Idomacromiidae**				
GEODE17881_Idomacromiidae_Neocordulia_griphus	2.855421687	3.833333333	Flyer	895
NESOMASC_Idomacromiidae_Nesocordulia_mascarenica	2.540816327	3	Flyer	687
RMNH.5008153_Idomacromiidae_Syncordulia_gracilis	2.90909091	3.8	Flyer	927
RMNH.INS.503184_Idomacromiidae_Idomacromia_proavita	2.93700787	3.70476191	Flyer	927
RMNH.INS.507576_Idomacromiidae_Oxygastra_curtisii	2.8995098	3.89776358	Percher	912
RU139_Idomacromiidae_Neocordulia_volxemi	2.855421687	3.833333333	Flyer	278
TM41_Idomacromiidae_Neocordulia_batesi	2.855421687	3.833333333	Flyer	916

**Isostictidae**				
GEODE7860_Isostictidae_Austrosticta_soror	7.55	6	Percher	829
OD1277_Isostictidae_Neosticta_fraseri	5.68115942	5.26666667	Percher	920
OD1298_Isostictidae_Cnemisticta_latilobata	5.84782609	5.85416667	Percher	924
OD1312_Isostictidae_Labidiosticta_vallisi	4.89361702	5.61702128	Percher	917
OD159_Isostictidae_Isosticta_robustior	5.825	5.75609756	Percher	924
OD55_Isostictidae_Rhadinosticta_simplex	5.21621622	5.28571429	Percher	913
RMNH.5011475_Isostictidae_Oristicta_filicicola	5.64102564	5.67901235	Percher	925
RMNH.5011582_Isostictidae_Tanymecosticta_leptalea	7.06666667	6.87096774	Percher	916
RMNH.INS.229243_Isostictidae_Selysioneura_capreola	7.61764706	7.25714286	Percher	918
**Lauromacromiidae**				
GEODE7771_Lauromacromiidae_Lauromacromia_dubitalis	3.166666667	3.829787234	Flyer	934
RU123_Lauromacromiidae_Lauromacromia_bedei	3.166666667	3.829787234	Flyer	608
RU126_Lauromacromiidae_Lauromacromia_picinguaba	3.166666667	3.829787234	Flyer	808
**Lestidae**				
GEODE7665_Lestidae_Indolestes_albicaudus	5.13513514	5.15789474	Percher	786
GEODE7728_Lestidae_Austrolestes_leda	4.11111111	4.525	Percher	906
GEODE7730_Lestidae_Archilestes_grandis	4.97674211	5.00195116	Percher	920
GEODE7813_Lestidae_Lestes_plagiatus	4.6041491	4.60267429	Percher	909
OD1127_Lestidae_Orolestes_octomaculatus	4.257575758	5.107692308	Percher	912
OD122_Lestidae_Sympecma_paedisca	4.85	4.6	Percher	888
OD1278_Lestidae_Indolestes_cyaneus	5.13513514	5.15789474	Percher	908
OD1321_Lestidae_Lestes_disjunctus	4.6041491	4.60267429	Percher	907
OD63_Lestidae_Austrolestes_annulosus	4.11111111	4.525	Percher	851
RMNH.INS.507448_Lestidae_Chalcolestes_viridis	4.365853659	4.585365854	Percher	912
**Lestoideidae**				
OD157_Lestoideidae_Lestoidea_conjuncta	5.780487805	5.697674419	Percher	922
OD43_Lestoideidae_Diphlebia_coerulescens	5.215384615	4.735294118	Percher	906
RMNH.5011424_Lestoideidae_Diphlebia_euphoeoides	5.215384615	4.735294118	Percher	933
**Libellulidae**				
GEODE12746_Libellulidae_Akrothemis_risi	3.025862069	4.461538462	Percher	387

GEODE12747_Libellulidae_Amphithemis_curvistyla	2.608695652	3.222222222	Percher	905
GEODE12748_Libellulidae_Celebophlebia_dactylogastra	3.24	4.195121951	Percher	869
GEODE12749_Libellulidae_Celebothemis_delecollei	3.37593985	4.55	Percher	896
GEODE17725_Libellulidae_Orthemis_macrostigma	3.18752405	4.59889563	Percher	931
GEODE17728_Libellulidae_Orthemis_sulphurata	3.18752405	4.59889563	Percher	924
GEODE17755_Libellulidae_Anatya_januaria	3.569620253	4.362318841	Percher	882
GEODE17756_Libellulidae_Antidythemis_trameiformis	3.54237288	4.55445545	Percher	236
GEODE17757_Libellulidae_Archaeophlebia_martini	2.72916667	3.81333333	Percher	651
GEODE17758_Libellulidae_Atoconeura_kenya	2.97487437	4.27083333	Percher	392
GEODE17761_Libellulidae_Boninthemis_insularis	3.08433735	4.63492064	Percher	223
GEODE17929_Libellulidae_Scapanea_frontalis	3.163793103	4.111111111	Percher	808
GEODE7612_Libellulidae_Cannaphila_insularis	3.47955016	4.75537578	Percher	182
GEODE7628_Libellulidae_Rhodopygia_hinei	3.31146482	4.53146285	Percher	250
GEODE7638_Libellulidae_Nannophlebia_axiagasta	3.0562249	4.03773585	Percher	626
GEODE7650_Libellulidae_Diplacina_spec	3.10746269	4.19011407	Percher	851
GEODE7655_Libellulidae_Diplacina_persephone	3.10746269	4.19011407	Percher	470
GEODE7659_Libellulidae_Microtrigonia_gomphoides	2.92391304	3.98648649	Percher	812
GEODE7676_Libellulidae_Misagria_calverti	3.69887374	4.92970163	Percher	899
GEODE7698_Libellulidae_Erythemis_credula	3.13688237	4.19493393	Percher	930
GEODE7699_Libellulidae_Erythrodiplax_umbrata	2.96539276	3.98291632	Percher	932
GEODE7700_Libellulidae_Oligoclada_sp	2.7792045	4.05936065	Percher	928
GEODE7701_Libellulidae_Agrionoptera_insignis	3.77620397	4.7359736	Percher	927
GEODE7702_Libellulidae_Uracis_fastigiata	2.75723368	3.72006014	Percher	928
GEODE7707_Libellulidae_Elga_sp	3.07471623	3.9556441	Percher	930
GEODE7708_Libellulidae_Plathemis_subornata	2.79724774	3.76418041	Percher	933
GEODE7709_Libellulidae_Rhyothemis_notata	3.04658436	4.02277096	Percher	930
GEODE7711_Libellulidae_Celithemis_eponina	2.60681332	3.79407154	Percher	929
GEODE7712_Libellulidae_Perithemis_sp	2.54409106	3.34655979	Percher	924
GEODE7714_Libellulidae_Brachythemis_leucosticta	2.95053989	3.89423445	Percher	930

GEODE7735_Libellulidae_Ladona_julia	2.9808918	4.02311388	Percher	931
GEODE7738_Libellulidae_Brechmorhoga_mendax	3.01418332	4.27825719	Percher	932
GEODE7739_Libellulidae_Perithemis_tenera	2.54409106	3.34655979	Percher	920
GEODE7746_Libellulidae_Paltothemis_lineatipes	2.66335907	4.30100673	Percher	932
GEODE7756_Libellulidae_Pachydiplax_longipennis	2.94262678	4.06280557	Percher	933
GEODE7759_Libellulidae_Nannophya_australis	2.34615385	3.42574257	Percher	930
GEODE7760_Libellulidae_Austrothemis_nigrescens	3.314655172	4.451428571	Percher	932
GEODE7761_Libellulidae_Tauriphila_argo	2.7707949	4.14907466	Flyer	927
GEODE7762_Libellulidae_Macrothemis_nobilis	2.928154	4.14129019	Percher	930
GEODE7764_Libellulidae_Miathyria_marcella	2.58674413	4.25459364	Flyer	930
GEODE7766_Libellulidae_Zenithoptera_fasciata	2.66324126	3.17657591	Percher	933
GEODE7767_Libellulidae_Nephepeltia_phryne	3.21571095	4.08368419	Percher	926
GEODE7768_Libellulidae_Elasmothemis_williamsoni	3.90510519	4.58482123	Percher	932
GEODE7769_Libellulidae_Argyrothemis_argentea	3.485714286	3.96875	Percher	926
GEODE7770_Libellulidae_Gynothemis_pumilia	2.896825397	3.514851485	Percher	926
GEODE7776_Libellulidae_Micrathyria_didyma	3.03889894	4.23231737	Percher	933
GEODE7777_Libellulidae_Dythemis_nigra	2.98945067	4.29395073	Percher	934
GEODE7798_Libellulidae_Palpopleura_lucia	2.93181818	3.4717608	Percher	908
GEODE7800_Libellulidae_Diplacodes_trivialis	2.96063261	3.98633147	Percher	928
GEODE7801_Libellulidae_Crocothemis_servilia	3.10775333	4.27019123	Percher	926
GEODE7802_Libellulidae_Aethriamanta_rezia	2.81132076	4.14193548	Percher	924
GEODE7803_Libellulidae_Diastatops_obscura	2.31090937	3.07790585	Percher	930
GEODE7804_Libellulidae_Tetrathemis_fraseri	2.87027027	3.53012048	Percher	934
GEODE7805_Libellulidae_Acisoma_trifidum	2.92199522	3.64458013	Percher	920
GEODE7806_Libellulidae_Orthetrum_julia	3.930232558	4.581081081	Percher	926
GEODE7807_Libellulidae_Tramea_transmarina	2.42767702	4.20920122	Flyer	923
GEODE7815_Libellulidae_Urothemis_assignata	3.37837838	4.2875817	Percher	864
OD107_Libellulidae_Pantala_flavescens	2.5287276	4.17827552	Flyer	872
OD1091_Libellulidae_Brachythemis_contaminata	2.95053989	3.89423445	Percher	783
OD1094_Libellulidae_Nannophlebia_alexia	3.0562249	4.03773585	Percher	743
OD1095_Libellulidae_Sympetrum_corruptum	2.63194789	3.74345689	Percher	916
OD1106_Libellulidae_Zygonyx_flavicosta	3.22428571	4.23948812	Percher	839

OD1319_Libellulidae_Macrodiplax_cora	2.56924349	4.07435084	Percher	920
OD77_Libellulidae_Rhyothemis_graphiptera	3.04658436	4.02277096	Percher	843
OD82_Libellulidae_Brachydiplax_denticauda	3.02	4.23684211	Percher	925
RMNH.5007803_Libellulidae_Atoconeura_biordinata	2.97487437	4.27083333	Percher	936
RMNH.5008306_Libellulidae_Neurothemis_fluctuans	2.67073171	3.5664557	Percher	927
RMNH.5011411_Libellulidae_Notolibellula_bicolor	3.139534884	3.641025641	Percher	926
RMNH.5011412_Libellulidae_Microtrigonia_curvata	2.92391304	3.98648649	Percher	930
RMNH.5011413_Libellulidae_Bironides_glochidion	3.06993007	4.15254237	Percher	931
RMNH.INS.228976_Libellulidae_Tetrathemis_irregularis	2.87027027	3.53012048	Percher	926
RMNH.INS.228999_Libellulidae_Raphismia_bispina	3.333333333	4.357142857	Percher	928
RMNH.INS.500592_Libellulidae_Huonia_epinephela	3.11163896	4.14328358	Percher	924
RMNH.INS.500644_Libellulidae_Protorthemis_coronata	3.74747475	4.30434783	Percher	935
RMNH.INS.501123_Libellulidae_Acisoma_panorpoides	2.92199522	3.64458013	Percher	931
RMNH.INS.501152_Libellulidae_Pseudothemis_jorina	3.62430939	3.60655738	Percher	925
RMNH.INS.501189_Libellulidae_Aethriamanta_brevipennis	2.81132076	4.14193548	Percher	932
RMNH.INS.501196_Libellulidae_Rhodothemis_rufa	3.35555556	4.25675676	Percher	932
RMNH.INS.501275_Libellulidae_Orchithemis_xanthosoma	3.30232558	4.50943396	Percher	932
RMNH.INS.501284_Libellulidae_Brachygonia_oculata	3.43636364	4.40425532	Percher	923
RMNH.INS.501296_Libellulidae_Tholymis_tillarga	2.69152229	4.04713805	Flyer	926
RMNH.INS.501373_Libellulidae_Chalybeothemis_fluviatilis	3.0483871	4.08163265	Percher	926
RMNH.INS.501377_Libellulidae_Zyxomma_petiolatum	2.95372751	3.80392157	Percher	930
RMNH.INS.501398_Libellulidae_Pornothemis_sp	3.416666667	4.618421053	Percher	929
RMNH.INS.501662_Libellulidae_Eleuthemis_buettikoferi	2.526315789	3.970149254	Percher	917
RMNH.INS.502121_Libellulidae_Oxythemis_phoenicosceles	2.92380952	4.03781513	Percher	924

RMNH.INS.502150_Libellulidae_Chalcostephia_flavifrons	3.31081081	4.4	Percher	925
RMNH.INS.502177_Libellulidae_Notiothemis_robertsi	3.13846154	4.11320755	Percher	923
RMNH.INS.502225_Libellulidae_Aethiothemis_erythromelas	2.96978852	3.92481203	Percher	932
RMNH.INS.502285_Libellulidae_Cyanothemis_simpsoni	3.06617647	4.11607143	Percher	925
RMNH.INS.502287_Libellulidae_Micromacromia_camerunica	3	3.96682465	Percher	926
RMNH.INS.502304_Libellulidae_Parazyxomma_flavicans	2.583333333	3.137931034	Percher	934
RMNH.INS.502305_Libellulidae_Hadrothemis_camarensis	3.43106796	4.33180778	Percher	929
RMNH.INS.502397_Libellulidae_Malgassophlebia_bispina	2.91358025	3.95454546	Percher	922
RMNH.INS.502400_Libellulidae_Nesciothemis_farinosa	3.05183585	3.88802083	Percher	929
RMNH.INS.502847_Libellulidae_Thermochoria_jeanneli	3.15625	4.078740157	Percher	924
RMNH.INS.503416_Libellulidae_Lyriothemis_cleis	3.04761905	4.39184953	Percher	931
RMNH.INS.503419_Libellulidae_Nesoxenia_lineata	3.61437909	4.6171875	Percher	929
RMNH.INS.503939_Libellulidae_Hydrobasileus_croceus	3.224	3.96091205	Percher	932
RMNH.INS.503943_Libellulidae_Indothemis_limbata	3.15819209	4.23188406	Percher	913
RMNH.INS.503957_Libellulidae_Potamarcha_congener	2.915662651	3.808823529	Percher	924
RMNH.INS.503963_Libellulidae_Lathrecista_asiatica	3.809090909	4.547368421	Percher	917
RMNH.INS.503964_Libellulidae_Urothemis_signata	3.37837838	4.2875817	Percher	918
RMNH.INS.504032_Libellulidae_Onychothemis_culminicola	3.08539945	4.27075812	Percher	916
RMNH.INS.504039_Libellulidae_Orchithemis_pulcherrima	3.30232558	4.50943396	Percher	924
RMNH.INS.504459_Libellulidae_Hemistigma_albipunctum	3.03846154	3.77272727	Percher	936
RMNH.INS.504999_Libellulidae_Orthetrum_cancellatum	3.930232558	4.581081081	Percher	927

RMNH.INS.505354_Libellulidae_Trithetrum_navasi	?	?	Percher	926
RMNH.INS.505362_Libellulidae_Crocothemis_erythraea	3.10775333	4.27019123	Percher	933
RMNH.INS.505380_Libellulidae_Olpogastra_lugubris	3.36111111	4.36046512	Percher	932
RMNH.INS.505547_Libellulidae_Porpax_risi	3.34228188	4.05555556	Percher	926
RMNH.INS.505588_Libellulidae_Diplacodes_lefebvrii	2.96063261	3.98633147	Percher	927
RMNH.INS.505652_Libellulidae_Bradinopyga_cornuta	3.27891157	4.30567686	Percher	930
RMNH.INS.505880_Libellulidae_Tapeinothemis_boharti	3.606741573	4.986111111	Percher	936
RMNH.INS.506254_Libellulidae_Camacinia_gigantea	3.01020408	4.17525773	Percher	802
RMNH.INS.506290_Libellulidae_Cratilla_lineata	3.80579784	4.78231228	Percher	920
RMNH.INS.507392_Libellulidae_Leucorrhinia_rubicunda	2.9122135	3.81909169	Percher	934
RMNH.INS.507400_Libellulidae_Libellula_depressa	3.14477186	4.3500372	Percher	930
RMNH.INS.507439_Libellulidae_Libellula_quadrimaculata	3.14477186	4.3500372	Percher	931
RMNH.INS.507465_Libellulidae_Sympetrum_danae	2.63194789	3.74345689	Percher	929
RMNH.INS.507492_Libellulidae_Nannodiplax_rubra	2.755555556	3.5	Percher	921
RMNH.INS.507529_Libellulidae_Trithemis_annulata	2.76976744	4.14527027	Percher	921
RMNH.INS.507551_Libellulidae_Selysiothemis_nigra	2.65591398	3.44444444	Percher	929
RMNH.INS.507746_Libellulidae_Hylaeothemis_clementia	3.11026616	4.15492958	Percher	932
RMNH.INS.507831_Libellulidae_Tyriobapta_torrida	2.74324324	3.92982456	Percher	932
RMNH.INS.508001_Libellulidae_Zygonyx_torridus	3.22428571	4.23948812	Percher	926
RMNH.INS.508447_Libellulidae_Palpopleura_sexmaculata	2.93181818	3.4717608	Percher	921
RMNH.INS.508515_Libellulidae_Nannophyopsis_clara	2.03030303	3.35294118	Percher	927
RMNH.INS.508797_Libellulidae_Neodythemis_afra	3.24200913	4.24719101	Percher	926

RMNH.INS.509795_Libellulidae_Thalassothemis_marchali	3.39047619	4.641025641	Percher	926
RMNH.INS.559789_Libellulidae_Zygonoides_fuelleborni	3.206349206	4.168421053	Percher	927
TM207_Libellulidae_Brechmorhoga_vivax	3.01418332	4.27825719	Percher	925
TM332_Libellulidae_Cannaphila_vibex	3.47955016	4.75537578	Percher	912
TM342_Libellulidae_Macrothemis_pseudimitans	2.928154	4.14129019	Percher	921
TM409_Libellulidae_Rhodopygia_hinei	3.31146482	4.53146285	Percher	918
TM532_Libellulidae_Erythemis_plebeja	3.13688237	4.19493393	Percher	910
**Macromidiidae**				
GEODE17874_Macromidiidae_Macromidia_genialis	3.01522843	4.21052632	Flyer	930
GEODE17876_Macromidiidae_Macromidia_kelloggi	3.01522843	4.21052632	Flyer	930
RMNH.INS.506247_Macromidiidae_Macromidia_fulva	3.01522843	4.21052632	Flyer	930
**Macromiidae**				
GEODE17622_Macromiidae_Macromia_astarte	3.41770018	4.41804763	Flyer	903
GEODE7715_Macromiidae_Phyllomacromia_picta	3.38243626	4.26086957	Flyer	927
OD1101_Macromiidae_Epophthalmia_elegans	3.58130081	4.387471	Flyer	903
RMNH.INS.507575_Macromiidae_Macromia_splendens	3.41770018	4.41804763	Flyer	843
**Megapodagrionidae**				
GEODE7863_Megapodagrionidae_Allopodagrion_contortum	3.42307692	4.55769231	Percher	845
OD1281_Megapodagrionidae_Teinopodagrion_meridionale	4.575	4.02272727	Percher	918
**Mesagrionidae**				
OD1183_Mesagrionidae_Mesagrion_leucorrhinum	4.5945946	6.16666667	Percher	927
**Mesopodagrionidae**				
OD1077_Mesopodagrionidae_Mesopodagrion_sp	3.39705882	4.35714286	Percher	914
**Neopetaliidae**				
OD1113_Neopetaliidae_Neopetalia_punctata	3.578947368	3.865384615	Flyer	931
**Neophyidae**				
RMNH.INS.502324_Neophyidae_Neophya_rutherfordi	2.99137931	3.453608247	Flyer	903
**Pentaphlebiidae**				

OD1139_Pentaphlebiidae_Pentaphlebia_mangana	4.276923077	5.111111111	Percher	909
RMNH.INS.500093_Pentaphlebiidae_Pentaphlebia_stahli	3.00760456	3.87614679	Percher	927
**Perilestidae**				
OD1120_Perilestidae_Perissolestes_remotus	5.2	5.142857143	Percher	903
**Petaluridae**				
GEODE17940_Petaluridae_Petalura_litorea	3.513513514	4.620689655	Percher	890
GEODE17943_Petaluridae_Uropetala_chiltoni	3.67460318	4.50691244	Percher	840
GEODE7743_Petaluridae_Tanypteryx_hageni	2.96820319	4.11517538	Percher	925
OD1318_Petaluridae_Petalura_gigantea	3.513513514	4.620689655	Percher	894
PD166_Petaluridae_Tanypteryx_pryeri	2.96820319	4.11517538	Percher	825
**Philogangidae**				
OD1079_Philogangidae_Philoganga_vetusta	4.91358025	6.15789474	Percher	927
**Philgeniidae**				
OD1124_Philogeniidae_Philogenia_carrillica	4.03961039	4.721171802	Percher	909
TM201_Philogeniidae_Philogenia_martae	4.03961039	4.721171802	Percher	916
**Philosinidae**				
OD1119_Philosinidae_Rhinagrion_sp	5.083333333	5.03125	Percher	928
OD1130_Philosinidae_Philosina_buchi	4.262135922	4.712871287	Percher	924
RMNH.INS.506835_Philosinidae_Rhinagrion_macrocephalum	5.083333333	5.03125	Percher	937
**Platycnemididae**				
GEODE2652_Platycnemididae_Asthenocnemis_stephanodera	4.03030303	4.48795181	Percher	682
GEODE7624_Platycnemididae_Disparoneura_apicalis	3.12984	0.62307	Percher	253
GEODE7653_Platycnemididae_Esme_mudiensis	6.42857143	5.11111111	Percher	740
GEODE7656_Platycnemididae_Nososticta_selysii	5.575757576	4.571428571	Percher	525
GEODE7660_Platycnemididae_Nososticta_exul	5.575757576	4.571428571	Percher	469
GEODE7668_Platycnemididae_Calicnemia_pulverulans	4.1682243	4.7184466	Percher	892
GEODE7673_Platycnemididae_Calicnemia_erythromelas	4.1682243	4.7184466	Percher	926
GEODE7724_Platycnemididae_Coeliccia_cyanomelas	5.24705882	5.51162791	Percher	927
GEODE7727_Platycnemididae_Prodasineura_autumnalis	5.54074074	5.78832117	Percher	923

GEODE7750_Platycnemididae_Copera_nyansana	4.06	4.25	Percher	924
GEODE7796_Platycnemididae_Elattoneura_acuta	2.52126	0.49266	Percher	914
GEODE7811_Platycnemididae_Copera_marginipes	4.06	4.25	Percher	916
GEODE7864_Platycnemididae_Hylaeargia_magnifica	4.72	5.163265306	Percher	880
GEODE7866_Platycnemididae_Lieftinckia_malaitae	4.221052632	4.935483871	Percher	887
GEODE7868_Platycnemididae_Rhyacocnemis_prothoracica	4.75609756	4.22222222	Percher	922
OD1122_Platycnemididae_Prodasineura_croconota	5.54074074	5.78832117	Percher	912
OD1123_Platycnemididae_Coeliccia_pyriformis	5.24705882	5.51162791	Percher	915
OD1132_Platycnemididae_Onychargia_atrocyana	3.34883721	3.63333333	Percher	917
OD1143_Platycnemididae_Mesocnemis_singularis	4.476190476	4.731707317	Percher	926
OD1306_Platycnemididae_Elattoneura_glauca	2.52126	0.49266	Percher	913
OD1317_Platycnemididae_Nososticta_solida	5.575757576	4.571428571	Percher	924
OD148_Platycnemididae_Platycnemis_pennipes	4.74045802	4.89230769	Percher	905
RMNH.5007870_Platycnemididae_Metacnemis_valida	?	4.851851852	Percher	907
RMNH.5011406_Platycnemididae_Macrocnemis_gracilis	4.120689655	4.767857143	Percher	925
RMNH.INS.229282_Platycnemididae_Paracnemis_alluaudi	4.218181818	4.592592593	Percher	929
RMNH.INS.229291_Platycnemididae_Torrenticnemis_filicornis	6.20930233	5.15384615	Percher	934
RMNH.INS.500162_Platycnemididae_Allocnemis_contraria	4.09803922	5	Percher	917
RMNH.INS.500513_Platycnemididae_Lochmaeocnemis_malacodora	6.525	5.02	Percher	923
RMNH.INS.500580_Platycnemididae_Palaiargia_charmosyna	4.125	4.37777778	Percher	919
RMNH.INS.500639_Platycnemididae_Idiocnemis_obliterata	4.92	5.040816327	Percher	918
RMNH.INS.501977_Platycnemididae_Arrhenocnemis_parvibullis	?	5.535714286	Percher	908
RMNH.INS.501995_Platycnemididae_Cyanocnemis_aureofrons	4.081632653	4.595744681	Percher	914
RMNH.INS.503929_Platycnemididae_Calicnemia_chaseni	4.1682243	4.7184466	Percher	884

RMNH.INS.503944_Platycnemididae_Pseudocopera_ciliata	4.93023256	3.30434783	Percher	760
RMNH.INS.505846_Platycnemididae_Arabicnemis_caerulea	?	4.381818182	Percher	865
RMNH.INS.506232_Platycnemididae_Matticnemis_doi	5.681818182	5.489795918	Percher	889
RMNH.INS.508201_Platycnemididae_Arabineura_khalidi	?	?	Percher	919
RMNH.INS.508637_Platycnemididae_Indocnemis_orang	4.07079646	4.626168224	Percher	928
RMNH.INS.508794_Platycnemididae_Stenocnemis_pachystigma	3.941176471	4.56	Percher	907
RMNH.INS.559644_Platycnemididae_Spesbona_angusta	4.804347826	5.363636364	Percher	908
**Platystictidae**				
GEODE7722_Platystictidae_Protosticta_satoi	5.7	5.71052632	Percher	909
GEODE7787_Platystictidae_Palaemnema_brevignoni	4.83728743	3.07290622	Percher	914
GEODE7871_Platystictidae_Sinosticta_sylvatica	5.5	4.94230769	Percher	899
GEODE7876_Platystictidae_Ceylonosticta_lankanensis	4.790697674	5.023255814	Percher	912
RMNH.5008397_Platystictidae_Telosticta_ulubaram	5.542857143	5.764705882	Percher	890
RMNH.INS.506261_Platystictidae_Telosticta_bidayuh	5.542857143	5.764705882	Percher	886
RMNH.INS.506492_Platystictidae_Sinosticta_ogatai	5.5	4.94230769	Percher	916
RMNH.INS.507659_Platystictidae_Drepanosticta_actaeon	3.72393	0.68103	Percher	911
**Polythoridae**				
ANCH188_Polythoridae_Miocora_peraltica	3.431034483	3.929824561	Percher	920
BOY03_Polythoridae_Cora_xanthostoma	4.189655172	2.983333333	Percher	919
PP330_Polythoridae_Polythore_procera	3.417556	3.48697946	Percher	929
RMNH.INS.505717_Polythoridae_Chalcopteryx_rutilans	3	3.12765957	Percher	920
TM154_Polythoridae_Polythore_gigantea	3.417556	3.48697946	Percher	916
UAE1_Polythoridae_Euthore_fassli	2.99409503	3.32047069	Percher	921
**Priscagrionidae**				

GEODE7872_Priscagrionidae_Sinocnemis_henanensis	5.32142857	5.12962963	Percher	919
OD1280_Priscagrionidae_Priscagrion_kiautai	6.044776119	5.56	Percher	913
**Protolestidae**				
OD1136_Protolestidae_Protolestes_fickei	4.547169811	4.959183673	Percher	923
OD1308_Protolestidae_Protolestes_kerckhoffae	4.547169811	4.959183673	Percher	922
**Pseudocorduliidae**				
GEODE17883_Pseudocorduliidae_Pseudocordulia_circularis	2.94387755	3.875	Flyer	882
GEODE17884_Pseudocorduliidae_Pseudocordulia_elliptica	2.94387755	3.875	Flyer	920
**Pseudolestidae**				
OD1283_Pseudolestidae_Pseudolestes_mirabilis	4.86538462	3.37931035	Percher	924
**Rhipidolestidae**				
OD1085_Rhipidolestidae_Agriomorpha_xinglongensis	4.127272727	4.482142857	Percher	916
OD1121_Rhipidolestidae_Agriomorpha_fusca	4.127272727	4.482142857	Percher	920
OD96_Rhipidolestidae_Rhipidolestes_aculeatus	3.98184739	4.32721569	Percher	908
RMNH.INS.502064_Rhipidolestidae_Rhipidolestes_truncatidens	3.98184739	4.32721569	Percher	929
**Rimanellidae**				
GEODE7704_Rimanellidae_Rimanella_arcana	5.56410256	5.18258258	Percher	926
**Synlestidae**				
GEODE7723_Synlestidae_Chlorolestes_elegans	4.295081967	5.35483871	Percher	926
GEODE7755_Synlestidae_Episynlestes_albicauda	4.4	4.62962963	Percher	921
GEODE7873_Synlestidae_Phylolestes_ethelae	5.41071429	5.27586207	Percher	350
OD1305_Synlestidae_Chlorolestes_umbratus	4.295081967	5.35483871	Percher	913
OD81_Synlestidae_Synlestes_weyersii	5.11627907	5.3125	Percher	919
RMNH.5007835_Synlestidae_Ecchlorolestes_nylephtha	3.88332	0.78246	Percher	911
RMNH.5011485_Synlestidae_Chorismagrion_risi	4.47826087	4.63829787	Percher	912
RMNH.INS.508632_Synlestidae_Megalestes_heros	4.8030303	5.08695652	Percher	919
RMNH.INS.508746_Synlestidae_Nubiolestes_diotima	5.68888889	5.63829787	Percher	917
**Synthemistidae**				

GEODE17885_Synthemistidae_Archaeosynthemis_leachii	3.0560166	3.84343434	Flyer	773
GEODE17887_Synthemistidae_Archaeosynthemis_orientalis	3.0560166	3.84343434	Flyer	895
GEODE17888_Synthemistidae_Archaeosynthemis_spiniger	3.0560166	3.84343434	Flyer	875
GEODE17889_Synthemistidae_Austrosynthemis_cyanitincta	3.09265176	3.89189189	Flyer	834
GEODE17891_Synthemistidae_Eusynthemis_guttata	3.11174242	4.05437352	Flyer	918
GEODE17896_Synthemistidae_Calesynthemis_campioni	2.98701299	3.92434211	Flyer	926
GEODE17901_Synthemistidae_Synthemis_tasmanica	2.98701299	3.92434211	Flyer	872
GEODE17902_Synthemistidae_Tonyosynthemis_ofarrelli	2.88852459	3.85294118	Flyer	354
GEODE7643_Synthemistidae_Palaeosynthemis_gracilenta	3.41237113	4.05882353	Flyer	338
GEODE7680_Synthemistidae_Palaeosynthemis_cervula	3.41237113	4.05882353	Flyer	662
GEODE7737_Synthemistidae_Choristhemis_flavoterminata	2.92328042	3.89768977	Flyer	927
GEODE7792_Synthemistidae_Eusynthemis_nigra	3.11174242	4.05437352	Flyer	930
OD1311_Synthemistidae_Parasynthemis_regina	3.501930502	4.213270142	Flyer	908
RMNH.5011432_Synthemistidae_Tonyosynthemis_claviculata	2.88852459	3.85294118	Flyer	929
RMNH.INS.500525_Synthemistidae_Palaeosynthemis_primigenia	3.41237113	4.05882353	Flyer	925
RMNH.INS.504910_Synthemistidae_Synthemis_eustalacta	2.98701299	3.92434211	Flyer	923
**Tatocnemididae**				
OD1137_Tatocnemididae_Tatocnemis_malgassica	5.33333333	3.92982456	Percher	927
**Thaumatoneuridae**				
GEODE7874_Thaumatoneuridae_Paraphlebia_duodecima	3.55128205	4.69333333	Percher	932
OD1135_Thaumatoneuridae_Paraphlebia_sp.	3.55128205	4.69333333	Percher	926
OD1141_Thaumatoneuridae_Thaumatoneura_inopinata	3.333333333	3.611570248	Percher	937

Supplemental Table 3. Genus level averages for both fore- and hindwing aspect ratios, genus level designation for flight behavior (flyer versus percher), and AHE loci recovery for each sample.

Genus	**Number of scans**
Acanthallagma	1
Acisoma	5
Acrogomphus	1
Aeschnophlebia	2
Aeschnosoma	1
Aeshna	133
Aethiothemis	4
Aethriamanta	3
Agriogomphus	3
Agriomorpha	1
Agrionoptera	4
Agyrtacantha	6
Akrothemis	1
Allopetalia	1
Amanipodagrion	1
Amphiaeschna	1
Amphigomphus	4
Amphithemis	1
Anaciaeschna	19
Anax	40
Andaeschna	1
Anisogomphus	4
Anormogomphus	1
Anotogaster	4
Antidythemis	1
Antiopodophlebia	1
Antipodochlora	1
Antipodogomphus	1

Aphylla	34
Arabicnemis	1
Archaeogomphus	4
Archaeophlebia	1
Archaeophya	2
Archaeosynthemis	5
Archilestes	1
Archineura	1
Argirothemis	1
Arigomphus	81
Armagomphus	1
Arrhenocnemis	1
Asiagomphus	8
Atocorneura	4
Atrocalopteryx	1
Austroaeschna	9
Austroceonagrion	1
Austrocordulia	9
Austroepigomphus	4
Austrogomphus	8
Austrogynacantha	1
Austropetalia	5
Austrophlebia	1
Austrophya	1
Austrosynthemis	4
Austrothemis	1
Basiaeschna	27
Bironides	2
Boyeria	11
Brachydiplax	2
Brachygonia	2
Brachymesia	22

Brachythemis	5
Brachytron	1
Bradinopyga	5
Brechmorhoga	5
Burmagomphus	4
Cacoides	1
Camacinia	3
Cannaphila	4
Castoraeschna	1
Celebophlebia	1
Celithemis	150
Cephalaeschna	8
Ceratogomphus	3
Ceylonosticta	1
Chalcolestes	1
Chalcostephia	1
Chalybeothemis	2
Chlorogomphus	9
Choristhemis	4
Copera	1
Cordulegaster	90
Cordulephya	3
Cordulia	8
Corduliochlora	1
Cornigomphus	1
Coryphaeschna	25
Cratilla	3
Crenigomphus	4
Crocothemis	3
Cyanocnemis	1
Cyanogomphus	2
Cyclogomphus	1

Cyclophaea	1
Dasythemis	2
Davidius	4
Dendroaeschna	1
Diaphlebia	1
Diastatomma	2
Diastatops	13
Didymops	6
Diplacina	4
Diplacodes	5
Disparocypha	1
Dorocordulia	17
Dromaeschna	4
Dromogomphus	45
Dythemis	21
Ebegomphus	1
Elasmothemis	11
Eleuthemis	1
Elga	3
Epiaeschna	22
Epiophlebia	2
Epitheca	130
Epophthalmia	4
Erpetogomphus	57
Erythemis	175
Erythrodiplax	107
Eusynthemis	5
Euthygomphus	1
Fukienoogomphus	2
Fylgia	1
Gatrogomphus	1
Gomphaeschna	17

Gomphidia	7
Gomphidictinus	2
Gomphoides	2
Gomphomacromia	4
Gomphurus	172
Guadalca	1
Gynacantha	30
Gynacanthaeschna	1
Gynothemis	2
Hadrothemis	5
Hagenius	17
Heliaeschna	8
Heliogomphus	6
Helocordulia	13
Hemicordulia	5
Hemigomphus	8
Hemistigma	1
Hesperocordulia	2
Heteronaias	1
Heterophaea	1
Huonia	5
Hydrobasileus	6
Hylaeargia	1
Hylaeothemis	3
Hylogomphus	62
Hypothemis	1
Ictinogomphus	4
Idiataphe	1
Idiataphe	1
Idionyx	8
Idomacromia	2
Indaeschna	2

Indothemis	2
Isoaeshna	1
Isomma	1
Labrogomphus	1
Ladona	55
Lamelligomphus	6
Lanthanusa	2
Lanthrecista	1
Lanthus	21
Lathrocordulia	2
Lauromacromia	1
Leptocnemis	1
Leptogomphus	8
Lestinogomphus	2
Leucorrhinia	70
Libellula	343
Libyogomphus	1
Lindenia	1
Luzonobasis	1
Lyriothemis	8
Macrocnemis	1
Macrodiplax	9
Macrogomphus	8
Macromia	39
Macromidia	8
Macrothemis	43
Malgassophlebia	2
Matronoides	1
Mattigomphus	1
Megalogomphus	4
Melligomphus	4
Merogomphus	3

Mesocnemis	1
Metacnemis	1
Metagrion	1
Metaphya	1
Miathryia	16
Micrathyria	84
Microgomphus	2
Micromacromia	3
Micromidia	8
Microtrigonia	1
Miniargiolestes	1
Miocora	1
Misagria	2
Nannodiplax	1
Nannophlebia	8
Nannophya	5
Nannophyopsis	1
Nannothemis	20
Nasiaeschna	14
Navicordulia	1
Neallogaster	1
Nehalennia	1
Neocordulia	1
Neodythemis	6
Neogomphus	3
Neopetalia	1
Neophya	1
Nephepeltia	7
Nepogomphoides	1
Nepogomphus	1
Nesciothemis	5
Nesobasis	1

Nesocordulia	1
Nesogonia	1
Nesolestes	1
Nesoxenia	4
Neuraeschna	4
Neurocordulia	7
Neurogomphus	6
Neurothemis	5
Nihonogomphus	4
Notiothemis	4
Notoaeschna	1
Notogomphus	8
Notolibellula	1
Nychogomphus	11
Octogomphus	9
Oligoaeschna	7
Oligoclada	5
Olpogastra	1
Ophiogomphus	88
Oplonaeschna	2
Orchithemis	4
Oreaeschna	1
Orientogomphus	1
Orthemis	36
Orthetrum	1
Oxygastra	4
Oxythemis	4
Pachydiplax	68
Pacificothemis	2
Palaeosynthemis	2
Palpopleura	5
Paltothemis	14

Pantala	86
Paracnemis	1
Paracordulia	2
Paragomphus	4
Parasynthemis	1
Parazyxomma	1
Pentaphlebia	1
Pentathemis	3
Periaeschna	6
Pericnemis	2
Perigomphus	1
Perissogomphus	1
Perissolestes	1
Perithemis	79
Peruviogomphus	1
Phaenandrogomphus	1
Phaenandrogomphus	3
Phanogomphus	234
Phenes	1
Philoganga	1
Philogenia	2
Philosina	1
Phyllocycla	17
Phyllogomphoides	29
Phyllogomphus	1
Phyllomacromia	2
Phyllopetalia	2
Pinheyschna	1
Plagulibasis	1
Planaeschna	8
Planiplax	2
Plathemis	88

Plattycantha	2
Platystigma	1
Pleiogomphus	1
Podopteryx	1
Polycanthagyna	7
Pornothemis	1
Porpax	4
Potamarcha	1
Procordulia	7
Progomphus	62
Protolestes	1
Protorthemis	1
Pseudocordulia	2
Pseudoleon	22
Pseudothemis	4
Pyrrhargiolestes	1
Raphismia	1
Remartinia	1
Rhinagrion	1
Rhinoneura	1
Rhionaeschna	4
Rhipidolestes	1
Rhodopygia	3
Rhodothemis	4
Rhyacocnemis	1
Rhyothemis	5
Rialla	1
Rimanella	1
Saraeschna	1
Scalmogomphus	2
Scapanea	1
Selysiothemis	1

Shaogomphus	1
Sieboldius	1
Sinictinogomphus	1
Sinogomphus	1
Somatochlora	55
Spesbona	1
Spinaeschna	1
Staurophlebia	1
Stenagrion	1
Stenocnemis	1
Stenocypha	2
Stenogomphurus	7
Stylogomphus	18
Stylurus	38
Sympeca	1
Sympetrum	151
Syncordulia	2
Synlestes	1
Synthemiopsis	2
Synthemis	7
Tachopteryx	10
Tanypteryx	3
Tapeinothemis	1
Tauriphila	21
Telephlebia	7
Telosticta	1
Tetracanthagyna	1
Tetrathemis	3
Thalassothemis	1
Thaumatagrion	1
Thaumatonerua	1
Tholymis	9

Tonyosynthemis	3
Tramea	85
Tricanthagyna	13
Trigomphus	1
Trithemis	5
Tyriobapta	2
Uracis	9
Uropetala	2
Urothemis	4
Wahnesia	1
Watanabeopetalia	2
Williamsonia	2
Xanthocnemis	1
Zenithoptera	4
Zygonyx	5
Zyxomma	5

Supplemental Table 4. The number of wing scans conducted for each genus. For more details on our wing scan process and access to the wing scan database, please see the TOWD project (https://digitizingdragonflies.org/).

Sample ID	Nearctic	Neotropics	Palaearctic	Afrotropics	Madagascar & Seychelles	Indo-Malayan	Wallacea	Australasia	Oceania
GEODE7682_Aeschnosomatidae_Aeschnosoma_forcipula		x							
OD1158_Aeschnosomatidae_Pentathemis_sp								x	
GEODE17607_Aeshnidae_Andaeschna_rufipes		x							
GEODE17609_Aeshnidae_Epiaeschna_heros	x								

GEODE17610_Aeshnidae_Gomphaeschna_furcillata	x								
GEODE17614_Aeshnidae_Oreaeschna_dictatrix								x	x
GEODE7669_Aeshnidae_Amphiaeschna_ampla						x			
GEODE7690_Aeshnidae_Plattycantha_cornuta								x	
GEODE7691_Aeshnidae_Heliaeschna_crassa				x		x	x		
GEODE7740_Aeshnidae_Rhionaeschna_multicolor	x	x							
OD1303_Aeshnidae_Gynacanthaeschna_sikkima						x			
OD1316_Aeshnidae_Gynacantha_gracilis	x	x	x	x	x	x	x	x	x
OD830_Aeshnidae_Anax_walsinghami	x	x	x	x	x	x	x	x	x
RMNH.5011576_Aeshnidae_Austrogynacantha_heterogena								x	x
RMNH.INS.502232_Aeshnidae_Gynacantha_victoriae	x	x	x	x	x	x	x	x	x
RMNH.INS.504908_Aeshnidae_Telephlebia_godeffroyi								x	
RMNH.INS.504941_Aeshnidae_Notoaeschna_sagittata								x	
RMNH.INS.504965_Aeshnidae_Acanthaeschna_victoria								x	
RMNH.INS.505016_Aeshnidae_Anax_imperator	x	x	x	x	x	x	x	x	x
RMNH.INS.505017_Aeshnidae_Isoaeshna_isoceles			x						

RMNH.INS.506912_Aeshnidae_Indaeschna_grubaueri			x			x	x		
RMNH.INS.507402_Aeshnidae_Brachytron_pratense			x			x			
RMNH.INS.507528_Aeshnidae_Boyeria_cretensis	x		x			x			
RMNH.INS.509145_Aeshnidae_Cephalaeschna_obversa						x			
TM170_Aeshnidae_Remartinia_luteipennis	x	x							
TM392_Aeshnidae_Rhionaeschna_joannisi	x	x							
TM581_Aeshnidae_Andaeschna_occidentalis		x							
OD1133_Amanipodagrionidae_Amanipodagrion_gilliesi				x					
OD1148_Amphipterygidae_Amphipteryx_agrioides		x							
GEODE7678_Argiolestidae_Luzonargiolestes_baltazarae							x		
GEODE7716_Argiolestidae_Griseargiolestes_albescens								x	
GEODE7820_Argiolestidae_Archiargiolestes_pusillissimus								x	
GEODE7821_Argiolestidae_Eoargiolestes_ochraceus								x	
GEODE7822_Argiolestidae_Nesolestes_radama					x				
OD1083_Argiolestidae_Austroargiolestes_icteromelas								x	
OD1309_Argiolestidae_Nesolestes_sp					x				

OD162_Argiolestidae_Caledargiolestes_uniseries								x	
RMNH.5008334_Argiolestidae_Podolestes_orientalis						x			
RMNH.INS.500397_Argiolestidae_Allolestes_maclachlanii					x				
RMNH.INS.500853_Argiolestidae_Eoargiolestes_ochraceus								x	
RMNH.INS.500854_Argiolestidae_Trineuragrion_percostale								x	
RMNH.INS.500862_Argiolestidae_Podopteryx_selysi								x	
RMNH.INS.501973_Argiolestidae_Argiolestes_muller							x	x	
RMNH.INS.501990_Argiolestidae_Pyrrhargiolestes_aulicus								x	
RMNH.INS.502075_Argiolestidae_Metagrion_ornatum								x	
RMNH.INS.502077_Argiolestidae_Miniargiolestes_minimus								x	
RMNH.INS.502084_Argiolestidae_Archiargiolestes_parvulus								x	
RMNH.INS.503407_Argiolestidae_Caledopteryx_sarasini								x	
RMNH.INS.506145_Argiolestidae_Neurolestes_trinervis				x					
RMNH.INS.543752_Argiolestidae_Wahnesia_kirbyi								x	
GEODE17873_Austrocorduliidae_Lathrocordulia_metallica								x	

GEODE7744_Austrocorduliidae_Micromidia_convergens								x	
GEODE7791_Austrocorduliidae_Hesperocordulia_berthoudi								x	
OD1172_Austrocorduliidae_Austrocordulia_refracta								x	
OD1310_Austrocorduliidae_Cordulephya_pygmaea								x	
GEODE17604_Austropetaliidae_Austropetalia_patricia								x	
OD1112_Austropetaliidae_Phyllopetalia_apollo		x							
OD1115_Austropetaliidae_Hypopetalia_pestilens		x							
GEODE7705_Calopterygidae_Noguchiphaea_yoshikoae						x			
GEODE7719_Calopterygidae_Calopteryx_aequabilis	x		x						
GEODE7721_Calopterygidae_Neurobasis_chinensis						x	x	x	
GEODE7748_Calopterygidae_Mnesarete_williamsoni		x							
GEODE7779_Calopterygidae_Mnesarete_cupraea		x							
GEODE7780_Calopterygidae_Hetaerina_caja	x	x							
GEODE7823_Calopterygidae_Bryoplathanon_globifer		x							
OD102_Calopterygidae_Matrona_basilaris			x			x			

OD1125_Calopterygidae_Neurobasis_ianthinipennis						x	x	x	
OD1170_Calopterygidae_Hetaerina_americana	x	x							
OD126_Calopterygidae_Vestalis_apicalis						x	x		
OD976_Calopterygidae_Ormenophlebia_imperatrix		x							
RMNH.INS.501070_Calopterygidae_Echo_modesta						x			
RMNH.INS.502054_Calopterygidae_Caliphaea_confusa						x			
RMNH.INS.502132_Calopterygidae_Phaon_iridipennis				x	x				
RMNH.INS.503116_Calopterygidae_Sapho_fumosa				x					
RMNH.INS.505747_Calopterygidae_Vestalaria_smaragdina						x			
RMNH.INS.505806_Calopterygidae_Psolodesmus_kuroiwae			x			x			
RMNH.INS.507722_Calopterygidae_Matronoides_cyaneipennis						x			
RMNH.INS.507799_Calopterygidae_Vestalis_amaryllis						x	x		
RMNH.INS.508607_Calopterygidae_Mnais_mneme			x			x			
RMNH.INS.508616_Calopterygidae_Atrocalopteryx_melli			x			x			
RMNH.INS.508674_Calopterygidae_Archineura_incarnata						x			

RMNH.INS.508844_Calopterygidae_Umma_longistigma				x					
GEODE7666_Chlorocyphidae_Heliocypha_bisignata						x			
GEODE7667_Chlorocyphidae_Libellago_greeni						x	x	x	
GEODE7672_Chlorocyphidae_Cyrano_unicolor							x		
GEODE7675_Chlorocyphidae_Disparocypha_biedermanni							x		
GEODE7812_Chlorocyphidae_Heliocypha_perforata						x			
OD120_Chlorocyphidae_Heliocypha_biseriata						x			
OD134_Chlorocyphidae_Libellago_semiopaca						x	x	x	
OD35_Chlorocyphidae_Rhinocypha_tincta						x	x	x	
RMNH.INS.500989_Chlorocyphidae_Libellago_aurantiaca						x	x	x	
RMNH.INS.501024_Chlorocyphidae_Sundacypha_petiolata						x			
RMNH.INS.505345_Chlorocyphidae_Platycypha_caligata				x					
RMNH.INS.505736_Chlorocyphidae_Aristocypha_fenestrella						x			
RMNH.INS.506163_Chlorocyphidae_Africocypha_lacuselephantum				x					
RMNH.INS.507707_Chlorocyphidae_Rhinoneura_villosipes						x			

RMNH.INS.508833_Chlorocyphidae_Stenocypha_gracilis				x					
GEODE7689_Chlorogomphidae_Chlorogomphus_spec			x			x	x		
OD1315_Chlorogomphidae_Chlorogomphus_auratus			x			x	x		
RMNH.INS.508528_Chlorogomphidae_Watanabeopetalia_usignata						x			
GEODE2711_Coenagrionidae_Luzonobasis_glauca							x		
GEODE2758_Coenagrionidae_Plagulibasis_ciliata								x	
GEODE7697_Coenagrionidae_Protoneura_tenuis		x							
GEODE7725_Coenagrionidae_Mecistogaster_linearis	x	x							
GEODE7741_Coenagrionidae_Psaironeura_tenuissima		x							
GEODE7749_Coenagrionidae_Amphiagrion_abbreviatum	x								
GEODE7753_Coenagrionidae_Telebasis_salva	x	x							
GEODE7758_Coenagrionidae_Acanthagrion_adustum	x	x							
GEODE7778_Coenagrionidae_Metaleptobasis_bicornis		x							
GEODE7781_Coenagrionidae_Phasmoneura_exigua		x							
GEODE7782_Coenagrionidae_Tuberculobasis_yanomami		x							

GEODE7783_Coenagrionidae_Telebasis_demarara	x	x							
GEODE7784_Coenagrionidae_Epipotoneura_nehalennia		x							
GEODE7785_Coenagrionidae_Inpabasis_rosea		x							
GEODE7786_Coenagrionidae_Acanthallagma_luteum		x							
GEODE7794_Coenagrionidae_Microstigma_anomalum		x							
GEODE7819_Coenagrionidae_Aeolagrion_dorsale		x							
GEODE7826_Coenagrionidae_Aceratobasis_macilenta		x							
GEODE7827_Coenagrionidae_Amphiallagma_parvum						x			
GEODE7828_Coenagrionidae_Anisagrion_allopterum		x							
GEODE7829_Coenagrionidae_Antiagrion_grinbergsi		x							
GEODE7830_Coenagrionidae_Apanisagrion_lais	x	x							
GEODE7832_Coenagrionidae_Caliagrion_billinghursti								x	
GEODE7833_Coenagrionidae_Calvertagrion_mauffrayi		x							
GEODE7835_Coenagrionidae_Cyanallagma_angelae		x							
GEODE7836_Coenagrionidae_Diceratobasis_melanogaster		x							

GEODE7839_Coenagrionidae_Telagrion_longum		x							
GEODE7841_Coenagrionidae_Peristicta_aeneoviridis		x							
GEODE7844_Coenagrionidae_Mesamphiagrion_demarmelsi		x							
GEODE7848_Coenagrionidae_Oxyagrion_hermosae		x							
GEODE7852_Coenagrionidae_Homeoura_lindneri		x							
GEODE7854_Coenagrionidae_Leptobasis_melinogaster	x	x							
GEODE7857_Coenagrionidae_Platystigma_astictum		x							
GEODE7879_Coenagrionidae_Forcepsioneura_sancta		x							
GEODE7880_Coenagrionidae_Denticulobasis_dunklei		x							
GEODE7882_Coenagrionidae_Andinagrion_peterseni		x							
OD103_Coenagrionidae_Neoneura_mariana	x	x							
OD105_Coenagrionidae_Protoneura_capillaris		x							
OD1116_Coenagrionidae_Cyanallagma_sp		x							
OD136_Coenagrionidae_Papuagrion_occipitale								x	
RMNH.5007797_Coenagrionidae_Azuragrion_nigridorsum			x	x	x				

RMNH.5007810_Coenagrionidae_Africallagma_glaucum				x	x				
RMNH.5010307_Coenagrionidae_Proischnura_polychromatica				x					
RMNH.INS.229208_Coenagrionidae_Oreocnemis_phoenix				x					
RMNH.INS.500398_Coenagrionidae_Leptocnemis_cyanops					x				
RMNH.INS.501979_Coenagrionidae_Thaumatagrion_funereum								x	
RMNH.INS.503604_Coenagrionidae_Archibasis_viola						x	x	x	
RMNH.INS.503619_Coenagrionidae_Aciagrion_borneense			x	x		x	x	x	
RMNH.INS.503951_Coenagrionidae_Argiocnemis_rubescens					x	x	x	x	
RMNH.INS.504852_Coenagrionidae_Austrocoenagrion_lyelli								x	
RMNH.INS.505254_Coenagrionidae_Austrocnemis_splendida								x	
RMNH.INS.507703_Coenagrionidae_Stenagrion_dubium						x	x		
RMNH.INS.509787_Coenagrionidae_Coenagriocnemis_rufipes					x				
RMNH.INS.509800_Coenagrionidae_Coenagriocnemis_insularis					x				
RMNH.INS.557636_Coenagrionidae_Pericnemis_stictica						x	x		

TM305_Coenagrionidae_Acanthagrion_trilobatum	x	x							
TMCH16_Coenagrionidae_Protoneura_amatoria		x							
OD1164_Cordulegastridae_Cordulegaster_sp	x	x	x			x			
OD1304_Cordulegastridae_Neallogaster_hermionae			x			x			
RMNH.INS.508536_Cordulegastridae_Anotogaster_sieboldii			x			x			
GEODE17698_Corduliidae_Dorocordulia_lepida	x								
GEODE17699_Corduliidae_Dorocordulia_libera	x								
GEODE17703_Corduliidae_Epitheca_cynosura	x		x						
GEODE17704_Corduliidae_Epitheca_marginata	x		x						
GEODE17707_Corduliidae_Epitheca_semiaquea	x		x						
GEODE17711_Corduliidae_Guadalca_insularis								x	x
GEODE17712_Corduliidae_Helocordulia_selysii	x								
GEODE17713_Corduliidae_Helocordulia_uhleri	x								
GEODE17717_Corduliidae_Hemicordulia_fidelis			x	x	x	x	x	x	x
GEODE17720_Corduliidae_Hemicordulia_kalliste			x	x	x	x	x	x	x

GEODE17779_Corduliidae_Navicordulia_errans		x							
GEODE17781_Corduliidae_Neurocordulia_virginiensis	x								
GEODE17783_Corduliidae_Neurocordulia_yamaskanensis	x								
GEODE17788_Corduliidae_Procordulia_jacksoniensis						x	x	x	x
GEODE17789_Corduliidae_Procordulia_moroensis						x	x	x	x
GEODE17793_Corduliidae_Neurocordulia_michaeli	x								
GEODE17832_Corduliidae_Somatochlora_cingulata	x		x			x			
GEODE7747_Corduliidae_Cordulia_shurtleffii	x		x						
GEODE7765_Corduliidae_Paracordulia_sericea		x							
OD1322_Corduliidae_Rialla_villosa		x							
OD146_Corduliidae_Cordulia_aenea	x		x						
OD83_Corduliidae_Hemicordulia_australiae			x	x	x	x	x	x	x
RMNH.5009497_Corduliidae_Corduliochlora_borisi			x						
RMNH.5011578_Corduliidae_Metaphya_tillyardi						x		x	
RMNH.INS.500518_Corduliidae_Procordulia_leopoldi						x	x	x	x
RMNH.INS.506657_Corduliidae_Heteronaias_heterodoxa							x		

OD151_Devadattidae_Devadatta_podolestoides						x	x		
RMNH.INS.501127_Devadattidae_Devadatta_argyoides						x	x		
GEODE7718_Dicteriadidae_Dicterias_atrosanguinea		x							
GEODE7788_Dicteriadidae_Heliocharis_amazona		x							
SRR1695233_Ephemeroptera_Ecdyonurus_insignis	x	x	x						
SRR921569_Ephemeroptera_Baetis_sp	x	x	x	x	x	x		x	
SRR921591_Ephemeroptera_Ephemera_danica	x	x	x	x		x			
SRR921596_Ephemeroptera_Eurylophella_sp	x		x		x				
SRR921608_Ephemeroptera_Isonychia_bicolor	x	x	x			x			
OD1076_Epiophlebiidae_Epiophlebia_superstes			x			x			
OD1302_Epiophlebiidae_Epiophlebia_laidlawi			x			x			
GEODE2756_Euphaeidae_Heterophaea_barbata							x		
GEODE7683_Euphaeidae_Anisopleura_lestoides						x			
GEODE7686_Euphaeidae_Euphaea_cardinalis			x			x	x		
GEODE7687_Euphaeidae_Cyclophaea_cyanifrons							x		
GEODE7694_Euphaeidae_Euphaea_refulgens			x			x	x		

GEODE7858_Euphaeidae_Cryptophaea_saukra						x			
OD132_Euphaeidae_Euphaea_ochracea			x			x	x		
OD99_Euphaeidae_Bayadera_brevicauda			x			x			
RMNH.5009494_Euphaeidae_Epallage_fatime			x			x			
RMNH.INS.501089_Euphaeidae_Dysphaea_dimidiata						x	x		
RMNH.INS.505757_Euphaeidae_Anisopleura_furcata						x			
RMNH.INS.505817_Euphaeidae_Cryptophaea_vietnamensis						x			
GEODE12753_Gomphidae_Lindenia_tetraphylla			x						
GEODE12754_Gomphidae_Mattigomphus_pinratani						x			
GEODE12755_Gomphidae_Perissogomphus_stevensi						x			
GEODE17903_Gomphidae_Anisogomphus_anderi			x			x			
GEODE17904_Gomphidae_Arigomphus_furcifer	x								
GEODE17909_Gomphidae_Zonophora_diversa		x							
GEODE17911_Gomphidae_Cacoides_latro		x							
GEODE17912_Gomphidae_Hagenius_brevistylus	x								
GEODE17913_Gomphidae_Fukienogomphus_promineus						x			

GEODE17914_Gomphidae_Shaogomphus_postocularis			x			x			
GEODE17915_Gomphidae_Peruviogomphus_pearsoni	x								
GEODE17917_Gomphidae_Octogomphus_specularis	x								
GEODE17919_Gomphidae_Gastrogomphus_abdominalis			x			x			
GEODE17922_Gomphidae_Melligomphus_ardens			x			x			
GEODE17923_Gomphidae_Merogomphus_longistigma						x			
GEODE17933_Gomphidae_Scalmogomphus_falcatus						x			
GEODE17935_Gomphidae_Anormogomphus_kiritshenkoi			x			x			
GEODE17936_Gomphidae_Phanogomphus_exilis	x								
GEODE7634_Gomphidae_Sinogomphus_telamon			x			x			
GEODE7681_Gomphidae_Phyllogomphoides_undulatus	x	x							
GEODE7692_Gomphidae_Phyllogomphoides_fuliginosus	x	x							
GEODE7710_Gomphidae_Ebegomphus_sp		x							
GEODE7731_Gomphidae_Ictinogomphus_pertinax			x	x		x	x	x	x
GEODE7732_Gomphidae_Gomphurus_externus	x								

GEODE7733_Gomphidae_Tragogomphus_ellioti				x					
GEODE7734_Gomphidae_Heliogomphus_scorpio						x	x		
GEODE7736_Gomphidae_Erpetogomphus_compositus	x	x							
GEODE7745_Gomphidae_Armagomphus_armiger								x	
GEODE7754_Gomphidae_Stenogomphurus_rogersi	x					x			
GEODE7763_Gomphidae_Archaeogomphus_nanus		x							
GEODE7772_Gomphidae_Ebegomphus_minutus		x							
GEODE7773_Gomphidae_Zonophora_batesi		x							
GEODE7774_Gomphidae_Desmogomphus_tigrivensis		x							
GEODE7775_Gomphidae_Agriogomphus_sylvicola		x							
GEODE7790_Gomphidae_Antipodogomphus_acolythus								x	
GEODE7818_Gomphidae_Aphylla_sp	x	x							
OD1314_Gomphidae_Ophiogomphus_morrisoni	x		x			x			
OD69_Gomphidae_Ictinogomphus_australis			x	x		x	x	x	x
OD85_Gomphidae_Austrogomphus_amphiclitus								x	
RMNH.5008090_Gomphidae_Ceratogomphus_pictus				x					

RMNH.5009376_Gomphidae_Onychogomphus_styx			x	x	x	x			
RMNH.5011399_Gomphidae_Stylurus_flavipes	x	x	x			x			
RMNH.5011415_Gomphidae_Austroepigomphus_praeruptus								x	
RMNH.5011429_Gomphidae_Hemigomphus_comitatus								x	
RMNH.5011478_Gomphidae_Austrogomphus_prasinus							H		
RMNH.INS.229080_Gomphidae_Macrogomphus_quadratus						x			
RMNH.INS.501184_Gomphidae_Phaenandrogomphus_asthenes						x	x		
RMNH.INS.501444_Gomphidae_Libyogomphus_christinae				x					
RMNH.INS.501518_Gomphidae_Microgomphus_camerunensis				x		x	x		
RMNH.INS.502394_Gomphidae_Lestinogomphus_congoensis				x					
RMNH.INS.502555_Gomphidae_Gomphidia_gamblesi			x	x		x	x		
RMNH.INS.502891_Gomphidae_Nepogomphoides_stuhlmanni				x					
RMNH.INS.503108_Gomphidae_Diastatomma_gamblesi				x					
RMNH.INS.503523_Gomphidae_Sieboldius_japponicus			x			x			

RMNH.INS.503524_Gomphidae_Acrogomphus_jubilaris						x	x		
RMNH.INS.504961_Gomphidae_Austrogomphus_ochraceus								x	
RMNH.INS.505397_Gomphidae_Neurogomphus_cocytius				x					
RMNH.INS.505653_Gomphidae_Notogomphus_praetorius				x					
RMNH.INS.505754_Gomphidae_Amphigomphus_somnuki						x			
RMNH.INS.506264_Gomphidae_Leptogomphus_coomansi			x			x	x		
RMNH.INS.506367_Gomphidae_Megalogomphus_borneensis						x			
RMNH.INS.507519_Gomphidae_Ophiogomphus_cecilia	x		x			x			
RMNH.INS.507929_Gomphidae_Phyllogomphus_annulus				x					
RMNH.INS.508012_Gomphidae_Paragomphus_genei			x	x	x	x	x		
RMNH.INS.508545_Gomphidae_Trigomphus_beatus			x			x			
RMNH.INS.508624_Gomphidae_Fukienogomphus_promineus						x			
RMNH.INS.508661_Gomphidae_Lamelligomphus_camelus			x			x			
RMNH.INS.508662_Gomphidae_Melligomphus_sinicus			x			x			
RMNH.INS.508663_Gomphidae_Nihono			x			x			

gomphus_semanticus									
RMNH.INS.508672_Gomphidae_Nepogomphus_walli						x	x		
RMNH.INS.508691_Gomphidae_Asiagomphus_hainanensis			x			x			
RMNH.INS.508700_Gomphidae_Labrogomphus_torvus						x			
RMNH.INS.509134_Gomphidae_Euthygomphus_koxingai						x			
RMNH.INS.509144_Gomphidae_Dubitogomphus_bicornutus			x			x			
RMNH.INS.509146_Gomphidae_Sinictinogomphus_clavatus			x			x			
RMNH.INS.509207_Gomphidae_Burmagomphus_divaricatus			x			x	x		
RMNH.INS.509920_Gomphidae_Lamelligomphus_castor			x			x			
RMNH.INS.509971_Gomphidae_Orientogomphus_minor						x			
RMNH.INS.554426_Gomphidae_Mastigogomphus_chapini				x					
RMNH.INS.554510_Gomphidae_Cornigomphus_guineensis				x					
RMNH.INS.559802_Gomphidae_Crenigomphus_hartmanni				x					
GEODE17894_Gomphomacromiidae_Gomphomacromia_nodisticta		x							
OD1169_Gomphomacromiidae_Gomphomacromia_paradoxa		x							

OD1074_Hemiphlebiidae_Hemiphlebia_mirabilis								x	
GEODE7742_Heteragrionidae_Oxystigma_petiolatum		x							
OD1126_Heteragrionidae_Dimeragrion_percubitale		x							
TM155_Heteragrionidae_Heteragrion_tatama		x							
TM404_Heteragrionidae_Heteragrion_erythrogastrum		x							
OD1146_Hypolestidae_Hypolestes_sp		x							
GEODE17864_Idionychidae_Idionyx_claudia						x	x		
GEODE17870_Idionychidae_Idionyx_optatus						x	x		
RMNH.INS.506617_Idionychidae_Idionyx_philippa						x	x		
GEODE17881_Idomacromiidae_Neocordulia_griphus		x							
NESOMASC_Idomacromiidae_Nesocordulia_mascarenica					x				
RMNH.5008153_Idomacromiidae_Syncordulia_gracilis				x					
RMNH.INS.503184_Idomacromiidae_Idomacromia_proavita				x					
RMNH.INS.507576_Idomacromiidae_Oxygastra_curtisii			x						
RU139_Idomacromiidae_Neocordulia_volxemi		x							
TM41_Idomacromiidae_Neocordulia_batesi		x							

GEODE7860_Isostictidae_Austrosticta_soror								x	
OD1277_Isostictidae_Neosticta_fraseri								x	
OD1298_Isostictidae_Cnemisticta_latilobata								x	
OD1312_Isostictidae_Labidiosticta_vallisi								x	
OD159_Isostictidae_Isosticta_robustior								x	
OD55_Isostictidae_Rhadinosticta_simplex								x	
RMNH.5011475_Isostictidae_Oristicta_filicicola								x	
RMNH.5011582_Isostictidae_Tanymecosticta_leptalea							x	x	
RMNH.INS.229243_Isostictidae_Selysioneura_capreola							x	x	
GEODE7771_Lauromacromiidae_Lauromacromia_dubitalis		x							
RU123_Lauromacromiidae_Lauromacromia_bedei		x							
RU126_Lauromacromiidae_Lauromacromia_picinguaba		x							
GEODE7665_Lestidae_Indolestes_albicaudus			x			x	x	x	x
GEODE7728_Lestidae_Austrolestes_leda								x	x
GEODE7730_Lestidae_Archilestes_grandis	x	x							
GEODE7813_Lestidae_Lestes_plagiatus	x	x	x	x	x	x	x	x	x

OD1127_Lestidae_Orolestes_octomaculatus						x			
OD122_Lestidae_Sympecma_paedisca			x			x			
OD1278_Lestidae_Indolestes_cyaneus			x			x	x	x	x
OD1321_Lestidae_Lestes_disjunctus	x	x	x	x	x	x	x	x	x
OD63_Lestidae_Austrolestes_annulosus								x	x
RMNH.INS.507448_Lestidae_Chalcolestes_viridis			x						
OD157_Lestoideidae_Lestoidea_conjuncta								x	
OD43_Lestoideidae_Diphlebia_coerulescens								x	
RMNH.5011424_Lestoideidae_Diphlebia_euphoeoides								x	
GEODE12746_Libellulidae_Akrothemis_risi								x	
GEODE12747_Libellulidae_Amphithemis_curvistyla						x			
GEODE12748_Libellulidae_Celebophlebia_dactylogastra							x		
GEODE12749_Libellulidae_Celebothemis_delecollei							x		
GEODE17725_Libellulidae_Orthemis_macrostigma	x	x							x
GEODE17728_Libellulidae_Orthemis_sulphurata	x	x							x
GEODE17755_Libellulidae_Anatya_januaria		x							

GEODE17756_Libellulidae_Antidythemis_trameiformis		x							
GEODE17757_Libellulidae_Archaeophlebia_martini					x				
GEODE17758_Libellulidae_Atoconeura_kenya				x					
GEODE17761_Libellulidae_Boninthemis_insularis									x
GEODE17929_Libellulidae_Scapanea_frontalis		x							
GEODE7612_Libellulidae_Cannaphila_insularis	x	x							
GEODE7628_Libellulidae_Rhodopygia_hinei		x							
GEODE7638_Libellulidae_Nannophlebia_axiagasta							x	x	
GEODE7650_Libellulidae_Diplacina_spec							x	x	
GEODE7655_Libellulidae_Diplacina_persephone							x	x	
GEODE7659_Libellulidae_Microtrigonia_gomphoides								x	
GEODE7676_Libellulidae_Misagria_calverti		x							
GEODE7698_Libellulidae_Erythemis_credula	x	x							
GEODE7699_Libellulidae_Erythrodiplax_umbrata	x	x							
GEODE7700_Libellulidae_Oligoclada_sp		x							
GEODE7701_Libellulidae_Agrionoptera_insignis			x			x	x	x	x

GEODE7702_Libellulidae_Uracis_fastigiata		x							
GEODE7707_Libellulidae_Elga_sp		x							
GEODE7708_Libellulidae_Plathemis_subornata	x								
GEODE7709_Libellulidae_Rhyothemis_notata			x	x	x	x	x	x	x
GEODE7711_Libellulidae_Celithemis_eponina	x								
GEODE7712_Libellulidae_Perithemis_sp	x	x							
GEODE7714_Libellulidae_Brachythemis_leucosticta			x	x	x	x	x		
GEODE7735_Libellulidae_Ladona_julia	x								
GEODE7738_Libellulidae_Brechmorhoga_mendax	x	x							
GEODE7739_Libellulidae_Perithemis_tenera	x	x							
GEODE7746_Libellulidae_Paltothemis_lineatipes	x								
GEODE7756_Libellulidae_Pachydiplax_longipennis	x								
GEODE7759_Libellulidae_Nannophya_australis			x			x		x	x
GEODE7760_Libellulidae_Austrothemis_nigrescens								x	
GEODE7761_Libellulidae_Tauriphila_argo	x	x							
GEODE7762_Libellulidae_Macrothemis_nobilis	x	x							

GEODE7764_Libellulidae_Miathyria_marcella	x	x							
GEODE7766_Libellulidae_Zenithoptera_fasciata		x							
GEODE7767_Libellulidae_Nephepeltia_phryne		x							
GEODE7768_Libellulidae_Elasmothemis_williamsoni		x							
GEODE7769_Libellulidae_Argyrothemis_argentea		x							
GEODE7770_Libellulidae_Gynothemis_pumilia		x							
GEODE7776_Libellulidae_Micrathyria_didyma	x	x							
GEODE7777_Libellulidae_Dythemis_nigra	x	x							
GEODE7798_Libellulidae_Palpopleura_lucia			x	x	x	x			
GEODE7800_Libellulidae_Diplacodes_trivialis			x	x	x	x	x	x	x
GEODE7801_Libellulidae_Crocothemis_servilia	x	x	x	x	x	x	x	x	x
GEODE7802_Libellulidae_Aethriamanta_rezia				x	x	x	x	x	x
GEODE7803_Libellulidae_Diastatops_obscura		x							
GEODE7804_Libellulidae_Tetrathemis_fraseri				x	x	x	x	x	
GEODE7805_Libellulidae_Acisoma_trifidum			x	x	x	x	x		

GEODE7806_Libellulidae_Orthetrum_julia			x	x	x	x	x	x	x
GEODE7807_Libellulidae_Tramea_transmarina	x	x	x	x	x	x	x	x	x
GEODE7815_Libellulidae_Urothemis_assignata			x	x	x	x	x	x	
OD107_Libellulidae_Pantala_flavescens	x	x	x	x	x	x	x	x	x
OD1091_Libellulidae_Brachythemis_contaminata			x	x	x	x	x		
OD1094_Libellulidae_Nannophlebia_alexia							x	x	
OD1095_Libellulidae_Sympetrum_corruptum	x	x	x	x	x	x			
OD1106_Libellulidae_Zygonyx_flavicosta			x	x	x	x	x		
OD1319_Libellulidae_Macrodiplax_cora	x	x	x	x	x	x	x	x	x
OD77_Libellulidae_Rhyothemis_graphiptera			x	x	x	x	x	x	x
OD82_Libellulidae_Brachydiplax_denticauda			x			x	x	x	x
RMNH.5007803_Libellulidae_Atoconeura_biordinata				x					
RMNH.5008306_Libellulidae_Neurothemis_fluctuans			x			x	x	x	x
RMNH.5011411_Libellulidae_Notolibellula_bicolor								x	
RMNH.5011412_Libellulidae_Microtrigonia_curvata								x	
RMNH.5011413_Libellulidae_Bironides_glochidion								x	

RMNH.INS.228976_Libellulidae_Tetrathemis_irregularis				x	x	x	x	x	
RMNH.INS.228999_Libellulidae_Raphismia_bispina						x	x	x	
RMNH.INS.500592_Libellulidae_Huonia_epinephela							x	x	
RMNH.INS.500644_Libellulidae_Protorthemis_coronata							x		x
RMNH.INS.501123_Libellulidae_Acisoma_panorpoides			x	x	x	x	x		
RMNH.INS.501152_Libellulidae_Pseudothemis_jorina			x			x			
RMNH.INS.501189_Libellulidae_Aethriamanta_brevipennis				x	x	x	x	x	x
RMNH.INS.501196_Libellulidae_Rhodothemis_rufa							x	x	x
RMNH.INS.501275_Libellulidae_Orchithemis_xanthosoma						x	x		
RMNH.INS.501284_Libellulidae_Brachygonia_oculata						x	x		
RMNH.INS.501296_Libellulidae_Tholymis_tillarga	x	x	x	x	x	x	x	x	x
RMNH.INS.501373_Libellulidae_Chalybeothemis_fluviatilis						x			
RMNH.INS.501377_Libellulidae_Zyxomma_petiolatum			x	x	x	x	x	x	x
RMNH.INS.501398_Libellulidae_Pornothemis_sp						x	x		
RMNH.INS.501662_Libellulidae_Eleuthemis_buettikoferi				x					

RMNH.INS.502121_Libellulidae_Oxythemis_phoenicosceles				x					
RMNH.INS.502150_Libellulidae_Chalcostephia_flavifrons				x	x				
RMNH.INS.502177_Libellulidae_Notiothemis_robertsi				x					
RMNH.INS.502225_Libellulidae_Aethiothemis_erythromelas				x	x				
RMNH.INS.502285_Libellulidae_Cyanothemis_simpsoni				x					
RMNH.INS.502287_Libellulidae_Micromacromia_camerunica				x					
RMNH.INS.502304_Libellulidae_Parazyxomma_flavicans				x	x				
RMNH.INS.502305_Libellulidae_Hadrothemis_camarensis				x					
RMNH.INS.502397_Libellulidae_Malgassophlebia_bispina				x	x				
RMNH.INS.502400_Libellulidae_Nesciothemis_farinosa			x	x					
RMNH.INS.502847_Libellulidae_Thermochoria_jeanneli				x					
RMNH.INS.503416_Libellulidae_Lyriothemis_cleis			x			x	x	x	
RMNH.INS.503419_Libellulidae_Nesoxenia_lineata						x	x	x	x
RMNH.INS.503939_Libellulidae_Hydrobasileus_croceus			x			x	x	x	x
RMNH.INS.503943_Libellulidae_Indothemis_limbata						x			

RMNH.INS.503957_Libellulidae_Potamarcha_congener						x	x	x	
RMNH.INS.503963_Libellulidae_Lathrecista_asiatica			x			x	x	x	x
RMNH.INS.503964_Libellulidae_Urothemis_signata			x	x	x	x	x	x	
RMNH.INS.504032_Libellulidae_Onychothemis_culminicola						x	x		
RMNH.INS.504039_Libellulidae_Orchithemis_pulcherrima						x	x		
RMNH.INS.504459_Libellulidae_Hemistigma_albipunctum				x	x				
RMNH.INS.504999_Libellulidae_Orthetrum_cancellatum			x	x	x	x	x	x	x
RMNH.INS.505354_Libellulidae_Trithetrum_navasi				x					
RMNH.INS.505362_Libellulidae_Crocothemis_erythraea	x	x	x	x	x	x	x	x	x
RMNH.INS.505380_Libellulidae_Olpogastra_lugubris				x	x				
RMNH.INS.505547_Libellulidae_Porpax_risi				x					
RMNH.INS.505588_Libellulidae_Diplacodes_lefebvrii			x	x	x	x	x	x	x
RMNH.INS.505652_Libellulidae_Bradinopyga_cornuta				x		x			
RMNH.INS.505880_Libellulidae_Tapeinothemis_boharti								x	x
RMNH.INS.506254_Libellulidae_Camacinia_gigantea						x	x	x	x

RMNH.INS.506290_Libellulidae_Cratilla_lineata						x	x	x	
RMNH.INS.507392_Libellulidae_Leucorrhinia_rubicunda	x		x						
RMNH.INS.507400_Libellulidae_Libellula_depressa	x	x	x			x			
RMNH.INS.507439_Libellulidae_Libellula_quadrimaculata	x	x	x			x			
RMNH.INS.507465_Libellulidae_Sympetrum_danae	x	x	x	x	x	x			
RMNH.INS.507492_Libellulidae_Nannodiplax_rubra								x	
RMNH.INS.507529_Libellulidae_Trithemis_annulata			x	x	x	x	x	x	
RMNH.INS.507551_Libellulidae_Selysiothemis_nigra			x			x			
RMNH.INS.507746_Libellulidae_Hylaeothemis_clementia						x			
RMNH.INS.507831_Libellulidae_Tyriobapta_torrida						x			
RMNH.INS.508001_Libellulidae_Zygonyx_torridus			x	x	x	x	x		
RMNH.INS.508447_Libellulidae_Palpopleura_sexmaculata			x	x	x	x			
RMNH.INS.508515_Libellulidae_Nannophyopsis_clara						x			
RMNH.INS.508797_Libellulidae_Neodythemis_afra				x	x				
RMNH.INS.509795_Libellulidae_Thalassothemis_marchali					x				

RMNH.INS.559789_Libellulidae_Zygonoides_fuelleborni				x	x				
TM207_Libellulidae_Brechmorhoga_vivax	x	x							
TM332_Libellulidae_Cannaphila_vibex	x	x							
TM342_Libellulidae_Macrothemis_pseudimitans	x	x							
TM409_Libellulidae_Rhodopygia_hinei		x							
TM532_Libellulidae_Erythemis_plebeja	x	x							
GEODE17874_Macromidiidae_Macromidia_genialis			x			x	x		
GEODE17876_Macromidiidae_Macromidia_kelloggi			x			x	x		
RMNH.INS.506247_Macromidiidae_Macromidia_fulva			x			x	x		
GEODE17622_Macromiidae_Macromia_astarte	x		x			x	x	x	
GEODE7715_Macromiidae_Phyllomacromia_picta				x	x				
OD1101_Macromiidae_Epophthalmia_elegans			x			x	x		
RMNH.INS.507575_Macromiidae_Macromia_splendens	x		x			x	x	x	
GEODE7863_Megapodagrionidae_Allopodagrion_contortum		x							
OD1281_Megapodagrionidae_Teinopodagrion_meridionale		x							
OD1183_Mesagrionidae_Mesagrion_leucorrhinum		x							

OD1077_Mesopodagrionidae_Mesopodagrion_sp						x			
OD1113_Neopetaliidae_Neopetalia_punctata		x							
RMNH.INS.502324_Neophyidae_Neophya_rutherfordi				x					
OD1139_Pentaphlebiidae_Pentaphlebia_mangana				x					
RMNH.INS.500093_Pentaphlebiidae_Pentaphlebia_stahli				x					
OD1120_Perilestidae_Perissolestes_remotus		x							
GEODE17940_Petaluridae_Petalura_litorea								x	
GEODE17943_Petaluridae_Uropetala_chiltoni								x	x
GEODE7743_Petaluridae_Tanypteryx_hageni	x		x						
OD1318_Petaluridae_Petalura_gigantea								x	
PD166_Petaluridae_Tanypteryx_pryeri	x		x						
OD1079_Philogangidae_Philoganga_vetusta						x			
OD1124_Philogeniidae_Philogenia_carrillica		x							
TM201_Philogeniidae_Philogenia_martae		x							
OD1119_Philosinidae_Rhinagrion_sp						x	x		
OD1130_Philosinidae_Philosina_buchi						x			
RMNH.INS.506835_Philosinidae_Rhinag						x	x		

rion_macrocephalum									
GEODE2652_Platycnemididae_Asthenocnemis_stephanodera							x		
GEODE7624_Platycnemididae_Disparoneura_apicalis						x			
GEODE7653_Platycnemididae_Esme_mudiensis						x			
GEODE7656_Platycnemididae_Nososticta_selysii						x	x	x	
GEODE7660_Platycnemididae_Nososticta_exul						x	x	x	
GEODE7668_Platycnemididae_Calicnemia_pulverulans						x			
GEODE7673_Platycnemididae_Calicnemia_erythromelas						x			
GEODE7724_Platycnemididae_Coeliccia_cyanomelas			x			x	x		
GEODE7727_Platycnemididae_Prodasineura_autumnalis						x	x		
GEODE7750_Platycnemididae_Copera_nyansana				x		x	x		
GEODE7796_Platycnemididae_Elattoneura_acuta				x		x			
GEODE7811_Platycnemididae_Copera_marginipes				x		x	x		
GEODE7864_Platycnemididae_Hylaeargia_magnifica								x	
GEODE7866_Platycnemididae_Lieftinckia_malaitae								x	x

GEODE7868_Platycnemididae_Rhyacocnemis_prothoracica								x	
OD1122_Platycnemididae_Prodasineura_croconota						x	x		
OD1123_Platycnemididae_Coeliccia_pyriformis			x			x	x		
OD1132_Platycnemididae_Onychargia_atrocyana						x	x		
OD1143_Platycnemididae_Mesocnemis_singularis			x	x					
OD1306_Platycnemididae_Elattoneura_glauca				x		x			
OD1317_Platycnemididae_Nososticta_solida						x	x	x	
OD148_Platycnemididae_Platycnemis_pennipes			x			x			
RMNH.5007870_Platycnemididae_Metacnemis_valida				x					
RMNH.5011406_Platycnemididae_Macrocnemis_gracilis								x	
RMNH.INS.229282_Platycnemididae_Paracnemis_alluaudi					x				
RMNH.INS.229291_Platycnemididae_Torrenticnemis_filicornis								x	
RMNH.INS.500162_Platycnemididae_Allocnemis_contraria				x					
RMNH.INS.500513_Platycnemididae_Lochmaeocnemis_malacodora								x	
RMNH.INS.500580_Platycnemididae_Palaiargia_charmosyna							x	x	

RMNH.INS.500639_Platycnemididae_Idiocnemis_obliterata								x	
RMNH.INS.501977_Platycnemididae_Arrhenocnemis_parvibullis								x	
RMNH.INS.501995_Platycnemididae_Cyanocnemis_aureofrons								x	
RMNH.INS.503929_Platycnemididae_Calicnemia_chaseni						x			
RMNH.INS.503944_Platycnemididae_Pseudocopera_ciliata			x			x			
RMNH.INS.505846_Platycnemididae_Arabicnemis_caerulea			x						
RMNH.INS.506232_Platycnemididae_Matticnemis_doi						x			
RMNH.INS.508201_Platycnemididae_Arabineura_khalidi			x						
RMNH.INS.508637_Platycnemididae_Indocnemis_orang						x			
RMNH.INS.508794_Platycnemididae_Stenocnemis_pachystigma				x					
RMNH.INS.559644_Platycnemididae_Spesbona_angusta				x					
GEODE7722_Platystictidae_Protosticta_satoi						x	x		
GEODE7787_Platystictidae_Palaemnema_brevignoni	x	x							
GEODE7871_Platystictidae_Sinosticta_sylvatica						x			

GEODE7876_Platystictidae_Ceylonosticta_lankanensis						x			
RMNH.5008397_Platystictidae_Telosticta_ulubaram						x	x		
RMNH.INS.506261_Platystictidae_Telosticta_bidayuh						x	x		
RMNH.INS.506492_Platystictidae_Sinosticta_ogatai						x			
RMNH.INS.507659_Platystictidae_Drepanosticta_actaeon						x	x	x	x
ANCH188_Polythoridae_Miocora_peraltica		x							
BOY03_Polythoridae_Cora_xanthostoma		x							
PP330_Polythoridae_Polythore_procera		x							
RMNH.INS.505717_Polythoridae_Chalcopteryx_rutilans		x							
TM154_Polythoridae_Polythore_gigantea		x							
UAE1_Polythoridae_Euthore_fassli		x							
GEODE7872_Priscagrionidae_Sinocnemis_henanensis						x			
OD1280_Priscagrionidae_Priscagrion_kiautai						x			
OD1136_Protolestidae_Protolestes_fickei					x				
OD1308_Protolestidae_Protolestes_kerckhoffae					x				
GEODE17883_Pseudocorduliidae_Pseudocordulia_circularis								x	

GEODE17884_Pseudocorduliidae_Pseudocordulia_elliptica								x	
OD1283_Pseudolestidae_Pseudolestes_mirabilis						x			
OD1085_Rhipidolestidae_Agriomorpha_xinglongensis						x			
OD1121_Rhipidolestidae_Agriomorpha_fusca						x			
OD96_Rhipidolestidae_Rhipidolestes_aculeatus			x			x			
RMNH.INS.502064_Rhipidolestidae_Rhipidolestes_truncatidens			x			x			
GEODE7704_Rimanellidae_Rimanella_arcana		x							
GEODE7723_Synlestidae_Chlorolestes_elegans				x					
GEODE7755_Synlestidae_Episynlestes_albicauda								x	
GEODE7873_Synlestidae_Phylolestes_ethelae		x							
OD1305_Synlestidae_Chlorolestes_umbratus				x					
OD81_Synlestidae_Synlestes_weyersii								x	
RMNH.5007835_Synlestidae_Ecchlorolestes_nylephtha				x					
RMNH.5011485_Synlestidae_Chorismagrion_risi								x	
RMNH.INS.508632_Synlestidae_Megalestes_heros						x			

RMNH.INS.508746_Synlestidae_Nubiolestes_diotima				x					
GEODE17885_Synthemistidae_Archaeosynthemis_leachii								x	
GEODE17887_Synthemistidae_Archaeosynthemis_orientalis								x	
GEODE17888_Synthemistidae_Archaeosynthemis_spiniger								x	
GEODE17889_Synthemistidae_Austrosynthemis_cyanitincta								x	
GEODE17891_Synthemistidae_Eusynthemis_guttata								x	
GEODE17896_Synthemistidae_Calesynthemis_campioni								x	
GEODE17901_Synthemistidae_Synthemis_tasmanica								x	
GEODE17902_Synthemistidae_Tonyosynthemis_ofarrelli								x	
GEODE7643_Synthemistidae_Palaeosynthemis_gracilenta							x	x	
GEODE7680_Synthemistidae_Palaeosynthemis_cervula							x	x	
GEODE7737_Synthemistidae_Choristhemis_flavoterminata								x	
GEODE7792_Synthemistidae_Eusynthemis_nigra								x	
OD1311_Synthemistidae_Parasynthemis_regina								x	
RMNH.5011432_Synthemistidae_Tonyosynthemis_claviculata								x	

RMNH.INS.500525_Synthemistidae_Palaeosynthemis_primigenia							x	x	
RMNH.INS.504910_Synthemistidae_Synthemis_eustalacta								x	
OD1137_Tatocnemididae_Tatocnemis_malgassica					x				
GEODE7874_Thaumatoneuridae_Paraphlebia_duodecima		x							
OD1135_Thaumatoneuridae_Paraphlebia_sp		x							
OD1141_Thaumatoneuridae_Thaumatoneura_inopinata		x							

Supplemental Table 5. Genus level biogeographic realm designations for each sample. The ‘x’ depicts the genus as present in that biogeographic realm, whereas a blank entry means the genus is not present.

CrownNodes	Age (Mya)	95% HPD CI	Geological Time Period
Paleoptera	316.8	(292.59, 331.78)	Carboniferous
Odonata	313.73	(290.78, 325.19)	Carboniferous
Zygoptera	278.35	(233.23, 319.18)	Permian
"Calopterygoidea"+Coenagrionoidea	212.98	(173.53, 244.67)	Triassic
Coenagrionoidea	160.53	(127.24, 188.54)	Jurassic
Lestoidea	149.15	(73.23, 231.38)	Jurassic
Argiolestidae	84.78	(63.64, 104.84)	Cretaceous
Calopterygidae	96.46	(70.51, 116.61)	Cretaceous
Chlorocyphidae	91.84	(70.14, 112.96)	Cretaceous
Coenagrionidae	122.6	(81.42, 143.29)	Cretaceous
Devadattidae	19.52	(6.36, 41.23)	Miocene
Dicteriadidae	32.58	(14.70, 50.24)	Oligocene
Euphaeidae	59.77	(43.26, 80.06)	Palaeocene
Heteragrionidae	35.07	(17.53, 57.84)	Oligocene

Isostictidae	53.14	(29.25, 87.43)	Palaeocene
Lestidae	89.25	(54.93, 148.33)	Cretaceous
Lestoideidae	39.17	(18.84, 65.96)	Eocene
Megapodagrionidae	27.88	(7.09, 55.81)	Oligocene
Pentaphlebiidae	26.21	(8.68, 45.40)	Oligocene
Philogeniidae	22.64	(7.64, 42.60)	Miocene
Philosinidae	34.69	(12.55, 62.44)	Eocene
Platycnemididae	106.63	(84.49, 132.32)	Cretaceous
Platystictidae	133.61	(48.93, 228.96)	Cretaceous
Platystictoidea	133.61	(48.93, 228.96)	Cretaceous
Polythoridae	48.88	(31.86, 81.42)	Eocene
Priscagrionidae	38.6	(7.48, 90.72)	Eocene
Protolestidae	28.43	(9.01, 52.70)	Eocene
Rhipidolestidae	40.62	(24.29, 66.34)	Eocene
Synlestidae	76.77	(41.41, 130.72)	Cretaceous
Thaumatoneuridae	26.6	(13.66, 44.54)	Oligocene
Epiprocta	290.08	(259.83, 312.93)	Permian
Epiophlebiidae	32.95	(6.35, 79.93)	Oligocene
Anisoptera	274.07	(244.76, 299.84)	Permian
Aeschnosomiidae	17.12	(5.96, 37.16)	Miocene
Aeshnidae	164.75	(113.45, 225.06)	Jurassic
Aeshnoidea	229.82	(163.04, 282.77)	Triassic
Austrocorduliidae	91.35	(34.75, 183.86)	Cretaceous
Austropetaliidae	59.95	(15.15, 145.87)	Palaeocene
Chlorogomphidae	52.79	(18.88, 111.62)	Palaeocene
Cordulegastridae	43.37	(23.30, 69.72)	Eocene
Cordulegastroidea	137.25	(89.66, 196.70)	Cretaceous
Corduliidae	112.87	(81.93, 163.87)	Cretaceous
Gomphoidea	233.99	(214.51, 256.32)	Triassic
Gomphomacromiidae	69.77	(13.14, 144.68)	Cretaceous
Idionychidae	43.89	(14.86, 77.24)	Eocene
Idomacromiidae	73.68	(34.64, 117.92)	Cretaceous
Lauromacromiidae	51.3	(15.76, 98.64)	Eocene
Libellulidae	106.96	(78.82, 142.27)	Cretaceous
Libelluloidea	220.3	(185.96, 251.63)	Triassic

Macromidiaiidae	45.84	(14.62, 88.40)	Eocene
Macromiidae	39.64	(18.82, 59.19)	Oligocene
Petaluroidea	152.8	(114.93, 205.79)	Cretaceous
Pseudocorduliidae	25.47	(6.84, 54.02)	Miocene
Synthemistidae	116.51	(69.24, 156.75)	Cretaceous
**StemNodes**	**Age (Mya)**	**95% HPD CI**	
Amanipodagrionidae+Mesopodagrionidae	45.75	(10.94, 97.54)	Eocene
Amphipterygidae+Devadattidae	29.47	(10.43, 51.24)	Miocene
Hypolestidae+Dicteriadidae	18.25	(7.52, 32.00)	Miocene
Mesagrionidae+(Hypolestidae+Dicteriadidae)	48.8	(23.42, 80.20)	Eocene
Philogangidae+Philosinidae	48.64	(20.57, 81.30)	Eocene
Pseudolestidae+(Lestoideidae+Euphaeidae)	84.22	(67.37, 100.33)	Cretaceous
Rimanellidae+Polythoridae	94.38	(56.44, 141.34)	Cretaceous
Tatocnemididae+(Pentaphlebiidae+Protolestidae)	53.65	(23.96, 85.94)	Eocene
Neopetaliidae+Cordulegastridae	85.75	(35.82, 143.33)	Cretaceous
Hemiphlebiidae+((Perilestidae+Synlestidae)+Lestidae)	149.15	(73.23, 231.38)	Jurassic
Perilestidae+Synlestidae	91.59	(49.03, 146.13)	Cretaceous
Neophyidae+((Libellulidae+Aeschnosomiidae)+Corduliidae)	143.6	(125.77, 181.59)	Jurassic

Supplemental Table 6. Divergence time estimates, 95% Credible Intervals (CI), and the geological time period in which divergence times occurred for the deeper level crown- and stem-nodes (i.e., ordinal, subordinal, superfamily, and family).

Lineage	Vicariance Events	Dispersal Events							Ratio Vicariance / Dispersal
		1-4	5	6	7	8	9	Total	
**Zygoptera**	**34**	**68**	**2**	**-**	**-**	**-**	**2**	**72**	**32/68**
Lestoidea	**5**	**2**	**1**	-	-	-	**2**	**5**	**50/50**
Hemiphlebidae	-	-	-	-	-	-	-	**-**	
Lestidae	1	1	1				2	**4**	**20/80**

Perilestidae	-	-	-	-	-	-	-	**-**	
Synlestidae	3	3	-	-	-	-	-	**3**	**50/50**
Platystictoidea	1	3	-	-	-	-	-	**3**	**25/75**
Platystictictidae	1	3	-	-	-	-	-	**3**	**25/75**
"Calopterygoidea"	**18**	**15**	-	-	-	-	-	**15**	**55/45**
Group 1	-	-	-	-	-	-	-	**-**	
Priscagrionidae	-	-	-	-	-	-	-	**-**	
Group 2	**2**	**2**	-	-	-	-	-	**2**	**50/50**
Tatocnemididae	-	-	-	-	-	-	-	**-**	
Pentaphlebidae	-	-	-	-	-	-	-	**-**	
Protolestidae	-	-	-	-	-	-	-	**-**	
Rimanellidae	-	-	-	-	-	-	-	**-**	
Polythoridae	-	-	-	-	-	-	-	**-**	
Mesagrionidae	-	-	-	-	-	-	-	**-**	
Dicteriadidae	-	-	-	-	-	-	-	**-**	
Hypolestidae	-	-	-	-	-	-	-	**-**	
Philogeniidae	-	-	-	-	-	-	-	**-**	
Group 3	**7**	**10**	-	-	-	-	-	**10**	**41/59**
Aminopodagrionidae	-	-	-	-	-	-	-	**-**	
Mesopodagrionidae	-	-	-	-	-	-	-	-	
Amphipterygidae	1	-	-	-	-	-	-	-	
Devadattidae	-	-	-	-	-	-	-	**-**	
Thaumatoneuridae	-	-	-	-	-	-	-	**-**	
Rhipolestidae	-	1	-	-	-	-	-	**1**	
Lestoideidae	-	-	-	-	-	-	-	**-**	
Euphaeidae	2	6	-	-	-	-	-	**6**	**25/75**
Group 4	**9**	**26**	-	-	-	-	-	**26**	**26/74**
Philogangidae	-	-	-	-	-	-	-	**-**	
Philosinidae	-	1	-	-	-	-	-	**1**	
Megapodagrionidae	-	-	-	-	-	-	-	**-**	
Argiolestidae	5	5	-	-	-	-	-	**5**	**50/50**
Chlorophycidae	1	5	-	-	-	-	-	**5**	**17/83**
Calopterygidae	2	11	-	-	-	-	-	**11**	**15/85**
Coenagrionoidea	**12**	**48**	**1**	-	-	-	-	**49**	**20/80**
Isostictidae	-	2	1	-	-	-	-	**3**	
Platycnecmidae	4	19	-	-	-	-	-	**19**	**17/83**

Coenagrionidae	8	26	-	-	-	-	-	**26**	**24/76**
**Epiprocta**	**43**	189	18	8	8	1	4	**228**	**16/84**
Epiophlebioidea	-	1	-	-	-	-	-	**1**	
Epiophlebidae	-	1	-	-	-	-	-	**1**	
Aeshnoidea	**5**	**12**	**3**	**1**	**1**	**-**	**2**	**19**	**21/79**
Austropetalidae	1		-	-	-	-	-	**0**	**100/0**
Aeshnidae	4	10	3	1	1	-	2	**17**	**19/81**
Petaluroidea	**1**	**2**	-	-	-	-	-	**2**	**33/67**
Petaluridae	1	2	-	-	-	-	-	**2**	**33/67**
Gomphoidea	**11**	**49**	**-**	**-**	**1**	**-**	**-**	**50**	**18/82**
Gomphidae	11	49	-	-	1	-	-	**50**	**18/82**
Cordulegastroidea	-	**5**	-	-	-	-	-	**5**	
Chlorogomphidae	-	1	-	-	-	-	-	**1**	
Cordulegastridae	-	2	-	-	-	-	-	**2**	
Neopetalidae	-	-	-	-	-	-	-	**0**	
Libelluloidea	**26**	**120**	**15**	**7**	**6**	**1**	**2**	**151**	**15/85**
Pseudocordulidae	-							**0**	
Gomphomacromiidae	-							**0**	
Synthemistidae	4	1						**1**	**80/20**
Austrocordulidae	-							**0**	
Idionychidae	-	1						**1**	
Macromiidae	1	1	2					**3**	**25/75**
Idomacromiidae	4	3						**3**	**57/43**
Lauromacromiidae	-							**0**	
Macromidiidae	-							**0**	
Neophyidea	-							**0**	
Corduliidae	3	10			2			**12**	**20/80**
Aeshnosomatidae	1	1						**1**	**50/50**
Libellulidae	13	103	13	7	4	1	2	**130**	**9/91**

Supplemental Table 7. The number of vicariance and dispersal events for each of the families, superfamilies, and suborders with the last column indicating the ratio between vicariance and dispersal events.
